# 1,2-Migration of oxamoyl-protected alcohols via reversible C–H sampling

**DOI:** 10.1093/nsr/nwag260

**Published:** 2026-05-07

**Authors:** Yinyan Su, Duoqing Wang, Ken Chen, Yan Xu

**Affiliations:** Beijing National Laboratory of Molecular Sciences, Key Laboratory of Bioorganic Chemistry and Molecular Engineering of Ministry of Education, College of Chemistry and Molecular Engineering, Peking University, Beijing 100871, China; Beijing National Laboratory of Molecular Sciences, Key Laboratory of Bioorganic Chemistry and Molecular Engineering of Ministry of Education, College of Chemistry and Molecular Engineering, Peking University, Beijing 100871, China; Beijing National Laboratory of Molecular Sciences, Key Laboratory of Bioorganic Chemistry and Molecular Engineering of Ministry of Education, College of Chemistry and Molecular Engineering, Peking University, Beijing 100871, China; Beijing National Laboratory of Molecular Sciences, Key Laboratory of Bioorganic Chemistry and Molecular Engineering of Ministry of Education, College of Chemistry and Molecular Engineering, Peking University, Beijing 100871, China

**Keywords:** reversible C–H sampling, oxamoyl protecting group, 1,2-acyloxy shift, hydrogen atom transfer, photocatalysis

## Abstract

Alcohols are ubiquitous in natural products and serve as versatile synthetic handles; however, the relocation of a hydroxyl group to an adjacent C–H site remains an underdeveloped yet highly valuable transformation. Herein, we report photocatalytic 1,2-translocation of oxamoyl-protected alcohols enabled by a reversible C–H sampling strategy. Utilizing a cooperative hydrogen atom transfer catalytic system, a range of carbon radicals are transiently generated, while the *β*-acyloxy alkyl radical selectively undergoes a 1,2-acyloxy shift to afford the rearranged product. The oxamoyl protecting group exhibits superior performance, likely accelerating the radical-acyloxy rearrangement while simultaneously enhancing *β*-hydrogen atom abstraction (HAA) probability. This redox-neutral methodology accommodates a broad substrate scope with predictable regio- and stereoselectivity, including the late-stage modification of complex bioactive molecules. Beyond facile hydrolytic removal, the oxamoyloxy handle can be directly converted into alkyl iodides, providing an additional entry point for further diverse functionalizations.

## Teaser

Using an oxamoyl protecting group, a reversible C–H sampling photocatalytic strategy enables 1,2-relocation of alcohols to adjacent unactivated C–H sites under mild conditions, with predictable site- and stereoselectivity.

## INTRODUCTION

**Figure 1.**
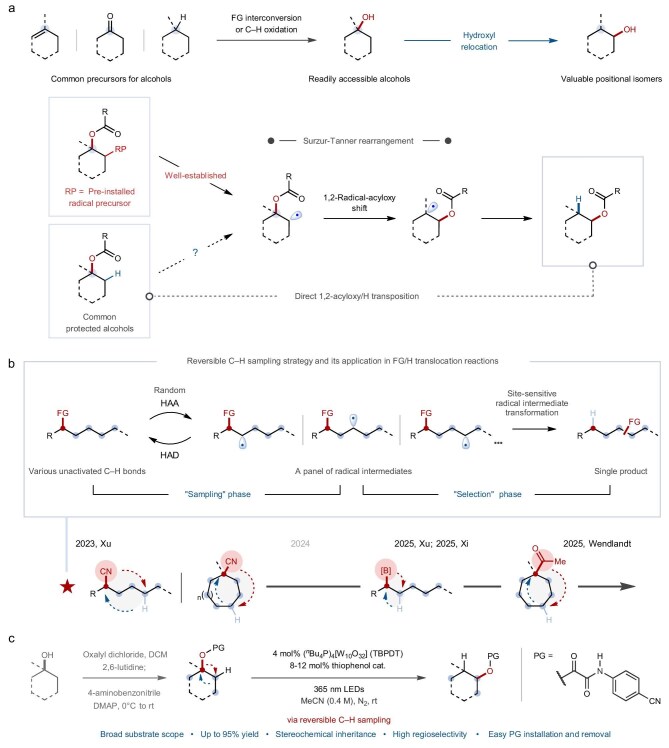
Hydroxyl relocation of alcohols. (a) Utility of hydroxyl relocation and inspiration from the Surzur–Tanner rearrangement. (b) Reversible C–H sampling strategy and its application in FG/H translocation reactions. (c) Overview of this work: 1,2-migration of oxamoyl-protected alcohols *via* reversible C–H sampling.

Hydroxyl groups are ubiquitous in natural products and advanced synthetic intermediates [1,2]. As both hydrogen-bonding donors and acceptors, their presence and positioning exert a profound influence on a molecule’s physical, chemical, and biological properties [3]. Given their versatile roles as oxygen nucleophiles or precursors for carbon electrophiles and radicals, hydroxyl groups also serve as adaptable synthetic handles in diverse transformations—ranging from classic esterification and Mitsunobu reactions to cutting-edge dehydroxylative C–C couplings [4,5]. From a retrosynthetic perspective, hydroxyl groups are commonly introduced either through reduction of or addition to carbonyl compounds, or through hydration or oxidation of unsaturated C–C bonds; alternatively, they may also be introduced *via* direct C–H oxidation, especially in naturally occurring skeletons or at a late stage [6,7]. Yet in both cases, the position where a hydroxyl group can be introduced is still limited by the location of its precursor functional group (FG) or the innate site-selectivity of C–H oxidation. As such, there is a natural need for methods to relocate existing hydroxyl groups, such as to an adjacent carbon. Conventionally, such a translocation could be achieved through a three-step sequence of dehydration, hydroboration, and oxidation [8,9]. Nevertheless, this approach often faces challenges in regio- and stereoselectivity, while requiring both reductive and oxidative conditions. Alternatively, the Surzur–Tanner rearrangement represents an intriguing 1,2-acyloxy shift of *β*-(acyloxy)alkyl radicals, capable of translocating an acyl-protected alcohol [10–13]; yet, its synthetic utility is largely constrained by the requirement for a pre-installed radical precursor at the *β*-carbon in the prior art [14,15]. As such, it would be highly attractive if a radical could be directly generated at the *β*-C–H site of a masked alcohol to induce a similar radical rearrangement, thus bypassing the need for pre-functionalization. Such a method could facilitate the late-stage diversification of hydroxyl-containing lead compounds and provide a new strategic manifold for accessing complex alcohol building blocks. However, a key challenge remains: the *β*-C–H bonds of acyloxy groups are typically unactivated, and the electron-withdrawing inductive effect of the acyloxy group further disfavors hydrogen atom abstraction (HAA) at this position by common heteroatom-centered radicals due to polarity mismatch [16,17]. These factors collectively render highly site-selective intermolecular HAA at the desired *β*-C–H sites rather challenging (Fig. 1a) [18,19].

Recently, our group established a reversible C–H sampling strategy capable of achieving site-selective C(*sp^3^*)–H functionalization across substrates containing diverse unactivated C–H bonds [20], without requiring a site-selective C–H cleavage step. Instead of relying on innate bond bias or directing group strategies [21–23], an indiscriminate photoinduced HAA cycle is employed to transiently cleave various C–H bonds within the substrate, generating a panel of radical intermediates (the “*sampling*” phase). This is followed by a site-sensitive radical transformation step that selectively intercepts a specific radical intermediate (the “*selection*” phase) (Fig. 1b). A rate-tunable hydrogen atom donor (HAD) catalytic cycle is further integrated to quench off-target radicals and recycle them to the starting material, thus providing a crucial restorative pathway to suppress undesired pathways and ensure high mass balance [24–29]. Given that the rates of intramolecular radical addition/*β*-scission or direct radical-FG rearrangements are typically sensitive to the location of the radical center relative to the targeted FG [15,30–34], this reversible C–H sampling strategy is particularly suitable for developing direct FG translocation toward unactivated C–H sites. Since our seminal report in 2023 on the direct 1,4-translocation of cyano groups in alkyl nitriles [20], this strategy has been successfully extended by our group and others [35–38] to 1,2-boryl/H translocations of secondary and tertiary boronates [36,37], as well as 1,4-acyl/H translocations in seven-membered ring substrates (Fig. 1b) [38]. We are therefore interested in further expanding this strategy to address the aforementioned challenge in developing a radical-precursor-free 1,2-translocation reaction of masked alcohols, motivated by both the importance of this transformation and the capability of this strategy in promoting radical reactions at C–H sites that are kinetically less favored for HAA. Herein, we report photocatalytic 1,2-translocation of oxamoyl-protected alcohols to unactivated *β*-C–H sites under redox-neutral conditions with predictable site- and stereoselectivity. The oxamoyl protecting group (PG), which can be conveniently installed and removed in high yields, is proposed to not only facilitate the radical-acyloxy rearrangement through its potent electron-withdrawing nature relative to regular acyl groups, but also enhance HAA probability at spatially adjacent C–H positions *via* N–H hydrogen bonding with the catalyst (Fig. 1c) [39].

## RESULTS AND DISCUSSION

**Figure 2.**
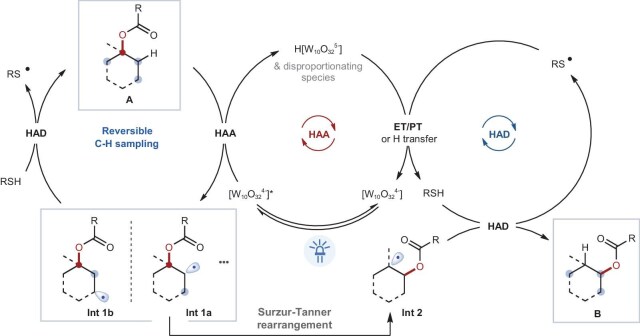
The mechanistic design of the transformation.

A detailed working mechanism is depicted in Fig. 2. First, a decatungstate HAA catalyst is employed [40–44]; upon excitation, it promotes the cleavage of various C(*sp^3^*)–H bonds in the ester substrate **A**, generating a range of carbon radical intermediates (e.g., **Int 1a, Int 1b**, *etc.*). Meanwhile, a thiol or thiophenol catalyst is also introduced, which can efficiently recycle these intermediates through HAD, thereby regenerating **A**. Among these intermediates, radical **Int 1a**, generated *β* to the C–O bond, may further undergo the desired 1,2-acyloxy migration, thus facilitating a productive pathway to the FG-relocated product **B** upon subsequent C–H formation. While such radical 1,2-acyloxy migration seems to be universal among a range of ester structures [10–15,45], the choice of the ester PG is still expected to be crucial for the present transformation. On one hand, a more electron-withdrawing PG would accelerate the key acyloxy rearrangement (from **Int 1a** to **Int 2**) by stabilizing the charge-separated transition state, thus rendering this productive step kinetically more competitive against the background HAD process [45,46]. However, during the C–H sampling phase (from **A** to **Int 1a**), a more electron-withdrawing PG would generally diminish the relative HAA probability at its adjacent C–H positions due to polarity-matching requirements, thus exerting an adverse effect on the overall reaction efficiency [17,47]. Such a trade-off highlights a primary challenge in the titled transformation and underscores the need for a judiciously designed PG motif to balance—or ideally, to simultaneously reinforce—both favorable acyloxy migration kinetics and sufficient *β*-HAA probability.

**Figure 3.**
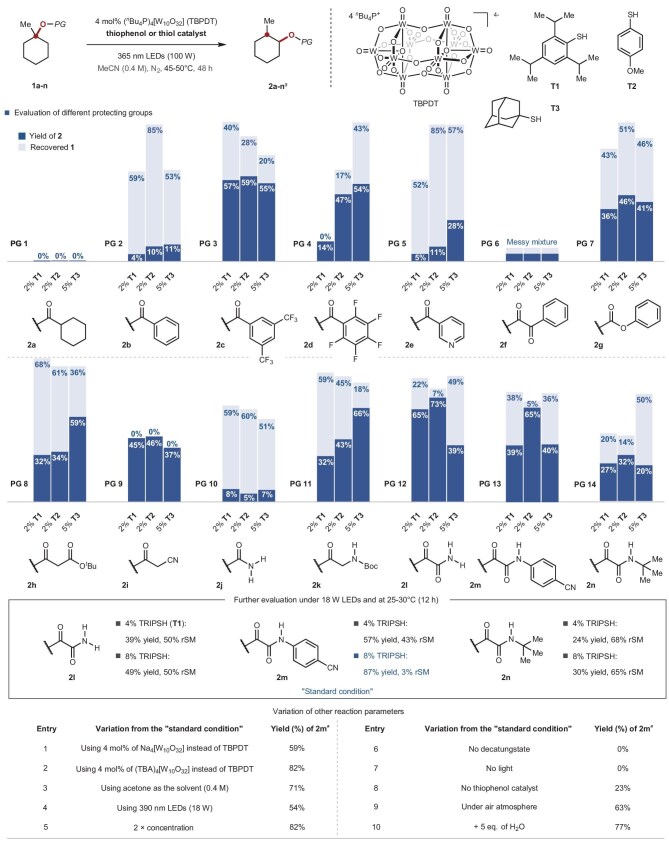
Reaction optimization. Evaluation of different protection groups, further optimization, and controlled studies. *^a^*The reactions were conducted on a 0.1 mmol scale and the yields were determined by ^1^H NMR using 1,3,5-trimethoxybenzene as the internal standard; rSM, recovered starting material. For details, see Supplementary Information.

Following this, 1-methylcyclohexan-1-ol was selected as the model alcohol to test our hypothesis, first by evaluating a diverse array of ester PGs (**1a–n**) under conditions designed to facilitate reversible C–H sampling. Specifically, the reactions were conducted with 4 mol% tetrabutylphosphonium decatungstate (TBPDT) as the photo-HAA catalyst and various thiols as the HAD catalysts (**T1–T3**), under 365 nm irradiation in MeCN at 45–50°C (Fig. 3). While cyclohexyl ester **1a** failed to yield any desired product, we were pleased to observe the formation of the translocation product **2b** when benzoate **1b** was employed, albeit in low yields. When increasing the electron deficiency of the aromatic esters (**1c** and **1d**), the yields could be improved to around 50%; however, the yield could not be further increased despite extensive screening, which is likely attributed, at least in part, to the strong electron-withdrawing inductive effect of these PGs that adversely affect *β*-HAA. Further, 3-picolinate **1e** provided consistently low yields, while 2-oxo-2-phenylacetate **1f** resulted in a complex reaction mixture, the latter likely due to its own photoexcitation under irradiation [48]. Other motifs, such as the less electron-deficient carbonates (**1g**) and carbamates (**1j**), as well as the more electron-deficient *α*-carbonylated (**1h**) or *α*-cyano (**1i**) esters, were also evaluated; however, these also failed to provide yields exceeding 60%, further underscoring the challenging trade-off between accelerating acyloxy migration and maintaining sufficient *β*-HAA reactivity. To our delight, *N*-Boc-glycinate (**1k**) afforded a 66% yield using 5 mol% 1-adamantanethiol (**T3**) as the HAD catalyst, and an oxamoyl PG (**1l**) further increased the yield to 73%. An *N*-arylated oxamoylate (**1m**) was also rather effective and gave a 65% yield, while an *N‑tert*‑butylated oxamoylate (**1n**) led to much poorer results. Given the superior performance of the oxamoyl group, we hypothesize that it may have two distinct beneficial effects: first, its strong electron-withdrawing nature (pKa ≈ 2.3 for oxamic acid [49] and ≈2.0 for oxanilic acid [50]) accelerates the 1,2-acyloxy migration step; and second, the amide N–H bond may enhance HAA probability at spatially adjacent C–H sites *via* hydrogen-bonding interactions [28] with the negatively charged decatungstate catalyst [51,52]. Notably, while carbamate **1j** and amino acid **1k** also possess amide N–H bonds, the former is insufficiently electron-deficient to facilitate acyloxy migration, whereas the latter (**1k**) indeed showed enhanced efficiency. Given that notable decomposition of the tertiary oxamoylate was consistently observed under elevated temperatures with relatively intense irradiation, further optimization was carried out at room temperature with a milder light source (18 W). Ultimately, the *N*‑aryl‑oxamoyl protected alcohol **1m** smoothly generated the desired product **2m** in 87% yield with nearly full conversion, using 4 mol% TBPDT and 8 mol% 2,4,6-triisopropylbenzenethiol (**T1**) under 365 nm irradiation for 12 h. A series of control experiments based on the optimal conditions was further conducted. Tetrabutylammonium decatungstate (TBADT) was also found to be a competent HAA catalyst, while the less soluble sodium decatungstate (NaDT) led to a lower yield (entries 1 and 2). The use of acetone as the solvent resulted in a slightly reduced yield of **2m** (entry 3), while the yield also decreased to 54% under 390 nm irradiation (entry 4). The reaction was virtually independent of concentration over a certain range, as a two-fold increase in reaction concentration resulted in nearly identical outcomes (entry 5). Further control experiments confirmed the essential roles of the HAA catalyst and photo-irradiation (entries 6 and 7). In the absence of thiophenol, product **2m** could still be obtained in 23% yield (entry 8), which is presumably attributed to the baseline but insufficient HAD capability of the reduced decatungstate species itself [36]. Furthermore, the reaction demonstrated remarkable tolerance to both air and moisture (entries 9 and 10).

**Figure 4.**
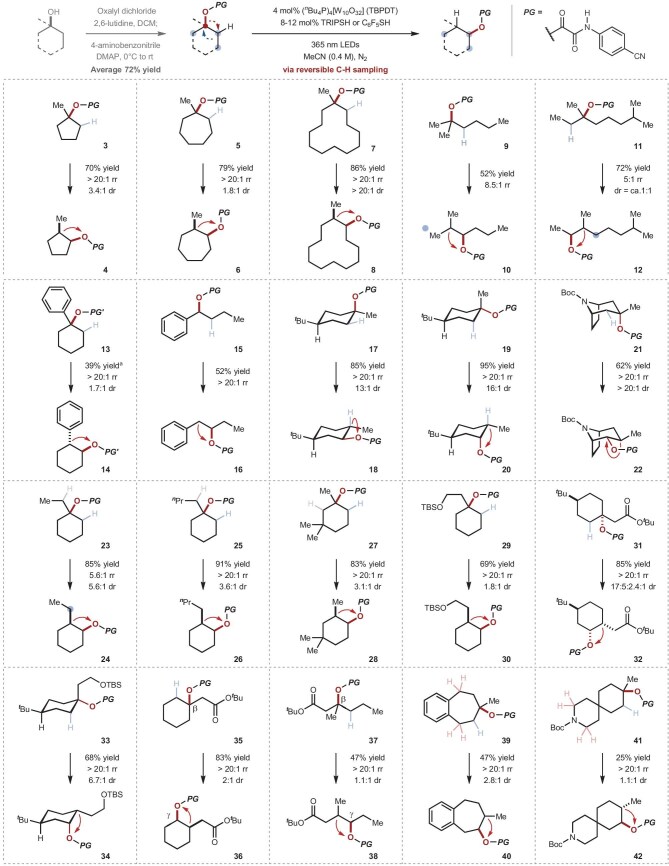
Substrate scope. Reactions were performed on a 0.1 mmol scale using two parallel runs, which were combined for isolation. Minor regioisomers are denoted by gray circles. *^a^*A simpler oxamoyl protecting group, analogous to that in **2l**, was employed. See the Supplementary Information for full details.

With the optimal conditions in hand, the substrate scope was next examined (Fig. 4). First, the *N*-aryl-oxamoyl PG was readily introduced by treating the alcohols with oxalyl chloride followed by 4-aminobenzonitrile in a one-pot procedure, affording an average yield of 72% across all substrates. Afterwards, we were delighted to observe that a diverse range of oxamoyl-protected alcohols, including cyclic and acyclic tertiary alkyl alcohols as well as secondary benzylic alcohols, could readily undergo this transformation under standard or slightly modified conditions. The 1,2-translocation products were formed exclusively, and no remote translocation was observed. The general reaction modes include relocation of the C–O bond from a tertiary to a secondary position, or from a benzylic to a homo-benzylic position, which aligns with the preference for forming more stable carbon-centered radicals in previously reported Surzur–Tanner rearrangements [10–15,45]. Conversely, migration of an acyloxy group between common secondary positions was not observed under our conditions, likely due to the lack of driving force and consequently a higher activation barrier. When substrates contain two or more *β* C–H positions, the site-selectivity follows distinct trends: (1) in linear substrates, the acyloxy group preferentially migrates toward a less hindered ethyl group over longer alkyl chains (**12**), likely due to a sterically favored HAA process; (2) in cyclic substrates, endocyclic migration appears to be more favored than exocyclic pathways (**24**), and between two different endocyclic *β*-methylenes, the less hindered one is preferred (**28**); (3) in all cases examined, *β*-methyl groups are the least favored, yielding only minor regioisomers if any (**6** and **10**). Furthermore, as the 1,2-acyloxy radical migration proceeds through a concerted three- or five-membered cyclic transition state [46], the reaction exhibits high stereospecificity at the chiral center α to the acyloxy group in cyclic substrates; the acyloxy group resides on the same face of the ring plane in both substrate and product, as clearly evidenced by substrates **17** and **19** where bulky *tert*-butyl groups fix the ring conformations. In these conformationally locked substrates, the diastereoselectivity is also generally high, positioning the “left-over” alkyl groups predominantly at the thermodynamically favored equatorial positions. A wide variety of tertiary alcohols with different ring sizes (**3, 5**, and **7**) are compatible, and substrates with C–O bonds oriented either axially (**17**) or equatorially (**19**) exhibit similar reactivity. Bridged or spirocyclic skeletons are also viable; for example, the nortropinone-derived substrate **21** afforded the 1,2-acyloxy relocated product **22** as a single diastereomer and regioisomer in 62% yield. The reaction is compatible with various functional groups, including Boc-protected amines (**22** and **42**), esters (**32**), and *tert*-butyldimethylsilyl (TBS)-protected alcohols (**30**). This reaction can also relocate the acyloxy group from the *β* to the *γ* position in carbonyl compounds (**32** and **38**)—a transformation that is nontrivial to achieve *via* conventional dehydration–hydroboration–oxidation sequence due to the propensity for forming conjugated double bonds during the initial step. Finally, it is noteworthy that substrates with highly reactive C(*sp^3^*)–H bonds at non-*β* positions (**39** and **41**) still underwent the desired 1,2-acyloxy translocation at unactivated *β* C(*sp^3^*)–H sites, further highlighting the capability of this reversible C–H sampling system to promote selective transformations at positions less favored by HAA.

**Figure 5.**
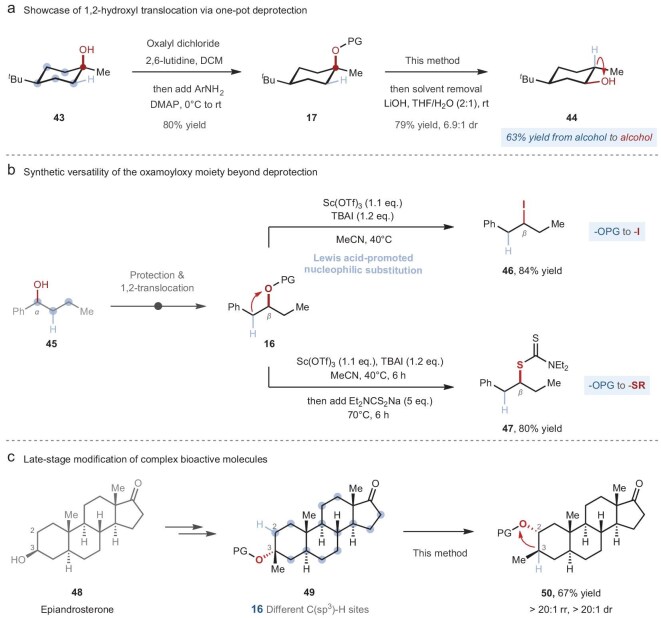
Further studies. (a) Showcase of 1,2-hydroxyl translocation *via* one-pot deprotection. (b) Synthetic versatility of the oxamoyloxy moiety beyond deprotection. (c) Late-stage modification of complex bioactive molecules. For details, see Supplementary Information.

To demonstrate the synthetic utility of this methodology, we next explored several downstream transformations and applications (Fig. 5). First, we demonstrated that deprotection of the oxamoyl group could be carried out in one pot after acyloxy translocation reaction using LiOH in THF/H_2_O at room temperature; for example, tertiary alcohol **43** was converted to secondary alcohol **44** in 63% overall yield with high regio- and diastereoselectivity *via* a sequential protection/translocation/deprotection protocol (Fig. 5a). Beyond simple hydrolysis, we further demonstrated that the *N*-aryl-oxamoyl group could serve as a versatile handle for Lewis acid-promoted nucleophilic substitution, thus enabling direct access to FGs beyond hydroxyl. Specifically, using Sc(OTf)_3_ and tetrabutylammonium iodide (TBAI) as the reagent, the translocated oxamoylate **16** was directly converted into iodide **46**, thus providing an entry point for further *S_N_*2-type transformations or cross-couplings. The alkyl iodide intermediate also enabled the one-pot, sequential *S_N_*2 synthesis of C–S bond formation product **47** (Fig. 5b). Finally, the applicability of our method was evaluated in the late-stage modification of an epiandrosterone derivative: despite the presence of 16 distinct C(*sp^3^*)–H sites, the reaction precisely translocated the acyloxy group from the C3 to the C2 position in 67% yield with exclusive stereospecificity (Fig. 5c). Collectively, these transformations and applications establish this method as a versatile platform for both hydroxyl repositioning and subsequent functionalization.

## CONCLUSION

In summary, we have developed a photocatalytic protocol for the direct 1,2-translocation of oxamoyl-protected alcohols to unactivated *β*-C–H sites via a reversible C–H sampling strategy. This redox-neutral method bypasses the need for pre-functionalization, achieving predictable regio- and stereoselectivity across a broad range of substrates, including complex bioactive molecules. The oxamoyl PG is proposed to play a dual role: its electron-withdrawing nature facilitates the radical 1,2-acyloxy shift, while the amide N–H likely enhances *β*-HAA probability through hydrogen-bonding interactions with the decatungstate catalyst. Beyond enabling the repositioning of hydroxyl groups, the oxamoyloxy handle could further undergo direct nucleophilic substitution after translocation, offering rapid access to diverse structural motifs. This work highlights once again the utility of the reversible C–H sampling strategy, and the development of other useful transformations based on this strategy is currently underway in our laboratory.

## METHODS

Representative procedure for 1,2-acyloxy/H translocation: An oven-dried 4-mL vial was charged with the oxamoyl-protected alcohol substrate (0.1 mmol, 1.0 equiv) and TBPDT (13.6 mg, 0.004 mmol, 0.04 equiv). The vial was transferred to a nitrogen-filled glovebox, where MeCN (0.23 mL) and a stock solution of 2,4,6-triisopropylbenzenethiol (**T1**) (0.4 M in MeCN, 20 µL, 0.08 equiv) were added. After sealing with a polytetrafluoroethylene-lined screw cap, the vial was removed from the glovebox and placed in a photoreactor (365 nm LED, 18 W). The reaction mixture was stirred at room temperature for 48 h. Two parallel reactions were conducted and combined upon completion. The mixture was concentrated under reduced pressure, and the residue was purified by flash column chromatography on silica gel to afford the desired product.

## Supplementary Material

nwag260_Supplemental_File
