# Supplementary material for: 1,2-Migration of oxamoyl-protected alcohols via reversible C–H sampling
Source: Natl Sci Rev. 2026 May 7;13(15):nwag260. doi: 10.1093/nsr/nwag260 (PMC13455613; doi:10.1093/nsr/nwag260)

Supplemental Information

1,2-Migration of Oxamoyl-Protected Alcohols *via* Reversible C–H Sampling

Yinyan Su[1], Duoqing Wang[1], Ken Chen[1], Yan Xu[1,*]

[1] *Beijing National Laboratory of Molecular Sciences, Key Laboratory of Bioorganic Chemistry and Molecular Engineering of Ministry of Education, College of Chemistry and Molecular Engineering, Peking University, Beijing 100871, China*

**Corresponding author, e-mail: yanx@pku.edu.cn*

1. General consideration 2

2. Substrate synthesis 3

3. Condition optimization 8

3.1 Evaluation of different protecting groups by using **1a-1n** 8

3.2 Further condition optimization at room temperature with a milder light source (18 W) 9

3.3 Variation of other reaction parameters 9

4. Installation of the oxamyl protecting group 16

5. Substrate scope 27

6. Further studies 43

7. References 45

8. Spectra 46

1. General consideration

All reactions were set up using standard Schlenk techniques or in a N_2 glovebox, unless otherwise specified. The 4 mL vials used for the key reaction and the 20 mL vials used for the material preparation reaction were purchased from commercial sources and dried in a 120 °C oven. Their specifications are as follows: 4 mL (1 dram, 15×45 mm) and 20 mL (5 dram, 27×57 mm), all supplied with PTFE-lined caps attached. Anhydrous MeCN, DMF, THF and DCM were purchased from *J&K Scientific* and used as received (with *J&K Seal*). Other reagents and solvents were directly used from the supplier without further purification. The retention factor (R_f) of analytical thin-layer chromatography (TLC) was recorded by using the regular commercial GF254 silica gel plate (0.23 mm) and visualized by UV light or TLC stains (potassium permanganate, iodine, PMA or CAM). Regular commercial silica gel (200-300 or 300-400 mesh) was used for flash column chromatography unless noted. The boiling point of the petroleum ether used for flash column chromatography in material preparation ranges from 60 to 90 °C, while that used for the same purpose ranges from 30 to 60 °C High-resolution mass spectra (HRMS) were obtained on an AB SCIEX X500R MS spectrometer (LC-Q-TOF, ESI) and are reported as *m/z*. ^{1}H NMR, ^{13}C NMR and ^{19}F NMR nuclear magnetic resonance spectra were recorded with a Bruker Avance III 400 MHz instrument (^{1}H at 400 MHz, ^{13}C at 101 MHz), a Bruker 600 MHz AVANCE NEO instrument (^{1}H at 600 MHz, ^{13}C at 151 MHz, ^{19}F at 565 MHz), or a Bruker Avance III 500 MHz instrument (^{1}H at 500 MHz, ^{13}C at 126 MHz, ^{19}F at 471 MHz) unless noted. For $CDCl_3$ solutions, the chemical shifts are reported as parts per million (ppm) referenced to residual protium or carbon of the solvents; $CHCl_3$ δ H (7.26 ppm) and $CDCl_3$ δ C (77.16 ppm). For C_6D_6 solutions the chemical shifts are reported as parts per million (ppm) referenced to residual protium or carbon of the solvents; C_6D_5H δ H (7.16 ppm) and C_6D_6 δ C (128.06 ppm). Coupling constants are reported in Hertz (Hz). Data for ^{1}H NMR spectra are reported as follows: chemical shift (ppm, referenced to protium: s = singlet, d = doublet, t = triplet, q = quartet, p = pentet (quintet), h = heptet, dd = doublet of doublets, td = triplet of doublets, ddd = doublet of doublet of doublets, dddd = doublet of doublet of doublet of doublets, m = multiplet, the coupling constant (Hz), and integration).

2. Substrate synthesis

In *Figure S1*, compounds **S4**[1], **43**[2], **S8**[2], **S20**[3], **T1**[4], NaDT[5], TBADT[6] and TBPDT[7] were synthesized according to the literature procedure. Compounds **S1**, **S2**, **S3**, **S5**, **S6**, **S7**, **45**, **S9**, **S10**, **S11**, **S12**, **T2**, **T3**, **T4** were commercially available and used as received. The others were prepared according to the following procedure.

Figure S1 | Substrates used in this study.

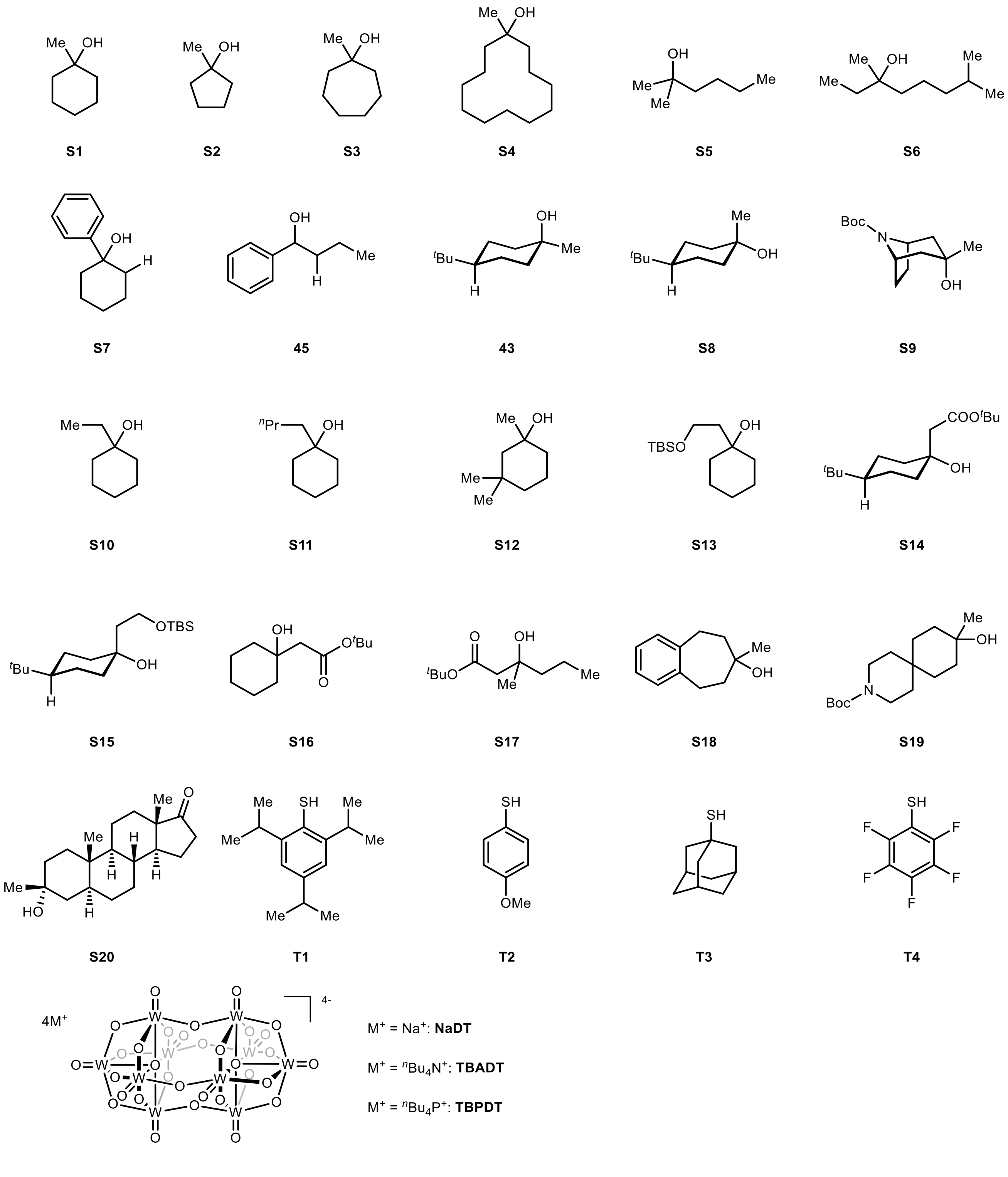

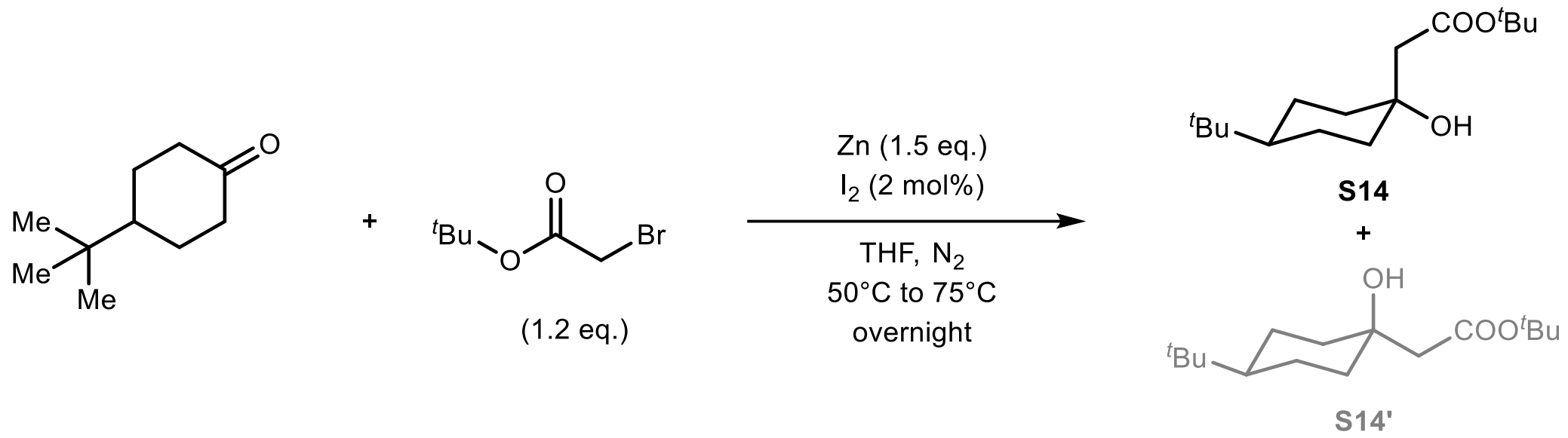

S14: To a 250 mL three-necked round-bottom flask equipped with a magnetic stir bar were added zinc powder (3.8 g, 58 mmol, 1.5 equiv), THF (120 mL) and 4-*tert*-butyl-cyclohexanone (5.9 g, 39 mmol, 1.0 equiv). The mixture was heated to 50 °C, and a solution of iodine (0.20 g, 0.78 mmol, 0.02 equiv) in THF (3 mL) was added. After stirring for 10 min, *tert*-butyl bromoacetate (9.1 g, 46 mmol, 1.2 equiv) was added dropwise, and the reaction mixture was heated to 75 °C and stirred overnight. Upon completion, the reaction was quenched with 2 M HCl (24 mL) and extracted with diethyl ether. The combined organic layers were washed with saturated aqueous $NaHCO_3$, dried over anhydrous Na_2SO_4 and concentrated *in vacuo*. The residue was purified by flash silica column chromatography (petroleum ether/ethyl acetate = 10:1). A portion of **S14** was collected as a pure compound (white solid, 2.2 g, 21% yield). R_f = 0.45 (petroleum ether/ethyl acetate = 6:1). ^{1}H NMR (500 MHz, $CDCl_3$) δ 3.92 (s, 1H), 2.46 (s, 2H), 1.86 – 1.76 (m, 2H), 1.74 – 1.65 (m, 2H), 1.47 (s, 11H), 1.09 – 0.96 (m, 3H), 0.85 (s, 9H). ^{13}C NMR (126 MHz, $CDCl_3$) δ 172.66, 81.66, 71.04, 47.60, 41.81, 38.52, 32.37, 28.29, 27.75, 24.43. HRMS: *Chemical formula* ([M]): $C_{16}H_{30}O_3$, calcd. 293.2087 $[M+Na]^+$, Found: 293.2090.

The remaining **S14** was collected as a mixture with **S14′** (white solid, 5.3 g, 50% yield in total).

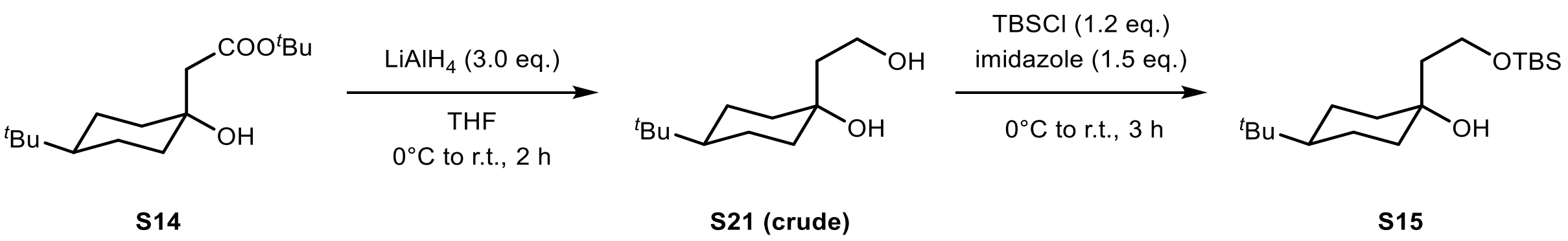

S21: To a 100 mL round-bottom flask containing **S14** (2.03 g, 7.5 mmol, 1.0 equiv) and 30 mL of anhydrous THF was added $LiAlH_4$ (0.85 g, 22.5 mmol, 3.0 equiv) in portions at 0 °C. The mixture was stirred for 1 h, and the reaction was monitored by TLC until completion. The reaction was quenched sequentially with distilled water (1 mL), aqueous NaOH (15%, 1 mL), and additional water (3 mL) at 0 °C. The resulting mixture was filtered, and the filtrate was extracted with diethyl ether. The combined organic layers were dried over anhydrous Na_2SO_4 concentrated *in vacuo*. The crude product was used directly in the next step without further purification.

S15: To a 100 mL round-bottom flask containing the crude product obtained from the previous step and imidazole (0.77 g, 11.3 mmol, 1.5 equiv) was added DMF (15 mL). In a separate vial, TBSCl (1.36 g, 9 mmol, 1.2 equiv) was dissolved in DMF (10 mL), and the resulting solution was added dropwise to the reaction mixture under ice-water bath cooling. The reaction was stirred for 3 h and monitored by TLC. Upon completion, the reaction was quenched by the addition of water (5 mL). The mixture was extracted with diethyl ether, and the combined organic layers were dried over anhydrous Na_2SO_4. The crude product was purified by column chromatography to afford **S15** as a colorless oil (1.25 g, 53% yield). R_f = 0.40 (petroleum ether/ethyl acetate = 10:1).^{1}H NMR (400 MHz, $CDCl_3$) δ 4.07 (s, 1H),

3.88 (t, J = 5.7 Hz, 2H), 1.88 – 1.81 (m, 2H), 1.77 (t, J = 5.7 Hz, 2H), 1.68 (dt, J = 12.8, 1.9 Hz, 2H), 1.43 (td, J = 13.0, 3.5 Hz, 2H), 1.11 – 0.91 (m, 4H), 0.90 (s, 9H), 0.84 (s, 9H), 0.09 (s, 6H). ^{13}C NMR (101 MHz, $CDCl_3$) δ 72.42, 60.55, 47.52, 38.25, 36.28, 32.25, 27.64, 25.84, 24.49, 18.04, -5.62. HRMS: *Chemical formula* ([M]): $C_{18}H_{38}O_2Si$, calcd. 315.2714 $[M+H]^+$, Found: 315.2708.

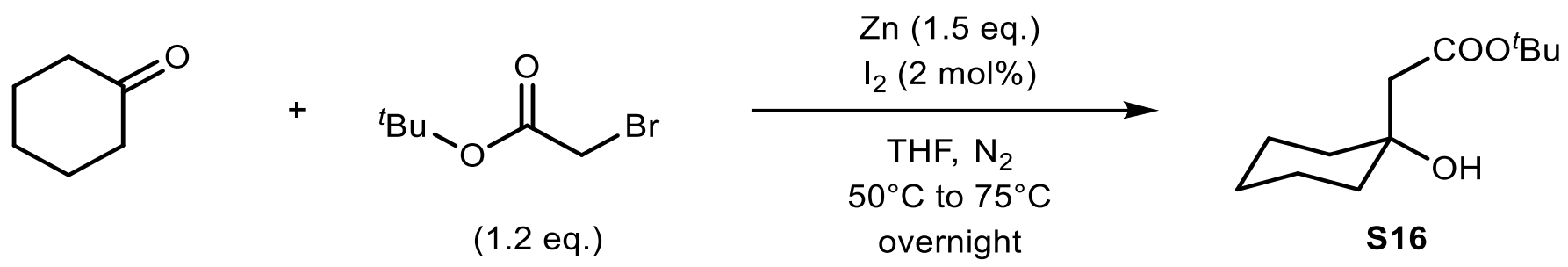

S16: To a 250 mL three-necked round-bottom flask equipped with a magnetic stir bar were added zinc powder (4.9 g, 75 mmol, 1.5 equiv), THF (160 mL) and cyclohexanone (4.9 g, 50 mmol, 1.0 equiv). The mixture was heated to 50 °C, and a solution of iodine (0.25 g, 1 mmol, 0.02 equiv) in THF (3 mL) was added. After stirring for 10 min, *tert*-butyl bromoacetate (11.7 g, 60 mmol, 1.2 equiv) was added dropwise, and the reaction mixture was heated to 75 °C and stirred overnight. Upon completion, the reaction was quenched with 2 M HCl (24 mL) and extracted with diethyl ether. The combined organic layers were washed with saturated aqueous $NaHCO_3$, dried over anhydrous Na_2SO_4 and concentrated *in vacuo*. The residue was purified by flash silica column chromatography (petroleum ether/ethyl acetate = 10:1) to afford **S16** as a colorless oil (7.3 g, 68% yield). R_f = 0.40 (petroleum ether/ethyl acetate = 6:1).^{1}H NMR (500 MHz, $CDCl_3$) δ 3.59 (s, 1H), 2.37 (s, 2H), 1.73 – 1.60 (m, 4H), 1.57 – 1.49 (m, 1H), 1.47 (s, 9H), 1.46 – 1.36 (m, 4H), 1.32 – 1.23 (m, 1H). The spectrum matches that reported in the literature.[8]

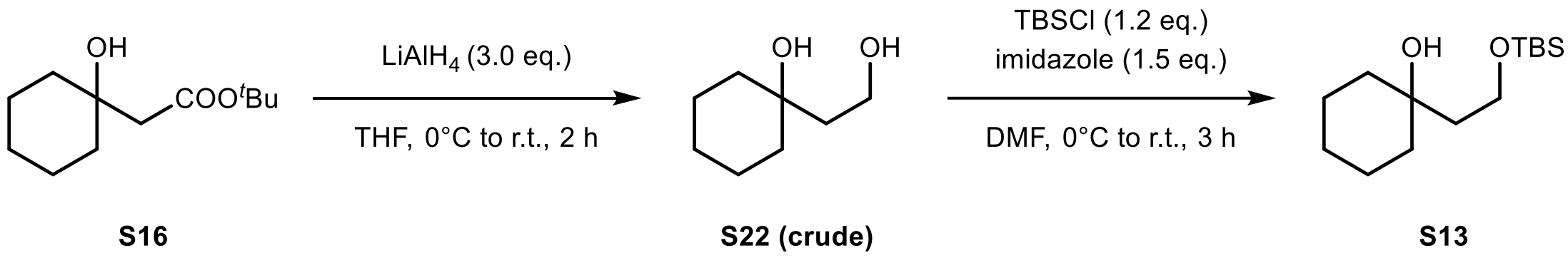

S22: To a 100 mL round-bottom flask containing **S16** (2.1 g, 10 mmol, 1.0 equiv) and 30 mL of THF was added $LiAlH_4$ (1.1g, 30 mmol, 3.0 equiv) in portions at 0 °C. The mixture was stirred at room temperature until completion, as monitored by TLC. The reaction was quenched sequentially with distilled water (1 mL), aqueous NaOH (15%, 1 mL), and additional water (3 mL) at 0 °C. The resulting mixture was filtered, and the filtrate was extracted with diethyl ether. The combined organic layers were dried over anhydrous Na_2SO_4 and concentrated *in vacuo*. The crude product **S22** was used directly in the next step without further purification.

S13: To a 100 mL round-bottom flask containing **S22** (from previous step) and imidazole (1.0 g, 15 mmol, 1.5 equiv) was added DMF (20 mL) at 0 °C. A solution of TBSCl (1.8 g, 12 mmol, 1.2 equiv) in DMF (10 mL) was added dropwise at 0 °C. The reaction was warmed to room temperature and stirred for 3 h. The reaction was quenched by the addition of water (5 mL). The mixture was extracted with diethyl ether. The combined organic layers were dried over anhydrous Na_2SO_4 and concentrated *in vacuo*. The crude product was purified by column chromatography to afford **S13** as a colorless oil (1.7 g, 64% yield). R_f = 0.38 (petroleum ether/ethyl acetate = 10:1). ^{1}H NMR (400 MHz,

$CDCl_3$) δ 3.89 (t, *J* = 5.7 Hz, 2H), 3.70 (s, 1H), 1.69 (t, *J* = 5.7 Hz, 2H), 1.67 – 1.55 (m, 4H), 1.54 – 1.46 (m, 1H), 1.45 – 1.37 (m, 4H), 1.35 – 1.25 (m, 1H), 0.89 (s, 9H), 0.08 (s, 6H). ^{13}C NMR (101 MHz, $CDCl_3$) δ 71.52, 60.22, 41.22, 37.73, 26.02, 25.86, 22.30, 18.07, -5.61. HRMS: *Chemical formula* ([M]): $C_{14}H_{30}O_2Si$, calcd. 259.2088 $[M+H]^+$, Found: 259.2088.

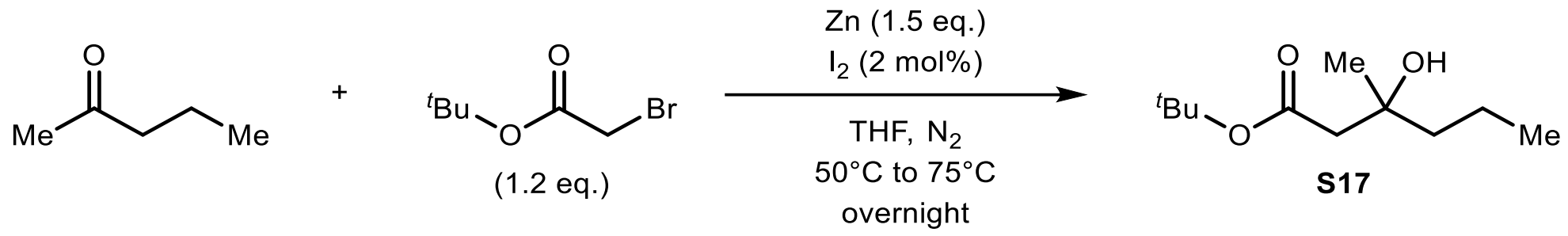

S17

S17: To a 250 mL three-necked round-bottom flask equipped with a magnetic stir bar were added zinc powder (4.9 g, 75 mmol, 1.5 equiv), THF (160 mL) and 2-pentanone (4.3 g, 50 mmol, 1.0 equiv). The mixture was heated to 50 °C, and a solution of iodine (0.25 g, 1 mmol, 0.02 equiv) in anhydrous THF (3 mL) was added. After stirring for 10 min, *tert*-butyl bromoacetate (11.7 g, 60 mmol, 1.2 equiv) was added dropwise. The reaction mixture was heated to 75 °C and stirred overnight. Upon completion, the reaction was quenched with 2 M HCl (24 mL) and extracted with diethyl ether. The combined organic layers were washed with saturated aqueous $NaHCO_3$ solution, dried over anhydrous Na_2SO_4 and concentrated *in vacuo*. The residue was purified by flash silica column chromatography (petroleum ether/ethyl acetate = 10:1) to afford **S17** as a colorless oil (4.5 g, 45% yield). R_f = 0.45 (petroleum ether/ethyl acetate = 6:1). ^1H NMR (400 MHz, $CDCl_3$) δ 3.71 (s, 1H), 2.46 – 2.29 (m, 2H), 1.48 (s, 11H), 1.42 – 1.31 (m, 2H), 1.21 (s, 3H), 0.92 (t, *J* = 7.1 Hz, 3H). ^{13}C NMR (101 MHz, $CDCl_3$) δ 172.68, 81.44, 71.08, 46.69, 44.47, 28.12, 26.65, 18.81, 15.92. HRMS: *Chemical formula* ([M]): $C_{11}H_{22}O_3$, calcd. 225.1461 $[M+Na]^+$, Found: 225.1462.

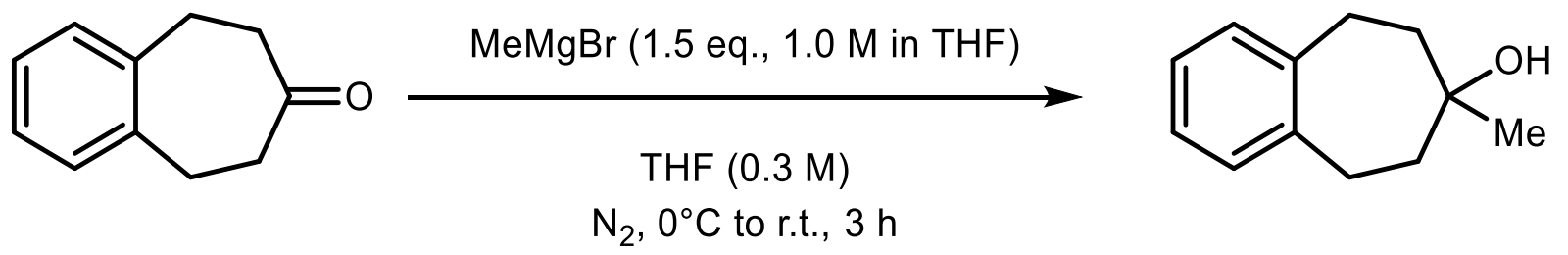

S18

S18: To a 50 mL flask containing MeMgBr (4.5 mL, 1.0 M in THF, 4.5 mmol, 1.5 equiv) at 0 °C was added a solution of 5,6,8,9-tetrahydro-7H-benzo[7]annulen-7-one (480 mg, 3.0 mmol, 1.0 equiv) in THF (5 mL). The resulting mixture was allowed to warm to room temperature and stirred for 3 h. The reaction was quenched by the addition of saturated aqueous NH_4Cl solution (3 mL) at 0 °C. The mixture was extracted with diethyl ether. The combined organic layers were dried over anhydrous Na_2SO_4 and concentrated *in vacuo.* The residue was purified by flash silica column chromatography to afford **S18** as a colorless liquid (453 mg, 86% yield). R_f = 0.35 (petroleum ether/ethyl acetate = 10:1). ^1H NMR (500 MHz, $CDCl_3$) δ 7.13 (s, 4H), 3.15 (t, *J* = 10.8 Hz, 2H), 2.59 – 2.48 (m, 2H), 1.83 (dd, *J* = 13.2, 6.6 Hz, 2H), 1.63 (t, *J* = 12.1 Hz, 2H), 1.42 (s, 1H), 1.28 (s, 3H). ^{13}C NMR (126 MHz, $CDCl_3$) δ 142.90, 128.86, 126.28, 72.38, 40.99, 32.00, 30.05. HRMS: *Chemical formula* ([M]): $C_{12}H_{16}O$, calcd. 199.1093 $[M+Na]^+$, Found: 199.1088.

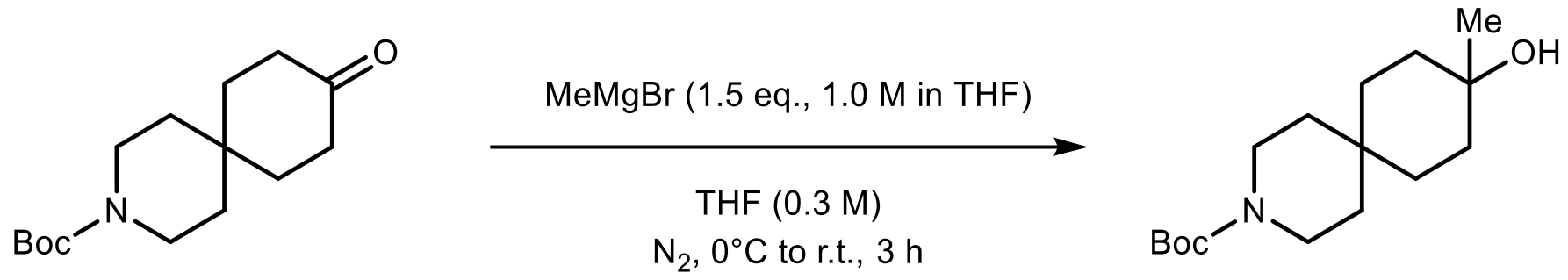

S19: To a 50 mL flask containing MeMgBr (5.6 mL, 1.0 M in THF, 5.6 mmol, 1.5 equiv) at 0 °C was added a solution of *tert*-butyl 9-oxo-3-azaspiro[5.5]undecane-3-carboxylate (1.0 g, 3.7 mmol, 1.0 equiv) in THF (5 mL). The resulting mixture was allowed to warm to room temperature and stirred for 3 h. The reaction was quenched by the addition of saturated aqueous NH_4Cl solution (5 mL) at 0 °C. The mixture was extracted with diethyl ether. The combined organic layers were dried over anhydrous Na_2SO_4 and concentrated *in vacuo.* The residue was purified by flash silica column chromatography to afford **S19** as a white solid (0.92 g, 58% yield). R_f = 0.37 (petroleum ether/ethyl acetate = 2:1). ^{1}H NMR (400 MHz, $CDCl_3$) δ 3.39 – 3.30 (m, 4H), 1.50 – 1.46 (m, 5H), 1.46 – 1.32 (m, 17H), 1.21 (s, 3H). ^{13}C NMR (101 MHz, $CDCl_3$) δ 155.14, 79.31, 69.89, 39.72, 37.82, 34.50, 33.14, 31.81, 30.61, 30.02, 28.59. HRMS: *Chemical formula* ([M]): $C_{16}H_{29}NO_3$, calcd. 284.2220 $[M+H]^+$, Found: 284.2220.

3. Condition optimization

3.1 Evaluation of different protecting groups by using 1a-1n

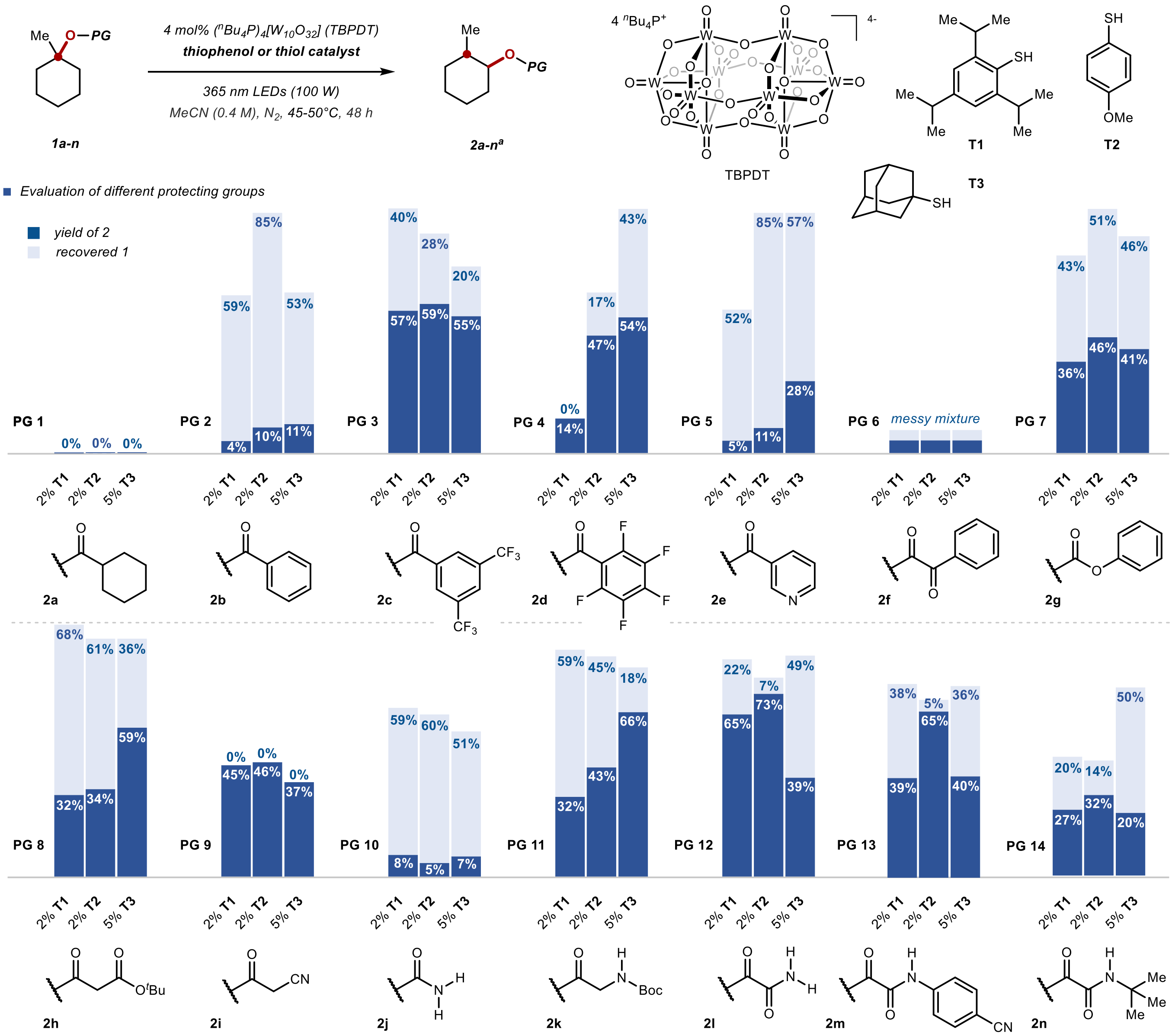

Figure S2 | Evaluation of different protecting groups *(adapted from Figure 3 of the manuscript)*. [a] The reactions were conducted on a 0.1 mmol scale and the yields were determined by ^{1}H NMR using 1,3,5-trimethoxybenzene as the internal standard.

Experimental procedure for evaluation of different protecting groups: A 4 mL oven-dried vial was charged with compounds **1a-n** (0.1 mmol, 1.0 equiv) and tetrabutylphosphonium decatungstate (13.6 mg, 0.004 mmol, 0.04 equiv). The vial was transferred to a nitrogen glovebox, where a stock solution of the corresponding thiophenol or thiol (0.4 M in MeCN) was added, followed by additional MeCN to bring the total solvent volume to 0.25 mL. The reaction mixture was sealed with a PTFE-lined screw cap, removed from the glovebox, and stirred in *photoreactor A* (see below for details) for 48 h. The reaction was then concentrated under reduced pressure. 1,3,5-Trimethoxybenzene (16.8 mg, 0.1 mmol) was added as the internal standard, and the residue was dissolved in $CDCl_3$ (0.6 mL). The resulting solution was transferred to an NMR tube, and the yields of **2a-n** in the crude reaction mixture were determined by ^{1}H NMR analysis.

3.2 Further condition optimization at room temperature with a milder light source (18 W)

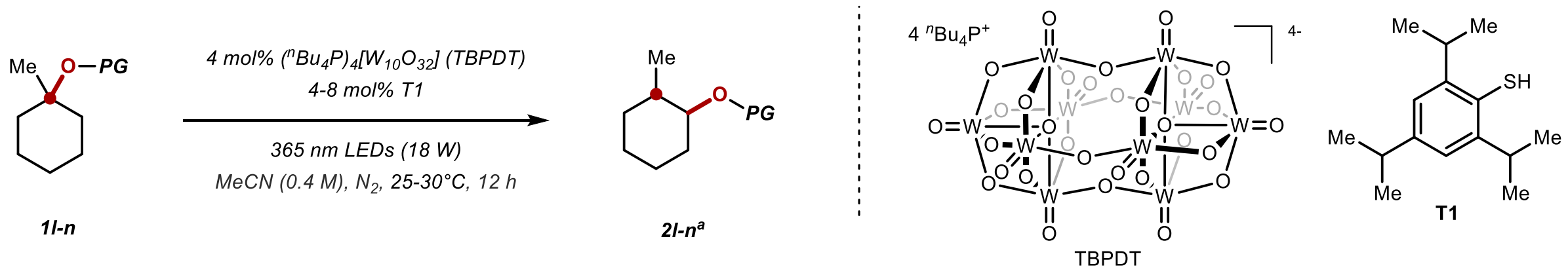

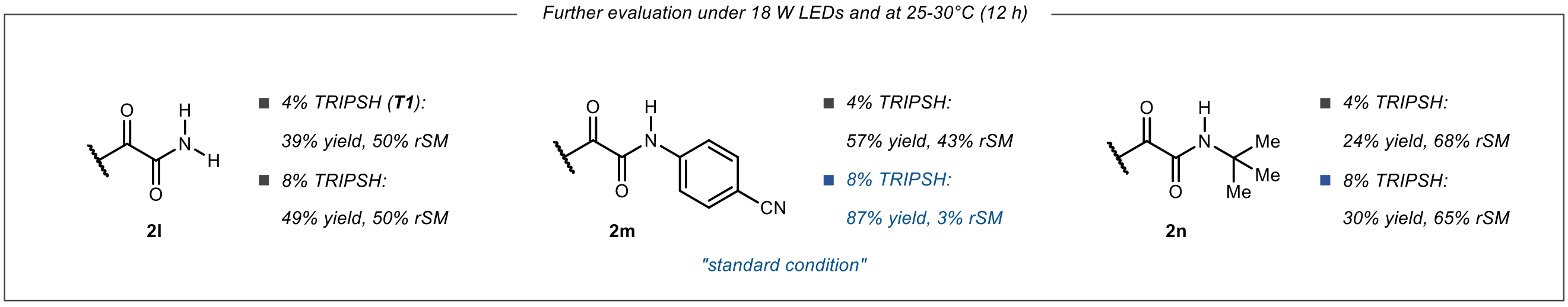

Figure S3 | Further optimization *(adapted from Figure 3 of the manuscript)*. [a] The reactions were conducted on a 0.1 mmol scale and the yields were determined by ^{1}H NMR using 1,3,5-trimethoxybenzene as the internal standard.

Experimental procedure for Figure S3: A 4 mL oven-dried vial was charged with compounds **1l**, **1m** or **1n** (0.1 mmol, 1.0 equiv) and tetrabutylphosphonium decatungstate (13.6 mg, 0.004 mmol, 0.04 equiv). The vial was transferred to a nitrogen glovebox, where a stock solution of 2,4,6-triisopropylbenzenethiol (**T1**) (0.4 M in MeCN) was added, followed by additional MeCN to bring the total solvent volume to 0.25 mL. The reaction mixture was sealed with a PTFE-lined screw cap, removed from the glovebox, and stirred in *photoreactor B* (see below for details) for 12 h. The reaction was then concentrated *in vacuo*. 1,3,5-Trimethoxybenzene (16.8 mg, 0.1 mmol) was added as the internal standard, and the residue was dissolved in $CDCl_3$ (0.6 mL). The resulting solution was transferred to an NMR tube, and the yields of **2l**, **2m** or **2n** in the reaction mixture were determined by ^{1}H NMR analysis.

3.3 Variation of other reaction parameters

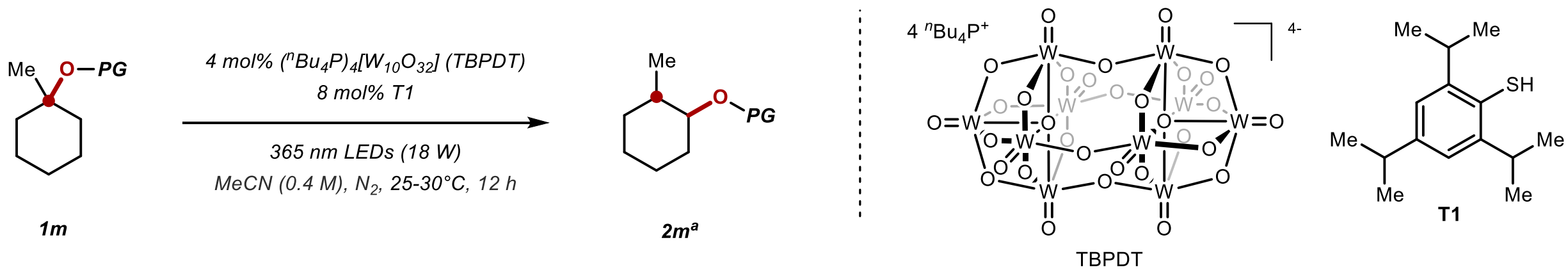

Variation of other reaction parameters

entry	variation from the "standard condition"	yield (%) of 2m [a]	entry	variation from the "standard condition"	yield (%) of 2m [a]
1	using 4 mol% of $Na_4[W_{10}O_{32}]$ instead of TBPDT	59%	6	no decatungstate	0%
2	using 4 mol% of $(TBA)_4[W_{10}O_{32}]$ instead of TBPDT	82%	7	no light	0%
3	using acetone as the solvent (0.4 M)	71%	8	no thiophonal catalyst	23%
4	using 390 nm LEDs (18 W)	54%	9	under air atmosphere	63%
5	2 × concentration	82%	10	+ 5 eq. of H_2O	77%

Figure S4 | Variation of other reaction parameters *(adapted from Figure 3 of the manuscript)*.[a] The reactions were conducted on a 0.1 mmol scale and the yields were determined by ^{1}H NMR using 1,3,5-trimethoxybenzene as the internal standard.

The “*standard condition*” for Figure S4: An oven-dried 4 mL vial was charged with **1m** (28.6 mg, 0.1 mmol, 1.0 equiv.) and tetrabutylphosphonium decatungstate (TBPDT, 13.6 mg, 4 mol%). The vial was transferred to a nitrogen-filled glovebox. MeCN (0.23 mL) and a stock solution of **T1** (0.4 M in MeCN, 20 μL, 0.008 mmol, 8 mol%) were added. The reaction was sealed with a PTFE-lined cap, transferred out of the glovebox, and stirred in *photoreactor B* for 12 h, then concentrated under reduced pressure. 1,3,5-Trimethoxybenzene (16.8 mg, 0.1 mmol) was added to the residue as the internal standard, and the mixture was dissolved in $CDCl_3$ (0.6 mL). The resulting solution was transferred to an NMR tube, and the yield of **2m** was determined to be 87% by ^{1}H NMR analysis of the crude reaction mixture.

For *entry 5*, the reaction was set up identically to the *standard condition*, except that the total volume of MeCN was reduced to 125 μL (doubling the concentration). For *entry 8*, the reaction was set up identically to the *standard condition*; however, after removal from the glovebox, the vial was opened and the mixture was stirred under an ambient atmosphere for 8 minutes before the cap was resealed and the vial was placed in the photoreactor. For all other entries, modifications were made according to the variations indicated in *Figure S4*.

Photoreactor A:

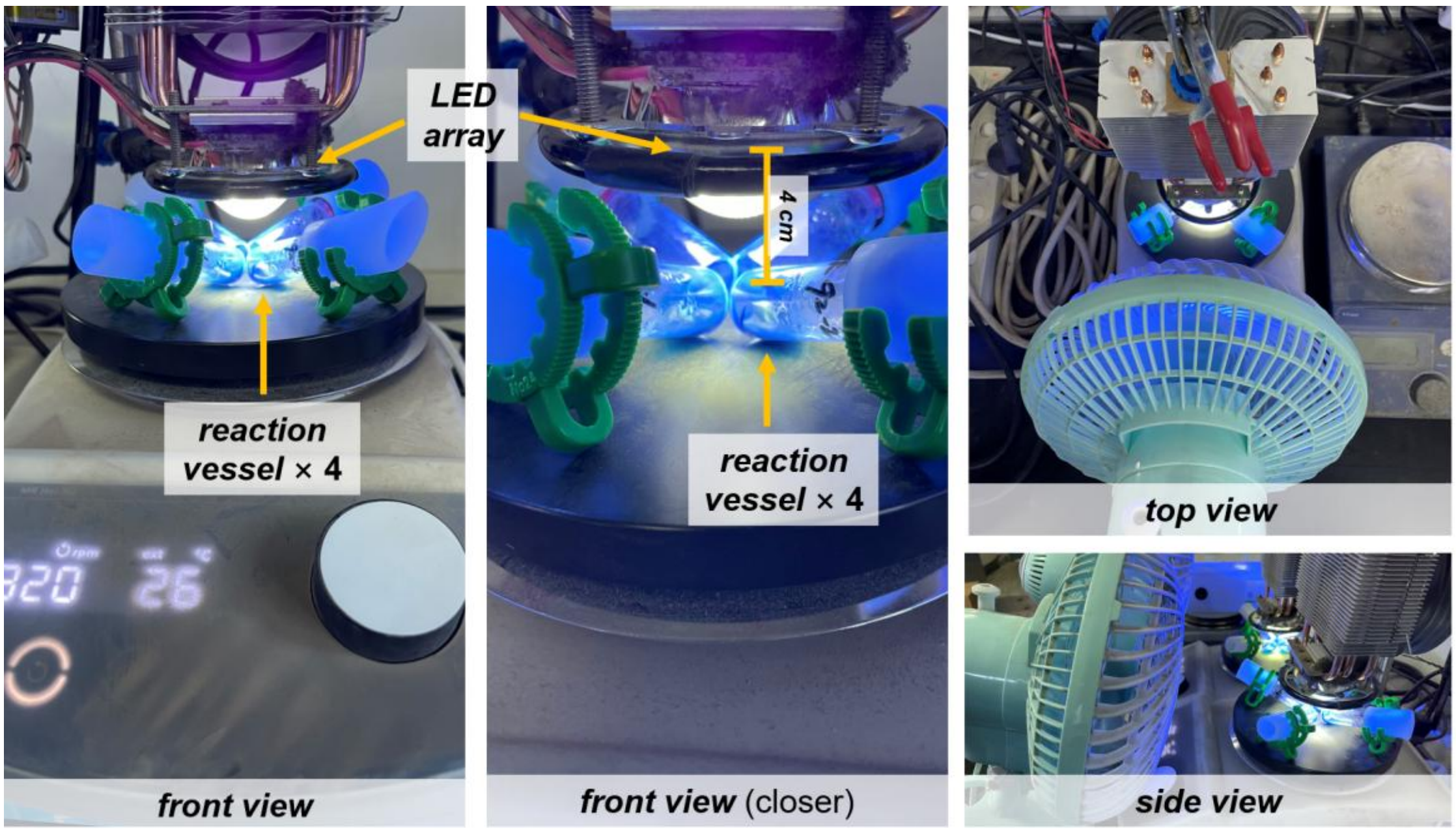

Figure S5 | Photoreactor A (*Figure adapted from the Supplementary Information of our previous work in Nature, 2023, 620, 1007-1012*)

The setup of *Photoreactor A* is identical to that reported in our previous work.[9] The reactor is equipped with a 100 W LED array (purchased from GuangHong Chips) with a peak emission wavelength of 366.5 nm. The emission spectrum is provided in *Figure S6*. The LED array was positioned 4 cm above the solution surface in the reaction vessels (4 mL, fabricated from standard borosilicate glass). At this distance, the average irradiance was measured to be approximately 800-1000 mW/cm^2 at the peak wavelength. A cooling fan was placed adjacent to the reaction vessels to maintain the internal temperature between 40-50 °C, as monitored by a handheld infrared (IR) thermometer.

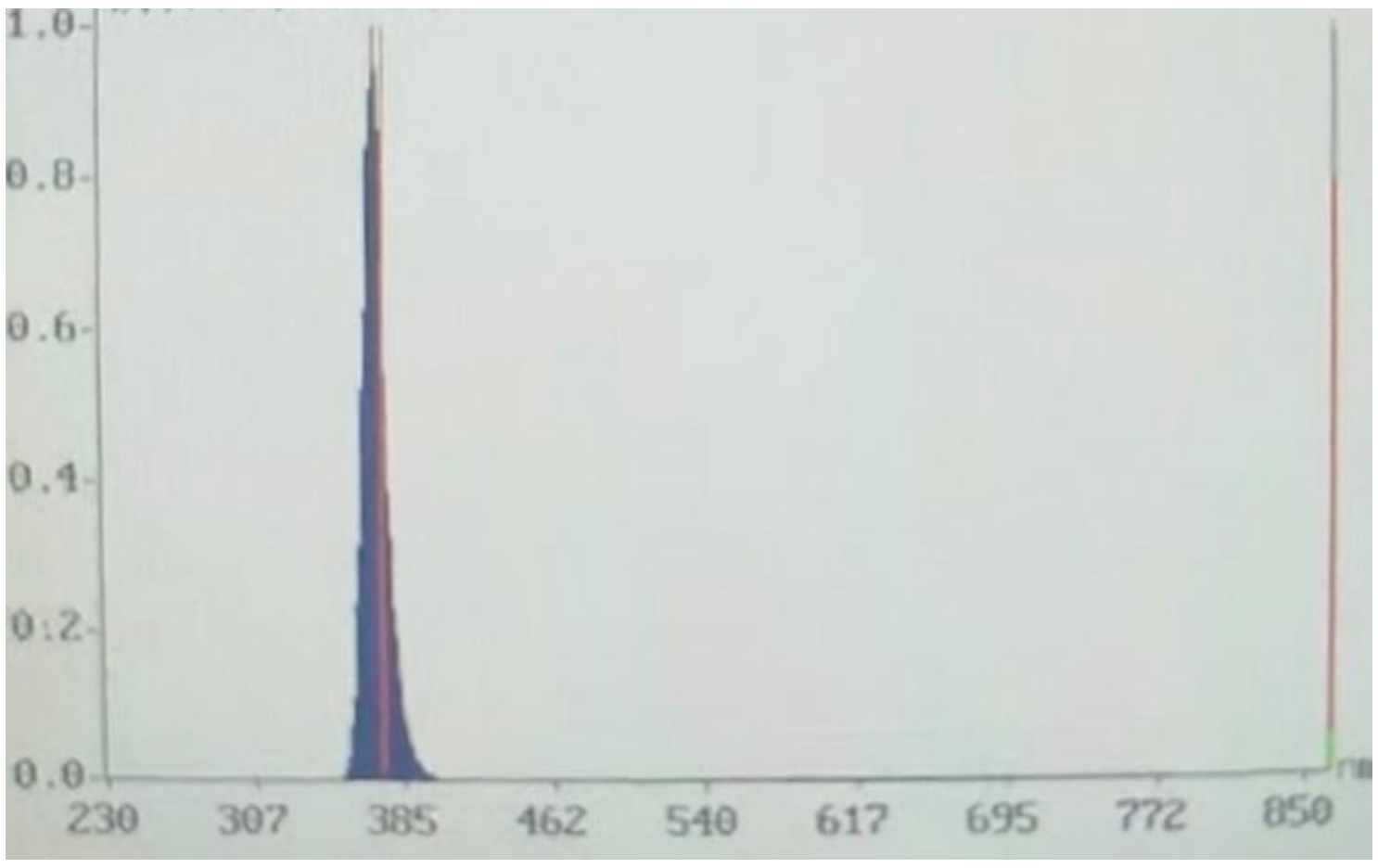

Figure S6 | The emission spectrum of the LED in *Photoreactor A* (*monitored by WATTCAS-PCS-230850*).

Photoreactor B:

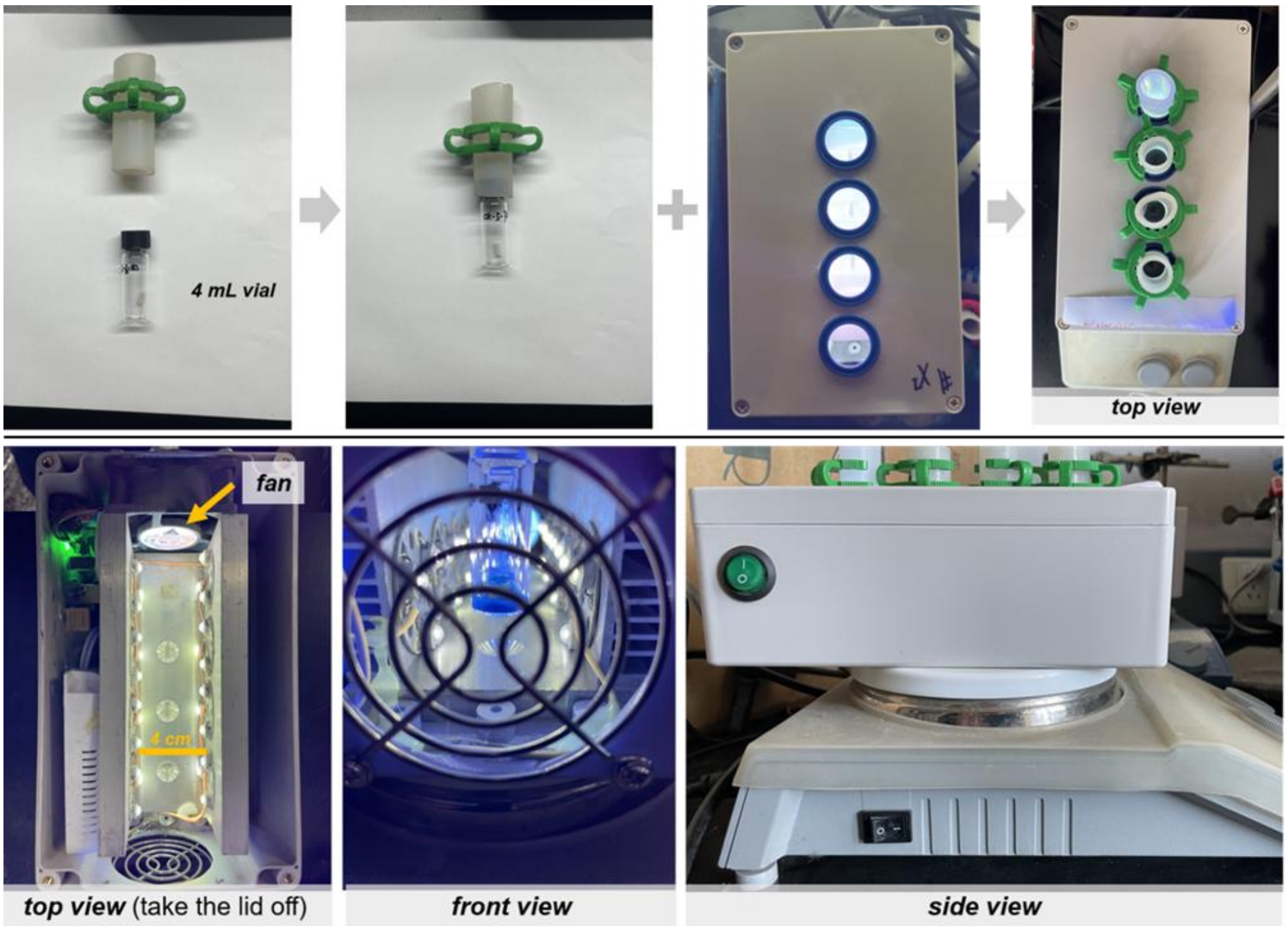

Figure S7 | Photoreactor B (*Figure adapted from the Supplementary Information of our previous work in Nature, 2023, 620, 1007-1012*)

The setup of *Photoreactor B* is identical to that reported in our previous work.[9] The photoreactor (Commercially available, $150/unit) consists of 24 × 3 W LEDs (peak wavelength: 369.7 nm) distribution across four illumination holes (18 W/well, 72 W in total) and an integrated cooling fan. The reaction setup was shown in *Figure S7*. The emission spectrum of the LED bead used in *Photoreactor B* is provided in *Figure S8*. In this setup, the irradiance at the bottom of the reaction vessels was measured to be approximately 32-35 mW/cm^2 at the peak wavelength, whereas the irradiance on the side walls and at the solution surface could not be conveniently measured. The temperature of the reaction was measured to be 25-30 °C by a handheld infrared (IR) thermometer.

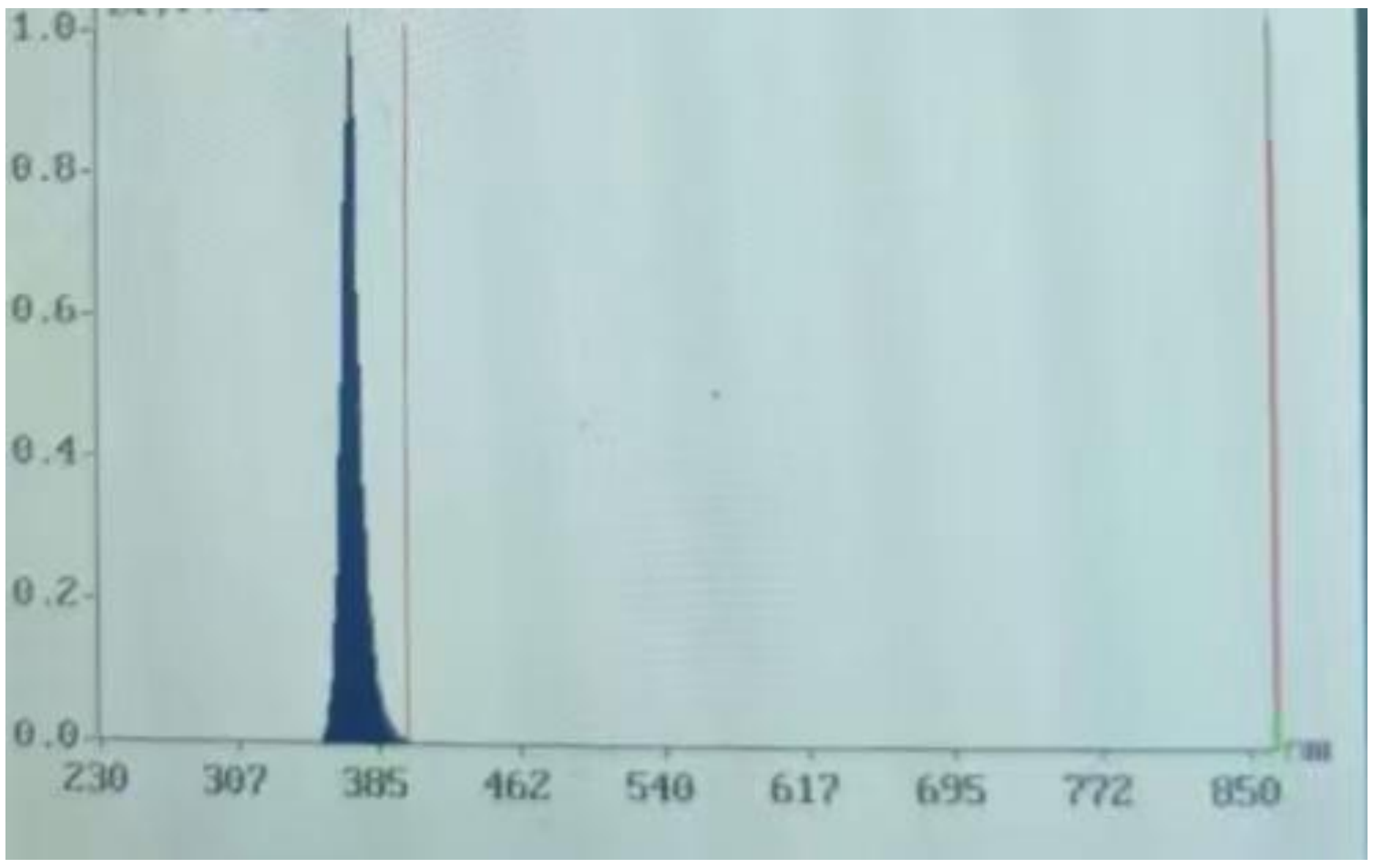

Figure S8 | The emission spectrum of the LED in *Photoreactor B* (*monitored by WATTCAS-PCS-230850*).

*Synthesis of substrates **1a-n***: compounds **1a**[10], **1b**[11], **1f**[12] and **1g**[13] were synthesized according to literature procedure; the remaining compounds were prepared as described below.

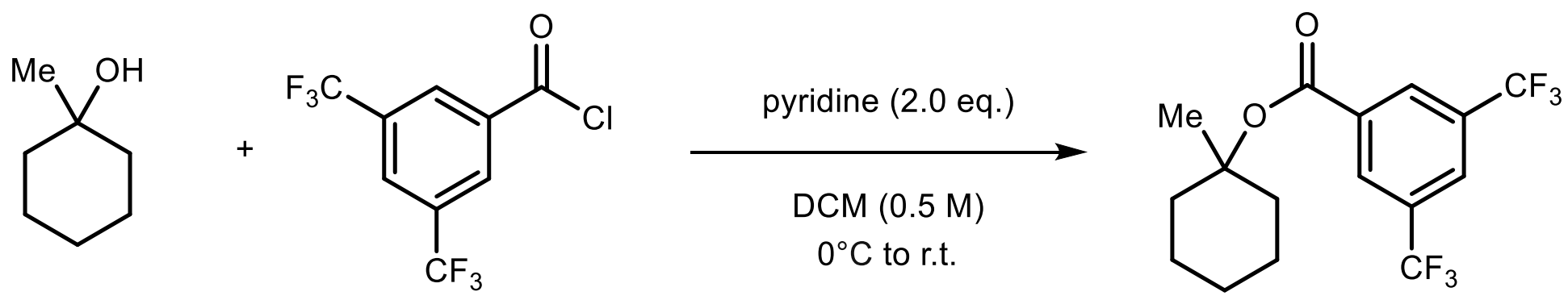

1c

1c: To a DCM (20 mL) solution of 1-methylcyclohexanol (1.14 g, 10 mmol, 1.0 equiv) and pyridine (1.58 g, 20 mmol, 2.0 equiv) was added 3,5-bis(trifluoromethyl)benzoyl chloride (3.32 g, 12 mmol, 1.2 equiv) at 0 °C. The reaction mixture was allowed to warm to room temperature and stirred overnight. The reaction was quenched by the addition of saturated aqueous NH_4Cl, and the mixture was extracted with DCM. The combined organic layers were washed successively with saturated aqueous Na_2CO_3 and brine. The resulting mixture was concentrated *in vacuo*, and the residue was purified by flash column chromatography on silica gel (petroleum ether/ethyl acetate) to give **1c** as a colorless oil (2.37 g, 67% yield). R_f = 0.6 (petroleum ether/ethyl acetate = 40:1). ^{1}H NMR (600 MHz, $CDCl_3$) δ 8.44 (s, 2H), 8.03 (s, 1H), 2.38 – 2.28 (m, 2H), 1.66 – 1.51 (m, 10H), 1.39 – 1.29 (m, 1H). ^{13}C NMR (151 MHz, $CDCl_3$) δ 162.82, 134.36, 132.17 (q, J = 33.9 Hz), 129.65 (q, J = 4.0 Hz), 126.00 (p, J = 3.7 Hz), 123.14 (q, J = 272.7 Hz), 84.91, 36.77, 25.61, 25.40, 22.39. ^{19}F NMR (565 MHz, $CDCl_3$) δ -63.01. HRMS: *Chemical formula* ([M]): $C_{16}H_{16}F_6O_2$, calcd. 377.0947 $[M+Na]^+$, Found: 377.0946.

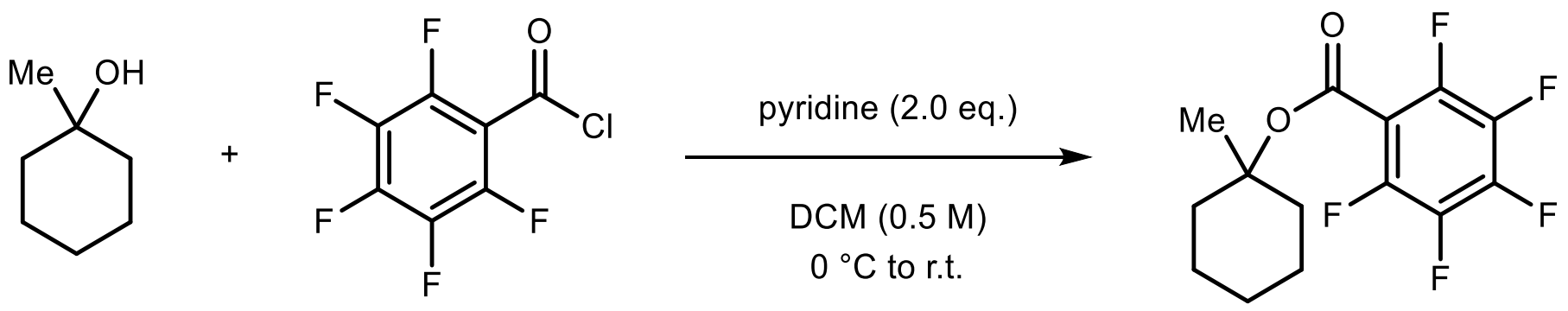

1d

1d: To a DCM (20 mL) solution of 1-methylcyclohexanol (1.14 g, 10 mmol, 1.0 equiv) and pyridine (1.58 g, 20

mmol, 2 equiv) was added pentafluorobenzoyl chloride (2.78 g, 12 mmol, 1.2 equiv) at 0 °C. The reaction mixture was allowed to warm to room temperature and stirred overnight. The reaction was quenched by the addition of saturated aqueous NH_4Cl, and the mixture was extracted with DCM. The combined organic layers were washed successively with saturated aqueous Na_2CO_3 and brine. The resulting mixture was concentrated *in vacuo*, and the residue was purified by flash column chromatography on silica gel (petroleum ether/ethyl acetate) to give **1d** as a colorless oil (2.55 g, 83% yield). R_f = 0.7 (petroleum ether/ethyl acetate = 40:1). ^{1}H NMR (400 MHz, $CDCl_3$) δ 2.36 – 2.09 (m, 2H), 1.68 – 1.38 (m, 10H), 1.34 – 1.22 (m, 1H). ^{13}C NMR (101 MHz, $CDCl_3$) δ 157.89, 145.05 (dddt, *J* = 255.6, 11.4, 7.5, 4.0 Hz), 144.37 – 141.24 (m), 139.31 – 136.06 (m), 110.29 (td, *J* = 17.1, 4.0 Hz), 87.09, 36.69, 25.74, 25.29, 21.92. ^{19}F NMR (471 MHz, $CDCl_3$) δ -139.62, -150.65, -160.88. HRMS: *Chemical formula* ([M]): $C_{14}H_{13}F_5O_2$, calcd. 326.1174 $[M+NH_4]^+$, Found: 326.1180.

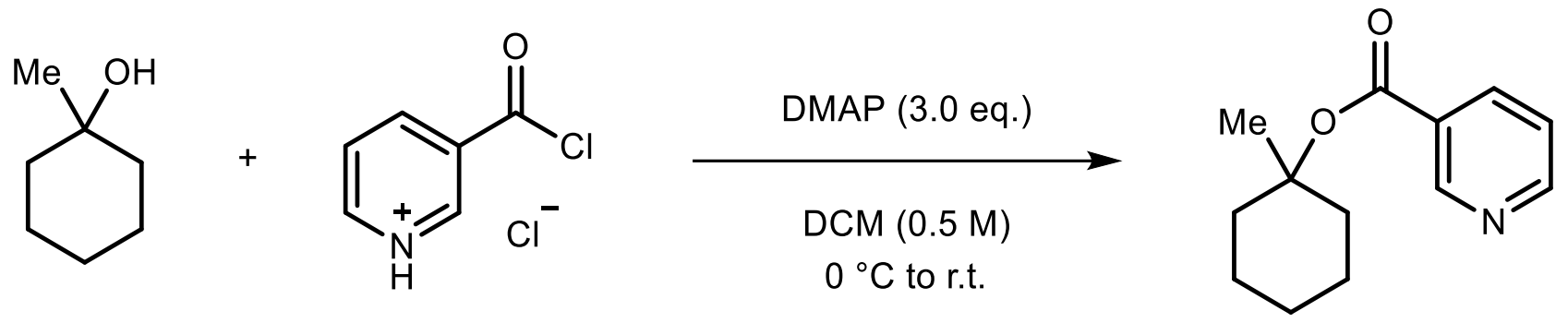

1e

1e: To a DCM (10 mL) solution of 1-methylcyclohexanol (0.77 g, 6.8 mmol, 1.0 equiv) and DMAP (1.83 g, 15 mmol, 2.2 equiv) was added 3-pyridinecarbonylchloride (1.34 g, 7.5 mmol, 1.1 equiv) at 0 °C. The reaction mixture was allowed to warm to room temperature and stirred overnight. The reaction was quenched by the addition of saturated aqueous NH_4Cl, and the mixture was extracted with DCM. The combined organic layers were washed successively with saturated aqueous Na_2CO_3 and brine. The resulting mixture was concentrated *in vacuo*, and the residue was purified by flash column chromatography on silica gel (petroleum ether/ethyl acetate) to give **1e** as a colorless oil (1.21 g, 81% yield). R_f = 0.3 (petroleum ether/ethyl acetate = 10:1). ^{1}H NMR (400 MHz, $CDCl_3$) δ 9.14 (d, *J* = 1.4 Hz, 1H), 8.68 (dd, *J* = 4.9, 1.7 Hz, 1H), 8.20 (dt, *J* = 7.8, 1.9 Hz, 1H), 7.31 (dd, *J* = 7.9, 4.9 Hz, 1H), 2.32 – 2.16 (m, 2H), 1.57 – 1.38 (m, 10H), 1.28 – 1.14 (m, 1H). ^{13}C NMR (101 MHz, $CDCl_3$) δ 164.10, 152.93, 150.82, 136.85, 127.76, 123.18, 83.53, 36.67, 25.74, 25.29, 22.14. HRMS: *Chemical formula* ([M]): $C_{13}H_{17}NO_2$, calcd. 220.1332 $[M+Na]^+$, Found: 220.1327.

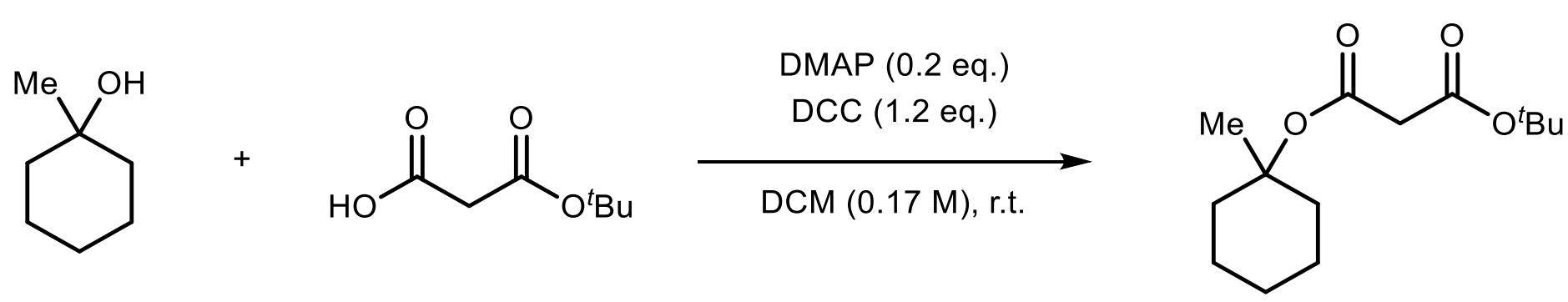

1h

1h: To a DCM (10 mL) solution of 1-methylcyclohexanol (0.57 g, 5 mmol, 1.0 equiv), DMAP (122 mg, 1 mmol, 0.2 equiv) and DCC (1.24 g, 6 mmol, 1.2 equiv) was added 3-*tert*-butoxy-3-oxopropanoic acid (1.08 g, 6 mmol, 1.2 equiv) at room temperature. The reaction mixture was stirred overnight. The reaction was quenched by the addition of saturated aqueous NH_4Cl, and the mixture was extracted with DCM. The combined organic layers were washed successively with saturated aqueous Na_2CO_3 and brine. The resulting mixture were concentrated *in vacuo*, and the residue was purified by flash column chromatography on silica gel (petroleum ether/ethyl acetate) to give **1h** as a

pale yellow oil (1.16 g, 91% yield). R_f = 0.4 (petroleum ether/ethyl acetate = 20:1). ^{1}H NMR (400 MHz, $CDCl_3$) δ 3.17 (s, 2H), 2.16 – 2.04 (m, 2H), 1.58 – 1.30 (m, 20H). ^{13}C NMR (101 MHz, $CDCl_3$) δ 166.18, 166.03, 83.35, 81.66, 44.51, 36.62, 28.01, 25.36, 22.00. HRMS: *Chemical formula* ([M]): $C_{14}H_{24}O_4$, calcd. 257.1747 $[M+H]^+$, Found: 257.1755.

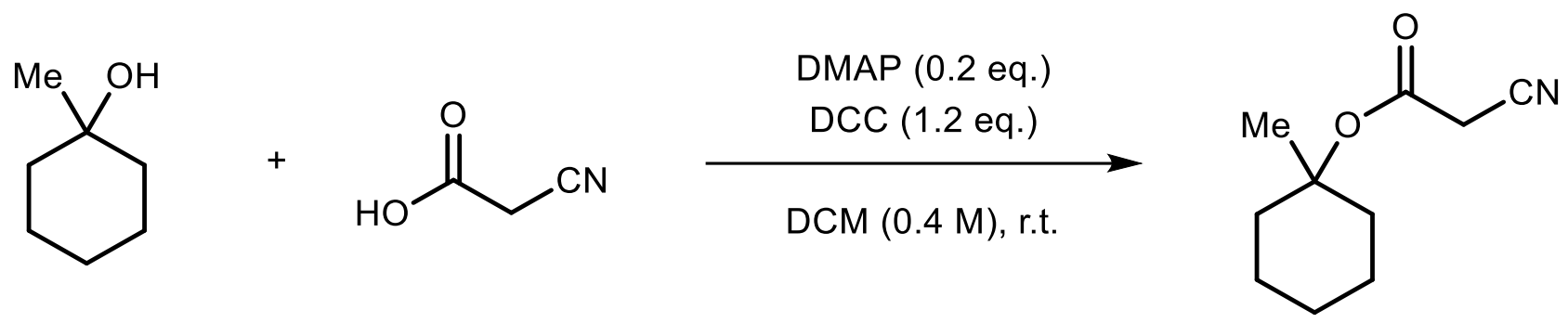

1i

1i: To a DCM (25 mL) solution of 1-methylcyclohexanol (1.42 g, 12.5 mmol, 1.25 equiv), DMAP (244 mg, 2 mmol, 0.2 equiv) and DCC (2.27 g, 11 mmol, 1.1 equiv) was added cyanoacetic acid (0.85 g, 10 mmol, 1.0 equiv) at room temperature. The reaction mixture was stirred overnight. The reaction was quenched by the addition of saturated aqueous NH_4Cl, and the mixture was extracted with DCM. The combined organic layers were washed successively with saturated aqueous Na_2CO_3 and brine. The resulting mixture was concentrated *in vacuo*, and the residue was purified by flash column chromatography on silica gel (petroleum ether/ethyl acetate) to give **1i** as a colorless oil (0.86 g, 48% yield). R_f = 0.4 (petroleum ether/ethyl acetate = 10:1). ^{1}H NMR (400 MHz, $CDCl_3$) δ 3.38 (s, 2H), 2.20 – 2.03 (m, 2H), 1.62 – 1.38 (m, 10H), 1.31 – 1.20 (m, 1H). ^{13}C NMR (101 MHz, $CDCl_3$) δ 161.69, 113.61, 86.08, 36.38, 25.86, 25.31, 25.15, 22.03. HRMS: *Chemical formula* ([M]): $C_{10}H_{15}NO_2$, calcd. 182.1176 $[M+H]^+$, Found: 182.1174.

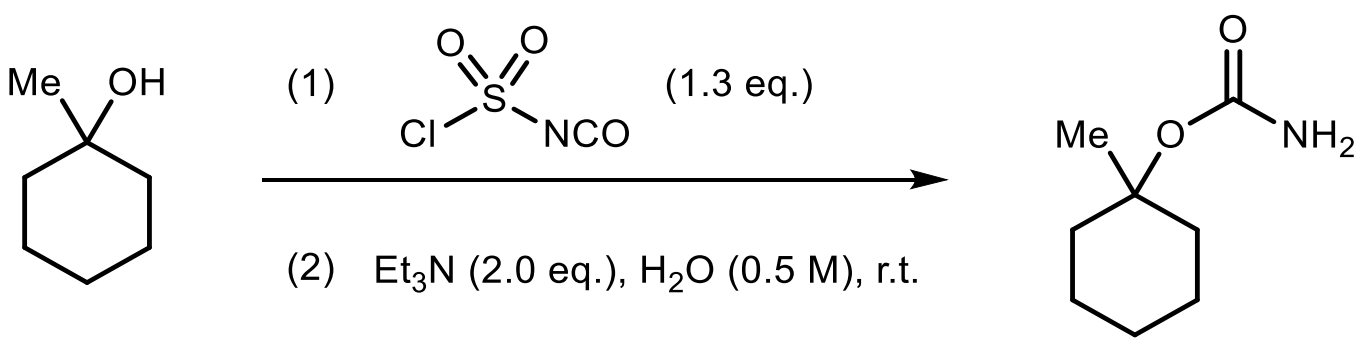

1j

1j: To an ethyl acetate (15 mL) solution of 1-methylcyclohexanol (0.57 g, 5 mmol, 1.0 equiv) was added chlorosulfonyl isocyanate (991 mg, 6.65 mmol, 1.33 equiv) at 0 °C. The reaction mixture was allowed to warm to room temperature and stirred overnight. H_2O (10 mL) was added to the mixture, followed by the dropwise addition of Et_3N (2.03 g, 10 mmol, 2.0 equiv). The reaction was quenched by the addition of saturated aqueous NH_4Cl, and the mixture was extracted with DCM. The combined organic layers were washed successively with saturated aqueous Na_2CO_3 and brine. The resulting mixture was concentrated *in vacuo*, and the residue was purified by flash column chromatography on silica gel (petroleum ether/ethyl acetate) to give **1j** as a white solid (0.64 g, 82% yield). R_f = 0.2 (petroleum ether/ethyl acetate = 1:1). ^{1}H NMR (400 MHz, $CDCl_3$) δ 4.58 (s, 2H), 2.14 – 2.04 (m, 2H), 1.59 – 1.35 (m, 10H), 1.32 – 1.20 (m, 1H). ^{13}C NMR (101 MHz, $CDCl_3$) δ 156.48, 81.15, 36.96, 25.70, 25.56, 22.27. HRMS: *Chemical formula* ([M]): $C_8H_{15}NO_2$, calcd. 97.1017 $[M+H-NH_3-CO_2]^+$, Found: 97.1012.

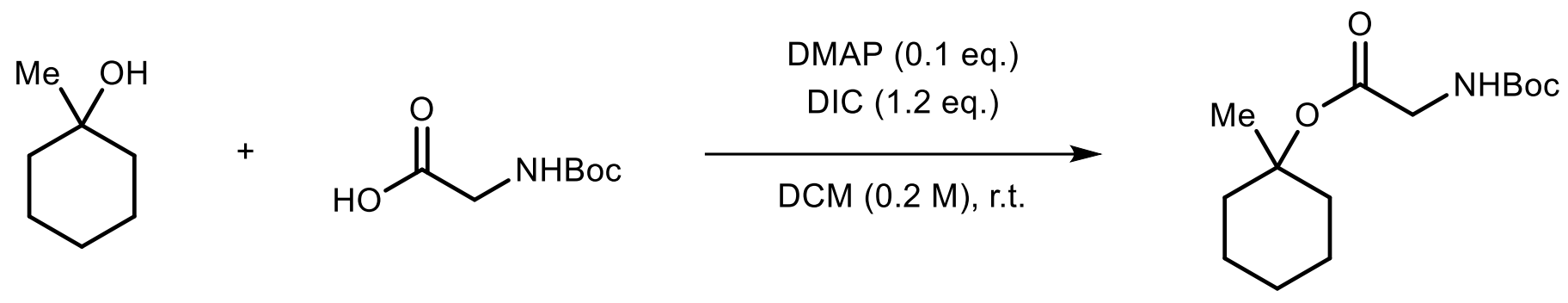

1k: To a DCM (25 mL) solution of 1-methylcyclohexanol (1.14 g, 10 mmol, 1.0 equiv), DMAP (122 mg, 1 mmol, 0.1 equiv) and DIC (1.46 mL, 12 mmol, 1.2 equiv) was added N-*tert*-butyloxycarbonyl glycine (1.75 g, 10 mmol, 1.0 equiv) at room temperature. The reaction mixture was stirred for 12 hours. The reaction was quenched by the addition of saturated aqueous NH_4Cl, and the mixture was extracted with DCM. The combined organic layers were washed successively with saturated aqueous Na_2CO_3 and brine. The resulting mixture was concentrated *in vacuo*, and the residue was purified by flash column chromatography on silica gel (petroleum ether/ethyl acetate) to give **1k** as a colorless oil (0.48 g, 18% yield). R_f = 0.40 (petroleum ether/ethyl acetate = 4:1). ^{1}H NMR (500 MHz, $CDCl_3$) δ 5.00 (s, 1H), 3.81 (d, J = 5.2 Hz, 2H), 2.14 – 2.08 (m, 2H), 1.49 – 1.41 (m, 19H), 1.28 – 1.20 (m, 1H). ^{13}C NMR (126 MHz, $CDCl_3$) δ 169.43, 155.78, 83.62, 79.79, 43.14, 36.67, 28.43, 25.57, 25.34, 22.12. HRMS: *Chemical formula* ([M]): $C_{14}H_{25}NO_4$, calcd. 294.1676 $[M+Na]^+$, Found: 294.1677.

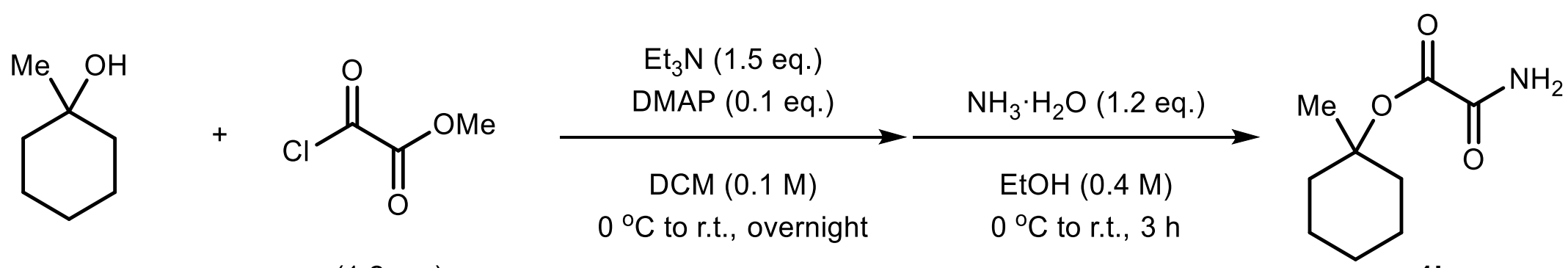

1l: Following a modified literature procedure,[14] 1-methylcyclohexan-1-ol (1.14 g, 10 mmol, 1.0 equiv) was dissolved in DCM (100 mL, 0.1 M). Triethylamine (1.7 mL, 12 mmol, 1.2 equiv) and DMAP (122 mg, 1 mmol, 0.1 equiv) were added. The reaction was cooled to 0 °C, followed by the dropwise addition of methyl chlorooxoacetate (1.1 mL, 12 mmol, 1.2 equiv). The reaction was warmed to room temperature and stirred overnight. The reaction was quenched with saturated aqueous NH_4Cl (20 mL) and extracted with DCM. The combined organic phases were dried over Na_2SO_4 and concentrated *in vacuo*. The residue was used directly in the next step without further purification. To a solution of the crude product in EtOH (25 mL, 0.4 M) cooled at 0 ºC was added aqueous ammonia (14.8 N, 0.81 mL, 12 mmol, 1.2 equiv). The reaction mixture was slowly warmed up to room temperature and stirred for 3 hours. The mixture was concentration under reduced pressure, and the residue was purified by flash column chromatography on silica gel (petroleum ether/ethyl acetate) to give **1l** as a white solid (1.41 g, 76% yield). R_f = 0.20 (petroleum ether/ethyl acetate = 4:1). ^{1}H NMR (500 MHz, $CDCl_3$) δ 6.94 (s, 1H), 6.47 (br, 1H), 2.29 – 2.16 (m, 2H), 1.63 – 1.47 (m, 10H), 1.36 – 1.25 (m, 1H). ^{13}C NMR (126 MHz, $CDCl_3$) δ 159.75, 158.96, 86.60, 36.36, 25.24, 24.90, 22.19. HRMS: *Chemical formula* ([M]): $C_9H_{15}NO_3$, calcd. 208.0944 $[M+Na]^+$, Found: 208.0947.

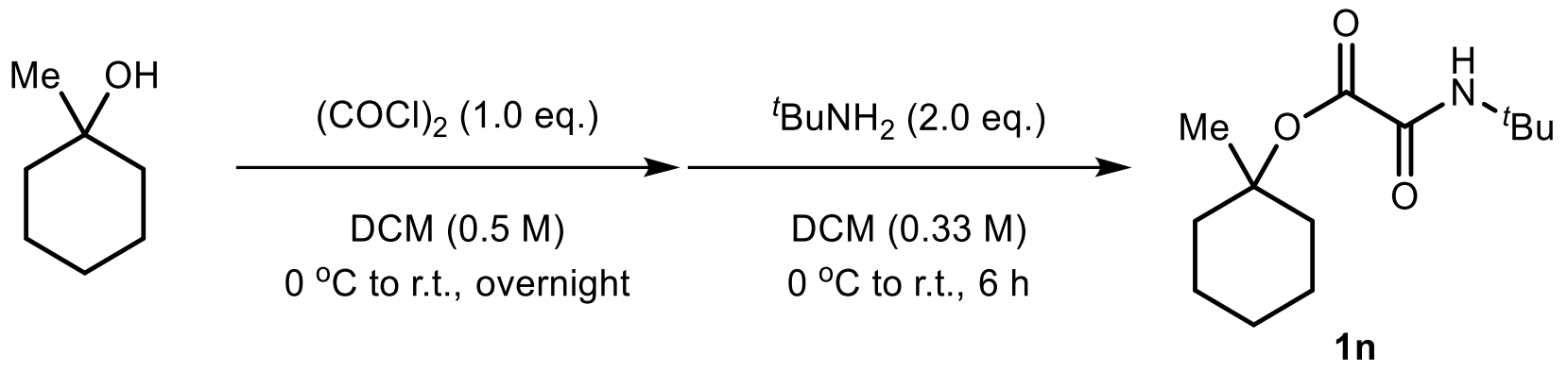

1n: Following a modified literature procedure,[15] an oven-dired flask was evacuated and backfilled with N_2 three times. The flask was charged with 1-methylcyclohexan-1-ol (1.14 g, 10 mmol, 1.0 equiv) and DCM (20 mL). The reaction was cooled to 0 °C, followed by the dropwise addition of oxalyl chloride (1.26 g, 10 mmol, 1.0 equiv). The reaction was warmed to room temperature and stirred overnight. The resulting mixture was concentrated under reduced pressure to afford crude product as a yellow liquid, which was used directly in the next step without further purification. To a solution of *tert*-butylamine (1.46 g, 20 mmol, 2.0 equiv) in DCM (30 mL) at 0 ºC was added the crude product mentioned above. The reaction was warmed to room temperature and stirred for 6 h. The mixture was quenched with saturated aqueous NH_4Cl (20 mL) and extracted with DCM. The combined organic layers were dried over Na_2SO_4 and concentrated. The residue was purified by flash column chromatography on silica gel to give **1n** as a white solid (0.64 g, 27% yield). R_f = 0.30 (petroleum ether/ethyl acetate = 4:1). ^{1}H NMR (500 MHz, $CDCl_3$) δ 6.96 (s, 1H), 2.25 – 2.16 (m, 2H), 1.61 – 1.46 (m, 10H), 1.38 (s, 9H), 1.32 – 1.24 (m, 1H). ^{13}C NMR (126 MHz, $CDCl_3$) δ 160.38, 156.52, 85.95, 51.72, 36.40, 28.37, 25.30, 24.84, 22.17. HRMS: *Chemical formula* ([M]): $C_{13}H_{23}NO_3$, calcd. 242.1751 $[M+H]^+$, Found: 242.1759.

4. Installation of the oxamyl protecting group

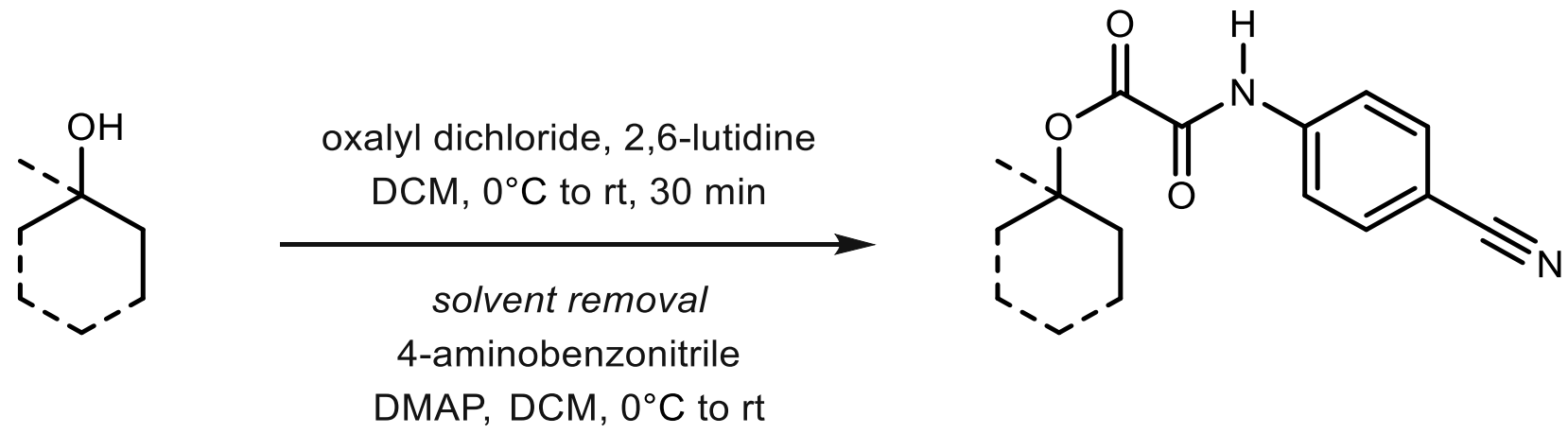

General procedure for the installation of the protecting group: To a DCM (3 mL/mmol alcohol) solution of the alcohol substrate (1.0 equiv) and 2,6-lutidine (1.2 equiv) was added oxalyl chloride (1.2 equiv) dropwise at 0 °C under a N_2 atmosphere. The reaction was allowed to warm to room temperature and stirred for 30 minutes. Upon completion (monitored by TLC), the reaction was concentrated *in vacuo*, and the residue was redissolved in DCM (1 mL/mmol alcohol). To this was then added a solution of 4-aminobenzonitrile (1.2 equiv) and DMAP (1.0 equiv) in DCM (2 mL/mmol alcohol) dropwise at 0 °C. The reaction was allowed to warm to room temperature and stirred for 2 h. Upon completion, the reaction mixture was quenched with a roughly equal volume of 2 M HCl and shaken vigorously. The precipitate was removed by filtration. The organic phase of the filtrate was separated and washed sequentially with 0.1 M HCl and deionized water. The aqueous wash was back-extracted with DCM. The combined organic phases were dried over anhydrous Na_2SO_4 and concentrated *in vacuo*. The crude product was purified either by column chromatography over silica gel or, for clean reactions, by passing through a short silica gel plug to afford the oxamyl-protected alcohol, typically as a white or pale-yellow solid.

For column chromatography purification, the recommended protocol involves pre-cooling the silica gel column prior to sample loading—either by placing it in a -20 °C refrigerator for 20 minutes or by rinsing it with cold solvent—followed by column elution using conventional room-temperature solvents. The standard chromatography procedure without pre-cooling remains straightforward and feasible, though it typically affords isolated yields that are approximately 25% lower, likely owing to partial decomposition during sample loading.

For passing through a short silica gel plug, the crude mixture is diluted with DCM, loaded onto a short pad of silica gel, eluted, and the eluent is concentrated *in vacuo*. As with column chromatography, it is also recommended to pre-chill the silica gel plug and the eluent in a -20 °C refrigerator for 20 minutes prior to use to avoid partial decomposition during sample loading.

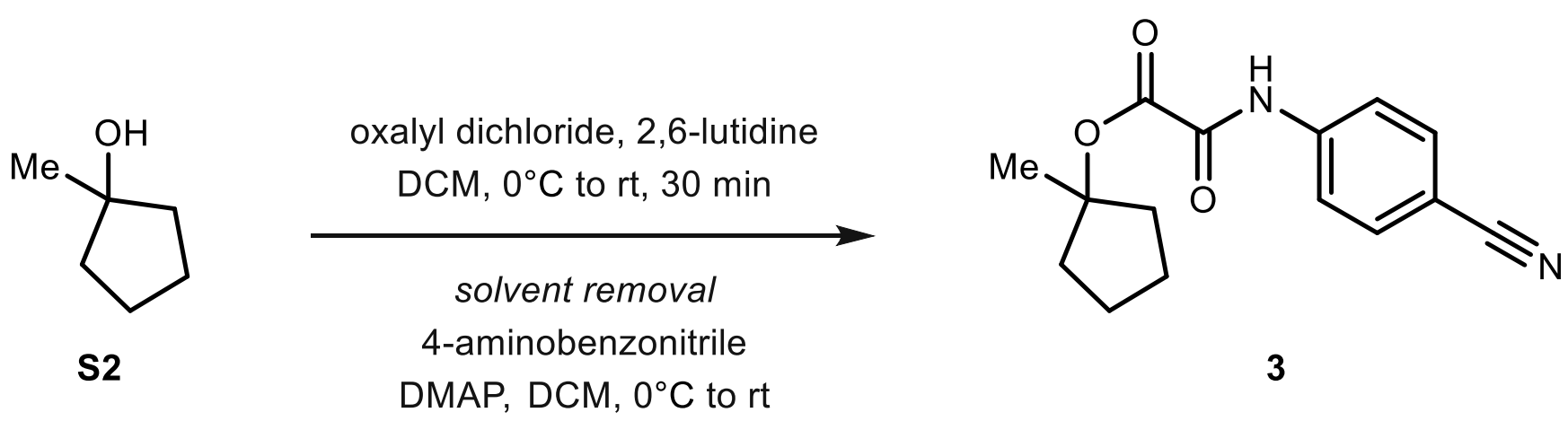

3 was prepared using the *General procedure* from 0.50 g (5.0 mmol) of **S2**. After column chromatography purification (eluent: petroleum ether/ethyl acetate = 4:1 v/v, with 0.1% triethylamine), **3** was obtained as a white solid (1.1 g, 80% yield). R_f = 0.35 (petroleum ether/ethyl acetate = 4:1). ^{1}H NMR (400 MHz, $CDCl_3$) δ 9.09 (s, 1H), 7.77 (d, J = 8.8 Hz, 2H), 7.66 (d, J = 6.7 Hz, 2H), 2.31 – 2.21 (m, 2H), 1.86 – 1.74 (m, 4H), 1.73 – 1.62 (m, 5H). ^{13}C NMR (101 MHz, $CDCl_3$) δ 159.69, 154.99, 140.40, 133.45, 119.77, 118.51, 108.47, 95.14, 38.87, 23.88, 23.74. HRMS: *Chemical formula* ([M]): $C_{15}H_{16}N_2O_3$, calcd. 273.1234 $[M+H]^+$, Found: 273.1239.

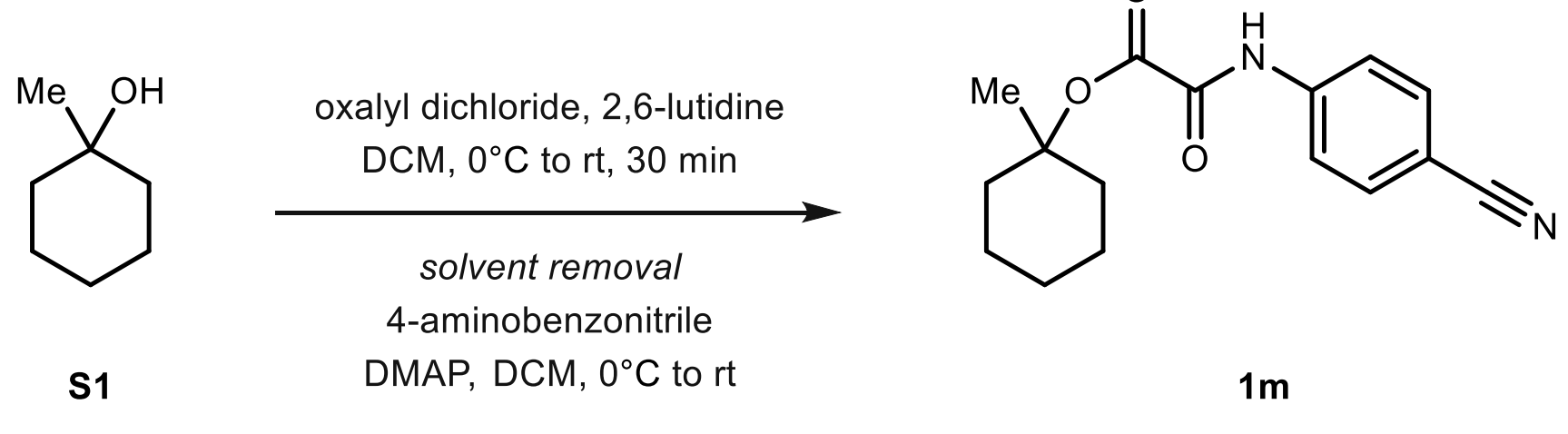

1m was prepared using the *General procedure* from 0.57 g (5.0 mmol) of **S1**. After purification by passing through a pre-chilled short silica gel plug (eluent: petroleum ether/ethyl acetate = 4:1 v/v, 150 mL), **1m** was obtained as a white solid (1.3 g, 88% yield). R_f = 0.40 (petroleum ether/ethyl acetate = 4:1). ^{1}H NMR (400 MHz, $CDCl_3$) δ 9.08 (s, 1H), 7.77 (d, J = 8.8 Hz, 2H), 7.66 (d, J = 8.7 Hz, 2H), 2.29 – 2.21 (m, 2H), 1.61 – 1.53 (m, 10H), 1.36 – 1.28 (m, 1H). ^{13}C NMR (101 MHz, $CDCl_3$) δ 159.15, 155.07, 140.45, 133.44, 119.74, 118.52, 108.43, 87.66, 36.26, 25.07, 24.85, 22.05. HRMS: *Chemical formula* ([M]): $C_{16}H_{18}N_2O_3$, calcd. 287.1390 $[M+H]^+$, Found: 287.1394.

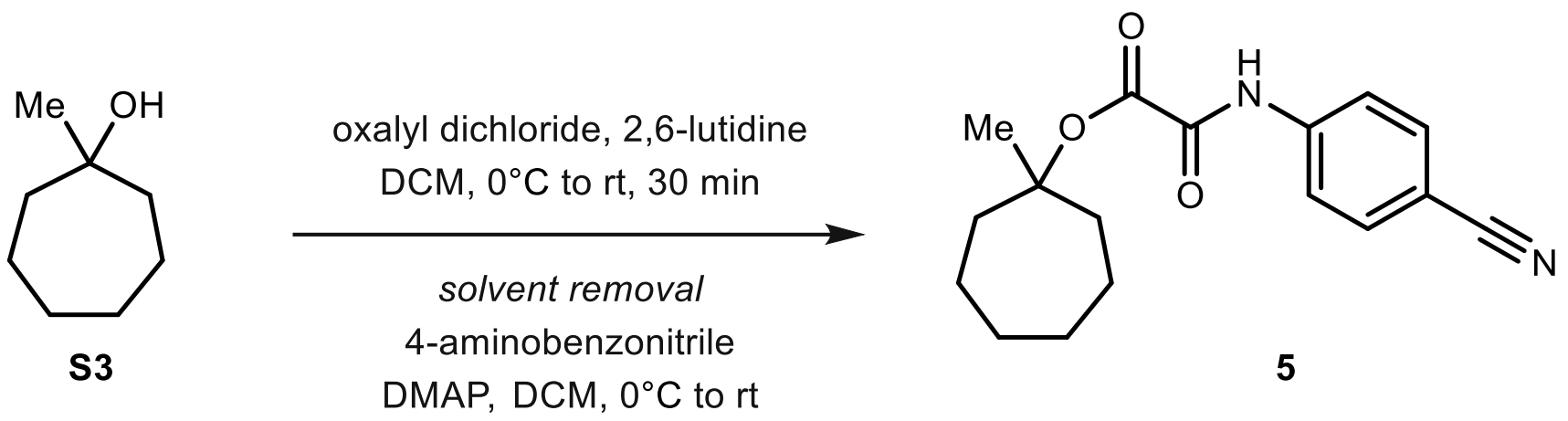

S3

5

5 was prepared using the *General procedure* from 0.64 g (5.0 mmol) of **S3**. After column chromatography purification (eluent: petroleum ether/ethyl acetate = 4:1 v/v, with 0.1% triethylamine), **5** was obtained as a white solid (1.1 g, 71% yield). R_f = 0.42 (petroleum ether/ethyl acetate = 4:1). ^{1}H NMR (400 MHz, $CDCl_3$) δ 9.11 (s, 1H), 7.78 (d, J = 8.7 Hz, 2H), 7.67 (d, J = 8.7 Hz, 2H), 2.26 (ddd, J = 14.8, 8.7, 1.7 Hz, 2H), 1.89 (ddd, J = 14.8, 9.7, 1.9 Hz, 2H), 1.69 – 1.54 (m, 9H), 1.52 – 1.43 (m, 2H). ^{13}C NMR (101 MHz, $CDCl_3$) δ 159.35, 155.19, 140.48, 133.44, 119.74, 118.53, 108.40, 92.12, 39.84, 29.38, 26.42, 22.57. HRMS: *Chemical formula* ([M]): $C_{17}H_{20}N_2O_3$, calcd. 301.1547 $[M+H]^+$, Found: 301.1555.

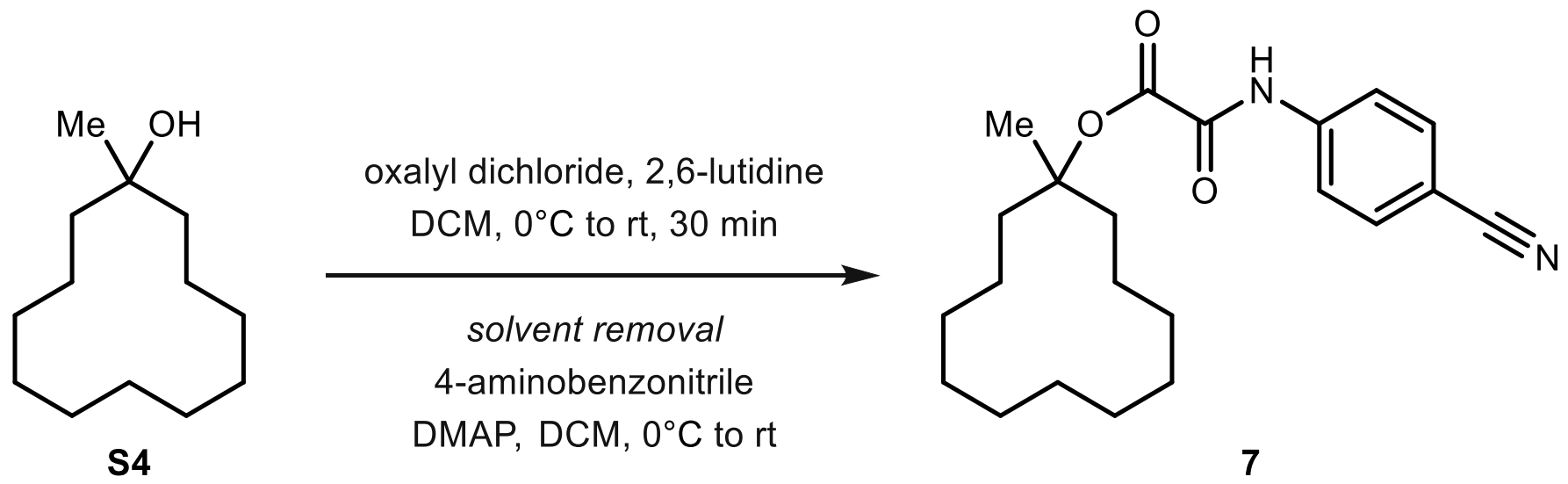

S4

7

7 was prepared using the *General procedure* from 0.60 g (3.0 mmol) of **S4**. After column chromatography purification (eluent: petroleum ether/ethyl acetate = 4:1 v/v, with 0.1% triethylamine), **7** was obtained as a white solid (0.78 g, 70% yield). R_f = 0.45 (petroleum ether/ethyl acetate = 4:1). ^{1}H NMR (400 MHz, $CDCl_3$) δ 9.06 (s, 1H), 7.76 (d, J = 8.7 Hz, 2H), 7.66 (d, J = 8.7 Hz, 2H), 2.14 – 2.02 (m, 2H), 1.79 – 1.69 (m, 2H), 1.58 (s, 3H), 1.53 – 1.44 (m, 2H), 1.41 – 1.26 (m, 16H). ^{13}C NMR (101 MHz, $CDCl_3$) δ 159.02, 155.04, 140.42, 133.45, 119.71, 118.51, 108.43, 91.72, 32.84, 26.05, 26.00, 23.83, 22.29, 21.89, 19.46. HRMS: *Chemical formula* ([M]): $C_{22}H_{30}N_2O_3$, calcd. 371.2329 $[M+H]^+$, Found: 371.2327.

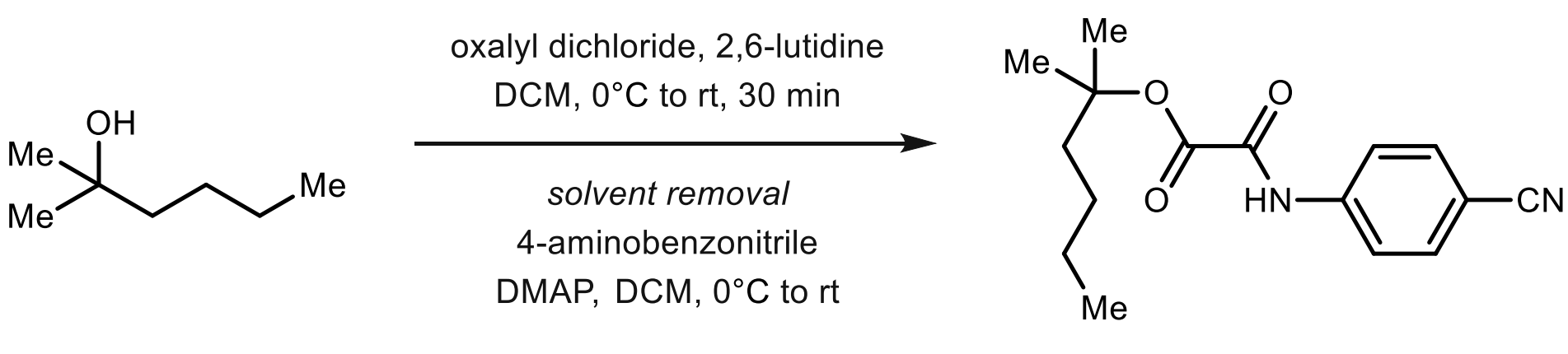

S5

9

9 was prepared using the *General procedure* from 0.39 g (3.4 mmol) of **S5**. After column chromatography purification (eluent: petroleum ether/ethyl acetate = 4:1 v/v, with 0.1% triethylamine), **9** was obtained as a white solid (0.84 g,

86% yield). R_f = 0.40 (petroleum ether/ethyl acetate = 4:1). ^{1}H NMR (400 MHz, $CDCl_3$) δ 9.09 (s, 1H), 7.78 (d, *J* = 7.7 Hz, 2H), 7.67 (d, *J* = 7.7 Hz, 2H), 1.91 – 1.84 (m, 2H), 1.57 (s, 6H), 1.35 (q, *J* = 4.1 Hz, 4H), 0.94 – 0.89 (m, 3H). ^{13}C NMR (101 MHz, $CDCl_3$) δ 159.27, 155.06, 140.44, 133.44, 119.74, 118.51, 108.43, 88.36, 40.21, 26.03, 25.61, 22.89, 13.98. HRMS: *Chemical formula* ([M]): $C_{16}H_{20}N_2O_3$, calcd. 289.1547 $[M+H]^+$, Found: 289.1555.

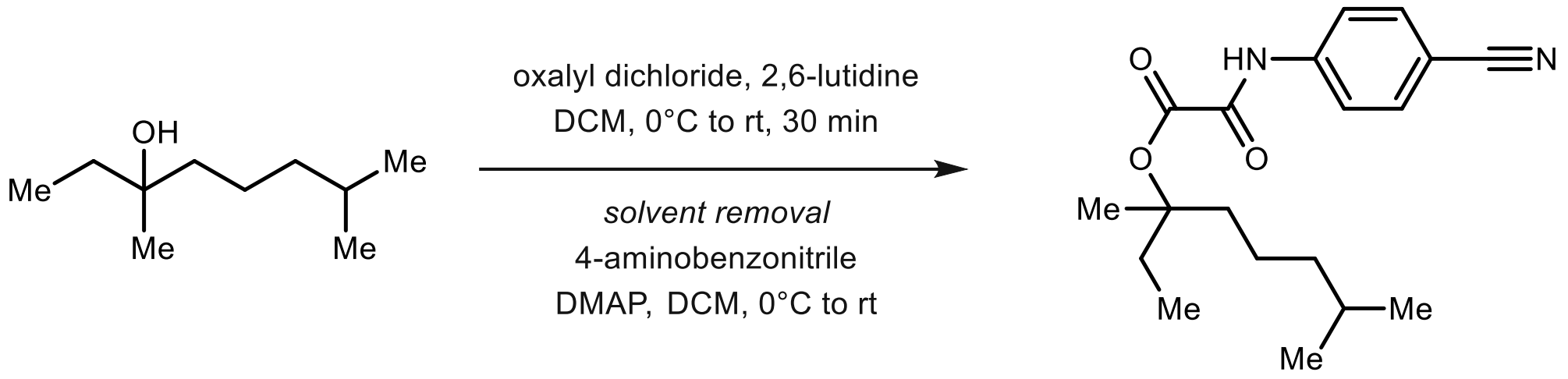

S6 **11**

11 was prepared using the *General procedure* from 0.79 g (5.0 mmol) of **S6**. After purification by passing through a pre-chilled short silica gel plug (eluent: petroleum ether/ethyl acetate = 4:1 v/v, 150 mL), **11** was obtained as a white solid (1.5 g, 92% yield). R_f = 0.40 (petroleum ether/ethyl acetate = 4:1). ^{1}H NMR (400 MHz, $CDCl_3$) δ 9.06 (s, 1H), 7.77 (d, *J* = 8.7 Hz, 2H), 7.66 (d, *J* = 8.8 Hz, 2H), 2.05 – 1.77 (m, 4H), 1.54 (s, 4H), 1.37 – 1.28 (m, 2H), 1.22 – 1.15 (m, 2H), 0.93 (t, *J* = 7.5 Hz, 3H), 0.88 (s, 3H), 0.86 (s, 3H). ^{13}C NMR (101 MHz, $CDCl_3$) δ 159.14, 154.97, 140.43, 133.45, 119.71, 118.51, 108.43, 91.27, 39.10, 37.66, 30.64, 27.77, 22.99, 22.56, 21.37, 8.06. HRMS: *Chemical formula* ([M]): $C_{19}H_{26}N_2O_3$, calcd. 348.2281 $[M+NH_4]^+$, Found: 348.2278.

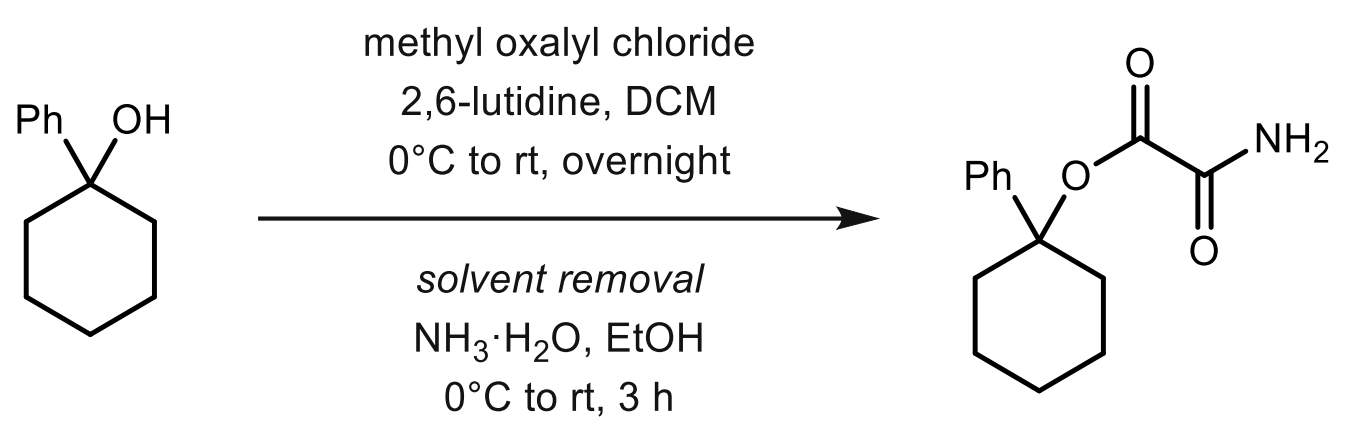

S7 **13**

13: To a 40 mL vial purged with N_2, **S7** (0.60 g, 3.4 mmol, 1.0 equiv) was dissolved in DCM (3 mL/mmol, relative to the alcohol). Methyl oxalyl chloride (0.48 g, 4.1 mmol, 1.2 equiv) and 2,6-lutidine (0.5 mL, 4.1 mmol, 1.2 equiv) were added dropwise at 0 °C (ice-water bath) with stirring. The reaction mixture was stirred at 0 °C to room temperature overnight and then concentrated *in vacuo*. The residue was diluted with 10 mL ethanol. Aqueous ammonia (conc., 0.5 mL, 2.4 equiv $NH_3{\cdot}H_2O$) was added dropwise to the reaction mixture at 0 °C. The resulting mixture was stirred at room temperature for 3 h. Upon completion, the reaction mixture was concentrated in vacuo. The crude mixture was purified by silica gel flash column chromatography to give pure **13** as a white solid (0.44 g, 53% yield). R_f = 0.15 (petroleum ether/ethyl acetate = 4:1). ^{1}H NMR (400 MHz, $CDCl_3$) δ 7.42 (m, 2H), 7.37 – 7.32 (m, 2H), 7.30 – 7.26 (m, 1H), 6.80 (s, 1H), 5.90 (s, 1H), 2.64 – 2.56 (m, 2H), 1.93 – 1.84 (m, 2H), 1.79 – 1.66 (m, 5H), 1.39 – 1.27 (m, 1H). ^{13}C NMR (101 MHz, $CDCl_3$) δ 158.92, 158.68, 143.59, 128.46, 127.70, 124.92, 86.64,

35.81, 25.13, 22.04. HRMS: *Chemical formula* ([M]): $C_{14}H_{17}NO_3$, calcd. 270.1100 $[M+Na]^+$, Found: 270.1107 .

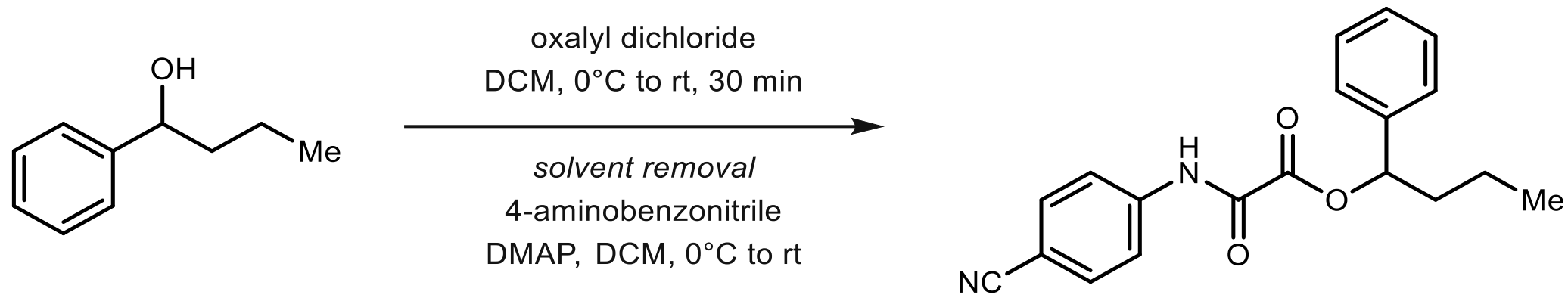

45 **15**

15 was prepared using the modified *General procedure* from 0.75 g (5.0 mmol) of **45** in which 2,6-lutidine was omitted. After column chromatography purification (eluent: petroleum ether/ethyl acetate = 4:1 v/v, with 0.1% triethylamine), **15** was obtained as a white solid (0.92 g, 57% yield). R_f = 0.40 (petroleum ether/ethyl acetate = 4:1). ^{1}H NMR (400 MHz, $CDCl_3$) δ 8.99 (s, 1H), 7.74 (d, *J* = 8.8 Hz, 2H), 7.65 (d, *J* = 8.8 Hz, 2H), 7.42 – 7.30 (m, 5H), 5.86 (dd, *J* = 8.0, 6.2 Hz, 1H), 2.18 – 2.07 (m, 1H), 1.91 (ddt, *J* = 13.9, 10.0, 6.0 Hz, 1H), 1.51 – 1.39 (m, 1H), 1.39 – 1.25 (m, 1H), 0.95 (t, *J* = 7.4 Hz, 3H). ^{13}C NMR (101 MHz, $CDCl_3$) δ 160.01, 154.11, 140.20, 138.88, 133.46, 128.69, 126.77, 119.80, 118.43, 108.66, 80.61, 37.94, 18.82, 13.66. HRMS: *Chemical formula* ([M]): $C_{19}H_{18}N_2O_3$, calcd. 323.1390 $[M+H]^+$, Found: 323.1388.

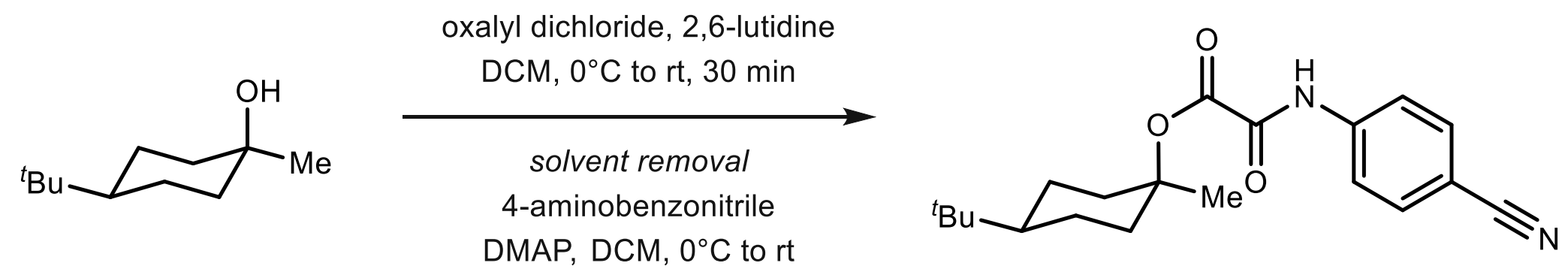

43 **17**

17 was prepared using the *General procedure* from 0.51 g (3.0 mmol) of **43**. After column chromatography purification (eluent: petroleum ether/ethyl acetate = 4:1 v/v, with 0.1% triethylamine), **17** was obtained as a white solid (0.82 g, 80% yield). R_f = 0.40 (petroleum ether/ethyl acetate = 4:1). ^{1}H NMR (400 MHz, $CDCl_3$) δ 9.06 (s, 1H), 7.77 (d, *J* = 8.7 Hz, 2H), 7.67 (d, *J* = 8.7 Hz, 2H), 2.54 – 2.46 (m, 2H), 1.68 – 1.61 (m, 2H), 1.57 (s, 3H), 1.42 – 1.22 (m, 4H), 1.04 (tt, *J* = 10.5, 2.8 Hz, 1H), 0.85 (s, 9H). ^{13}C NMR (101 MHz, $CDCl_3$) δ 159.22, 154.93, 140.45, 133.45, 119.71, 118.51, 108.43, 86.71, 47.02, 36.58, 32.35, 27.50, 25.65, 22.46. HRMS: *Chemical formula* ([M]): $C_{20}H_{26}N_2O_3$, calcd. 343.2016 $[M+H]^+$, Found: 343.2013.

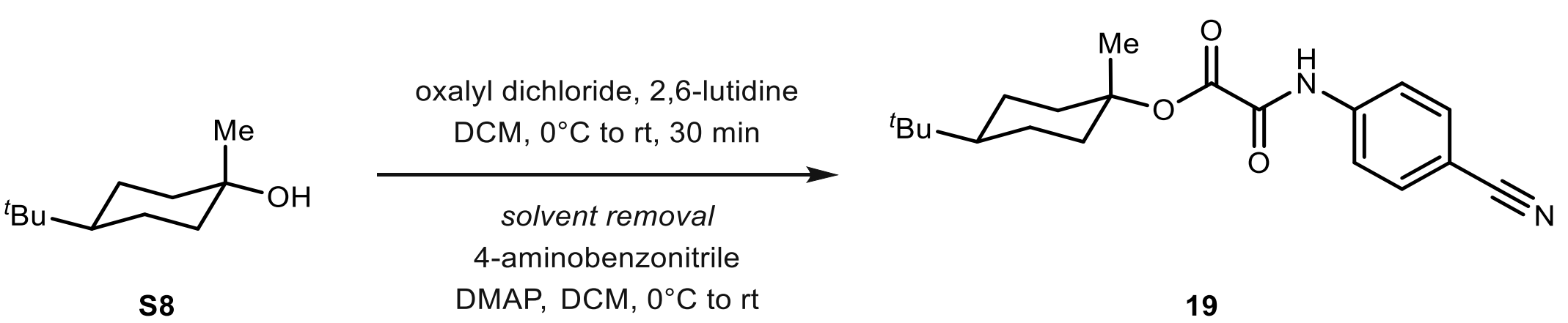

S8 **19**

19 was prepared using the *General procedure* from 0.51 g (3.0 mmol) of **S8**. After column chromatography purification (eluent: petroleum ether/ethyl acetate = 4:1 v/v, with 0.1% triethylamine), 1**9** was obtained as a white solid (0.58 g, 57% yield). R_f = 0.42 (petroleum ether/ethyl acetate = 4:1). ^{1}H NMR (400 MHz, $CDCl_3$) δ 9.09 (s, 1H), 7.77 (d, *J* = 8.7 Hz, 2H), 7.65 (d, *J* = 8.5 Hz, 2H), 2.34 – 2.25 (m, 2H), 1.81 – 1.72 (m, 4H), 1.61 (s, 3H), 1.25 (s, 2H), 1.22 – 1.03 (m, 4H), 0.86 (s, 9H). ^{13}C NMR (101 MHz, $CDCl_3$) δ 159.19, 155.19, 140.45, 133.43, 119.79, 119.77, 118.53, 108.42, 89.06, 47.30, 36.71, 32.23, 27.53, 24.31, 21.14. HRMS: *Chemical formula* ([M]): $C_{20}H_{26}N_2O_3$, calcd. 343.2016 $[M+H]^+$, Found: 343.2023.

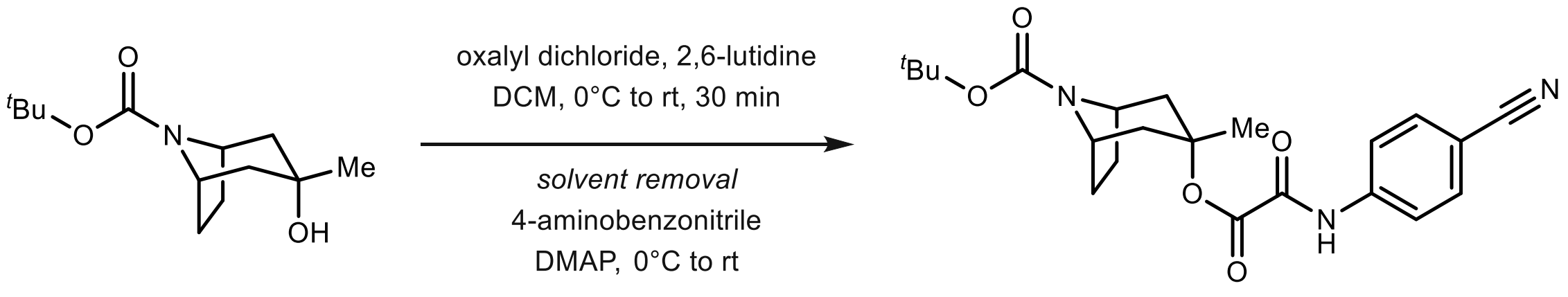

S9 **21**

21 was prepared using the *General procedure* from 0.72 g (3.0 mmol) of **S9**. After column chromatography purification (eluent: petroleum ether/ethyl acetate = 2:1 v/v, with 0.1% triethylamine), **21** was obtained as a white solid (0.28 g, 23% yield). R_f = 0.35 (petroleum ether/ethyl acetate = 2:1). ^{1}H NMR (400 MHz, $CDCl_3$) δ 9.04 (s, 1H), 7.77 (d, *J* = 8.8 Hz, 2H), 7.67 (d, *J* = 8.7 Hz, 2H), 4.30 – 4.14 (m, 2H), 2.47 (d, *J* = 15.0 Hz, 2H), 2.03 – 1.85 (m, 6H), 1.58 (s, 3H), 1.47 (s, 9H). ^{13}C NMR (101 MHz, $CDCl_3$) δ 159.48, 154.71, 153.32, 140.23, 133.48, 119.74, 118.42, 108.66, 86.34, 79.58, 28.48, 27.67. HRMS: *Chemical formula* ([M]): $C_{22}H_{27}N_3O_5$, calcd. 414.2024 $[M+H]^+$, Found: 414.2018.

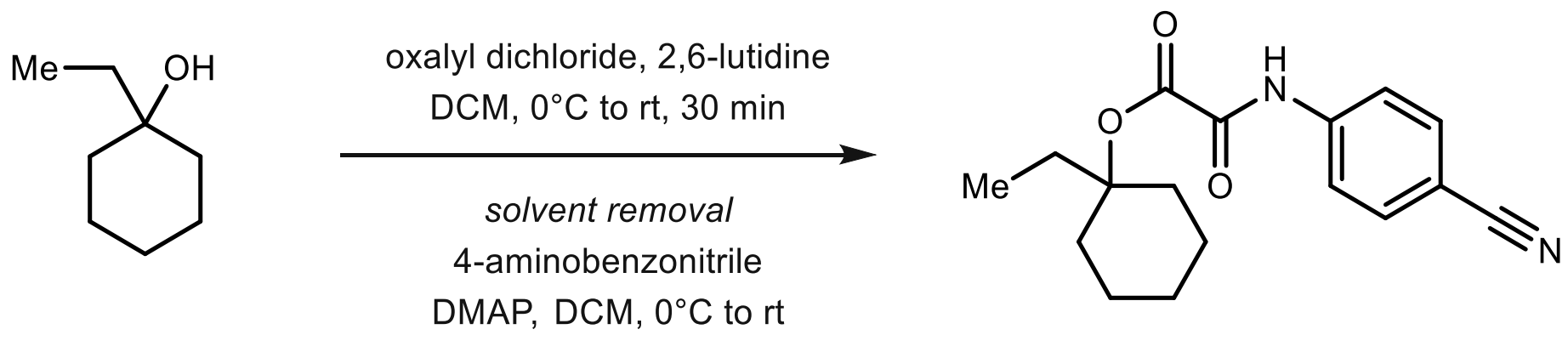

S10 **23**

23 was prepared using the *General procedure* from 0.64 g (5.0 mmol) of **S10**. After column chromatography purification (eluent: petroleum ether/ethyl acetate = 4:1 v/v, with 0.1% triethylamine), **23** was obtained as a white solid (1.1 g, 76% yield). R_f = 0.39 (petroleum ether/ethyl acetate = 4:1). ^{1}H NMR (400 MHz, $CDCl_3$) δ 9.07 (s, 1H), 7.78 (d, *J* = 8.8 Hz, 2H), 7.66 (d, *J* = 8.8 Hz, 2H), 2.36 – 2.22 (m, 2H), 2.00 (q, *J* = 7.5 Hz, 2H), 1.66 – 1.46 (m, 7H), 1.30 (tt, *J* = 13.0, 7.4 Hz, 1H), 0.88 (t, *J* = 7.5 Hz, 3H). ^{13}C NMR (101 MHz, $CDCl_3$) δ 158.94, 154.96, 140.45, 133.45, 119.71, 118.52, 108.43, 90.50, 33.73, 29.81, 25.30, 21.82, 7.49. HRMS: *Chemical formula* ([M]): $C_{17}H_{20}N_2O_3$, calcd. 301.1547 $[M+H]^+$, Found: 301.1548.

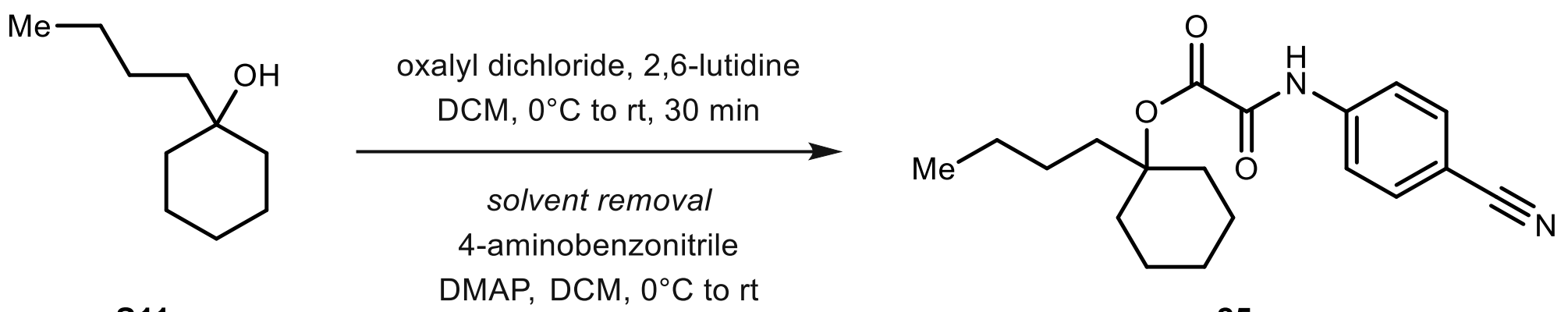

S11 25

25 was prepared using the *General procedure* from 0.78 g (5.0 mmol) of **S11**. After column chromatography purification (eluent: petroleum ether/ethyl acetate = 4:1 v/v, with 0.1% triethylamine), **25** was obtained as a white solid (1.1 g, 65% yield). R_f = 0.43 (petroleum ether/ethyl acetate = 4:1). ^{1}H NMR (400 MHz, $CDCl_3$) δ 9.10 (s, 1H), 7.78 (d, *J* = 8.5 Hz, 2H), 7.66 (d, *J* = 8.5 Hz, 2H), 2.37 – 2.23 (m, 2H), 1.99 – 1.88 (m, 2H), 1.64 – 1.46 (m, 7H), 1.33 – 1.22 (m, 5H), 0.88 (t, *J* = 6.6 Hz, 3H). ^{13}C NMR (101 MHz, $CDCl_3$) δ 158.95, 155.00, 140.49, 133.43, 119.73, 118.53, 108.39, 90.29, 36.94, 34.19, 25.29, 25.22, 22.94, 21.84, 13.97. HRMS: *Chemical formula* ([M]): $C_{19}H_{24}N_2O_3$, calcd. 329.1860 [M+H]$^+$, Found: 329.1865.

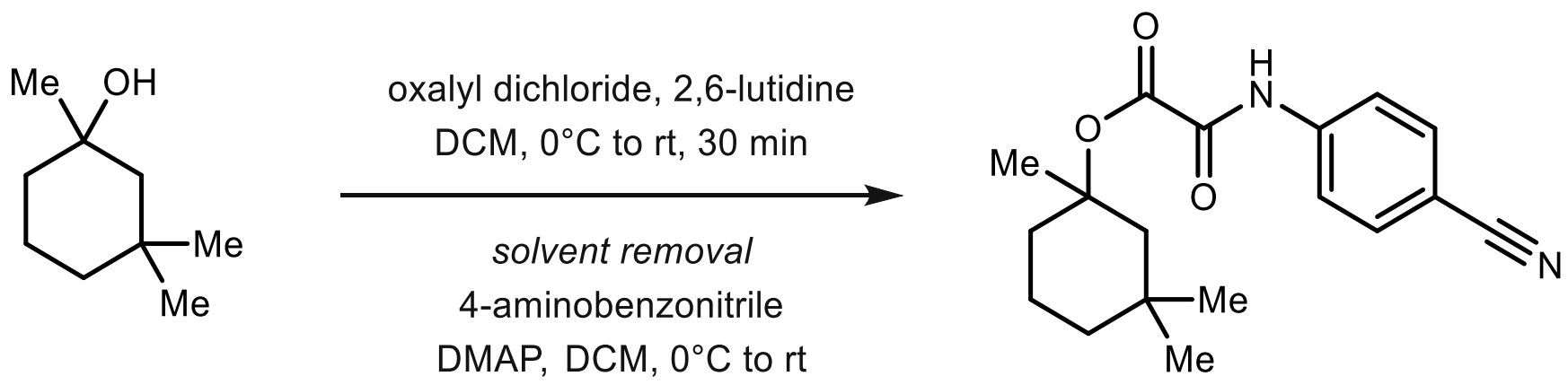

S12 27

27 was prepared using the *General procedure* from 0.71 g (5.0 mmol) of **S12**. After column chromatography purification (eluent: petroleum ether/ethyl acetate = 4:1 v/v, with 0.1% triethylamine), **27** was obtained as a white solid (1.2 g, 75% yield). R_f = 0.40 (petroleum ether/ethyl acetate = 4:1). ^{1}H NMR (400 MHz, $CDCl_3$) δ 9.13 (s, 1H), 7.78 (d, *J* = 7.4 Hz, 2H), 7.65 (d, *J* = 7.3 Hz, 2H), 2.35 – 2.23 (m, 2H), 1.81 – 1.66 (m, 1H), 1.56 (s, 3H), 1.55 – 1.49 (m, 1H), 1.47 – 1.41 (m, 1H), 1.35 – 1.23 (m, 2H), 1.15 (td, *J* = 12.9, 3.8 Hz, 1H), 0.99 (s, 3H), 0.91 (s, 3H). ^{13}C NMR (101 MHz, $CDCl_3$) δ 159.41, 155.03, 140.45, 133.41, 119.72, 118.51, 108.38, 87.62, 46.78, 38.42, 36.83, 33.53, 30.85, 26.63, 26.10, 18.48. HRMS: *Chemical formula* ([M]): $C_{18}H_{22}N_2O_3$, calcd. 315.1703 [M+H]$^+$, Found: 315.1701.

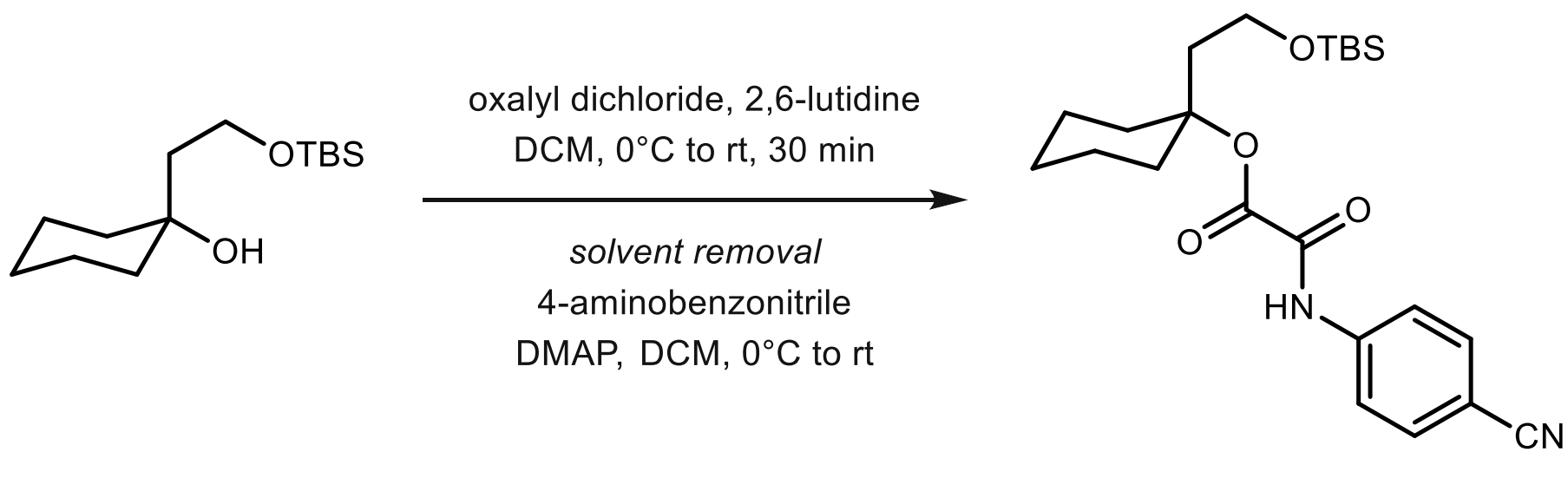

S13 29

29 was prepared using the *General procedure* from 1.3 g (5.0 mmol) of **S13**. After column chromatography

purification (eluent: petroleum ether/ethyl acetate = 4:1 v/v, with 0.1% triethylamine), **29** was obtained as a white solid (1.1 g, 52% yield). R_f = 0.39 (petroleum ether/ethyl acetate = 4:1). ^{1}H NMR (400 MHz, $CDCl_3$) δ 9.05 (s, 1H), 7.76 (d, J = 8.8 Hz, 2H), 7.66 (d, J = 8.8 Hz, 2H), 3.73 (t, J = 6.6 Hz, 2H), 2.39 – 2.28 (m, 2H), 2.23 (t, J = 6.6 Hz, 2H), 1.65 – 1.52 (m, 7H), 1.37 – 1.26 (m, 1H), 0.85 (s, 9H), 0.01 (s, 6H). ^{13}C NMR (101 MHz, $CDCl_3$) δ 159.06, 154.89, 140.44, 133.46, 119.69, 118.51, 108.43, 89.00, 58.49, 39.58, 34.53, 25.89, 25.20, 21.78, 18.27, -5.42. HRMS: *Chemical formula* ([M]): $C_{23}H_{34}N_2O_4Si$, calcd. 431.2361 $[M+NH_4]^+$, Found: 431.2356.

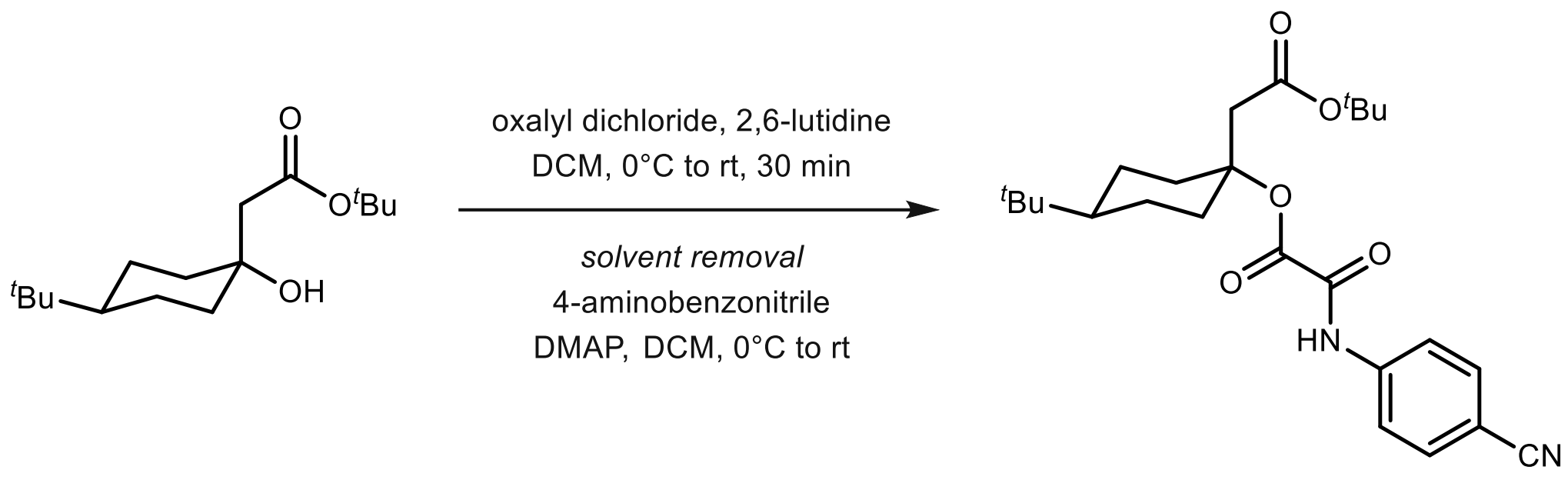

S14 **31**

31 was prepared using the *General procedure* from 1.1 g (4.0 mmol) of **S14**. After column chromatography purification (eluent: petroleum ether/ethyl acetate = 4:1 v/v, with 0.1% triethylamine), **31** was obtained as a white solid (1.3 g, 76% yield). R_f = 0.40 (petroleum ether/ethyl acetate = 4:1). ^{1}H NMR (400 MHz, $CDCl_3$) δ 9.44 (s, 1H), 7.81 (d, J = 8.8 Hz, 2H), 7.66 (d, J = 8.7 Hz, 2H), 2.95 (s, 2H), 2.36 – 2.29 (m, 2H), 2.04 – 1.96 (m, 2H), 1.86 – 1.79 (m, 2H), 1.44 (s, 9H), 1.23 – 1.13 (m, 3H), 0.88 (s, 9H). ^{13}C NMR (101 MHz, $CDCl_3$) δ 169.86, 158.21, 155.32, 140.78, 133.42, 119.72, 118.61, 108.27, 87.50, 81.77, 46.89, 39.11, 34.65, 32.25, 28.03, 27.51, 24.07. HRMS: *Chemical formula* ([M]): $C_{25}H_{34}N_2O_5$, calcd. 443.2541 $[M+H]^+$, Found: 443.2545.

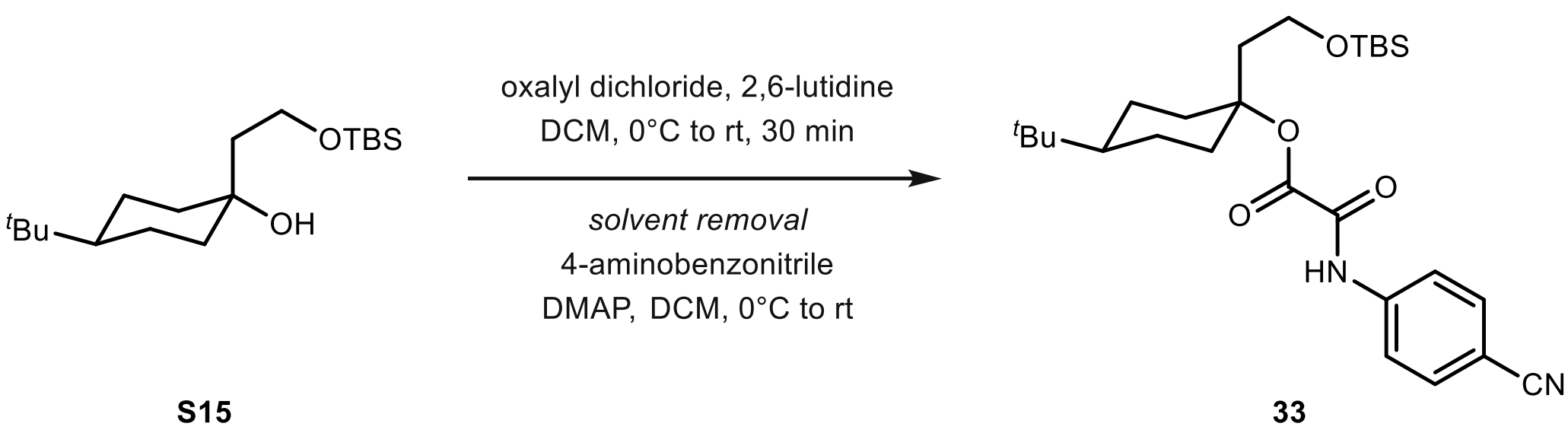

S15 **33**

33 was prepared using the *General procedure* from 0.94 g (3.0 mmol) of **S15**. After column chromatography purification (eluent: petroleum ether/ethyl acetate = 4:1 v/v, with 0.1% triethylamine), **33** was obtained as a white solid (1.1 g, 73% yield). R_f = 0.38 (petroleum ether/ethyl acetate = 4:1). ^{1}H NMR (400 MHz, $CDCl_3$) δ 9.02 (s, 1H), 7.75 (d, J = 8.7 Hz, 2H), 7.66 (d, J = 8.7 Hz, 2H), 3.75 (t, J = 6.5 Hz, 2H), 2.45 – 2.37 (m, 2H), 2.31 (t, J = 6.5 Hz, 2H), 1.84 – 1.73 (m, 4H), 1.26 – 1.07 (m, 4H), 0.87 (s, 9H), 0.86 (s, 9H), 0.02 (s, 6H). ^{13}C NMR (101 MHz, $CDCl_3$) δ 159.09, 154.94, 140.41, 133.46, 119.70, 118.51, 108.43, 90.26, 58.58, 47.08, 34.94, 34.46, 32.26, 27.54, 27.23,

25.90, 24.16, 18.30, -5.41. HRMS: *Chemical formula* ([M]): $C_{27}H_{42}N_2O_4Si$, calcd. 487.2987 $[M+H]^+$, Found: 487.2979.

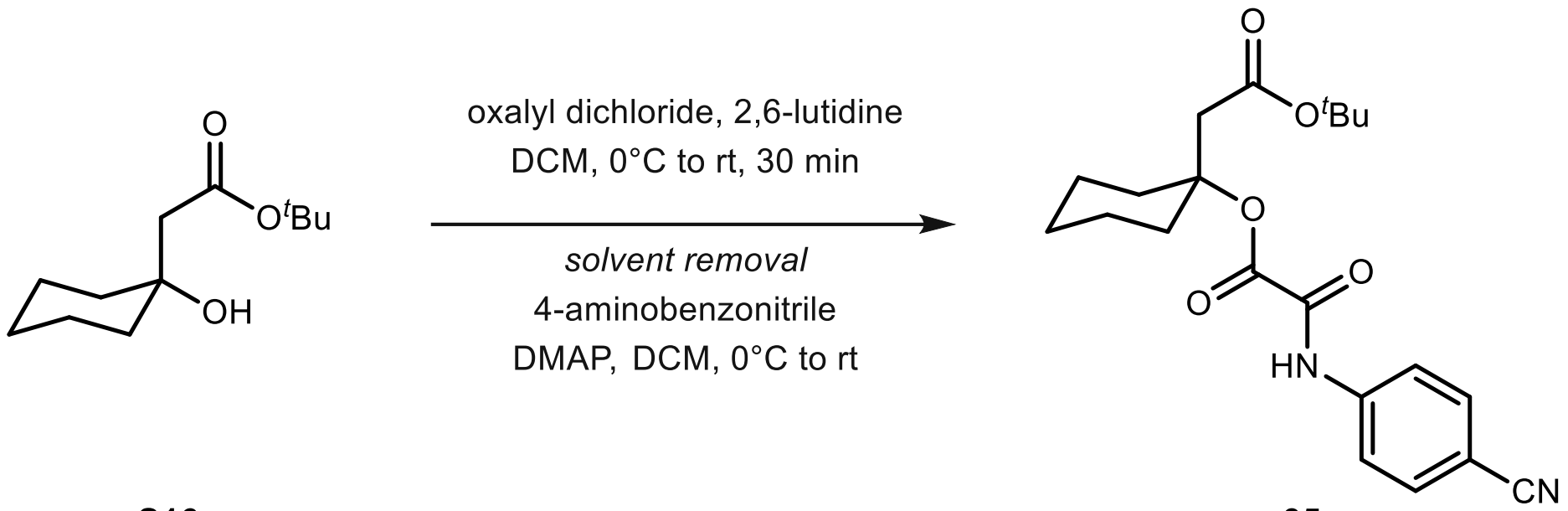

S16 **35**

35 was prepared using the *General procedure* from 1.1 g (5.0 mmol) of **S16**. After column chromatography purification (eluent: petroleum ether/ethyl acetate = 4:1 v/v, with 0.1% triethylamine), **35** was obtained as a white solid (1.1 g, 56% yield). R_f = 0.40 (petroleum ether/ethyl acetate = 4:1). ^{1}H NMR (400 MHz, $CDCl_3$) δ 9.28 (s, 1H), 7.78 (d, *J* = 8.8 Hz, 2H), 7.66 (d, *J* = 8.8 Hz, 2H), 2.90 (s, 2H), 2.40 – 2.30 (m, 2H), 1.71 – 1.55 (m, 8H), 1.42 (s, 9H). ^{13}C NMR (101 MHz, $CDCl_3$) δ 169.32, 158.98, 155.28, 140.64, 133.44, 119.72, 118.57, 108.33, 86.12, 81.58, 42.68, 34.47, 28.02, 24.91, 21.78. HRMS: *Chemical formula* ([M]): $C_{21}H_{26}N_2O_5$, calcd. 387.1915 $[M+H]^+$, Found: 387.1909.

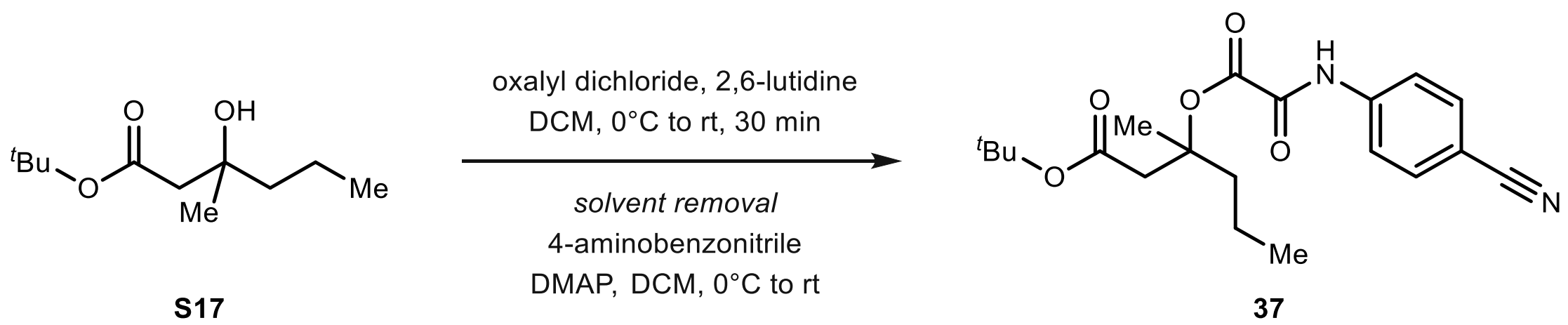

S17 **37**

37 was prepared using the *General procedure* from 1.0 g (5.0 mmol) of **S17**. After purification by passing through a pre-chilled short silica gel plug (eluent: petroleum ether/ethyl acetate = 4:1 v/v, 150 mL), **37** was obtained as a white solid (1.6 g, 84% yield). R_f = 0.37 (petroleum ether/ethyl acetate = 4:1). ^{1}H NMR (400 MHz, $CDCl_3$) δ 9.55 (s, 1H), 7.83 (d, *J* = 8.7 Hz, 2H), 7.66 (d, *J* = 8.7 Hz, 2H), 2.88 (d, *J* = 14.0 Hz, 1H), 2.73 (d, *J* = 14.0 Hz, 1H), 2.07 – 1.98 (m, 1H), 1.97 – 1.88 (m, 1H), 1.65 (s, 3H), 1.47 (s, 9H), 1.43 – 1.35 (m, 2H), 0.96 (t, *J* = 7.3 Hz, 3H). ^{13}C NMR (101 MHz, $CDCl_3$) δ 159.14, 154.97, 140.43, 133.45, 119.71, 118.51, 108.43, 91.27, 39.10, 37.66, 30.64, 27.77, 22.99, 22.56, 21.37, 8.06. HRMS: *Chemical formula* ([M]): $C_{20}H_{26}N_2O_5$, calcd. 375.1915 $[M+H]^+$, Found: 375.1908.

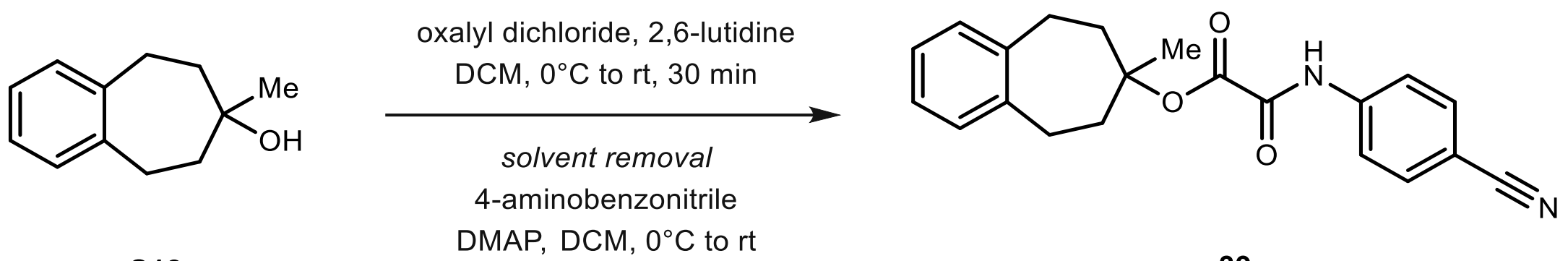

S18 39

39 was prepared using the *General procedure* from 0.46 g (2.6 mmol) of **S18**. After column chromatography purification (eluent: petroleum ether/ethyl acetate = 4:1 v/v, with 0.1% triethylamine), **39** was obtained as a white solid (0.78 g, 86% yield). R_f = 0.42 (petroleum ether/ethyl acetate = 4:1). ^{1}H NMR (400 MHz, $CDCl_3$) δ 9.10 (s, 1H), 7.79 (d, *J* = 8.8 Hz, 2H), 7.67 (d, *J* = 8.7 Hz, 2H), 7.12 (s, 4H), 3.17 – 3.07 (m, 2H), 2.67 – 2.56 (m, 4H), 1.75 – 1.66 (m, 2H), 1.65 (s, 3H). ^{13}C NMR (101 MHz, $CDCl_3$) δ 159.27, 154.88, 141.85, 140.37, 133.49, 128.91, 126.50, 119.76, 118.49, 108.55, 89.96, 37.93, 29.50, 26.65. HRMS: *Chemical formula* ([M]): $C_{21}H_{20}N_2O_3$, calcd. 349.1547 $[M+H]^+$, Found: 349.1545.

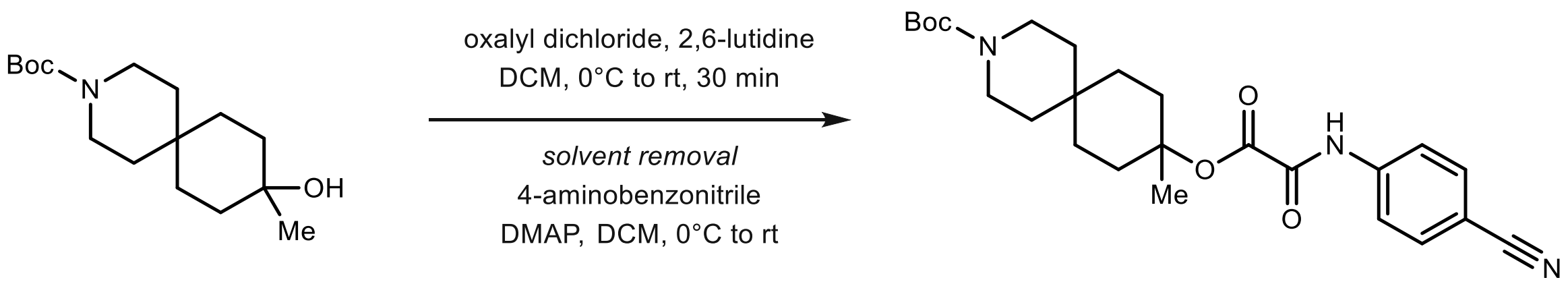

S19 41

41 was prepared using the *General procedure* from 0.68 g (2.4 mmol) of **S19**. After column chromatography purification (eluent: petroleum ether/ethyl acetate = 2:1 v/v, with 0.1% triethylamine), **41** was obtained as a white solid (0.93 g, 85% yield). R_f = 0.37 (petroleum ether/ethyl acetate = 2:1). ^{1}H NMR (400 MHz, $CDCl_3$) δ 9.09 (s, 1H), 7.77 (d, *J* = 8.7 Hz, 2H), 7.66 (d, *J* = 8.7 Hz, 2H), 3.39 – 3.32 (m, 4H), 2.29 – 2.18 (m, 2H), 1.69 – 1.61 (m, 2H), 1.59 (s, 3H), 1.51 – 1.45 (m, 6H), 1.44 (s, 9H), 1.37 (t, *J* = 5.9 Hz, 2H). ^{13}C NMR (101 MHz, $CDCl_3$) δ 159.28, 154.99, 154.91, 140.39, 133.45, 119.74, 118.48, 108.51, 87.30, 79.36, 31.29, 31.27, 30.28, 28.47, 24.68. HRMS: *Chemical formula* ([M]): $C_{25}H_{33}N_3O_5$, calcd. 456.2493 $[M+H]^+$, Found: 456.2492.

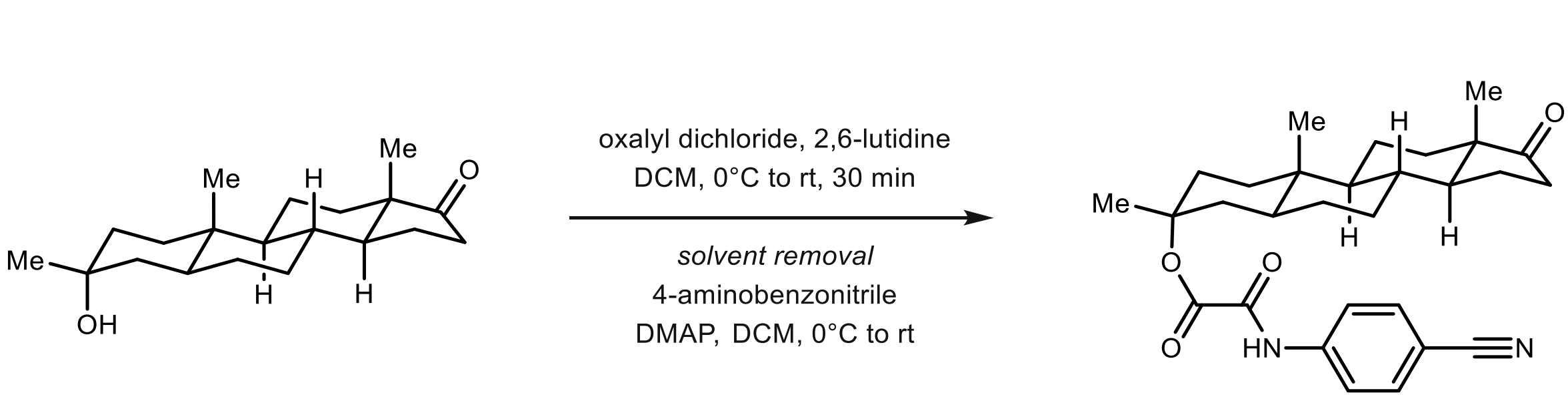

S20 49

49 was prepared using the *General procedure* from 0.24 g (0.80 mmol) of **S20**. After column chromatography purification (eluent: petroleum ether/ethyl acetate = 3:1 v/v, with 0.1% triethylamine), **49** was obtained as a white solid (0.30 g, 79% yield). R_f = 0.42 (petroleum ether/ethyl acetate = 3:1). ^{1}H NMR (500 MHz, $CDCl_3$) δ 9.07 (s, 1H),

7.77 (d, J = 8.8 Hz, 2H), 7.66 (d, J = 8.7 Hz, 2H), 2.42 (dd, J = 19.3, 8.7 Hz, 1H), 2.35 (dt, J = 14.8, 4.4 Hz, 1H), 2.11 – 2.06 (m, 1H), 1.96 – 1.87 (m, 1H), 1.82 – 1.74 (m, 2H), 1.67 – 1.60 (m, 1H), 1.60 – 1.58 (m, 1H), 1.58 (s, 3H), 1.57 – 1.52 (m, 2H), 1.52 – 1.43 (m, 2H), 1.42 – 1.36 (m, 1H), 1.36 – 1.15 (m, 7H), 1.01 (qd, J = 12.5, 4.4 Hz, 1H), 0.85 (s, 3H), 0.83 (s, 3H), 0.82 – 0.77 (m, 1H). ^{13}C NMR (126 MHz, $CDCl_3$) δ 159.47, 155.11, 140.50, 133.60, 119.82, 118.59, 108.66, 87.72, 54.05, 51.47, 47.92, 41.07, 38.82, 35.96, 35.60, 35.13, 33.93, 32.09, 31.59, 30.69, 27.84, 25.96, 21.88, 20.38, 13.95, 11.80. HRMS: *Chemical formula* ([M]): $C_{29}H_{36}N_2O_4$, calcd. 494.3013 $[M+NH_4]^+$, Found: 494.3005.

5. Substrate scope

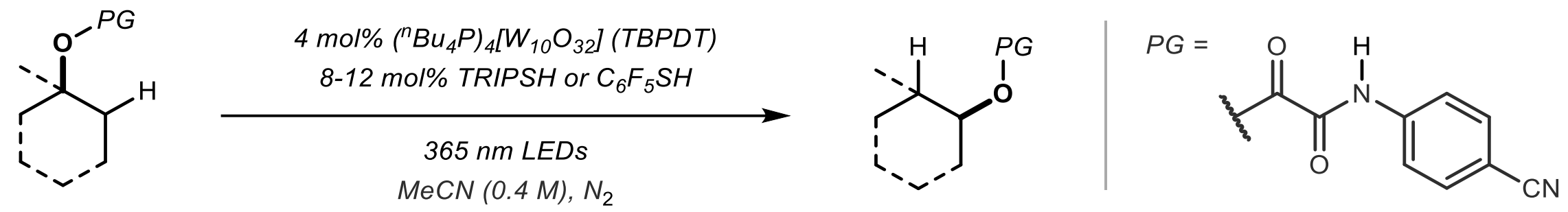

General Procedure A: An oven-dried 4 mL vial was charged with the oxamoyl-protected alcohol substrate (0.1 mmol, 1.0 equiv) and tetrabutylphosphonium decatungstate (TBPDT, 13.6 mg, 0.004 mmol, 0.04 equiv). The vial was transferred to a nitrogen-filled glovebox, where MeCN (0.23 mL) and a stock solution of 2,4,6-triisopropylbenzenethiol (**T1**) (0.4 M in MeCN, 20 µL, 0.08 equiv) were added. After sealing with a PTFE-lined screw cap, the vial was removed from the glovebox and placed in a photoreactor (365 nm LED). The reaction mixture was stirred at the indicated temperature for the specified duration. For each substrate, two parallel runs were conducted and combined upon completion. The resulting mixture was concentrated under reduced pressure, and the residue was purified by flash column chromatography on silica gel to afford the desired product.

General Procedure B: An oven-dried 4 mL vial was charged with the oxamoyl-protected alcohol substrate (0.1 mmol, 1.0 equiv) and tetrabutylphosphonium decatungstate (TBPDT, 13.6 mg, 0.004 mmol, 0.04 equiv). The vial was transferred to a nitrogen-filled glovebox, where MeCN (0.225 mL) and a stock solution of pentafluorobenzenethiol (**T4**) (0.4 M in MeCN, 25 µL, 0.1 equiv) were added. After sealing with a PTFE-lined screw cap, the vial was removed from the glovebox and placed in a photoreactor (365 nm LED). The reaction mixture was stirred at the indicated temperature for the specified duration. For each substrate, two parallel runs were conducted and combined upon completion. The resulting mixture was concentrated under reduced pressure, and the residue was purified by flash column chromatography on silica gel to afford the desired product.

Three types of photoreactors were employed in this study. *Photoreactors A and B* were detailed in the prior section. *Photoreactor C* is as follows.

Photoreactor C:

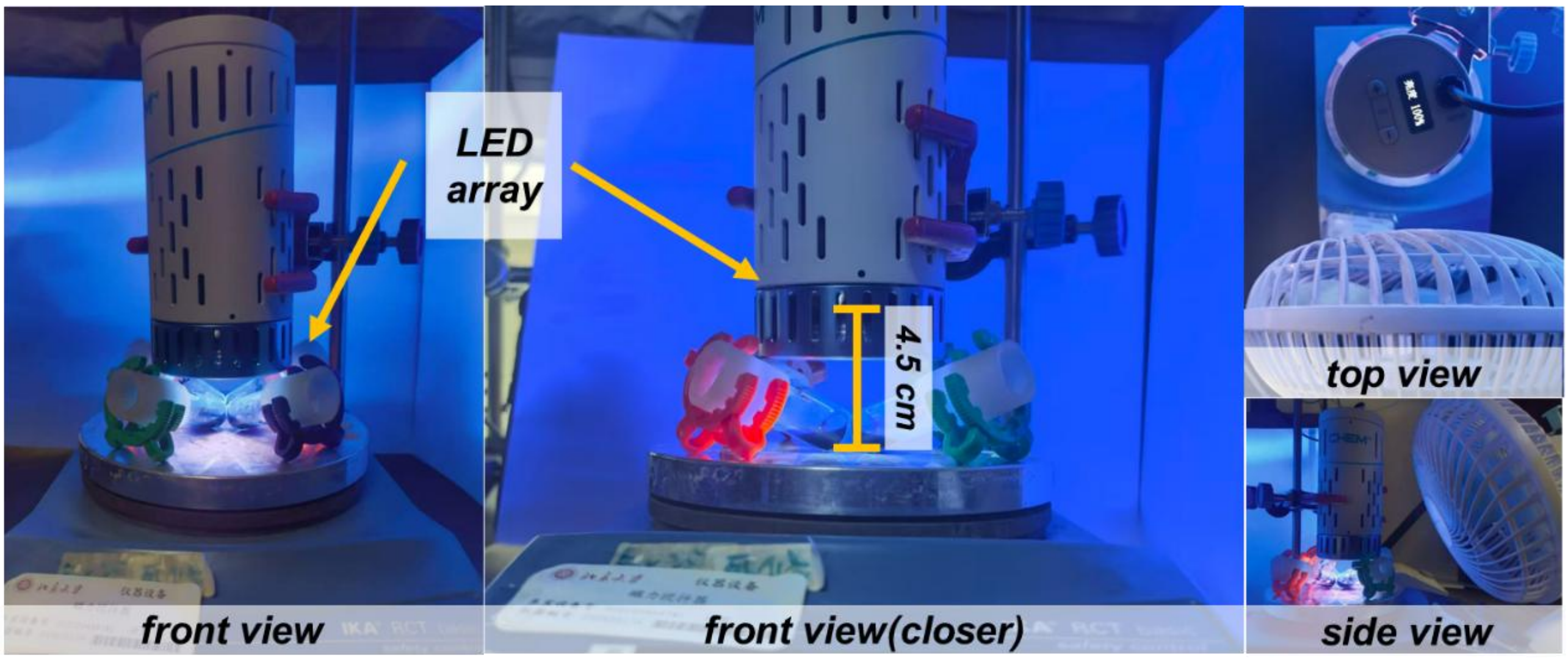

Figure S9 | Photoreactor C

The *Photoreactor C* is equipped with a 40 W LED array (purchased from Chem^{N} Co., Ltd) with a peak emission wavelength of 369.7 nm (*Figure S9*). The emission spectrum is provided in *Figure S10*. The LED array was positioned 4.5 cm above the solution surface in the reaction vessels (4 mL, fabricated from standard borosilicate glass). At this distance, the measured irradiance was approximately 230–235 mW/cm^2 at the peak wavelength. A cooling fan was placed adjacent to the reaction to maintain the internal temperature between 35–40 °C, as monitored by a handheld IR thermometer.

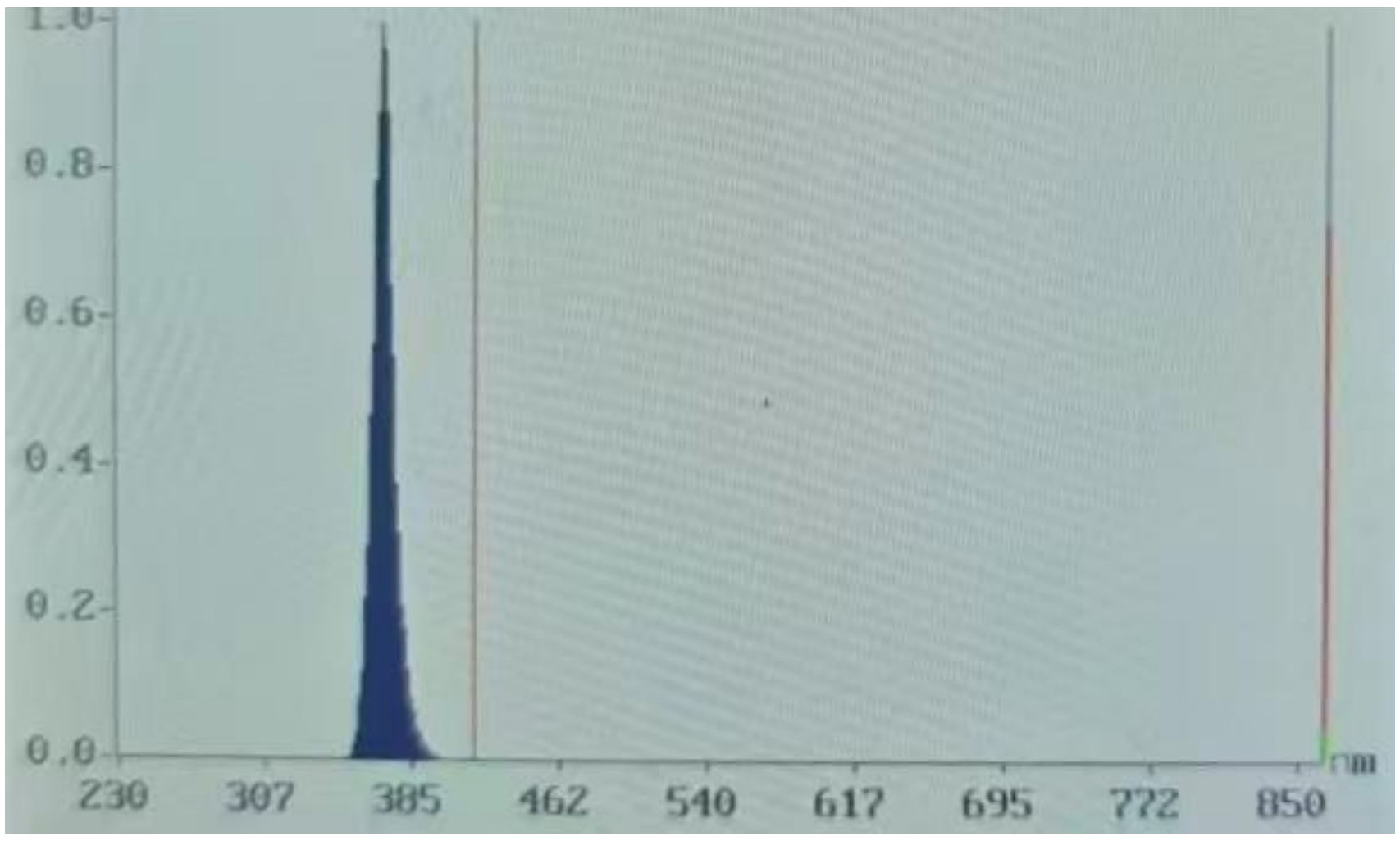

Figure S10 | The emission spectrum of the LED in *Photoreactor C* (*monitored by WATTCAS-PCS-230850*).

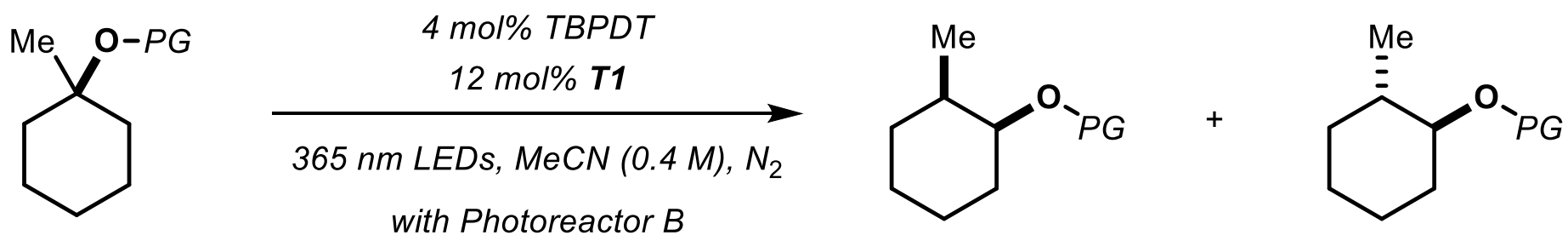

2m: Following modified *General Procedure A* using 12 mol% of **T1**, two parallel reactions (0.1 mmol scale each) of compound **1m** (57.2 mg total) were carried out in *Photoreactor B* for 12 h, affording **2m** as a pair of diastereomers. **2ma** was isolated as a pure compound (white solid, 36.6 mg, 64% yield). R_f = 0.35 (petroleum ether/MeCN/ethyl acetate = 10:1:2). **2mb** was isolated as a pure compound (white solid, 13.7 mg, 24% yield). R_f = 0.30 (petroleum ether/MeCN/ethyl acetate = 10:1:2). The regioselectivity of the acyloxy translocation reaction was determined to be >20:1 in the crude reaction mixture by ^{1}H NMR and >20:1 after isolation. The diastereomeric ratio of **2m** was determined to be 3:1 in the crude reaction mixture by ^{1}H NMR and 2.7:1 after isolation (**2ma**: **2mb**).

Characterization data for **2ma**: ^{1}H NMR (600 MHz, $CDCl_3$) δ 9.12 (s, 1H), 7.80 (d, *J* = 8.4 Hz, 2H), 7.66 (d, *J* = 7.6 Hz, 2H), 5.12 (d, *J* = 2.8 Hz, 1H), 1.94 (dt, *J* = 11.1, 5.4 Hz, 1H), 1.89 – 1.77 (m, 1H), 1.70 (dt, *J* = 13.3, 4.1 Hz, 1H), 1.62 – 1.46 (m, 5H), 1.38 – 1.30 (m, 1H), 0.94 (d, *J* = 6.8 Hz, 3H). ^{13}C NMR (151 MHz, $CDCl_3$) δ 160.14, 154.44, 140.42, 133.45, 119.82, 118.49, 108.53, 78.97, 34.60, 29.42, 29.19, 24.19, 20.87, 17.11. HRMS: *Chemical formula* ([M]): $C_{16}H_{18}N_2O_3$, calcd. 287.1390 [M+H]$^+$, Found: 287.1394.

Characterization data for **2mb**: ^{1}H NMR (600 MHz, $CDCl_3$) δ 9.07 (s, 1H), 7.79 (d, *J* = 8.3 Hz, 2H), 7.68 (d, *J* = 8.4

Hz, 2H), 4.62 (td, J = 10.4, 4.2 Hz, 1H), 2.03 (d, J = 12.3 Hz, 1H), 1.88 – 1.75 (m, 3H), 1.68 (d, J = 12.3 Hz, 1H), 1.54 – 1.46 (m, 1H), 1.42 – 1.33 (m, 1H), 1.27 (q, J = 12.4 Hz, 1H), 1.16 – 1.07 (m, 1H), 0.95 (d, J = 6.4 Hz, 3H). ^{13}C NMR (151 MHz, $CDCl_3$) δ 160.17, 154.37, 140.27, 133.48, 119.82, 118.46, 108.65, 83.43, 36.95, 33.40, 31.27, 25.06, 24.68, 18.45. HRMS: *Chemical formula* ([M]): $C_{16}H_{18}N_2O_3$, calcd. 309.1209 $[M+Na]^+$, Found: 309.1200.

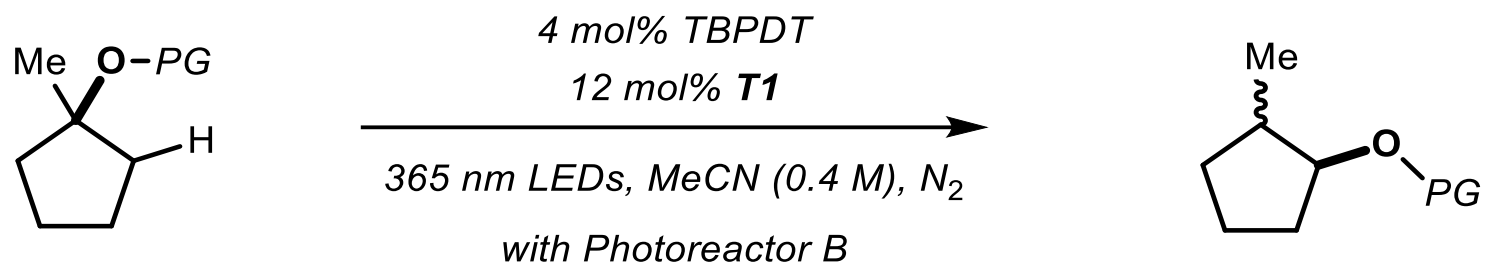

3 **4**

(as a pair of diastereomers)

4: Following modified *General Procedure A* using 12 mol% of **T1**, two parallel reactions (0.1 mmol scale each) of compound **3** (54.4 mg total) were carried out in *Photoreactor B* for 48 h, affording **4** as a pair of diastereomers (white solid, 38.1 mg, 70% yield). R_f = 0.5 (petroleum ether/ethyl acetate/dichloromethane = 5:1:2). The regioselectivity of the acyloxy translocation reaction was determined to be >20:1 in the crude reaction mixture by ^{1}H NMR and >20:1 after isolation. The diastereomeric ratio of **4** was determined to be 3.4:1 in the crude reaction mixture by ^{1}H NMR and 3.5:1 after isolation by ^{1}H NMR.

Characterization data for **4** *(as a pair of diastereomers)***:** ^{1}H NMR (400 MHz, $CDCl_3$) δ 9.11 (s, 1H), 7.83 – 7.75 (m, 2H), 7.72 – 7.63 (m, 2H), 5.46 – 5.23 (m, 0.78H), 4.93 – 4.85 (m, 0.22H), 2.25 – 2.01 (m, 2H), 2.00 – 1.74 (m, 3H), 1.74 – 1.58 (m, 1H), 1.57 – 1.47 (m, 0.78H), 1.30 – 1.25 (m, 0.22H), 1.08 – 1.00 (m, 3H). ^{13}C NMR (101 MHz, $CDCl_3$) δ 160.57, 160.39, 154.50, 154.47, 140.46, 140.41, 133.57 (*overlapped*), 119.96, 119.92, 118.60 (*overlapped*), 108.70, 108.66, 87.08, 83.71, 40.05, 39.09, 32.22, 32.02, 31.48, 31.35, 22.61, 22.36, 18.30, 13.88. HRMS: *Chemical formula* ([M]): $C_{15}H_{16}N_2O_3$, calcd. 273.1169 $[M+H]^+$, Found: 273.1239.

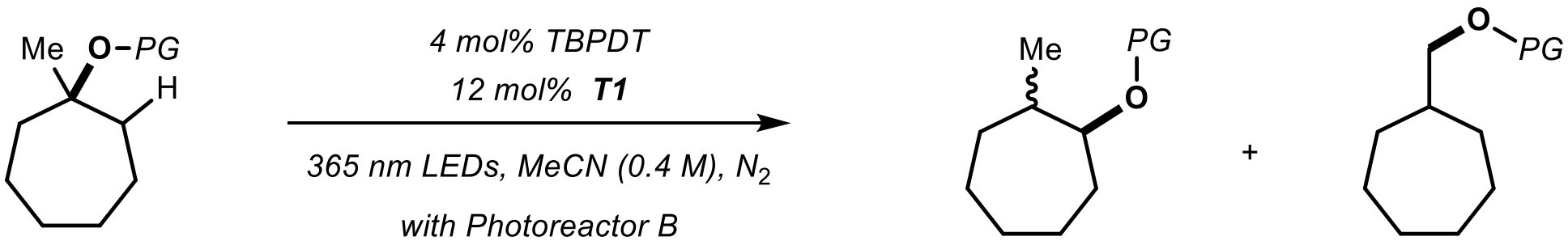

5 **6** **6'**

(as a pair of diastereomers)

6: Following modified *General Procedure A* using 12 mol% of **T1**, two parallel reactions (0.1 mmol scale each) of compound **5** (60.0 mg total) were carried out in *Photoreactor B* for 48 h, affording **6** as a pair of diastereomers together with a very minor amount of the regioisomer **6′**. A portion of **6-*diastereomer 1*** was isolated as pure compound (white solid, 20.5 mg, 34% yield). R_f = 0.36 (petroleum ether/ethyl acetate/acetonitrile = 10:2:1). A portion of **6-*diastereomer 2*** was isolated as a mixture with **6′** (white solid 16.3 mg, 27% yield, **6-*diastereomer 2*:6′**=0.94:0.06). R_f = 0.35 (petroleum ether/ethyl acetate/acetonitrile = 10:2:1). The remaining product was collected as a mixture of diastereomers of **6** (11.0 mg, 18% yield). This provided a combined total yield of 79%. The regioselectivity of the acyloxy translocation reaction was determined to be >20:1 in the crude reaction mixture by ^{1}H

NMR and >20:1 after isolation (**6**: **6′**). The diastereomeric ratio of **6** was determined to be 1.8:1 in the crude reaction mixture by ^{1}H NMR analysis and 1.6:1 after isolation.

Characterization data for **6-*diastereomer 1***: ^{1}H NMR (400 MHz, $CDCl_3$) δ 9.10 (s, 1H), 7.83 – 7.74 (m, 2H), 7.70 – 7.61 (m, 2H), 5.17 (dt, *J* = 7.5, 3.7 Hz, 1H), 2.02 (ddq, *J* = 13.6, 6.9, 3.6 Hz, 1H), 1.97 – 1.81 (m, 2H), 1.78 – 1.61 (m, 4H), 1.60 – 1.42 (m, 4H), 0.99 (d, *J* = 7.0 Hz, 3H). ^{13}C NMR (101 MHz, $CDCl_3$) δ 160.15, 154.54, 140.50, 133.56, 119.91, 118.59, 108.66, 82.55, 37.33, 31.18, 30.81, 28.04, 25.93, 22.62, 18.17. HRMS: *Chemical formula* ([M]): $C_{17}H_{20}N_2O_3$, calcd. 312.1812 $[M+NH_4]^+$, Found: 312.1815.

Characterization data for **6-*diastereomer 2***: ^{1}H NMR (400 MHz, $CDCl_3$) δ 9.10 (s, 1H), 7.82 – 7.76 (m, 2H), 7.69 – 7.64 (m, 2H), 4.76 (dt, *J* = 9.0, 5.7 Hz, 1H), 2.05 – 1.94 (m, 1H), 1.91 – 1.81 (m, 2H), 1.78 – 1.61 (m, 4H), 1.58 – 1.46 (m, 3H), 1.37 – 1.27 (m, 1H), 0.97 (d, *J* = 6.7 Hz, 3H). ^{13}C NMR (101 MHz, $CDCl_3$) δ 160.18, 154.61, 140.44, 133.58, 119.95, 118.59, 108.72, 85.97, 38.89, 32.72, 32.11, 28.17, 26.05, 22.48, 20.62. HRMS: *Chemical formula* ([M]): $C_{17}H_{20}N_2O_3$, calcd. 312.1366 $[M+Na]^+$, Found: 323.1368.

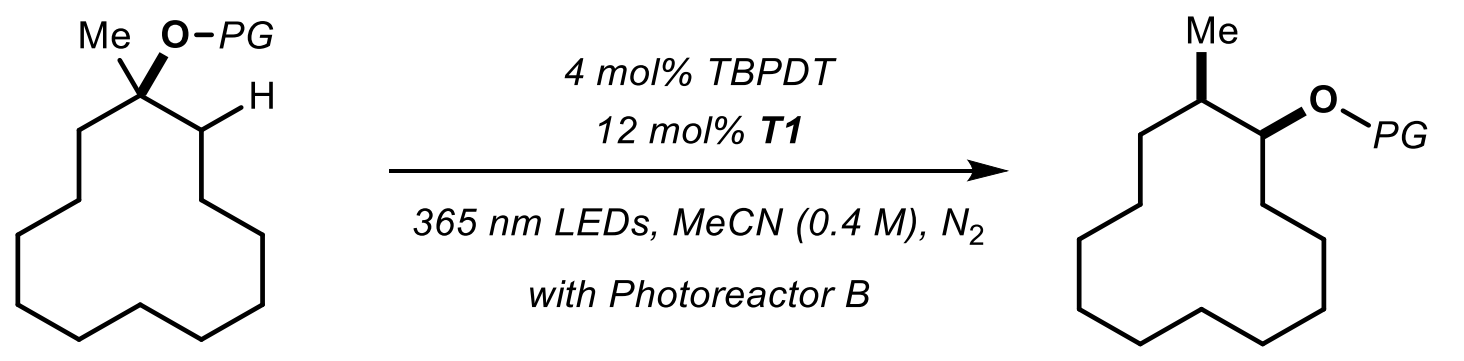

7 **8**

8: Following modified *General Procedure A* using 12 mol% of **T1**, two parallel reactions (0.1 mmol scale each) of compound **7** (74.0 mg total) were carried out in *Photoreactor B* for 48 h. **8** was isolated as a pure compound (white solid, 63.8 mg, 86% yield). R_f = 0.5 (petroleum ether/ethyl acetate = 6:1). The regioselectivity of the acyloxy translocation reaction was determined to be >20:1 in the crude reaction mixture by ^{1}H NMR analysis and >20:1 after isolation. The diastereomeric ratio of **8** was determined to be > 20:1 in the crude reaction mixture by ^{1}H NMR and > 20:1 after isolation.

Characterization data for **8:** ^{1}H NMR (400 MHz, $CDCl_3$) δ 9.05 (s, 1H), 7.79 – 7.75 (m, 2H), 7.71 – 7.63 (m, 2H), 5.27 – 5.19 (m, 1H), 1.94 – 1.79 (m, 2H), 1.68 – 1.58 (m, 1H), 1.56 – 1.48 (m, 1H), 1.49 – 1.39 (m, 6H), 1.40 – 1.23 (m, 11H), 0.97 (d, *J* = 6.8 Hz, 3H). ^{13}C NMR (101 MHz, $CDCl_3$) δ 160.47, 154.58, 140.51, 133.52, 119.93, 118.60, 108.58, 80.68, 33.98, 28.46, 25.05, 24.91, 24.65, 23.81, 22.93, 22.03, 21.98, 21.78, 16.29 (One sp^3 carbon was not detected, possibly due to overlap). HRMS: *Chemical formula* ([M]): $C_{22}H_{30}N_2O_3$, calcd. 388.2594 $[M+NH_4]^+$, Found: 388.2696.

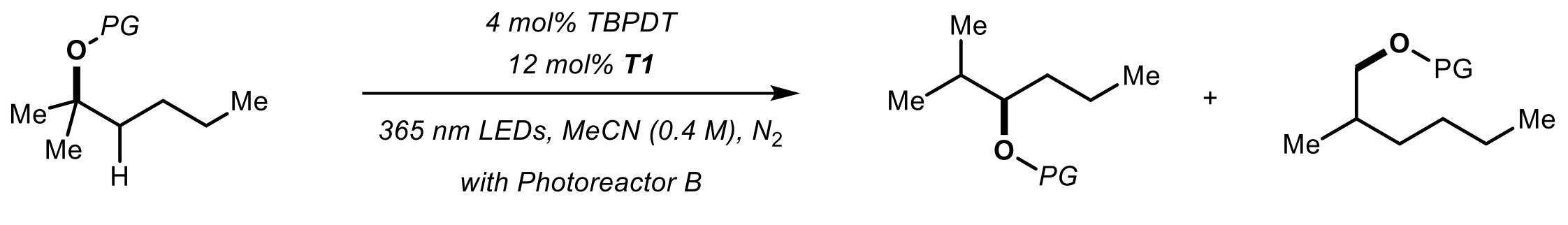

9 **10a** **10b**

10: Following modified *General Procedure A* using 12 mol% of **T1**, two parallel reactions of compound **9** (57.6 mg in total) were carried out in *Photoreactor B* for 48 h. Upon isolation, a portion of pure **10a** was obtained as a colorless oil (7 mg, 12% yield); R_f = 0.40 (petroleum ether/ethyl acetate = 6:1). Compound **10b** was isolated as a colorless oil (4.1 mg, 7% yield); R_f = 0.35 (petroleum ether/ethyl acetate = 6:1). The remainder of **10a** was obtained as a mixture with **9** (39.5 mg total, **10a**:**9** = 0.48:0.52), corresponding to 18.8 mg of **10a** (33% yield). This provides a combined total yield of compound **10** of 52%. The regioselectivity of the acyloxy translocation reaction was determined to be 8.5:1 in the crude reaction mixture by ^{1}H NMR and 6.4:1 after isolation (**10a**:**10b**).

Characterization data for **10a**: ^{1}H NMR (600 MHz, $CDCl_3$) δ 9.06 (s, 1H), 7.78 (d, *J* = 8.3 Hz, 2H), 7.68 (d, *J* = 8.4 Hz, 2H), 4.94 (ddd, *J* = 9.3, 5.5, 3.6 Hz, 1H), 2.03 – 1.93 (m, *J* = 6.7 Hz, 1H), 1.77 – 1.67 (m, 1H), 1.66 – 1.58 (m, 1H), 1.45 – 1.34 (m, 1H), 1.34 – 1.27 (m, 1H), 0.97 (dd, *J* = 6.7, 1.4 Hz, 6H), 0.93 (t, *J* = 7.3 Hz, 3H). ^{13}C NMR (151 MHz, $CDCl_3$) δ 160.53, 154.44, 140.43, 133.62, 119.91, 118.59, 108.76, 83.92, 33.19, 31.69, 18.95, 18.65, 17.87, 13.98. HRMS: *Chemical formula* ([M]): $C_{16}H_{20}N_2O_3$, calcd. 306.1812 $[M+NH_4]^+$, Found: 306.1821.

Characterization data for **10b**: ^{1}H NMR (400 MHz, $CDCl_3$) δ 9.02 (s, 1H), 7.81 – 7.74 (m, 2H), 7.71 – 7.65 (m, 2H), 4.25 (dd, *J* = 10.6, 5.9 Hz, 1H), 4.15 (dd, *J* = 10.7, 7.1 Hz, 1H), 2.02 – 1.92 (m, 1H), 1.50 – 1.40 (m, 1H), 1.40 – 1.16 (m, 5H), 1.01 (d, *J* = 6.7 Hz, 3H), 0.91 (t, *J* = 7.0 Hz, 3H). ^{13}C NMR (101 MHz, $CDCl_3$) δ 160.73, 154.18, 140.32, 133.64, 119.96, 118.56, 108.88, 73.05, 32.96, 32.60, 29.07, 22.94, 16.88, 14.17.HRMS: *Chemical formula* ([M]): $C_{16}H_{20}N_2O_3$, calcd. 311.1366 $[M+Na]^+$, Found: 311.1356.

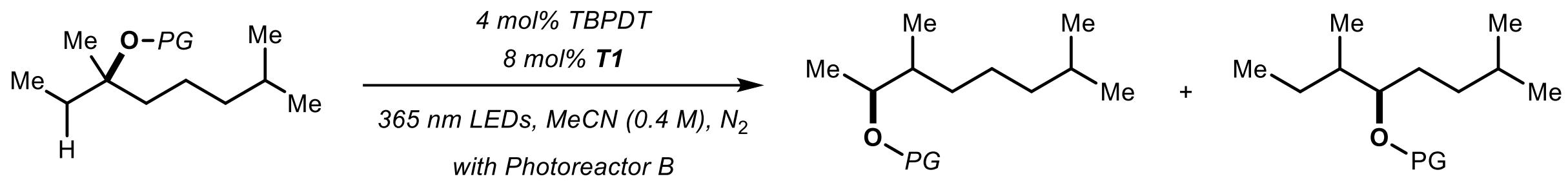

11 **12a** *(as a pair of diastereomers)* **12b** *(as a pair of diastereomers)*

12: Following *General Procedure A*, two parallel reactions (0.1 mmol scale each) of compound **11** (57.6 mg in total) was carried out in *Photoreactor B* for 48 h. The major regioismer **12a** was isolated as a pair of inseparable diastereomers (white solid, 40.5 mg, 61% yield) (*The diastereomers were indistinguishable by ^{1}H NMR spectroscopy, rendering the accurate determination of the diastereomeric ratio for **12a** unfeasible. However, based on the ^{13}C NMR spectrum, the diastereomeric ratio was estimated to be around 1:1).* The minor regioismer **12b** was isolated as a pair of inseparable diastereomers (white solid, 8.1 mg, *d.r.* = 1.2:1, 12% yield). The regioselectivity of the acyloxy translocation reaction can't be determined in the crude reaction mixture by ^{1}H NMR due to peak overlapping and was found to be 5:1 after isolation (**12a**:**12b**).

Characterization data for **12a**: ^{1}H NMR (400 MHz, $CDCl_3$) δ 9.19 – 9.06 (m, 1H), 7.82 – 7.77 (m, 2H), 7.68 – 7.63 (m, 2H), 5.05 – 4.95 (m, 1H), 1.88 – 1.68 (m, 1H), 1.57 – 1.45 (m, 1H), 1.45 – 1.11 (m, 9H), 0.99 – 0.92 (m, 3H), 0.87 – 0.82 (m, 6H). ^{13}C NMR (101 MHz, $CDCl_3$) δ 160.15, 160.09, 154.60, 140.52, 133.48, 119.98, 118.57, 108.57, 79.43, 79.32, 39.11, 37.68, 37.21, 32.72, 32.52, 27.95, 24.76, 22.74, 22.58, 16.89, 15.75, 14.81, 14.56 (*Due to the overlap of carbon resonances between the two diastereomers of **12a**, fewer signals were observed in the ^{13}C NMR spectrum than theoretically expected*). HRMS: *Chemical formula* ([M]): $C_{19}H_{26}N_2O_3$, calcd. 348.2281 $[M+NH_4]^+$, Found: 348.2278.

Characterization data for **12b**: ^{1}H NMR (400 MHz, $CDCl_3$) δ 9.07 (m, 1H), 7.83 – 7.75 (m, 2H), 7.71 – 7.63 (m, 2H), 5.04 (dt, J = 8.6, 4.5 Hz, 0.54H), 4.98 (ddd, J = 8.9, 5.7, 3.9 Hz, 0.46H), 1.80 – 1.63 (m, 3H), 1.56 – 1.40 (m, 2H), 1.27 – 1.13 (m, 3H), 0.99 – 0.90 (m, 6H), 0.88 (m, 6H). ^{13}C NMR (101 MHz, $CDCl_3$) δ160.48, 154.44, 154.43, 140.44, 133.61, 119.89, 118.60, 108.74, 83.64, 82.94, 38.23, 38.04, 34.83, 34.72, 29.23, 28.31, 28.07, 25.82, 24.95, 22.77, 22.65, 22.59, 22.49, 14.80, 14.06, 11.77, 11.55 (*Due to the overlap of carbon resonances between the two diastereomers of **12b**, fewer signals were observed in the ^{13}C NMR spectrum than theoretically expected*). HRMS: *Chemical formula* ([M]): $C_{19}H_{26}N_2O_3$, calcd. 331.2016 $[M+H]^+$, Found: 331.2015.

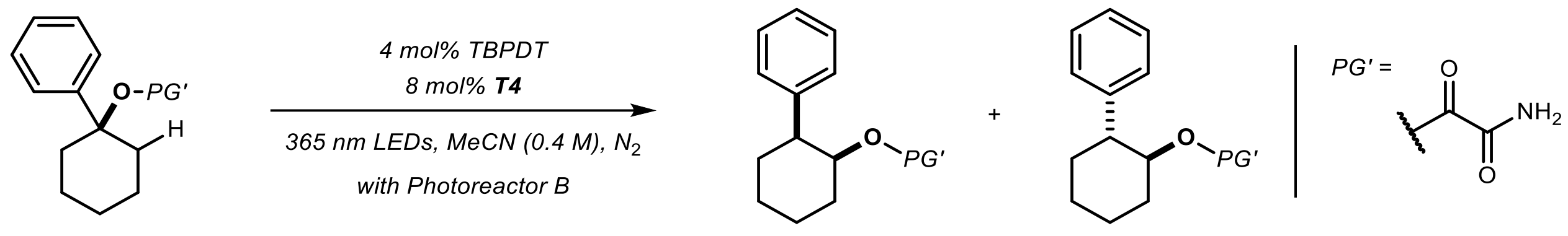

14: Following modified *General Procedure B* using 8 mol% of **T4**, two parallel reactions (0.1 mmol scale each) of compound **13** (49.2 mg total) were carried out in *Photoreactor B* for 48 h. affording **14** as a pair of diastereomers (19.6 mg, 39% yield). The minor diastereomer **14a** was isolated in pure form as a colorless oil (7.6 mg, 15% yield). R_f = 0.56 (petroleum ether/ethyl acetate = 1:1). The major diastereomer **14b** was isolated in nearly pure form as a white solid (12.0 mg, 24% yield). R_f = 0.42 (petroleum ether/ethyl acetate = 1:1). The regioselectivity of the acyloxy translocation reaction was determined to be >20:1 in the crude reaction mixture by ^{1}H NMR and >20:1 after isolation. The diastereomeric ratio of **14** was determined to be 1.7:1 in the crude reaction mixture by ^{1}H NMR and 1.6:1 after isolation (**14b**:**14a**).

Characterization data for **14a**: ^{1}H NMR (400 MHz, $CDCl_3$) δ 7.30 – 7.24 (m, 4H), 7.23 – 7.16 (m, 1H), 6.66 (s, 1H), 5.74 (s, 1H), 5.28 (d, J = 3.0 Hz, 1H), 2.86 (dt, J = 13.0, 3.1 Hz, 1H), 2.20 (qd, J = 13.0, 3.6 Hz, 1H), 2.14 – 2.07 (m, 1H), 1.95 (dt, J = 12.9, 4.2 Hz, 1H), 1.83 – 1.62 (m, 4H), 1.47 (qt, J = 11.9, 3.8 Hz, 1H). ^{13}C NMR (101 MHz, $CDCl_3$) δ 159.33, 158.36, 142.67, 128.52, 127.99, 126.87, 77.08, 46.97, 30.62, 26.00, 25.87, 20.10. HRMS: *Chemical formula* ([M]): $C_{14}H_{17}NO_3$, calcd. 270.1101 $[M+Na]^+$, Found: 270.1107.

Characterization data for **14b**: ^{1}H NMR (400 MHz, $CDCl_3$) δ 7.29 – 7.23 (m, 2H), 7.23 – 7.15 (m, 3H), 6.55 (s, 1H), 5.97 (s, 1H), 5.09 (td, J = 10.8, 4.5 Hz, 1H), 2.81 (ddd, J = 12.3, 10.7, 3.8 Hz, 1H), 2.15 (dq, J = 11.9, 4.3 Hz, 1H), 1.98 (dq, J = 13.0, 3.1 Hz, 1H), 1.92 (dq, J = 12.5, 2.9 Hz, 1H), 1.82 (dt, J = 12.3, 2.8 Hz, 1H), 1.69 – 1.56 (m, 2H),

1.50 (qt, J = 13.0, 3.0 Hz, 1H), 1.39 (qt, J = 12.9, 3.4 Hz, 1H). ^{13}C NMR (101 MHz, $CDCl_3$) δ 159.05, 158.48, 142.24, 128.60, 127.63, 126.93, 79.94, 49.51, 33.40, 31.97, 25.68, 24.82. HRMS: *Chemical formula* ([M]): $C_{14}H_{17}NO_3$, calcd. 270.1101 $[M+Na]^+$, Found: 270.1105.

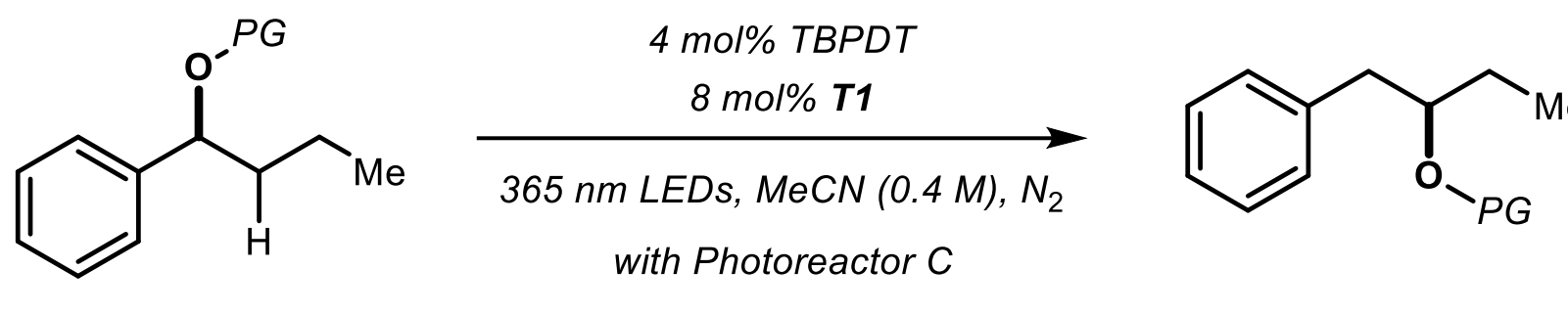

15 **16**

16: Following *General Procedure A*, two parallel reactions (0.1 mmol scale each) of compound **15** (64.4 mg total) were carried out in *Photoreactor C* for 48 h. A portion of **16** was isolated in pure form as a white solid (26.2 mg, 41% yield). R_f = 0.50 (petroleum ether/ethyl acetate/dichloromethane = 5:1:2); the remainder of **16** was obtained as a mixture with **15** (9.5 mg total, **16**:**15** = 0.77:0.23), corresponding to 7.3 mg of **16** (11% yield). This provided a combined total yield of 52%. The regioselectivity of the acyloxy translocation reaction was determined to be >20:1 in the crude reaction mixture by ^{1}H NMR and >20:1 after isolation.

Characterization data for **16**: ^{1}H NMR (400 MHz, $CDCl_3$) δ 8.91 (s, 1H), 7.75 (d, J = 8.7 Hz, 2H), 7.66 (d, J = 8.7 Hz, 2H), 7.33 – 7.26 (m, 2H), 7.25 – 7.19 (m, 3H), 5.20 (p, J = 6.1 Hz, 1H), 3.05 – 2.91 (m, 2H), 1.83 – 1.73 (m, 2H), 0.98 (t, J = 7.4 Hz, 3H). ^{13}C NMR (101 MHz, $CDCl_3$) δ 160.09, 154.25, 140.35, 136.88, 133.57, 129.52, 128.72, 126.98, 119.93, 118.57, 108.76, 81.28, 40.22, 26.67, 9.89. HRMS: *Chemical formula* ([M]): $C_{19}H_{18}N_2O_3$, calcd. 340.1655 $[M+NH_4]^+$, Found: 340.1663.

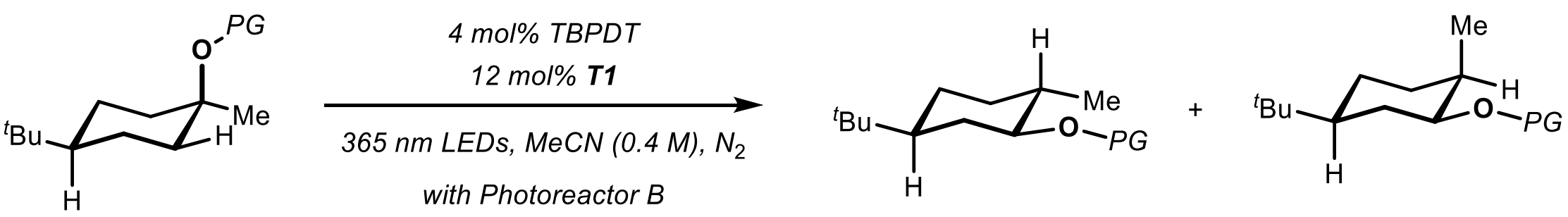

17 **18a** **18b**

18: Following *General Procedure A* using 12 mol% of **T1**, two parallel reactions (0.1 mmol scale each) of compound **17** (68.4 mg total) were carried out in *Photoreactor B* for 12 h. After column chromatography, the diastereomers **18a** and **18b** were isolated in 85% combined yield (57.8 mg). The remaining two diastereomers were formed in a very small amounts and were detectable only by ^{1}H NMR analysis of the crude reaction mixture, but were not isolated.

A portion of **18a** was isolated as a pure compound (white solid, 33.9 mg, 50% yield). R_f = 0.38 (petroleum ether/ethyl acetate = 6:1). The remaining product was isolated as an inseparable mixture of diastereomers (white solid, 23.9 mg, **18a**:**18b** = 0.84:0.16, 35% yield). R_f (**18b**) = 0.36 (petroleum ether/ethyl acetate = 6:1). This provided a combined total isolation yield of 85%. The regioselectivity of the acyloxy translocation reaction was determined to be >20:1 in the crude reaction mixture by ^{1}H NMR and >20:1 after isolation. The diastereomeric ratio of **18** was determined to be in 13:1 the crude reaction mixture by ^{1}H NMR and 14:1 after isolation (**18a**:**18b**).

Characterization data for the major diastereomer **18a**: ^{1}H NMR (400 MHz, $CDCl_3$) δ 9.15 (s, 1H), 7.80 (d, *J* = 8.8 Hz, 2H), 7.67 (d, *J* = 8.7 Hz, 2H), 4.64 (td, *J* = 10.5, 4.4 Hz, 1H), 2.04 (m, 1H), 1.90 – 1.82 (m, 1H), 1.75 – 1.66 (m, 2H), 1.31 – 1.15 (m, 2H), 1.13 – 0.96 (m, 2H), 0.92 (d, *J* = 6.4 Hz, 3H), 0.85 (s, 9H). ^{13}C NMR (101 MHz, $CDCl_3$) δ 160.28, 154.59, 140.45, 133.56, 119.99, 118.58, 108.70, 84.25, 46.83, 37.12, 33.21, 32.84, 32.50, 27.57, 26.35, 18.37. HRMS: *Chemical formula* ([M]): $C_{20}H_{26}N_2O_3$, calcd. 343.2016 $[M+H]^+$, Found: 343.2013.

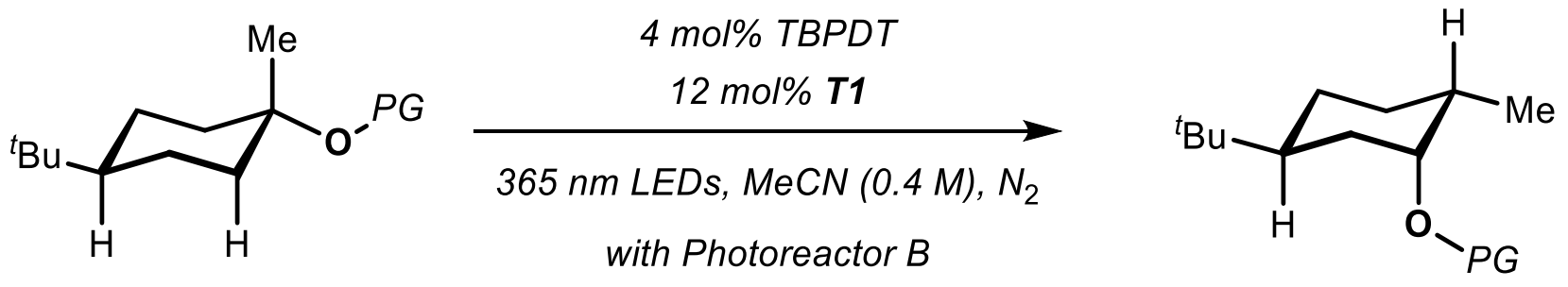

19 **20**

20: Following *General Procedure A* using 12 mol% of **T1**, two parallel reactions (0.1 mmol scale each) of compound **19** (68.4 mg total) were carried out in *Photoreactor B* for 12 h. Among the four possible diastereomers, compound **20** was obtained as the major diastereomer with an excellent diastereomeric ratio of 16:1:trace:trace. After column chromatography, **20** was isolated as a pure compound (white solid, 65.3 mg, 95% yield). R_f = 0.30 (petroleum ether/MeCN/ethyl acetate = 10:1:2). The regioselectivity of the acyloxy translocation reaction was determined to be >20:1 in the crude reaction mixture by ^{1}H NMR and >20:1 after isolation.

Characterization data for **20**: ^{1}H NMR (600 MHz, $CDCl_3$) δ 9.17 (s, 1H), 7.81 (d, *J* = 8.6 Hz, 2H), 7.71 – 7.60 (m, 2H), 5.22 (s, 1H), 2.03 (s, 1H), 1.81 (dt, *J* = 12.8, 3.1 Hz, 1H), 1.69 – 1.62 (m, 1H), 1.59 (dd, *J* = 13.5, 3.6 Hz, 1H), 1.55 – 1.47 (m, 1H), 1.39 (tt, *J* = 12.3, 3.0 Hz, 1H), 1.30 (td, *J* = 13.5, 2.4 Hz, 1H), 1.11 – 1.01 (m, 1H), 0.90 (d, *J* = 6.7 Hz, 3H), 0.81 (s, 9H). ^{13}C NMR (151 MHz, $CDCl_3$) δ 160.23, 154.46, 140.46, 133.43, 119.82, 118.49, 108.50, 79.46, 40.80, 35.36, 31.97, 31.55, 29.09, 27.29, 26.55, 18.02. HRMS: *Chemical formula* ([M]): $C_{20}H_{26}N_2O_3$, calcd. 343.2016 $[M+H]^+$, Found: 343.2023.

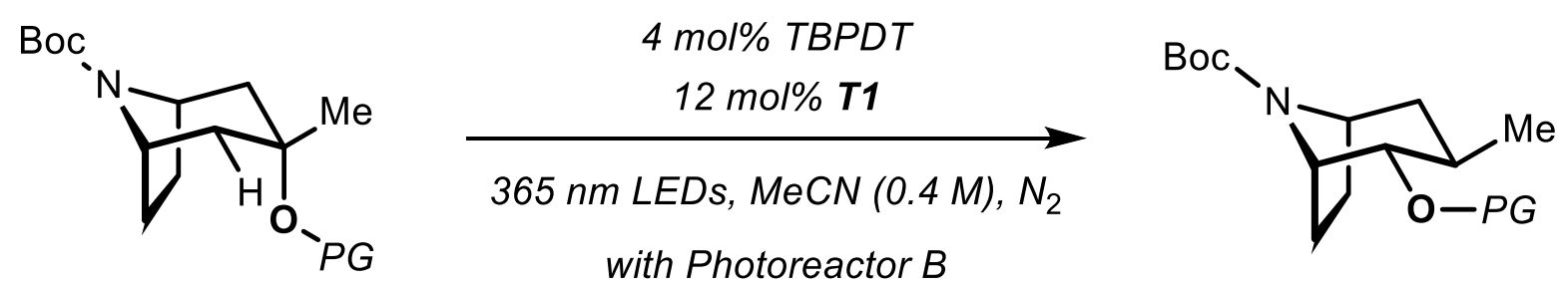

21 **22**

22: Following modified *General Procedure A* using 12 mol% of **T1**, two parallel reactions (0.1 mmol scale each) of compound **21** (82.6 mg total) were carried out in *Photoreactor B* for 48 h, affording **22** as a single diastereomer. A portion of **22** was isolated in pure form as a white solid (25.8 mg, 31% yield). R_f = 0.30 (petroleum ether/ethyl acetate = 3:1). A portion of **22** was isolated in nearly pure form as a white solid (25.9 mg total, mixed with 1.0% of 4-aminobenzonitrile, 31% yield). This provided a combined total isolation yield of 62%. The regioselectivity of the acyloxy translocation reaction was determined to be >20:1 in the crude reaction mixture by ^{1}H NMR and >20:1 after isolation.

Characterization data for **22**: ^{1}H NMR (500 MHz, $CDCl_3$) δ 9.06 (s, 1H), 7.78 (d, *J* = 8.7 Hz, 2H), 7.66 (d, *J* = 8.7 Hz, 2H), 4.73 (dd, *J* = 10.3, 3.7 Hz, 1H), 4.31 – 4.18 (m, 2H), 2.16 – 2.08 (m, 1H), 2.05 – 1.96 (m, 2H), 1.90 – 1.84 (m, 1H), 1.69 – 1.62 (m, 2H), 1.50 (s, 9H), 1.46 (s, 1H), 0.96 (d, *J* = 6.4 Hz, 3H). ^{13}C NMR (126 MHz, $CDCl_3$) δ 160.28, 154.18, 153.46, 140.46, 133.58, 120.11, 118.42, 109.08, 80.84, 80.09, 55.29, 53.56, 38.79, 28.91, 28.58, 28.18, 24.22, 18.25. HRMS: *Chemical formula* ([M]): $C_{22}H_{27}N_3O_5$, calcd. 414.2031 [M+H]$^+$, Found: 414.2024.

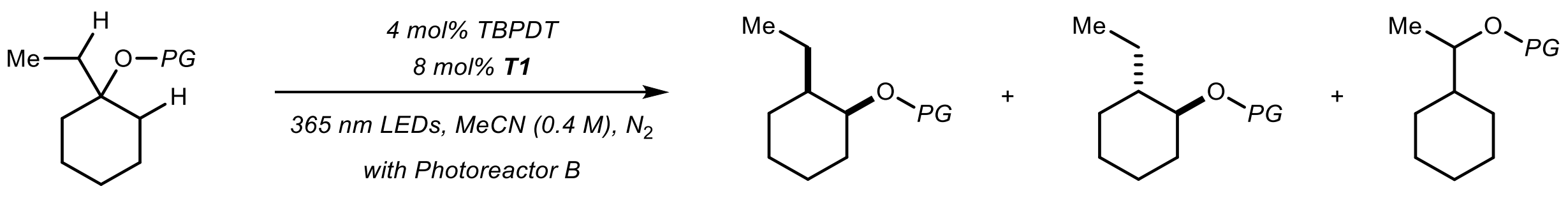

24: Following *General Procedure A*, two parallel reactions (0.1 mmol scale each) of compound **23** (60.0 mg total) were carried out in *Photoreactor B* for 48 h, affording **24** as a pair of diastereomers, together with the regioisomer **24′**. A portion of **24a** was isolated as a pure compound (white solid, 33.9 mg, 56% yield). R_f = 0.36 (petroleum ether/ethyl acetate = 6:1). A portion of **24b** was isolated as a mixture with **24′** (white solid, 9.7 mg, 16% yield, **24b**:**24′**=0.24:0.76). R_f = 0.35 (petroleum ether/ethyl acetate = 6:1). The remaining product was collected as a mixture (white solid, 9.2 mg, **24a**:**24b**:**24′** = 0.40:0.48:0.12, 15% yield). This provided a combined total isolated yield of **24** and **24′** of 85%. The regioselectivity of the acyloxy translocation reaction was determined to be 5.6:1 in the crude reaction mixture by ^{1}H NMR and 5.1:1 after isolation (**24**:**24′**). The diastereomeric ratio of **24** was determined to be 5.6:1 the crude reaction mixture by ^{1}H NMR analysis and 5.7:1 after isolation (**24a**:**24b**).

Characterization data for the major diastereomer **24a**: ^{1}H NMR (500 MHz, $CDCl_3$) δ 9.05 (s, 1H), 7.81 – 7.75 (m, 2H), 7.71 – 7.64 (m, 2H), 5.28 – 5.22 (m, 1H), 2.04 – 1.95 (m, 1H), 1.80 – 1.73 (m, 1H), 1.66 – 1.47 (m, 8H), 1.40 – 1.25 (m, 3H), 0.89 (t, *J* = 7.5 Hz, 3H).^{13}C NMR (101 MHz, $CDCl_3$) δ 160.21, 154.46, 140.47, 133.61, 119.87, 118.60, 108.71, 77.41, 42.09, 30.05, 26.97, 24.93, 24.80, 20.98, 11.62. HRMS: *Chemical formula* ([M]): $C_{17}H_{20}N_2O_3$, calcd. 301.1547 [M+H]$^+$, Found: 301.1548.

The spectra of a mixture of **24b** and **24′** (0.24:0.76) were also recorded: ^{1}H NMR (500 MHz, $CDCl_3$) δ 9.06 (s, 1H), 7.80 – 7.76 (m, 2H), 7.69 – 7.65 (m, 2H), 4.92 (p, *J* = 6.5 Hz, 0.76H), 4.72 (td, *J* = 10.6, 4.5 Hz, 0.24H), 2.06 – 2.00 (m, 0.24H), 1.99 – 1.92 (m, 0.24H), 1.84 – 1.60 (m, 5.28H), 1.51 (dd, *J* = 11.1, 3.8 Hz, 0.24H), 1.34 – 0.98 (m, 7.28H), 0.88 (t, *J* = 7.5 Hz, 0.72H). ^{13}C NMR (101 MHz, $CDCl_3$) δ 160.11, 154.42, 140.30, 140.29, 133.47, 119.83, 119.81, 118.46, 108.63, 108.62, 81.71, 79.89, 42.89, 42.33, 31.33, 29.34, 28.55, 28.43, 26.16, 25.86, 25.75, 24.91, 24.59, 24.52, 16.96, 10.57 (*While all expected carbon signals were observed for **24’** in the ^{13}C NMR spectrum, several signals for **24b** were not detected, likely due to low signal intensity or overlap with those of **24’***).

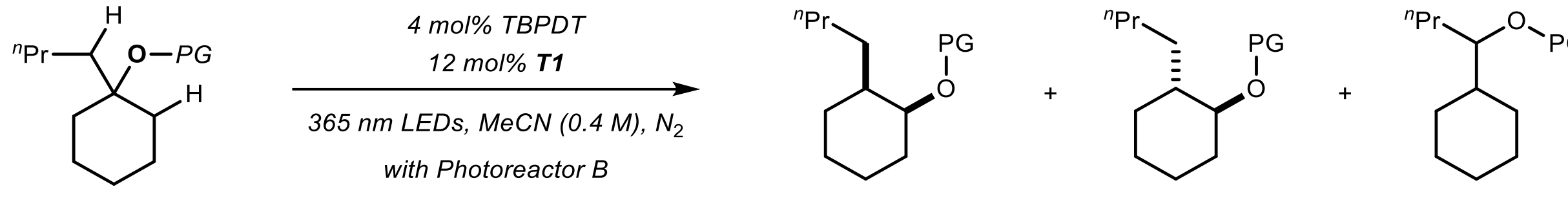

26: Following modified *General Procedure A* using 12 mol% of **T1**, two parallel reactions (0.1 mmol scale each) of compound **25** (65.6 mg total) were carried out in *Photoreactor B* for 48 h, affording **26** as a pair of diastereomers, together with the regioisomer **26′**. A portion of the major diastereomer **26a** was isolated as a pure compound (white solid, 28.8 mg, 44% yield); R_f = 0.35 (petroleum ether/ethyl acetate = 6:1). Another portion of **26a** was isolated as a mixture with **26′** (white solid, 20.5 mg, **26a**:**26′** = 0.88:0.12, 31% yield). The remaining product was collected as an mixture of **26a** and **26b** (10.2 mg, **26a**:**26b** = 0.26:0.74, 15% yield). This provided a combined total isolation yield of **26** and **26′** of 91%. The regioselectivity of the acyloxy translocation reaction was determined to be >20:1 in the crude reaction mixture by ^{1}H NMR and >20:1 after isolation (**26**:**26′**). The diastereomeric ratio of **26** was determined to be 9.2:1 the crude reaction mixture by ^{1}H NMR analysis and 6.6:1 after isolation (**26a**:**26b**).

Characterization data for the major diastereomer **26a**: ^{1}H NMR (400 MHz, $CDCl_3$) δ 9.10 (s, 1H), 7.79 (d, J = 8.8 Hz, 2H), 7.67 (d, J = 8.7 Hz, 2H), 5.27 – 5.15 (m, 1H), 2.03 – 1.92 (m, 1H), 1.78 – 1.71 (m, 1H), 1.64 – 1.50 (m, 6H), 1.34 – 1.20 (m, 7H), 0.90 – 0.82 (m, 3H). ^{13}C NMR (101 MHz, $CDCl_3$) δ 160.20, 154.50, 140.51, 133.57, 119.89, 118.60, 108.66, 77.81, 40.20, 31.62, 30.05, 29.20, 27.21, 24.94, 22.86, 20.94, 14.17. HRMS: *Chemical formula* ([M]): $C_{19}H_{24}N_2O_3$, calcd. 328.1860 $[M+H]^+$, Found: 328.1865.

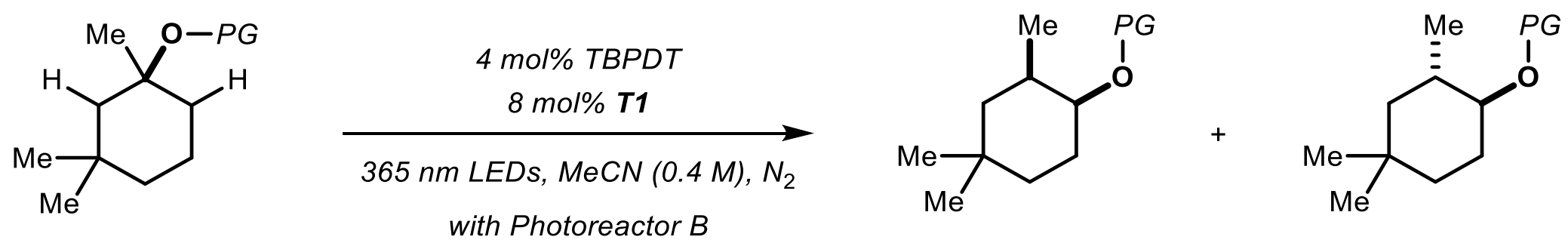

28: Following *General Procedure A*, two parallel reactions (0.1 mmol scale each) of compound **27** (62.8 mg total) were carried out in *Photoreactor B* for 48 h, affording **28** as a pair of diastereomers (51.9 mg, 83 % yield). **28** was isolated as an inseparable mixture of diastereomers (white solid, 51.9 mg, **28a**:**28b** = 0.79:0.21) R_f = 0.35 (petroleum ether/ethyl acetate = 6:1). The regioselectivity of the acyloxy translocation reaction was determined to be >20:1 in the crude reaction mixture by ^{1}H NMR and >20:1 after isolation. The diastereomeric ratio of **28** was determined to be 3.1:1 in the crude reaction mixture by ^{1}H NMR and 3.7:1 after isolation (**28a**:**28b**).

Characterization data for **28** (*as a pair of diastereomers*, **28a**:**28b =** 0.79:0.21):^{1}H NMR (400 MHz, $CDCl_3$) δ 9.16 (m, 1H), 7.80 (m, 2H), 7.70 – 7.63 (m, 2H), 5.10 (q, J = 2.8 Hz, 0.79H), 4.54 (td, J = 11.0, 4.7 Hz, 0.21H), 2.03 – 1.80 (m, 2H), 1.80 – 1.63 (m, 1.2H), 1.51 – 1.38 (m, 2H), 1.31 (td, J = 13.8, 4.2 Hz, 0.21H), 1.19 (ddq, J = 13.6, 4.9, 2.3 Hz, 1.58H), 0.97 – 0.86 (m, 8H). ^{13}C NMR (101 MHz, $CDCl_3$) δ 160.36, 154.58, 154.53, 140.54, 140.46, 133.53, 120.01, 119.91, 118.59, 108.68, 108.60, 84.18, 78.17, 46.77, 41.91, 37.46, 32.84, 32.68, 32.61, 32.36, 31.22, 30.57, 30.49, 27.47, 26.87, 24.68, 24.43, 18.67, 18.29. HRMS: *Chemical formula* ([M]): $C_{18}H_{22}N_2O_3$, calcd. 315.1703 $[M+H]^+$, Found: 315.1701.

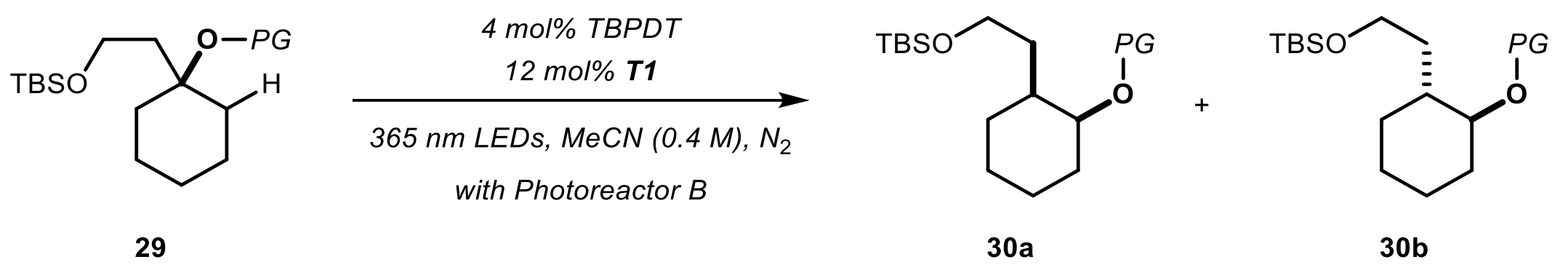

30: Following modified *General Procedure A* using 12 mol% of **T1**, two parallel reactions (0.1 mmol scale each) of compound **29** (82.6 mg total) were carried out in *Photoreactor B* for 48 h, affording **30** as a pair of diastereomers (57.6 mg, 69% yield). A portion of **30a** was isolated as a pure compound (white solid, 26.5 mg, 32% yield). R_f = 0.55 (petroleum ether/DCM/ethyl acetate = 5:2:1). A portion of **30b** was isolated as a pure compound (white solid, 8.6 mg, 10% yield). R_f = 0.5 (petroleum ether/DCM/ethyl acetate = 5:2:1). The remaining product, consisting of both **30a** and **30b**, was isolated as a diastereomeric mixture (white solid, 22.5 mg, **30a**:**30b** = 0.55:0.45, 27% yield). The regioselectivity of the acyloxy translocation reaction was determined to be >20:1 in the crude reaction mixture by ^{1}H NMR and >20:1 after isolation. The diastereomeric ratio of **30** was determined to be 1.8:1 in the crude reaction mixture by ^{1}H NMR analysis and 2.1:1 after isolation (**30a**:**30b**). A considerable amount of starting material **29** was also recovered (white solid, 14.3 mg, 17% recovery).

Characterization data for **30a**: ^{1}H NMR (400 MHz, $CDCl_3$) δ 9.06 (s, 1H), 7.82 – 7.75 (m, 2H), 7.70 – 7.63 (m, 2H), 5.26 – 5.16 (m, 1H), 3.68 – 3.58 (m, 2H), 2.05 – 1.94 (m, 1H), 1.86 (qt, *J* = 7.1, 3.7 Hz, 1H), 1.76 (dt, *J* = 12.4, 3.4 Hz, 1H), 1.63 – 1.41 (m, 7H), 1.41 – 1.29 (m, 1H), 0.87 (s, 9H), 0.03 (s, 6H). ^{13}C NMR (101 MHz, $CDCl_3$) δ 160.21, 154.45, 140.48, 133.59, 119.88, 118.59, 108.70, 77.97, 60.51, 36.55, 34.91, 29.96, 27.07, 26.07, 24.88, 20.90, 18.45, -5.22. HRMS: *Chemical formula* ([M]): $C_{23}H_{34}N_2O_4Si$, calcd. 436.2361 $[M+H]^+$, Found: 436.2356.

Characterization data for **30b**: ^{1}H NMR (400 MHz, $CDCl_3$) δ 9.04 (s, 1H), 7.83 – 7.73 (m, 2H), 7.71 – 7.64 (m, 2H), 4.74 (td, *J* = 10.1, 4.2 Hz, 1H), 3.72 – 3.60 (m, 2H), 2.09 – 1.92 (m, 2H), 1.88 – 1.66 (m, 4H), 1.52 (qd, *J* = 12.4, 3.4 Hz, 1H), 1.36 (td, *J* = 13.5, 6.6 Hz, 2H), 1.28 – 1.20 (m, 1H), 1.18 – 1.05 (m, 1H), 0.87 (s, 9H), 0.03 (d, *J* = 1.7 Hz, 6H). ^{13}C NMR (101 MHz, $CDCl_3$) δ 160.05, 154.27, 140.28, 133.48, 119.83, 112.23, 108.63, 81.79, 60.88, 38.78, 35.15, 31.23, 30.27, 25.96, 24.77, 24.31, 18.33, -5.34. HRMS: *Chemical formula* ([M]): $C_{23}H_{34}N_2O_4Si$, calcd. 453.2180 $[M+Na]^+$, Found: 453.2191.

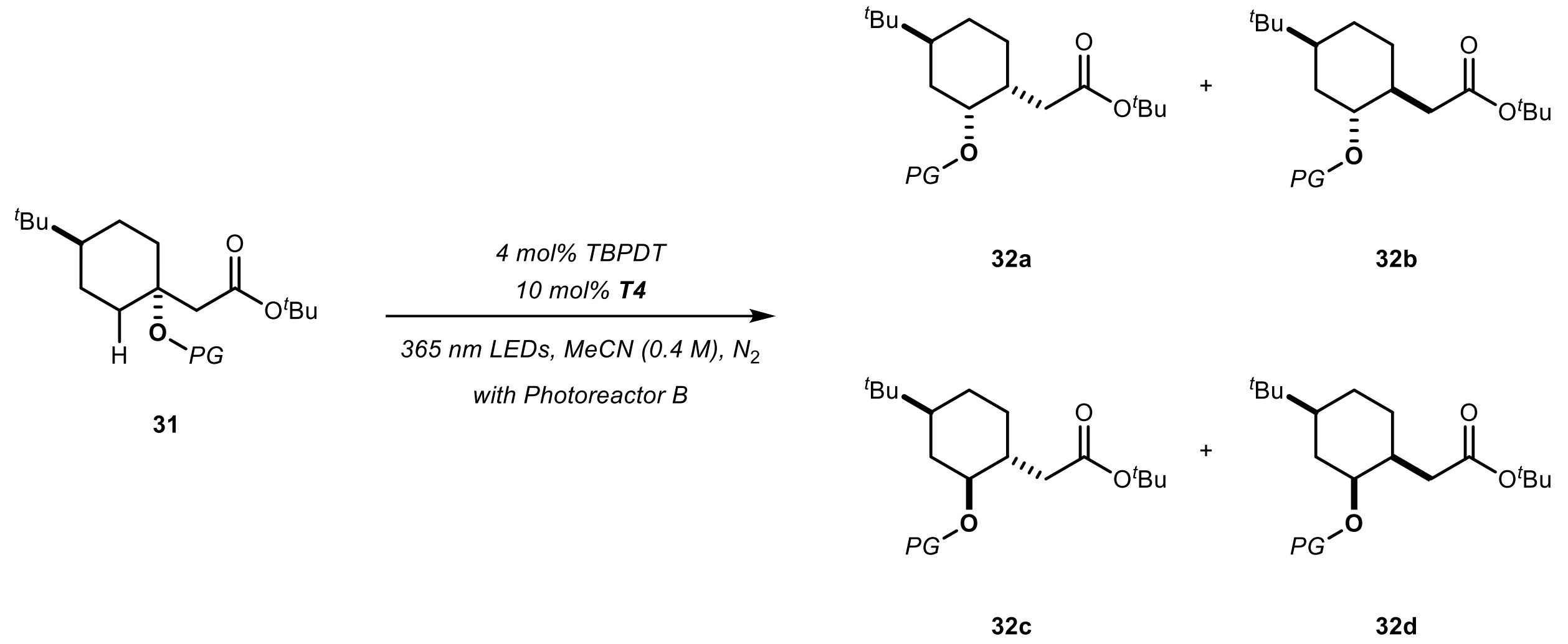

32: Following *General Procedure B*, two parallel reactions (0.1 mmol scale each) of compound **31** (88.4 mg total) were carried out in *Photoreactor B* for 48 h, affording **32** as four diastereomers (75.1 mg, 85% yield). Among them, **32a** was the major diastereomer and was isolated as a pure compound (white solid, 48.8 mg, 55% yield). R_f = 0.55 (petroleum ether/MeCN/ethyl acetate = 10:1:2). The other three minor diastereomers were isolated as an inseparable mixture of diastereomers (white solid, 26.3 mg, **32b**:**32c**:**32d** = 0.64:0.19:0.17, 30% yield). R_f = 0.5 (petroleum ether/MeCN/ethyl acetate = 10:1:2). The regioselectivity of the acyloxy translocation reaction was determined to be >20:1 in the crude reaction mixture by ^{1}H NMR and >20:1 after isolation. The diastereomeric ratio of **32** was determined to be 17:5:2.4:1 in the crude reaction mixture by ^{1}H NMR analysis and 11:4:1.1:1 after isolation (**32a**:**32b**:**32c**:**32d**).

Characterization data for **32a**: ^{1}H NMR (400 MHz, $CDCl_3$) δ 9.14 (s, 1H), 7.82 – 7.77 (m, 2H), 7.69 – 7.64 (m, 2H), 5.33 – 5.28 (m, 1H), 2.25 (dd, *J* = 15.3, 7.8 Hz, 1H), 2.17 (dd, *J* = 15.4, 6.7 Hz, 1H), 2.13 – 2.07 (m, 1H), 2.07 – 1.98 (m, 1H), 1.85 (dt, *J* = 12.5, 2.9 Hz, 1H), 1.73 – 1.65 (m, 1H), 1.63 – 1.51 (m, 1H), 1.43 – 1.30 (m, 11H), 1.11 (qd, *J* = 12.8, 3.7 Hz, 1H), 0.82 (s, 9H). ^{13}C NMR (101 MHz, $CDCl_3$) δ 171.81, 160.18, 154.43, 140.49, 133.57, 119.92, 118.58, 108.71, 80.83, 77.58, 40.92, 38.72, 37.63, 32.12, 31.36, 28.20, 27.38, 27.36, 26.46. HRMS: *Chemical formula* ([M]): C25H34N2O5, calcd. 443.2541 $[M+H]^+$, Found: 443.2545.

The spectra of a mixture of **32b**, **32c** and **32d** (0.64:0.19:0.17) were also recorded: ^{1}H NMR (500 MHz, $CDCl_3$) δ 9.37 (s, 0.17H), 9.27 (s, 0.64H), 9.11 (s, 0.19H), 7.88 – 7.78 (m, 2H), 7.70 – 7.65 (m, 2H), 5.21 – 5.18 (m, 0.19H), 5.10 (dt, *J* = 12.1, 4.7 Hz, 0.17H), 4.75 (td, *J* = 10.3, 4.3 Hz, 0.64H), 2.71 – 2.66 (m, 0.17H), 2.65 – 2.55 (m, 0.19H), 2.42 – 2.36 (m, 0.81H), 2.33 – 2.29 (m, 0.36H), 2.24 (dd, *J* = 15.5, 5.3 Hz, 0.19H), 2.17 – 2.05 (m, 2H), 2.00 – 1.88 (m, 1.17H), 1.84 – 1.75 (m, 0.83H), 1.69 – 1.50 (m, 1.28H), 1.47 (s, 1.71H), 1.41 (s, 5.76H), 1.40 (s, 1.53H), 1.33 – 1.27 (m, 1H), 1.23 – 1.05 (m, 2H), 0.89 – 0.85 (m, 9H). ^{13}C NMR (101 MHz, $CDCl_3$) δ 173.23, 172.10, 171.30, 159.83, 159.57, 159.14, 154.64, 154.58, 154.44, 140.78, 140.58, 140.44, 133.58, 133.53, 133.46, 120.05, 120.02, 119.91, 108.71, 108.66, 108.50, 82.01, 81.07, 81.03, 80.95, 79.22, 77.95, 46.81, 46.53, 41.30, 39.21, 38.94, 36.92, 34.09, 33.76, 33.09, 32.87, 32.61, 32.50, 32.31, 31.70, 30.86, 29.88, 28.21, 28.20, 28.19, 27.54, 27.51, 27.47, 27.32, 26.58, 26.22, 25.46, 22.76, 20.89, 20.69, 14.24. HRMS: *Chemical formula* ([M]): $C_{25}H_{34}N_2O_5$, calcd. 465.2360 $[M+H]^+$, Found: 465.2375.

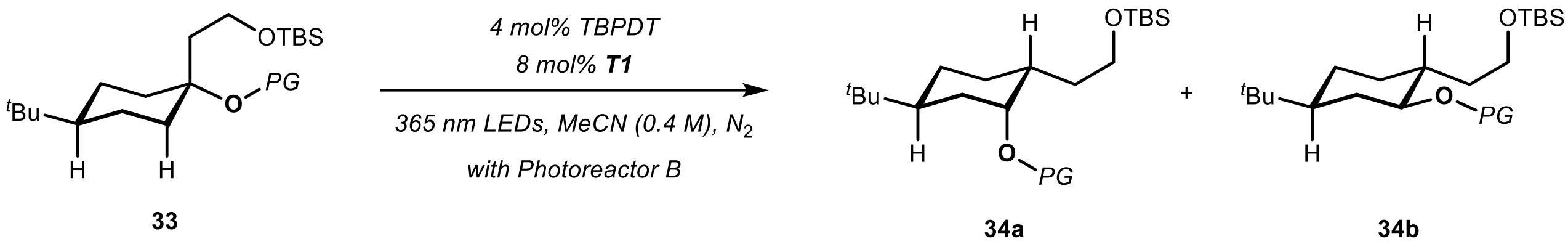

34: Following *General Procedure A*, two parallel reactions (0.1 mmol scale each) of compound **33** (97.4 mg total) were carried out in *Photoreactor B* for 12 h, affording **34** as two diastereomers (66.5 mg, 68% yield). The other two diastereomers were formed in trace amount and can only be observed on the ^{1}H NMR spectrum of the crude reaction.

The major diastereomer **34a** was isolated as a pure compound (white solid, 48.8 mg, 55% yield). R_f = 0.65 (petroleum ether/MeCN/ethyl acetate = 10:1:2). The minor diastereomer **34b** was isolated as a mixture with **33** (white solid, 26.3 mg, **34b**:**33** = 0.76:0.24, 11% yield). R_f = 0.5 (petroleum ether/MeCN/ethyl acetate = 10:1:2). This provided a combined total yield of 68%. The regioselectivity of the acyloxy translocation reaction was determined to be >20:1 in the crude reaction mixture by ^{1}H NMR and >20:1 after isolation. The diastereomeric ratio of **34** was determined to be 6.7:1 in the crude reaction mixture by ^{1}H NMR analysis and 5.4:1 after isolation (**34a**:**34b**).

Characterization data for the major diastereomer **34a**: ^{1}H NMR (600 MHz, $CDCl_3$) δ 9.14 (s, 1H), 7.80 (d, *J* = 8.7 Hz, 2H), 7.66 (d, *J* = 8.7 Hz, 2H), 5.30 (s, 1H), 3.66 – 3.58 (m, 2H), 2.07 (d, *J* = 14.0 Hz, 1H), 1.84 (d, *J* = 13.1 Hz, 1H), 1.74 – 1.66 (m, 2H), 1.56 – 1.48 (m, 2H), 1.47 – 1.38 (m, 2H), 1.29 (t, *J* = 13.4 Hz, 1H), 1.10 – 1.01 (m, 1H), 0.87 (s, 9H), 0.81 (s, 9H), 0.02 (s, 6H). ^{13}C NMR (151 MHz, $CDCl_3$) δ 160.13, 154.39, 140.42, 133.43, 119.79, 118.47, 108.54, 78.31, 60.41, 41.15, 36.80, 35.26, 32.00, 31.45, 27.29, 27.11, 26.52, 25.96, 18.34, -5.34. HRMS: *Chemical formula* ([M]): $C_{27}H_{42}N_2O_4Si$, calcd. 487.2987 $[M+H]^+$, Found: 487.2979.

The spectra of a mixture of **34b** and **33** (0.76:0.24 ratio) were also recorded: ^{1}H NMR (600 MHz, $CDCl_3$) δ 9.06 (s, 1H), 7.78 (d, *J* = 8.1 Hz, 2H), 7.67 (d, *J* = 8.4 Hz, 2H), 4.75 (td, *J* = 11.0, 4.1 Hz, 1H), 3.68 – 3.63 (m, 2H), 2.07 (d, *J* = 11.7 Hz, 1H), 2.02 (d, *J* = 13.7 Hz, 1H), 1.77 – 1.74 (m, 2H), 1.58 (d, *J* = 13.4 Hz, 1H), 1.47 (dt, *J* = 22.0, 12.7 Hz, 1H), 1.32 – 1.24 (m, 2H), 1.20 (t, *J* = 10.7 Hz, 2H), 0.87 (s, 9H), 0.86 (s, 9H), 0.03 – 0.02 (m, 6H). ^{13}C NMR (151 MHz, $CDCl_3$) δ 160.09, 154.28, 140.27, 133.46, 119.83, 118.45, 108.62, 82.74, 60.90, 46.49, 39.04, 35.22, 32.96, 32.37, 30.20, 27.43, 26.14, 25.96, 18.33, -5.28.

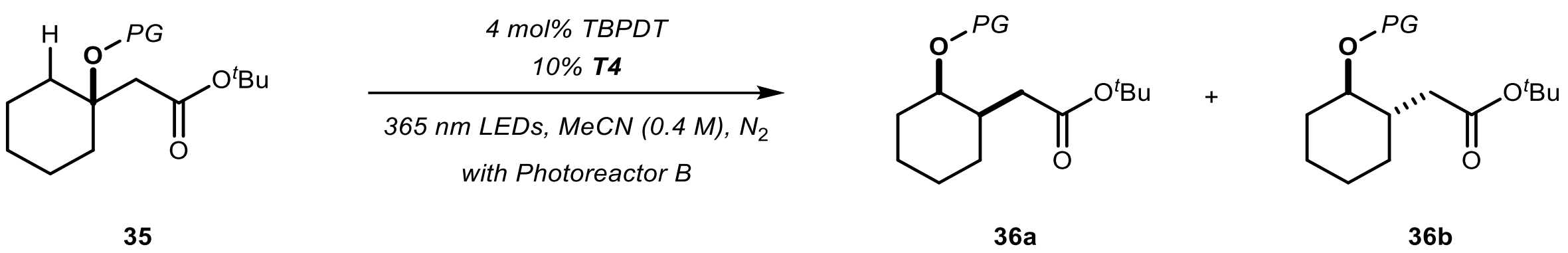

36: Following *General Procedure B*, two parallel reactions (0.1 mmol scale each) of compound **35** (77.2 mg total) were carried out in *Photoreactor B* for 48 h, affording **36** as a pair of diastereomers. A portion of the major diastereomer **36a** was isolated as a mixture with 4-aminobenzonitrile (white solid, 31.1 mg, *mixed with 5.5% of 4-aminobenzonitrile*, corresponding to 30.2 mg of **36a**, 39% yield). R_f = 0.5 (petroleum ether/DCM/ethyl acetate = 5:2:1). A portion of **36b** was isolated as a pure compound (white solid, 4.0 mg, 5 % yield). R_f = 0.48 (petroleum

ether/DCM/ethyl acetate = 5:2:1). The remaining product, consisting of both **36a** and **36b**, was isolated as a diastereomeric mixture (white solid, 30.3 mg, **36a**:**36b** = 0.45:0.55, 39% yield). This corresponds to a total yield of **36** of 83% yield. The regioselectivity of the acyloxy translocation reaction was determined to be >20:1 in the crude reaction mixture by ^{1}H NMR and >20:1 after isolation. The diastereomeric ratio of **36** was determined to be 2:1 in the crude reaction mixture by ^{1}H NMR and 2.1:1 after isolation (**36a**:**36b**). A considerable amount of starting material **35** was also recovered (white solid, 10.1 mg, 13% recovery).

Characterization data for the major diastereomer **36a** (*mixed with 5.5% of 4-aminobenzonitrile*): ^{1}H NMR (400 MHz, $CDCl_3$) δ 9.23 (s, 1H), 7.82 (d, *J* = 8.6 Hz, 2H), 7.68 (d, *J* = 8.6 Hz, 2H), 5.21 (dt, *J* = 5.4, 2.4 Hz, 1H), 2.40 – 2.27 (m, 1H), 2.27 – 2.15 (m, 2H), 2.02 – 1.92 (m, 1H), 1.75 – 1.48 (m, 6H), 1.47-1.35 (m, 10H). ^{13}C NMR (101 MHz, $CDCl_3$) δ 172.06, 159.94, 154.48, 140.56, 133.52, 119.96, 118.60, 108.61, 80.86, 77.08, 38.08, 36.83, 29.37, 28.18, 27.63, 24.23, 20.86. HRMS: *Chemical formula* ([M]): $C_{21}H_{26}N_2O_5$, calcd. 387.1915 $[M+H]^+$, Found: 387.1909.

Characterization data for the minor diastereomer **36b**: ^{1}H NMR (400 MHz, $CDCl_3$) δ 9.23 (s, 1H), 7.86 – 7.75 (m, 2H), 7.71 – 7.62 (m, 2H), 4.71 (td, *J* = 10.5, 4.3 Hz, 1H), 2.45 – 2.35 (m, 1H), 2.19 – 2.09 (m, 3H), 1.98 – 1.90 (m, 1H), 1.87 – 1.80 (m, 1H), 1.76 – 1.68 (m, 1H), 1.52 – 1.46 (m, 1H), 1.41 (s, 9H), 1.38 – 1.27 (m, 2H), 1.24 – 1.14 (m, 1H). ^{13}C NMR (101 MHz, $CDCl_3$) δ 172.12, 159.55, 154.52, 140.56, 133.57, 120.03, 118.64, 108.72, 81.31, 81.01, 39.34, 38.89, 31.43, 31.25, 28.24, 25.06, 24.52. HRMS: *Chemical formula* ([M]): $C_{21}H_{26}N_2O_5$, calcd. 409.1734 $[M+Na]^+$, Found: 409.1718.

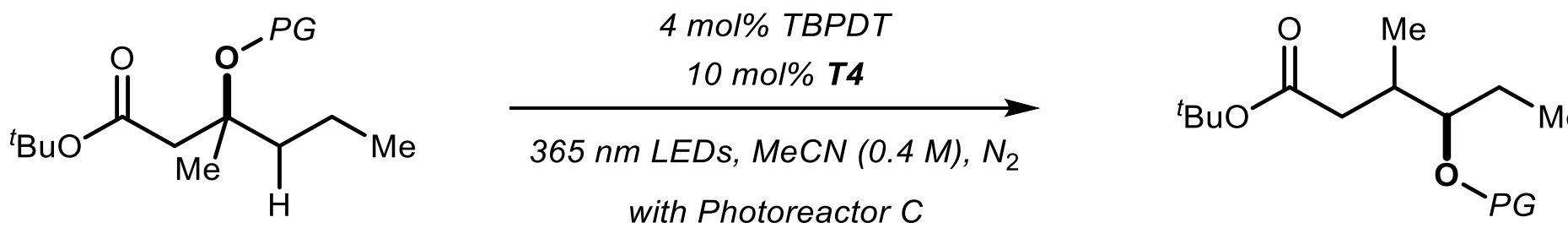

37 **38**

(obtained as a pair of diastereomers)

38: Following *General Procedure B*, two parallel reactions (0.1 mmol scale each) of compound **37** (74.8 mg total) were carried out in *Photoreactor C* for 48 h, affording **38** as a pair of diastereomers (white solid, 35.1 mg, 47% yield). R_f = 0.5 (petroleum ether/DCM/ethyl acetate = 5:2:1). This provided a combined total yield of 68%. The regioselectivity of the acyloxy translocation reaction was determined to be >20:1 in the crude reaction mixture by ^{1}H NMR and >20:1 after isolation. The diastereomeric ratio of **38** was determined to be 1.1:1 in the crude reaction mixture by ^{1}H NMR analysis and 1.2:1 after isolation. A considerable amount of starting material **37** was also recovered (white solid, 23.1 mg, 31% recovery).

Characterization data for **38** *(as a pair of diastereomers)*: ^{1}H NMR (400 MHz, $CDCl_3$) δ 9.26 (s, 0.46H), 9.19 (s, 0.54H), 7.86 – 7.79 (m, 2H), 7.71 – 7.65 (m, 2H), 5.02 (ddd, *J* = 8.4, 4.9, 3.7 Hz, 0.54H), 4.95 (q, *J* = 5.9 Hz, 0.46H), 2.43 – 2.32 (m, 2H), 2.21 – 2.11 (m, 1H), 1.82 – 1.64 (m, 2H), 1.47 – 1.42 (m, 9H), 1.07 – 1.00 (m, 3H), 0.98 – 0.92 (m, 3H). ^{13}C NMR (101 MHz, $CDCl_3$) δ 172.02, 171.78, 160.34, 160.13, 154.40, 154.39, 140.54, 140.49, 133.54, 133.53, 119.99, 119.97, 118.60, 118.59, 108.67, 108.66, 83.42, 82.90, 80.93, 80.91, 39.12, 38.38, 33.34, 33.16, 28.18, 24.16, 23.98, 16.44, 14.56, 10.25, 9.81. (*In the ^{13}C NMR spectrum, signal overlap between the two diastereomers of **38** occurs for the methyl groups of the tert-butyl ester, resulting in one fewer carbon signal than expected.*) HRMS: *Chemical formula* ([M]): $C_{20}H_{26}N_2O_5$, calcd. 375.1915 $[M+H]^+$, Found: 375.1908.

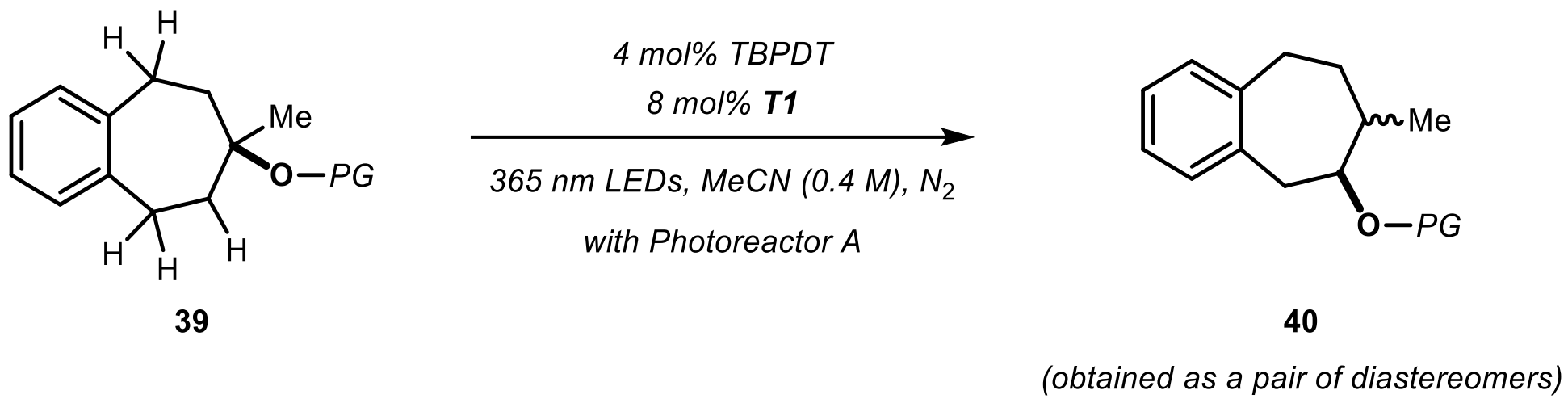

40: Following *General Procedure A*, two parallel reactions (0.1 mmol scale each) of compound **39** (69.6 mg in total) were carried out in *Photoreactor A* for 48 h, affording **40** as a pair of diastereomers. A portion of **40-*diastereomer 1*** was isolated as a pure compound (white solid, 8.9 mg, 13% yield). R_f = 0.32 (petroleum ether/MeCN/ethyl acetate = 10:1:2). The remaining product was collected as a mixture with **39** (white solid, 30.1 mg, **40-*diastereomer 1***:**40-*diastereomer 2***:**39** = 0.60:0.20:0.20, corresponding to 24.1 mg of **40**, 36% yield). This provided a combined total yield of 47%. The regioselectivity of the acyloxy translocation reaction was determined to be >20:1 in the crude reaction mixture by ^{1}H NMR and >20:1 after isolation. The diastereomeric ratio of **40** was determined to be 2.8:1 in the crude reaction mixture by ^{1}H NMR and 3.8:1 after isolation.

Characterization data for **40-*diastereomer 1***: ^{1}H NMR (600 MHz, $CDCl_3$) δ 8.27 (s, 1H), 7.62 (d, *J* = 8.4 Hz, 2H), 7.56 (d, *J* = 8.3 Hz, 2H), 7.20 – 7.17 (m, 2H), 7.09 (dt, *J* = 8.4, 4.3 Hz, 1H), 7.01 (d, *J* = 7.4 Hz, 1H), 5.21 (d, *J* = 8.1 Hz, 1H), 3.36 (dd, *J* = 14.6, 8.2 Hz, 1H), 3.12 (d, *J* = 14.1 Hz, 1H), 2.90 (dd, *J* = 14.1, 7.6 Hz, 1H), 2.88 – 2.80 (m, 1H), 2.23 – 2.14 (m, 1H), 1.87 – 1.81 (m, 1H), 1.66 – 1.57 (m, 1H), 1.07 (d, *J* = 7.0 Hz, 3H). ^{13}C NMR (151 MHz, $CDCl_3$) δ 158.82, 154.13, 142.73, 140.11, 135.29, 132.76, 130.16, 129.09, 127.39, 126.52, 119.74, 118.43, 108.55, 78.21, 40.10, 37.65, 30.41. (*Two alkyl carbon signals for **40-diastereomer 1** were not observed in the ^{13}C NMR spectrum, likely due to signal broadening.* HRMS: *Chemical formula* ([M]): $C_{21}H_{20}N_2O_3$, calcd. 349.1547 $[M+H]^+$, Found: 349.1549.

The spectra of a mixture of **40-*diastereomer 1***, **40-*diastereomer 2***, and **39** (0.60:0.20:0.20) were also recorded: ^{1}H NMR (500 MHz, $CDCl_3$) δ 9.14 – 9.05 (m, 0.4H), 8.31 (s, 0.6H), 7.81 – 7.76 (m, 0.8H), 7.69 – 7.65 (m, 0.8H), 7.63 – 7.60 (m, 1.2H), 7.58 – 7.54 (m, 1.2H), 7.19 – 7.15 (m, 1.8H), 7.13 – 7.07 (m, 1.6H), 7.01 (d, *J* = 7.2 Hz, 0.6H), 5.21 (d, *J* = 6.9 Hz, 0.6H), 4.67 (td, *J* = 10.2, 1.9 Hz, 0.2H), 3.36 (dd, *J* = 14.5, 8.2 Hz, 0.6H), 3.30 (dd, *J* = 13.7, 10.6 Hz, 0.2H), 3.16 – 3.06 (m, 1H), 2.96 – 2.75 (m, 1.6H), 2.68 – 2.55 (m, 0.8H), 2.30 – 2.14 (m, 0.8H), 2.04 (dddd, *J* = 14.3, 7.5, 3.8, 1.5 Hz, 0.2H), 1.87 – 1.80 (m, 0.6H), 1.72 – 1.56 (m, 1.8H), 1.36 – 1.27 (m, 0.2H), 1.07 (d, *J* = 6.9 Hz, 1.8H), 1.01 (d, *J* = 6.6 Hz, 0.6H). ^{13}C NMR (101 MHz, $CDCl_3$) δ 159.88, 159.34, 158.96, 155.03, 154.43, 154.28, 143.19, 142.81, 141.93, 140.52, 140.40, 140.26, 135.37, 135.28, 133.55, 133.55, 133.42, 130.26, 130.24, 129.18, 129.00, 128.89, 127.49, 127.47, 126.76, 126.59, 126.58, 119.99, 119.89, 119.88, 118.61, 118.57, 118.55, 108.74, 108.58, 108.57, 89.98, 82.09, 78.30, 41.50, 40.66, 40.04, 38.01, 37.72, 34.04, 33.89, 33.78, 30.49, 29.58, 26.74, 19.70, 18.73. (*The carbon signals at δ 40.04, 33.89, and 18.73 were observed as broad signals.*)

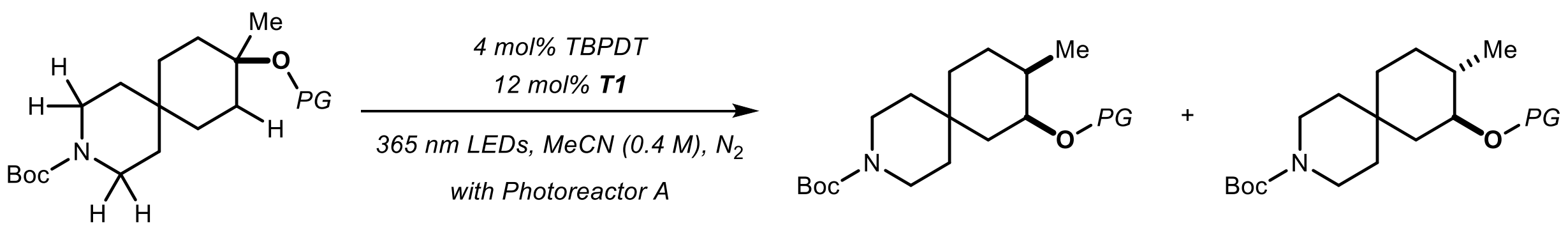

42: Following modified *General Procedure A* using 12 mol% of **T1**, two parallel reactions (0.1 mmol scale each) of compound **41** (91.1 mg total) were carried out in *Photoreactor A* for 48 h, affording **42** as a pair of diastereomers (22.5 mg, 25% yield). A portion of **42** was isolated as itself as a colorless oil (15.5 mg, **42a**:**42b** = 0.42:0.58, 17% yield). R_f = 0.36 (petroleum ether/ethyl acetate = 3:1). The remainder of **42** was obtained as a mixture with **41** (11.6 mg total, **42a**:**42b**:**41** = 0.36:0.24:0.40, corresponding to 7.0 mg of **42**, 8% yield). This provided a combined total yield of 25%. The regioselectivity of the acyloxy translocation reaction was determined to be >20:1 in the crude reaction mixture by ^{1}H NMR and >20:1 after isolation. The diastereomeric ratio of **42** was determined to 1:1.1 after isolation (**42a**:**42b**).

Characterization data for of **42** *(as a pair of diastereomers,* **42a**:**42b** = 0.42:0.58): ^{1}H NMR (400 MHz, $CDCl_3$) δ 9.07 (s, 0.58H), 9.05 (s, 0.42H), 7.82 – 7.74 (m, 2H), 7.71 – 7.64 (m, 2H), 5.19 (dt, *J* = 6.2, 3.4 Hz, 0.42H), 4.77 (td, *J* = 10.9, 4.2 Hz, 0.58H), 3.47 – 3.26 (m, 4H), 2.05 (ddd, *J* = 12.7, 4.3, 2.1 Hz, 0.58H), 1.97 – 1.84 (m, 1H), 1.84 – 1.13 (m, 18.42H), 0.98 (d, *J* = 3.0 Hz, 1.26H), 0.97 (d, *J* = 2.5 Hz, 1.74H). ^{13}C NMR (101 MHz, $CDCl_3$) *signals for **42a** and **42b** were observed at* δ 160.35, 160.24, 155.09, 154.34, 140.36, 133.61, 119.97, 119.93, 118.57, 108.80, 80.29, 79.58, 79.47, 78.08, 40.29, 39.89, 37.56, 34.99, 33.96, 28.59, 18.34. HRMS: *Chemical formula* ([M]): $C_{25}H_{33}N_3O_5$, calcd. 456.2493 $[M+H]^+$, Found: 456.2492.

6. Further studies

6.1 Showcase of 1,2-hydroxyl translocation *via* one-pot deprotection

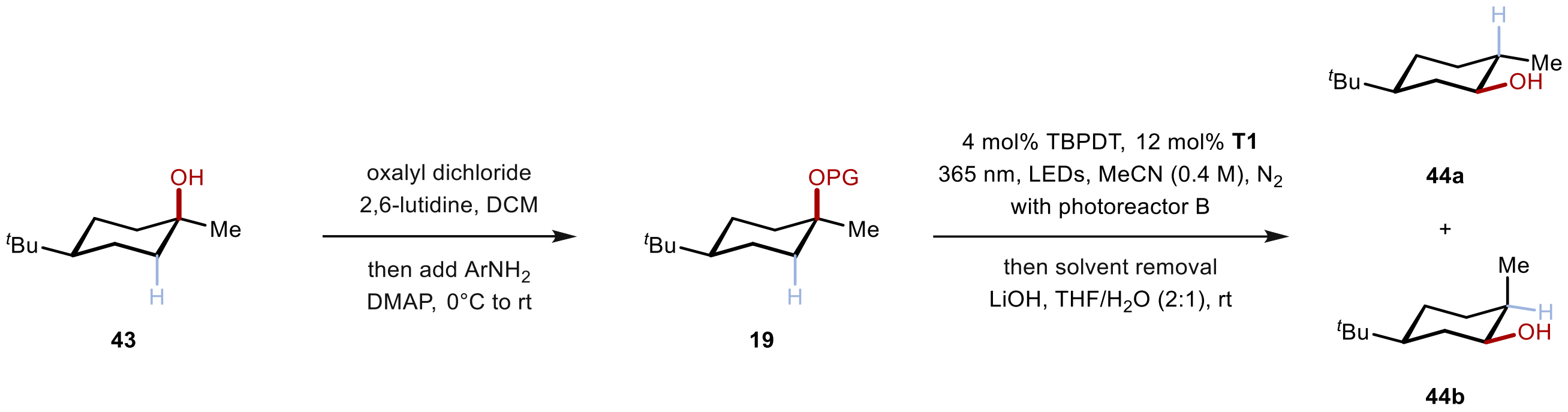

19: 19 was obtained following the procedure reported in *Section 4*.

44: An oven-dried 4 mL vial was charged with **19** (34.2 mg, 0.1 mmol, 1.0 equiv) and tetrabutylphosphonium decatungstate (TBPDT, 13.6 mg, 0.004 mmol, 0.04 equiv). The vial was transferred to a nitrogen-filled glovebox, where MeCN (0.22 mL) and a stock solution of 2,4,6-triisopropylbenzenethiol (**T1**) (0.4 M in MeCN, 30 μL, 0.12 equiv) were added. After sealing with a PTFE-lined screw cap, the vial was removed from the glovebox and placed in *photoreactor B* for 12 h. Two parallel runs were conducted, combined upon completion, and concentrated under reduced pressure. The residue was then dissolved in THF/H_2O (2:1, 1.2 mL), and LiOH·H_2O (16.8 mg, 0.4 mmol, 2.0 equiv) was added. The resulting mixture was stirred at room temperature for 20 min and monitored by TLC until complete consumption of the starting material. The reaction mixture was quenched with 2 M HCl (0.5 mL) and extracted with diethyl ether. The combined organic layers were dried over anhydrous Na_2SO_4 and concentrated under reduced pressure. The residue was purified by flash column chromatography on silica gel to afford two major products, **44a** and **44b**. **44a** was isolated as a white solid (20.9 mg, 61% yield). The remaining **44a** was isolated as a mixture with **44b** (6.1 mg, **44a**:**44b** = 0.56:0.44, 18%). The total yield of **44a** and **44b** was 79%. The diastereomeric ratio was found to be 6.9:1 for the isolated product. The characterization data for **44a**: ^{1}H NMR (500 MHz, $CDCl_3$) δ 3.12 (td, *J* = 10.0, 9.5, 4.2 Hz, 1H), 2.04 – 1.96 (m, 1H), 1.79 – 1.71 (m, 1H), 1.70 – 1.63 (m, 1H), 1.51 (s, 1H), 1.27 – 1.17 (m, 1H), 1.10 (tt, *J* = 11.9, 2.9 Hz, 1H), 1.03 – 0.93 (m, 6H), 0.86 (s, 9H). The spectrum matches that reported in literature.[16]

6.2 Synthetic versatility of the oxamoyloxy moiety beyond deprotection

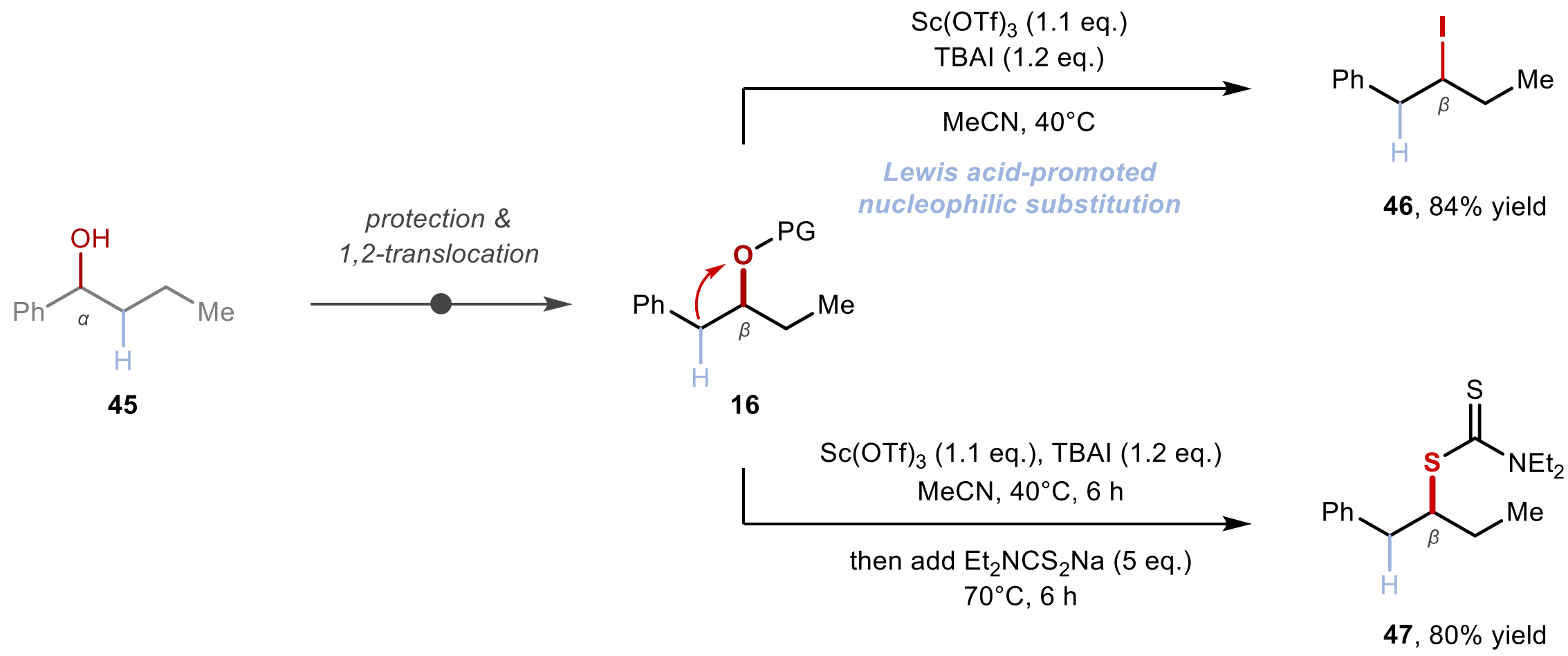

46: A 4 mL vial was charged with **16** (64.4 mg, 0.2 mmol, 1 equiv) and TBAI (88.6 mg, 0.24 mmol, 1.2 equiv). The vial was transferred into a glovebox, and $Sc(OTf)_3$ (108.2 mg, 0.22 mmol, 1.1 equiv) and MeCN (1.0 mL) were added. The vial was sealed with a PTFE-lined screw cap, removed from the glovebox, and stirred at 40 °C for 6 h. The reaction was cooled to room temperature and the solvent was removed under reduced pressure. The residue was purified by filtration through a silica gel plug using pentane as the eluent. The filtrate was concentrated under reduced pressure to afford **46** as a colorless oil (43.8 mg, 84% yield). R_f = 0.80 (petroleum ether). ^{1}H NMR (500 MHz, C_6D_6) δ 7.11 – 7.07 (m, 2H), 7.07 – 7.03 (m, 1H), 6.89 (dd, *J* = 6.8, 1.8 Hz, 2H), 3.95 (qd, *J* = 7.8, 4.0 Hz, 1H), 3.05 (dd, *J* = 14.2, 7.5 Hz, 1H), 2.82 (dd, *J* = 14.2, 7.3 Hz, 1H), 1.48 – 1.37 (m, 2H), 0.82 (t, *J* = 7.2 Hz, 3H). ^{13}C NMR (101 MHz, C_6D_6) δ 128.88, 128.29, 126.58, 47.04, 40.21, 32.28, 13.94.

47: A 4 mL vial was charged with **16** (64.4 mg, 0.2 mmol, 1 equiv) and TBAI (88.6 mg, 0.24 mmol, 1.2 equiv). The vial was transferred into a glovebox, and $Sc(OTf)_3$ (108 mg, 0.22 mmol, 1.1 equiv) and MeCN (1.0 mL) were added. The vial was sealed with a PTFE-lined screw cap, removed from the glovebox, and stirred at 40 °C for 6 h. The mixture was cooled to room temperature, and Et_2NCS_2Na (171 mg, 1.0 mmol, 5 equiv) was added. The reaction mixture was heated to 70 °C and stirred for 6 h. The solvent was removed under reduced pressure. The residue was purified by flash column chromatography on silica gel to afford **47** as a yellow oil (45.0 mg, 80% yield). R_f = 0.26 (petroleum ether/ethyl acetate = 40:1). ^{1}H NMR (400 MHz, $CDCl_3$) δ 7.36 – 7.25 (m, 4H), 7.23 – 7.17 (m, 1H), 4.24 (ddd, *J* = 13.7, 9.7, 5.0 Hz, 1H), 4.09 – 3.96 (m, 2H), 3.73 (h, *J* = 7.0 Hz, 2H), 3.26 (dd, *J* = 13.7, 5.3 Hz, 1H), 2.80 (dd, *J* = 13.7, 9.2 Hz, 1H), 1.77 (dqd, *J* = 14.6, 7.4, 4.7 Hz, 1H), 1.60 (dq, *J* = 21.6, 7.4 Hz, 1H), 1.27 (t, *J* = 7.1 Hz, 6H), 1.00 (t, *J* = 7.3 Hz, 3H). ^{13}C NMR (101 MHz, $CDCl_3$) δ 195.48, 139.42, 129.50, 128.21, 126.29, 77.43, 77.11, 76.79, 55.04, 49.23, 46.70, 40.89, 25.23, 12.52, 11.71, 11.50. HRMS: *Chemical formula* ([M]): $C_{15}H_{23}NS_2$, calcd. 282.1345 $[M+H]^+$, Found: 282.1343.

6.3 Late-stage modification of complex bioactive molecules

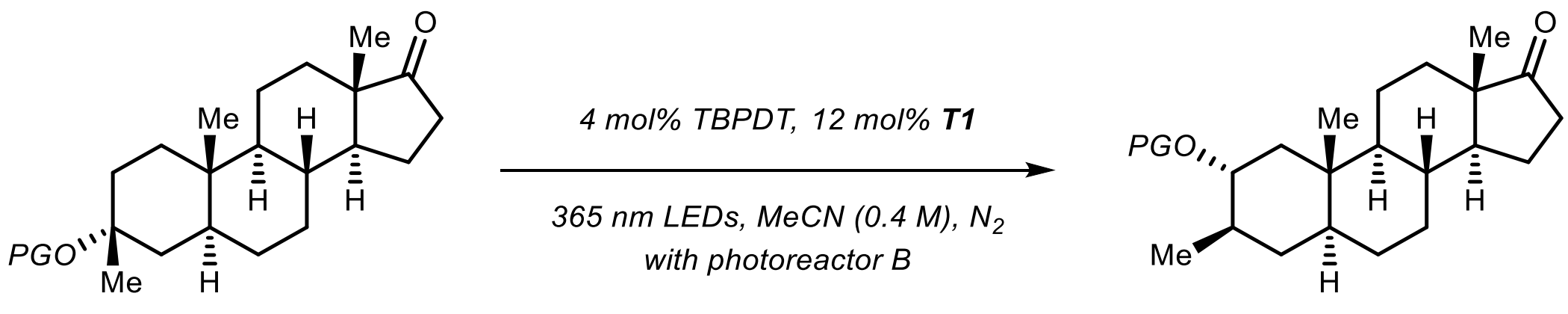

49 **50**

50: An oven-dried 4 mL vial was charged with **49** (47.6 mg, 0.1 mmol, 1.0 equiv) and tetrabutylphosphonium decatungstate (TBPDT, 13.6 mg, 0.004 mmol, 0.04 equiv). The vial was transferred to a nitrogen-filled glovebox, where MeCN (0.22 mL) and a stock solution of 2,4,6-triisopropylbenzenethiol (**T1**) (0.4 M in MeCN, 30 µL, 0.12 equiv) were added. After sealing with a PTFE-lined screw cap, the vial was removed from the glovebox and placed in *photoreactor B* for 48 h. Two parallel runs were conducted and combined upon completion. The resulting mixture was concentrated under reduced pressure, and the residue was purified by flash column chromatography on silica gel to afford **50** as a white solid (63.7 mg, 67% yield). R_f= 0.34 (petroleum ether/ethyl acetate = 6:1). The diastereomeric ratio and regioisomer ratio of **50** were determined to be >20:1 both in the crude reaction mixture and after isolation. ^{1}H NMR (400 MHz, $CDCl_3$) δ 9.22 (s, 1H), 7.79 (d, *J* = 8.7 Hz, 2H), 7.65 (d, *J* = 8.7 Hz, 2H), 4.80 (td, *J* = 11.1, 4.5 Hz, 1H), 2.41 (dd, *J* = 19.2, 8.8 Hz, 1H), 2.11 – 1.98 (m, 2H), 1.91 (ddd, *J* = 14.1, 8.6, 5.8 Hz, 1H), 1.77 (m, 3H),

1.61 – 1.42 (m, 4H), 1.42 – 1.08 (m, 9H), 1.04 – 0.72 (m, 12H). ^{13}C NMR (101 MHz, $CDCl_3$) δ 221.14, 160.24, 154.53, 140.49, 133.48, 120.02, 118.57, 108.58, 81.36, 54.48, 51.30, 47.81, 46.08, 43.29, 38.04, 37.43, 36.24, 35.87, 34.42, 31.47, 30.65, 27.53, 21.81, 20.54, 18.35, 13.86, 13.22. HRMS: *Chemical formula* ([M]): $C_{29}H_{36}N_2O_4$, calcd. 499.2561 $[M+Na]^+$, Found: 499.2576.

7. References

(1) Xu, W.; Ma, J.; Yuan, X.-A.; Dai, J.; Xie, J.; Zhu, C. Synergistic Catalysis for the Umpolung Trifluoromethylthiolation of Tertiary Ethers. *Angew. Chem. Int. Ed.* **2018**, *57*, 10357-10361.

(2) Graton, J.; Compain, G.; Besseau, F.; Bogdan, E.; Watts, J. M.; Mtashobya, L.; Wang, Z.; Weymouth-Wilson, A.; Galland, N.; Le Questel, J.-Y.; Linclau, B. Influence of Alcohol β-Fluorination on Hydrogen-Bond Acidity of Conformationally Flexible Substrates. *Chem. Eur. J.* **2017**, *23*, 2811-2819.

(3) Martinez B. G.; Salituro F. G.; Robichaud, A. J.; Harrison B. L. Compositions and Methods for Treating CNS Disorders. WO2016/061527, April 21, 2016.

(4) Miller, D. C.; Ganley, J. M.; Musacchio, A. J.; Sherwood, T. C.; Ewing, W. R.; Knowles, R. R. Anti-Markovnikov Hydroamination of Unactivated Alkenes with Primary Alkyl Amines. *J. Am. Chem. Soc.* **2019**, *141*, 16590–16594.

(5) Sarver, P. J.; Bissonnette, N. B.; MacMillan, D. W. C. Decatungstate-Catalyzed C(sp^3)- H Sulfinylation: Rapid Access to Diverse Organosulfur Functionality. *J. Am. Chem. Soc.* **2021**, *143*, 9737-9743.

(6) Perry, I. B.; Brewer, T. F.; Sarver, P. J.; Schultz, D. M.; DiRocco, D. A.; MacMillan, D. W. C. Direct Arylation of Strong Aliphatic C-H Bonds. *Nature* **2018**, *560*, 70-75.

(7) Oswood, C. J.; MacMillan, D. W. C. Selective Isomerization via Transient Thermodynamic Control: Dynamic Epimerization of Trans to Cis Diols. *J. Am. Chem. Soc.* **2022**, *144*, 93-98.

(8) Curran, D. P.; Scanga, S. A.; Fenk, C. J. Reduction of Substituted .DELTA.2-Isoxazolines. Synthesis of .Beta.-Hydroxy Acid Derivatives. *J. Org. Chem.* **1984**, *49*, 3474-3478.

(9) Chen, K.; Zeng, Q.; Xie, L.; Xue, Z.; Wang, J.; Xu, Y. Functional-Group Translocation of Cyano Groups by Reversible C-H Sampling. *Nature* **2023**, *620*, 1007-1012.

(10) Ishida, N.; Shirahama, S.; Okawa, T.; Matsui, S. Traction Drive Fluid. US6638417B2, October 28, 2003.

(11) Jordan, E. A.; Thorne, M. P. 13C nmr Determination of the Conformational Equilibria in the Acetates, Benzoates and n-Phenylcarbamates of 1-Methylcyclohexanol and Cyclohexanol. *Tetrahedron* **1986**, *42*, 93-99.

(12) Yang, Y.-C.; Li, W.-S.; Uang, T.-Y.; Kuo, T.-S.; Wu, H.-L. Access to Chiral Tertiary α-Hydroxyesters via Rhodium-Catalyzed Enantioselective Allylation of α-Ketoesters. *Chem. Asian J.* **2026**, *21*, e70585.

(13) Gao, Y.; Wu, Z.; Yu, L.; Wang, Y.; Pan, Y. Alkyl Carbazates for Electrochemical Deoxygenative Functionalization of Heteroarenes. *Angew. Chem. Int. Ed.* **2020**, *59*, 10859-10863.

(14) Bartel, K.; Goosen, A.; Scheffer, A. Hypoiodite Reaction: The Decomposition of Oxalic Acid Half-Esters. *J. Chem. Soc. C* **1971**, 3766-3769.

(15) Ogbu, I. M.; Bassani, D. M.; Robert, F.; Landais, Y. Photocatalyzed Decarboxylation of Oxamic Acids under Near-Infrared Conditions. *Chem. Commun.* **2022**, *58*, 8802-8805.

(16) Iimori, T.; Ohtsuka, Y.; Oishi, T. Regioselective Dehydration in Cyclic System with Triphenylphosphine-Azodicarboxylate. *Tetrahedron Lett.* **1991**, *32*, 1209-1212.

8. Spectra

Figure S11 | ^{1}H NMR (500 MHz, $CDCl_3$) spectrum of **S14**.

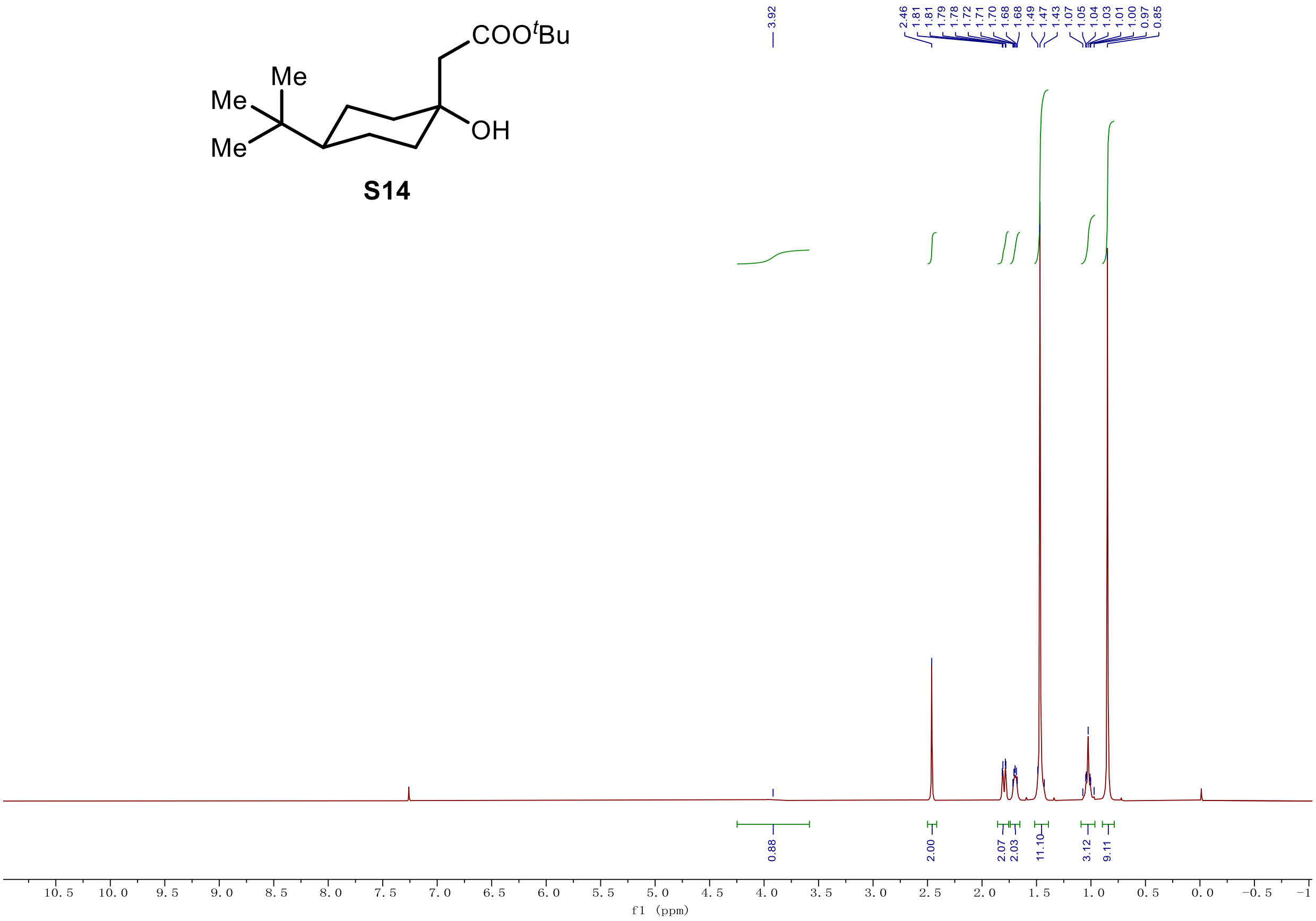

Figure S12 | ^{13}C NMR (126 MHz, $CDCl_3$) spectrum of **S14**.

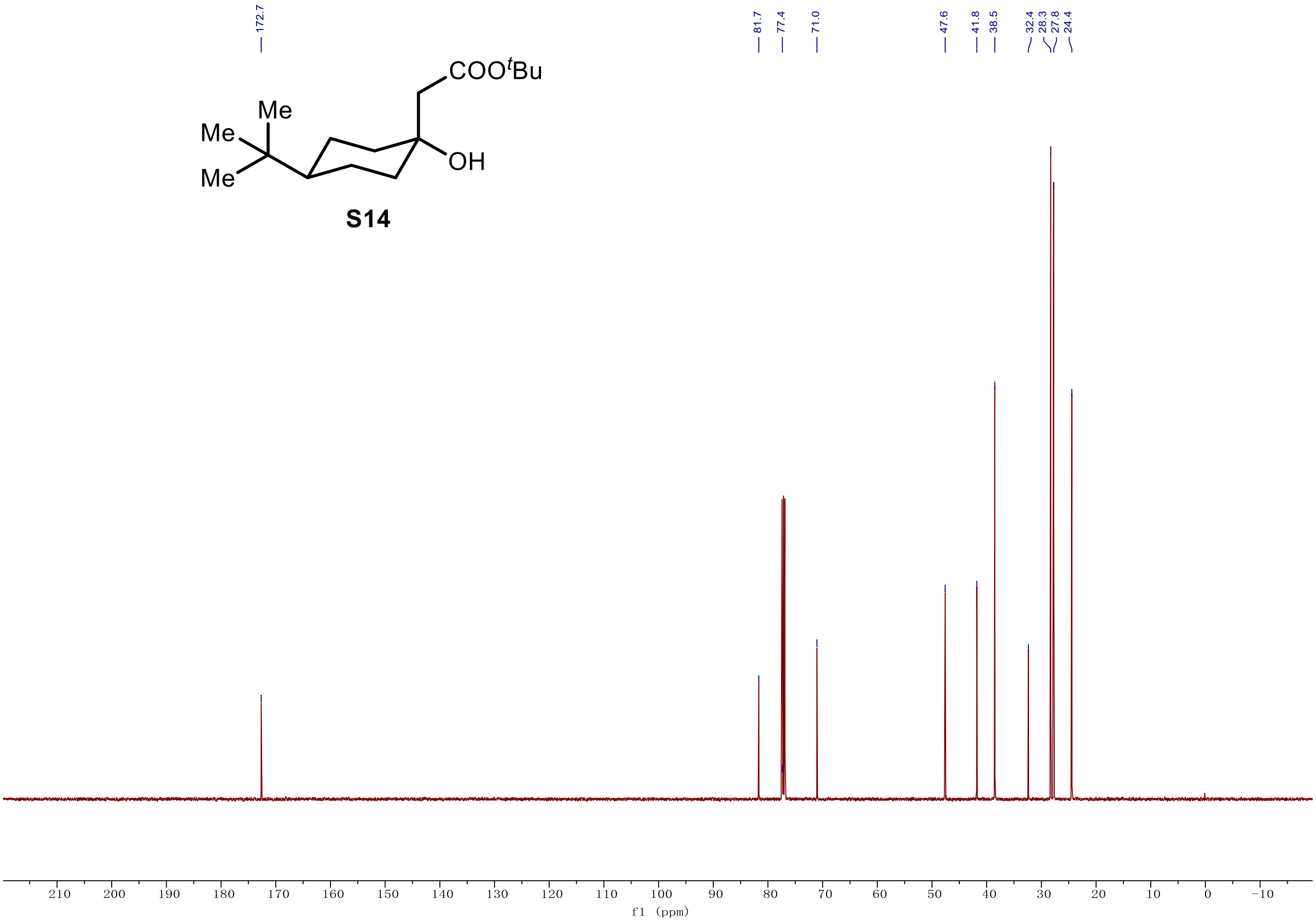

Figure S13 | ^{1}H NMR (400 MHz, $CDCl_3$) spectrum of **S15**.

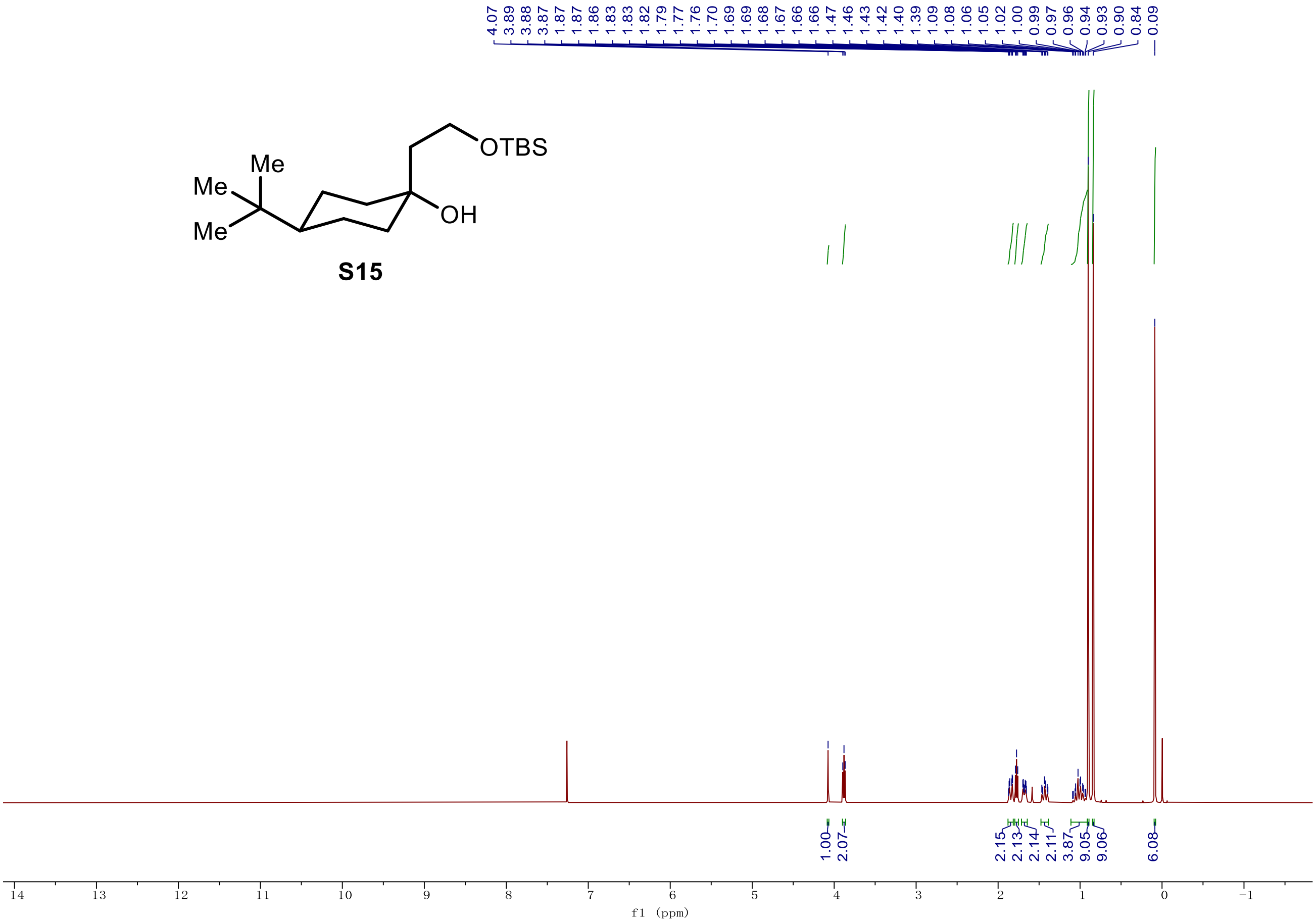

Figure S14 | ^{13}C NMR (101 MHz, $CDCl_3$) spectrum of **S15**.

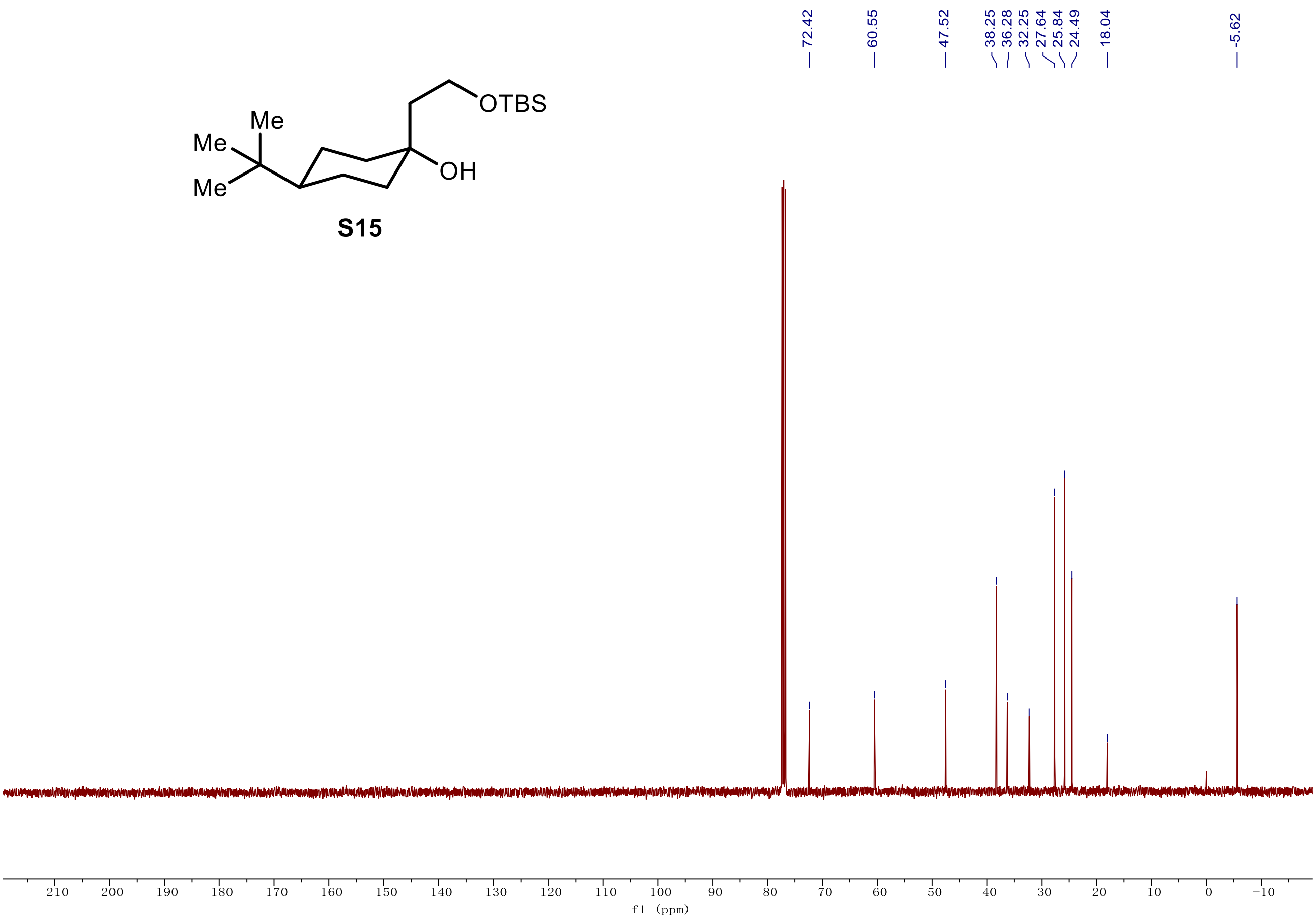

Figure S15 | ^{1}H NMR (500 MHz, $CDCl_3$) spectrum of **S16**.

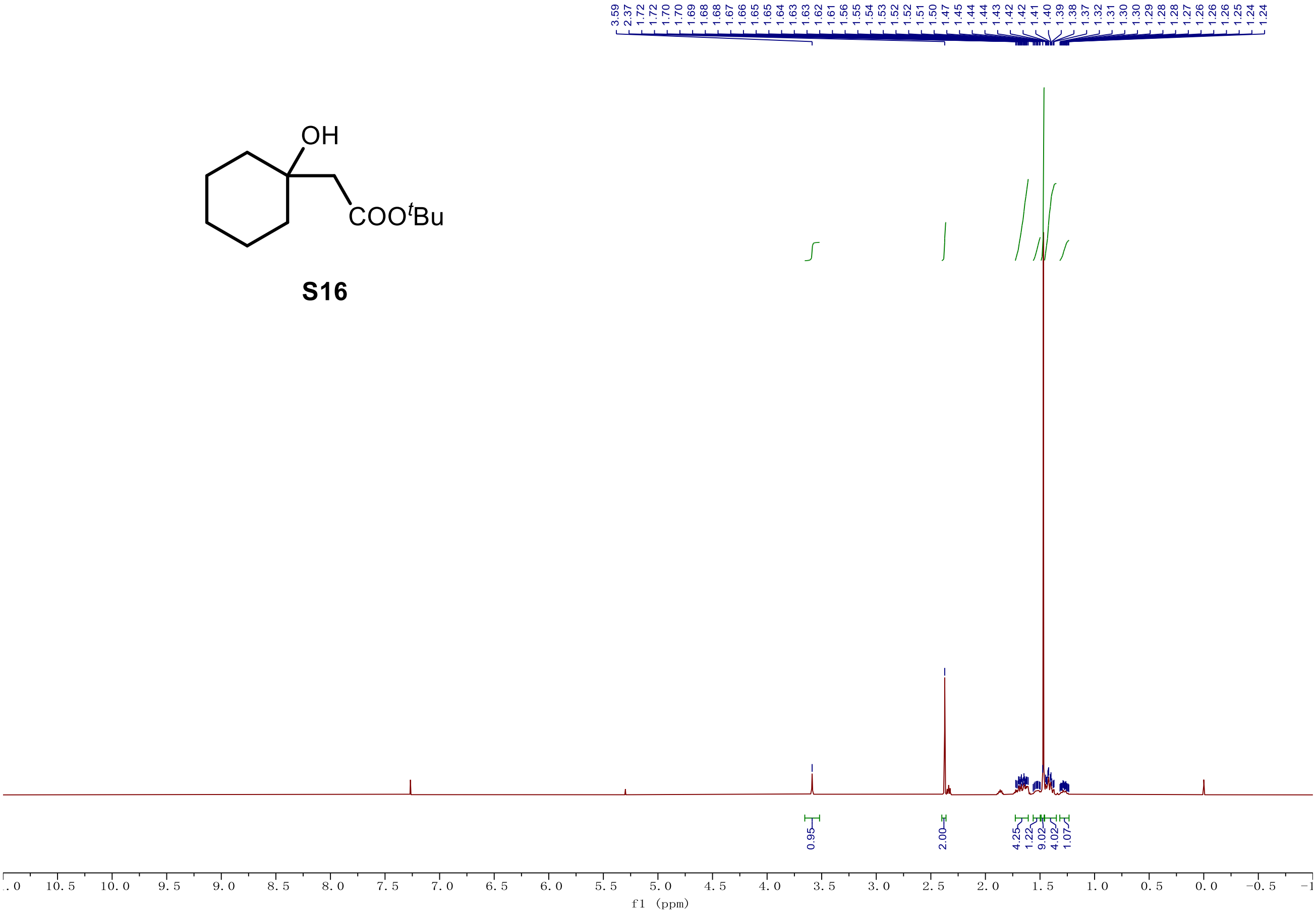

Figure S16 |^{1}H NMR (400 MHz, $CDCl_3$) spectrum of **S13**.

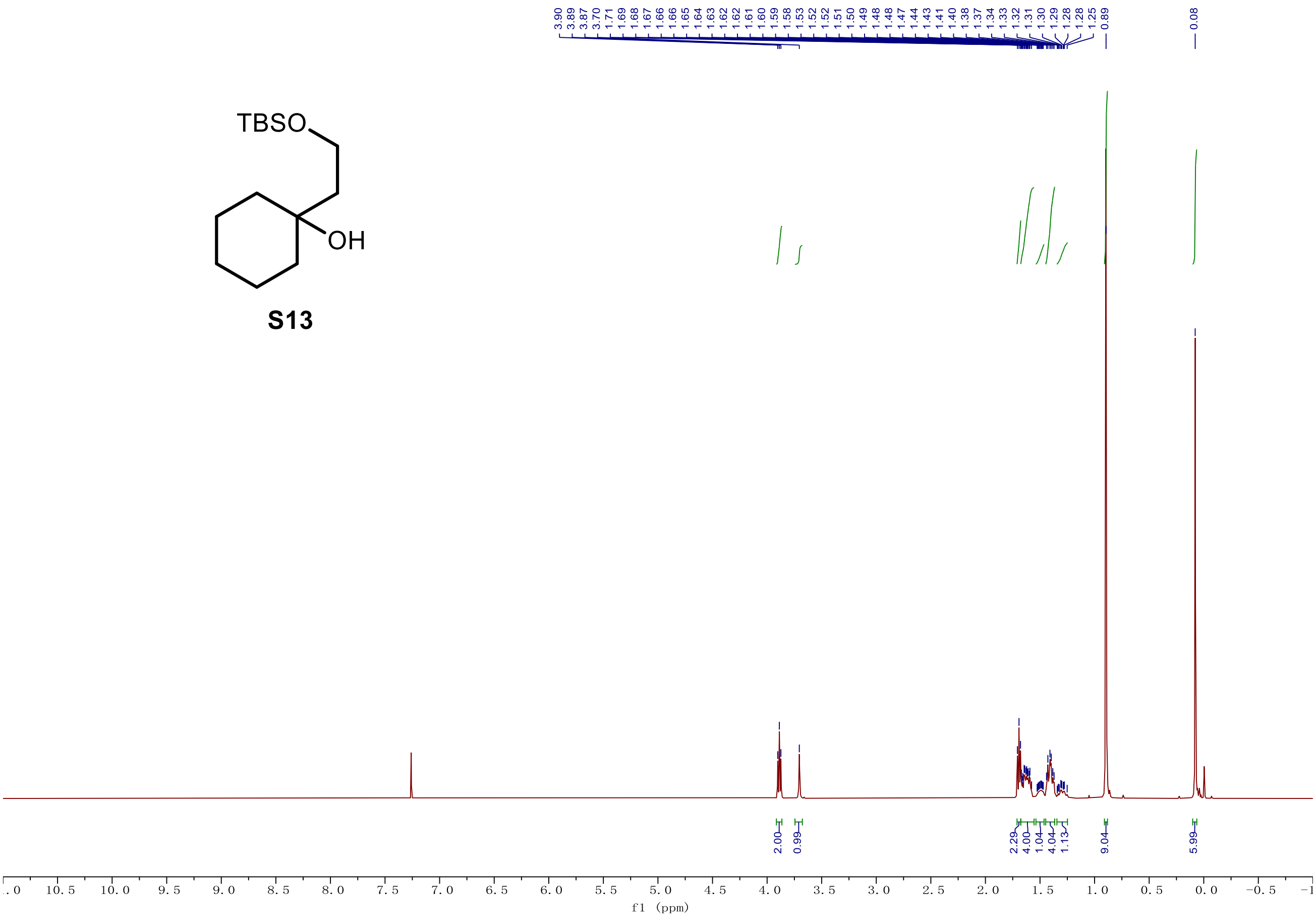

Figure S17 | ^{13}C NMR (101 MHz, $CDCl_3$) spectrum of **S13**.

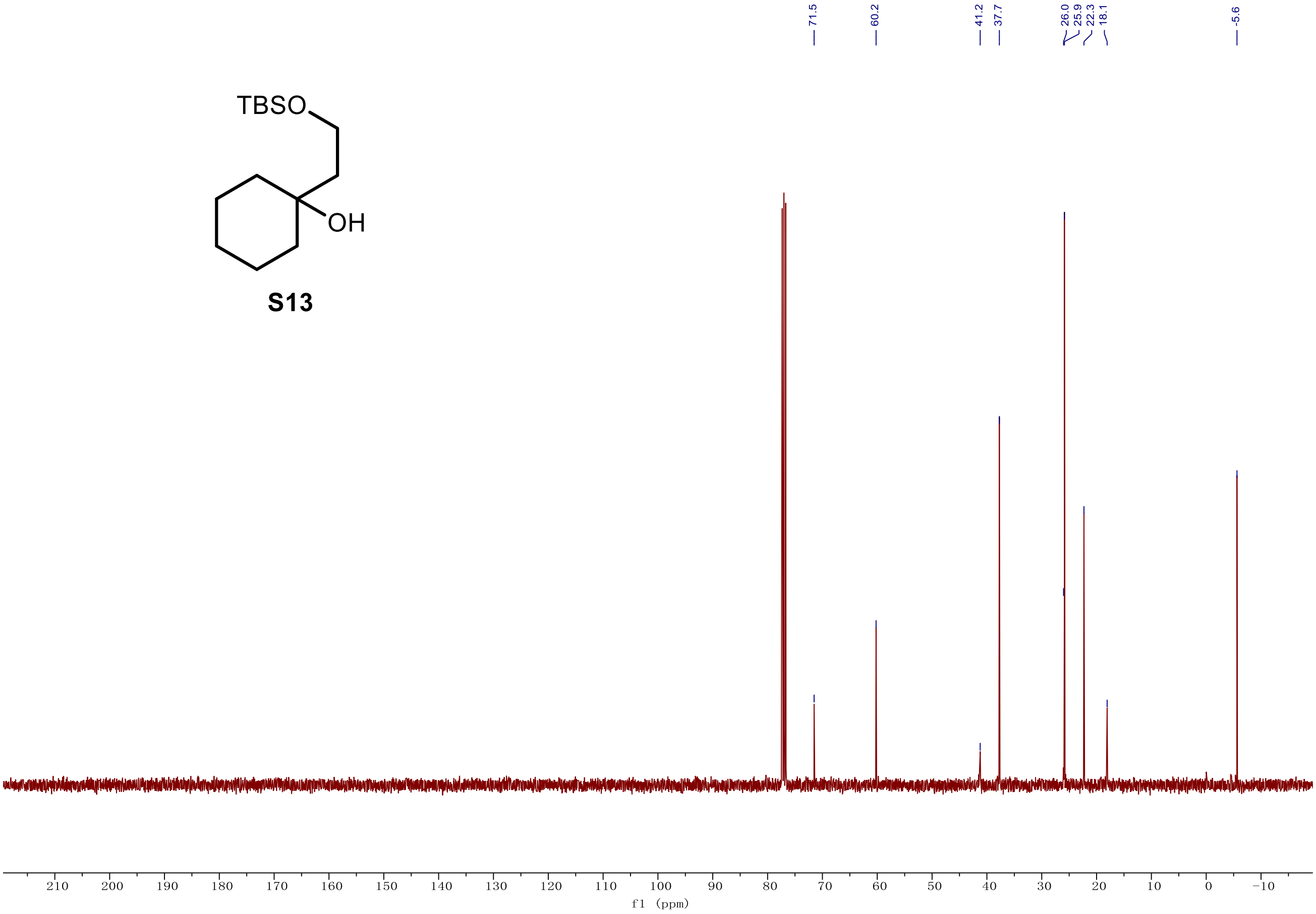

Figure S18 | ^{1}H NMR (400 MHz, $CDCl_3$) spectrum of **S17**.

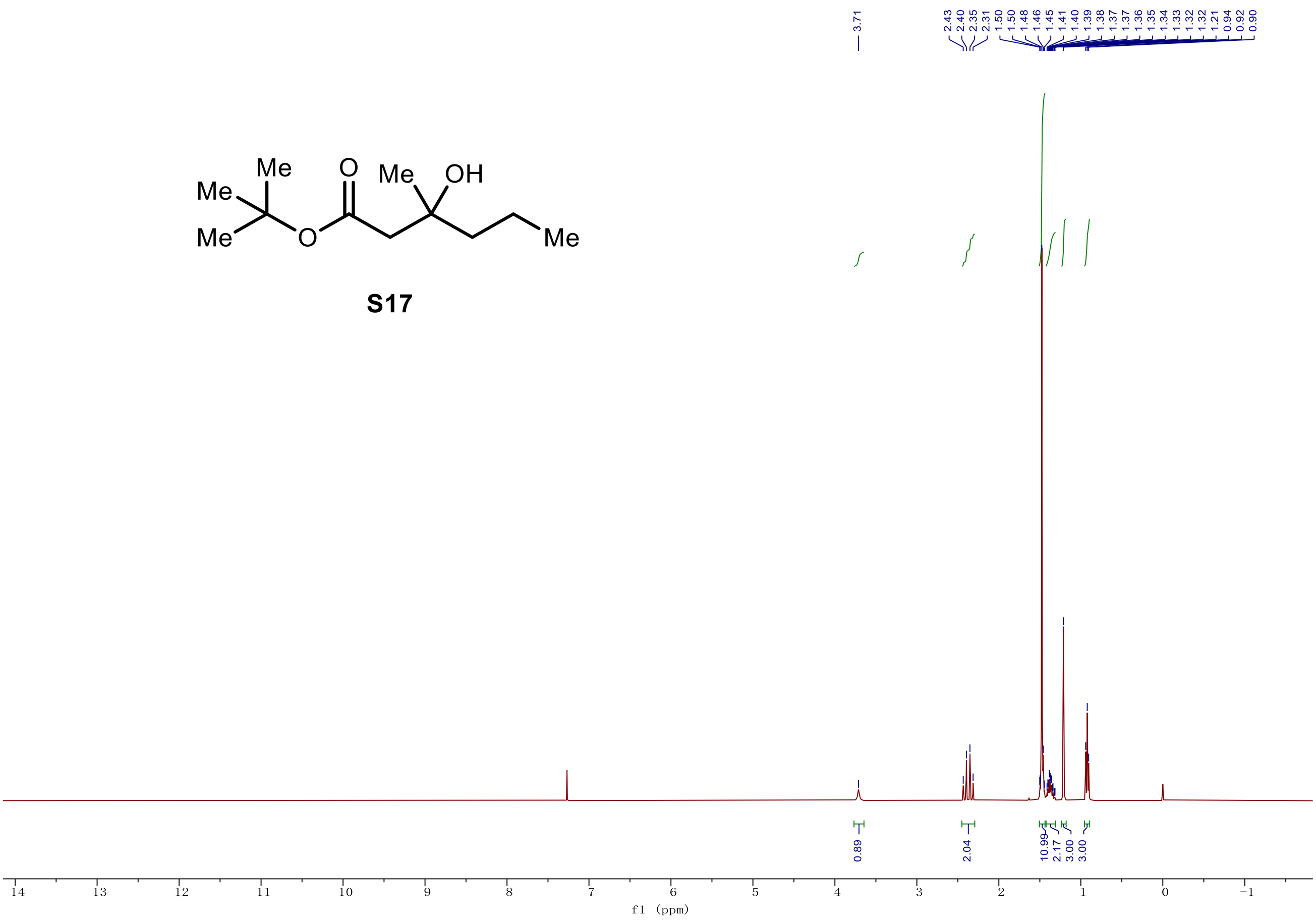

Figure S19 | ^{13}C NMR (101 MHz, $CDCl_3$) spectrum of **S17**.

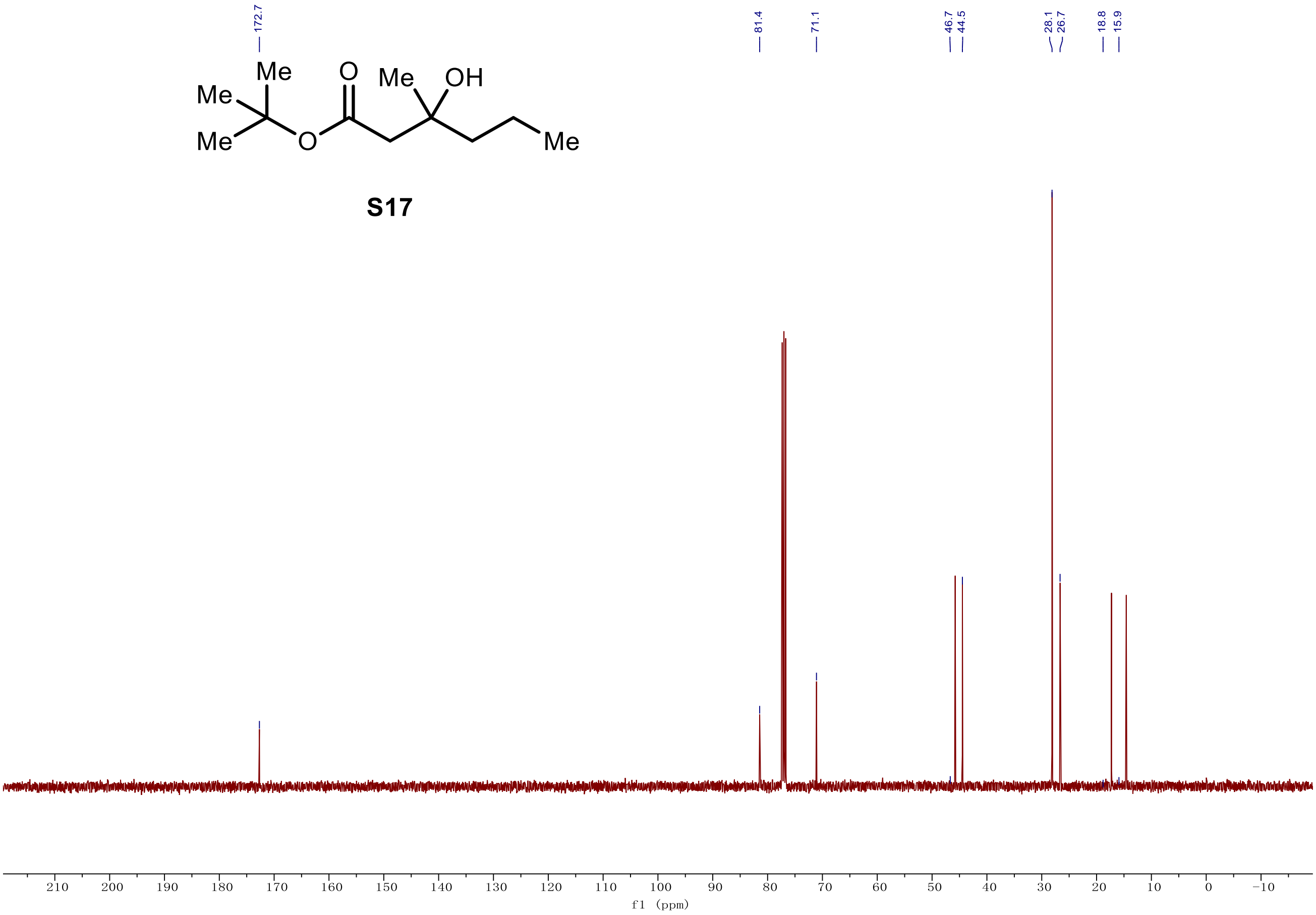

Figure S20 | ^{1}H NMR (500 MHz, $CDCl_3$) spectrum of **S18**.

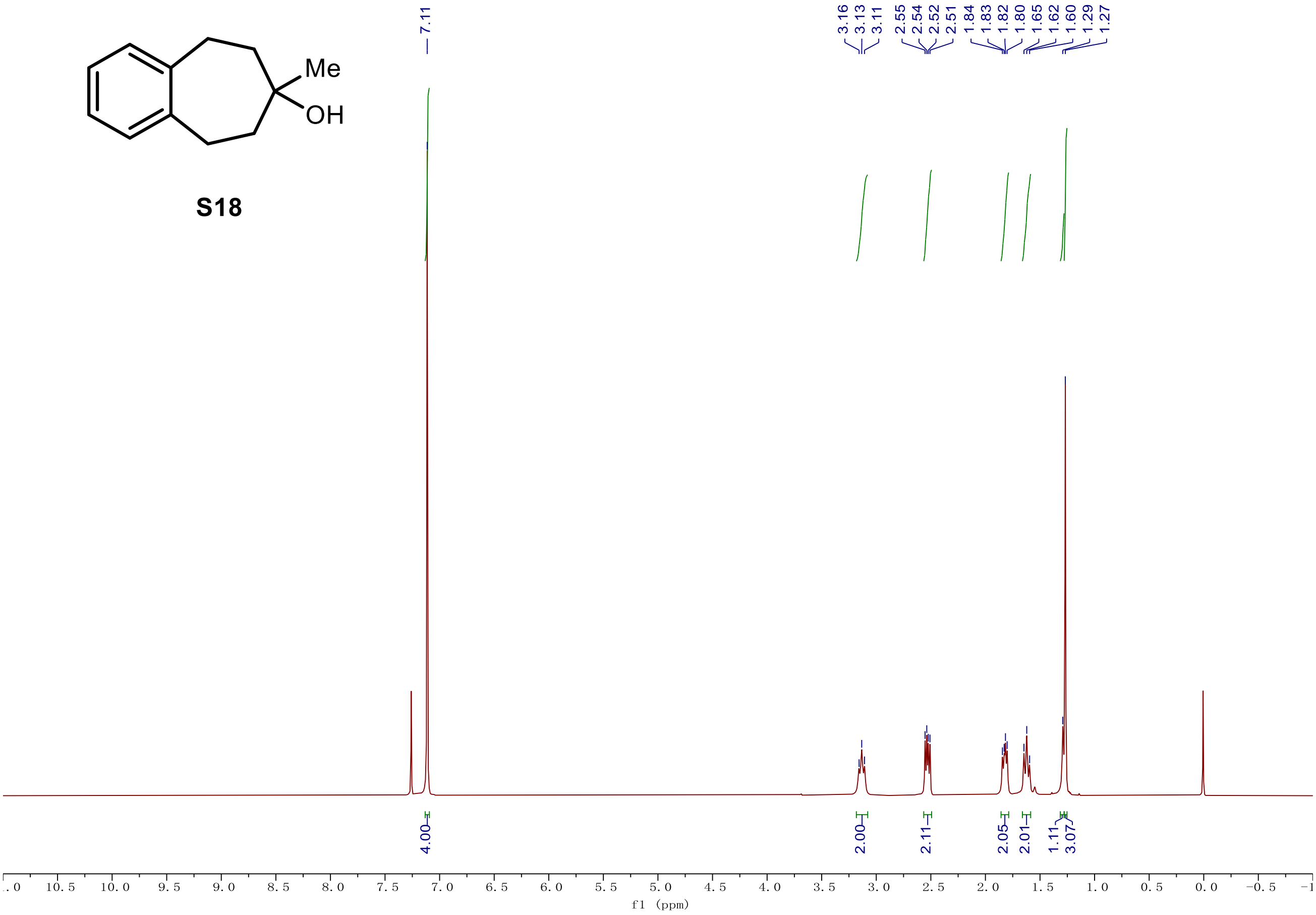

Figure S21 | ^{13}C NMR (126 MHz, $CDCl_3$) spectrum of **S18**.

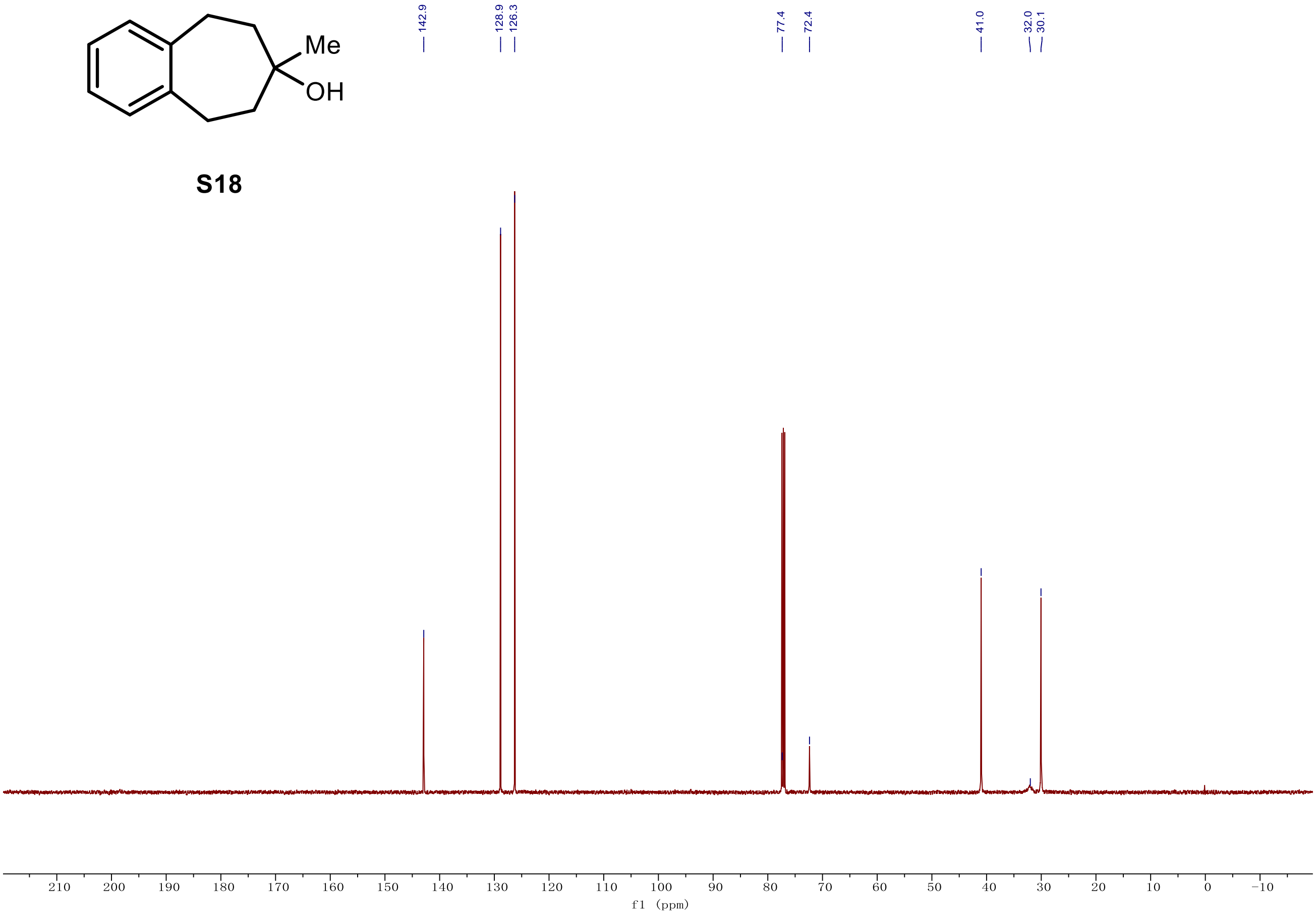

S18

Figure S22 | ^{1}H NMR (400 MHz, $CDCl_3$) spectrum of **S19**.

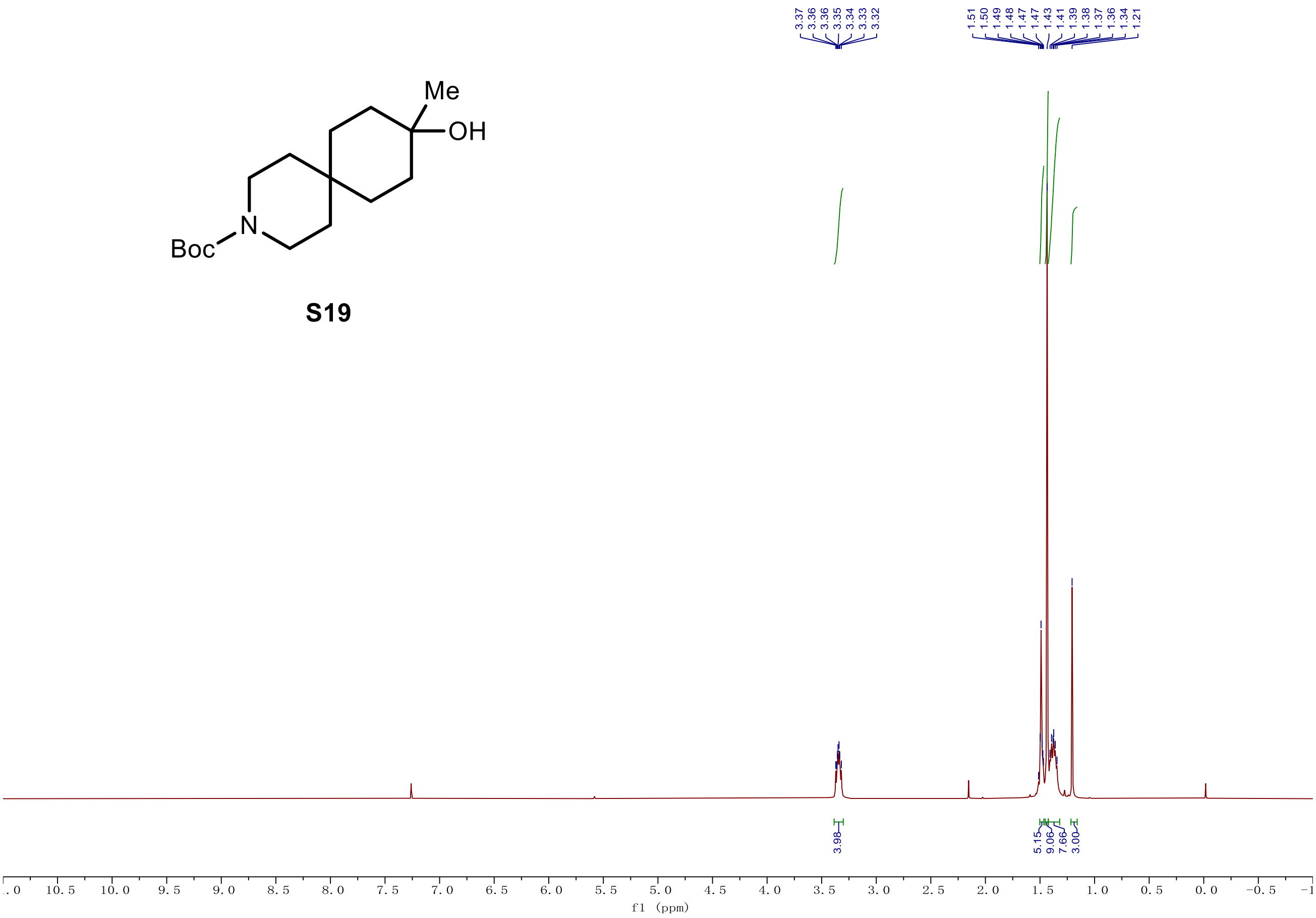

S19

Figure S23 | ^{13}C NMR (101 MHz, $CDCl_3$) spectrum of **S19**.

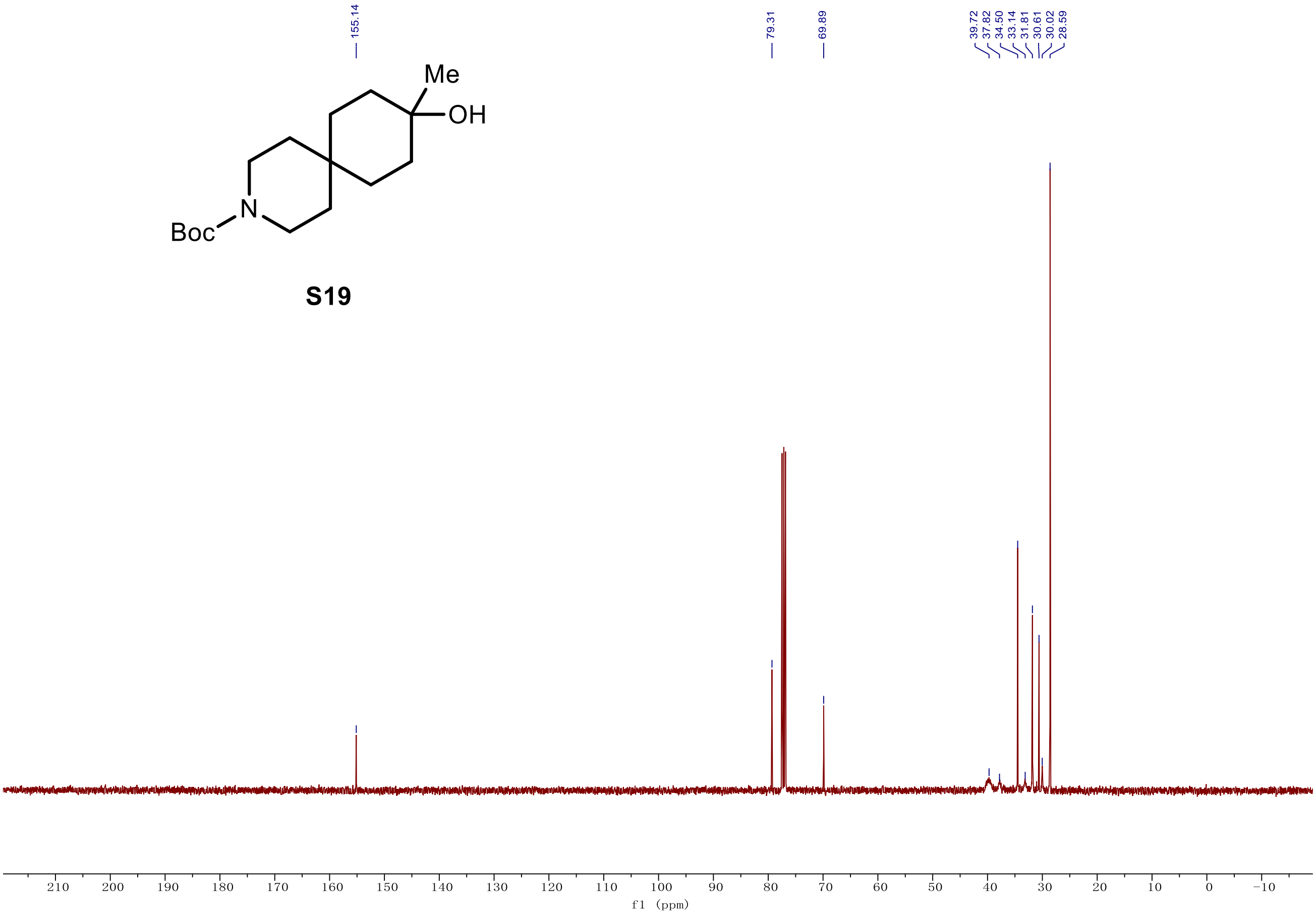

Figure S24 | ^{1}H NMR (600 MHz, $CDCl_3$) spectrum of **1c**.

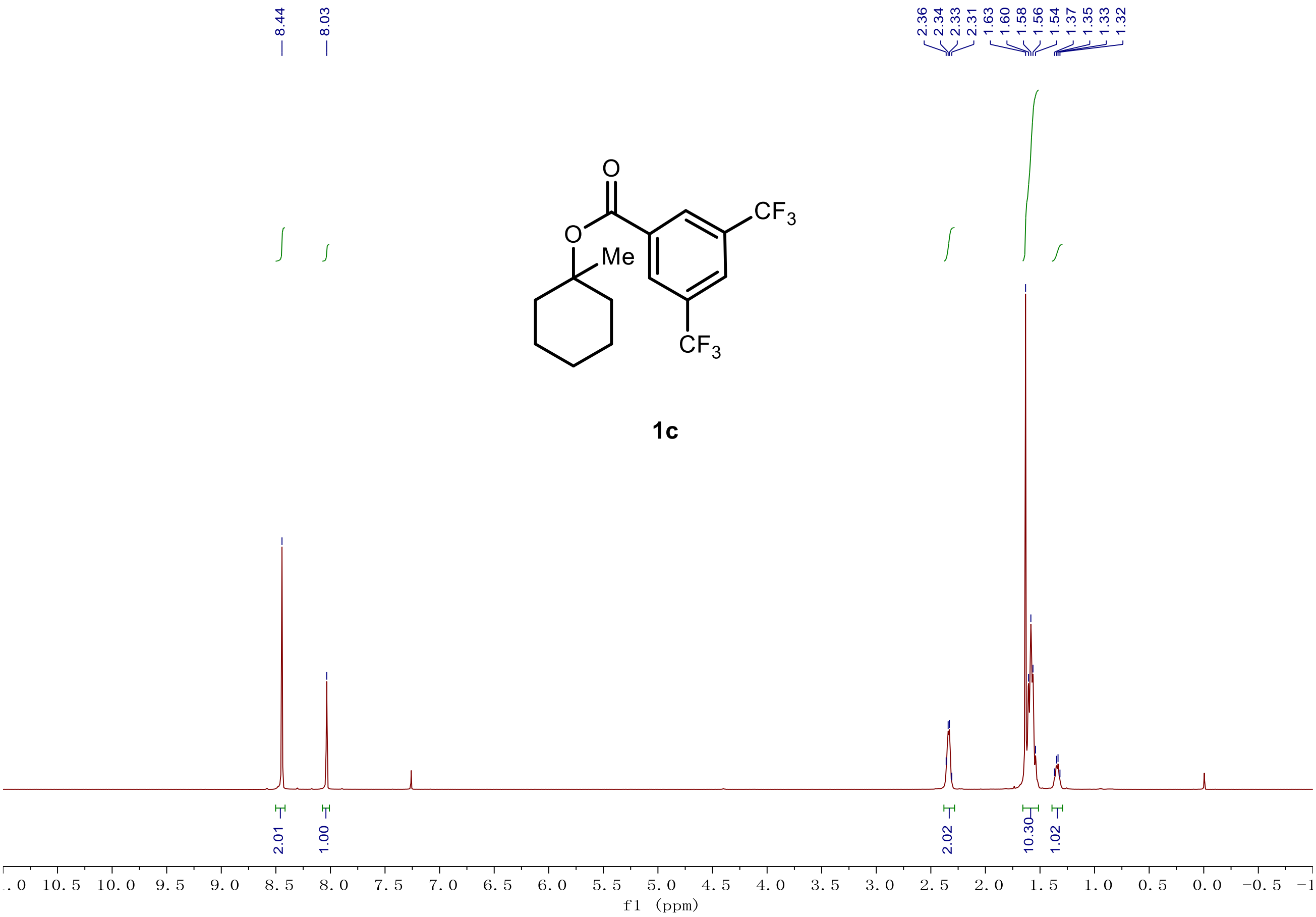

Figure S25 | ^{13}C NMR (151 MHz, $CDCl_3$) spectrum of **1c**.

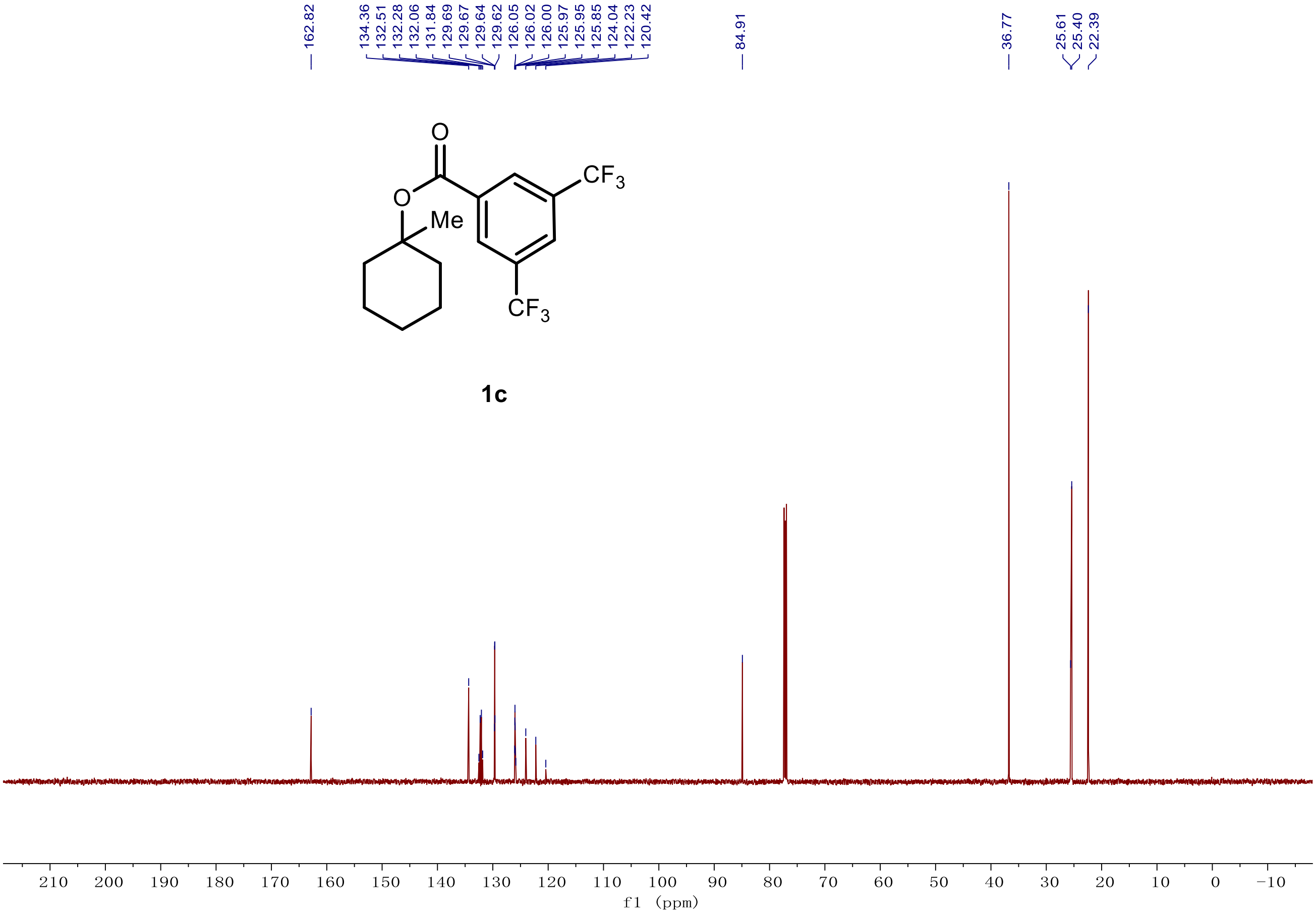

Figure S26 | ^{19}F NMR (565 MHz, $CDCl_3$) spectrum of **1c**.

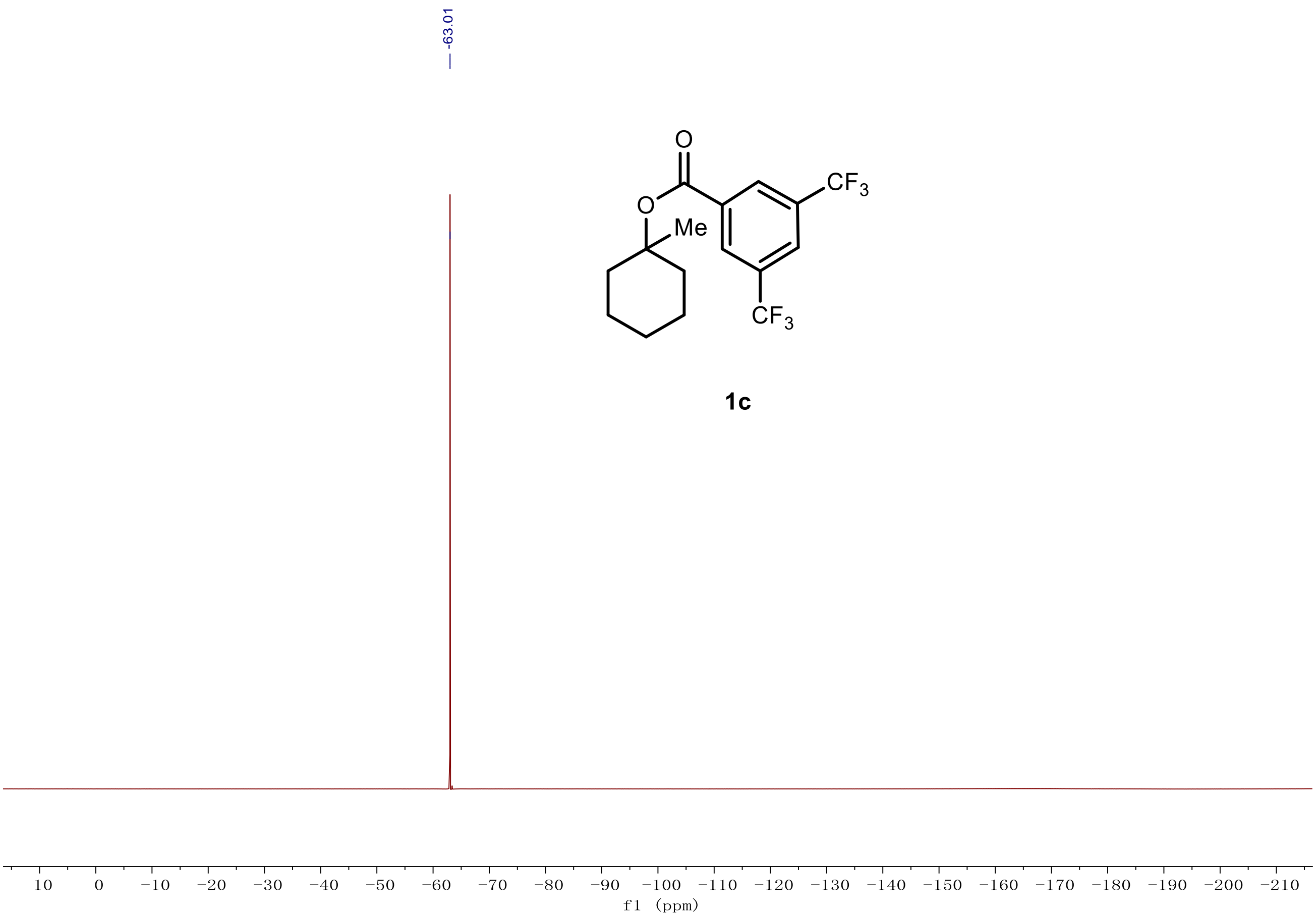

Figure S27 | ^{1}H NMR (400 MHz, $CDCl_3$) spectrum of **1d**.

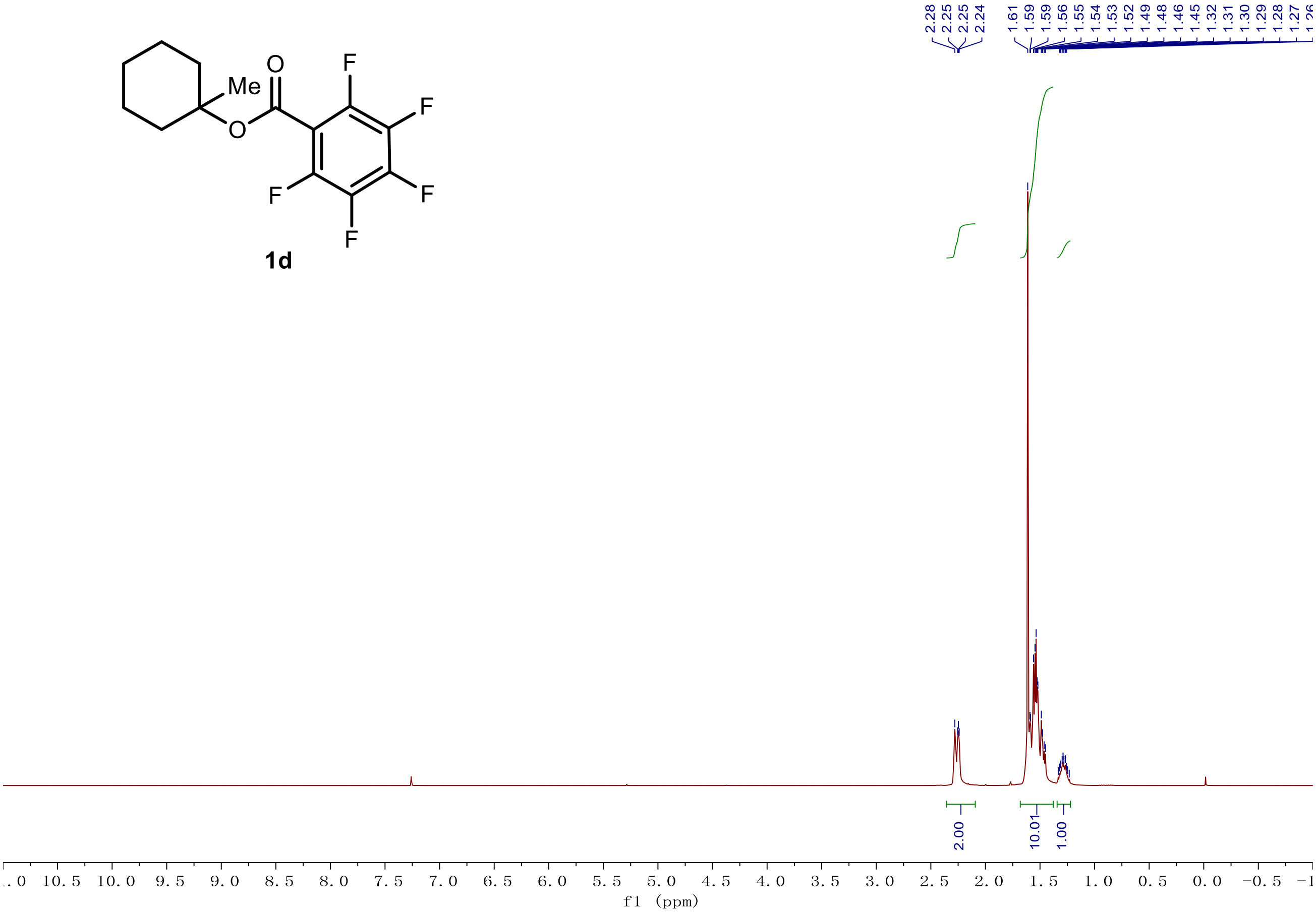

Figure S28 | ^{13}C NMR (101 MHz, $CDCl_3$) spectrum of **1d**.

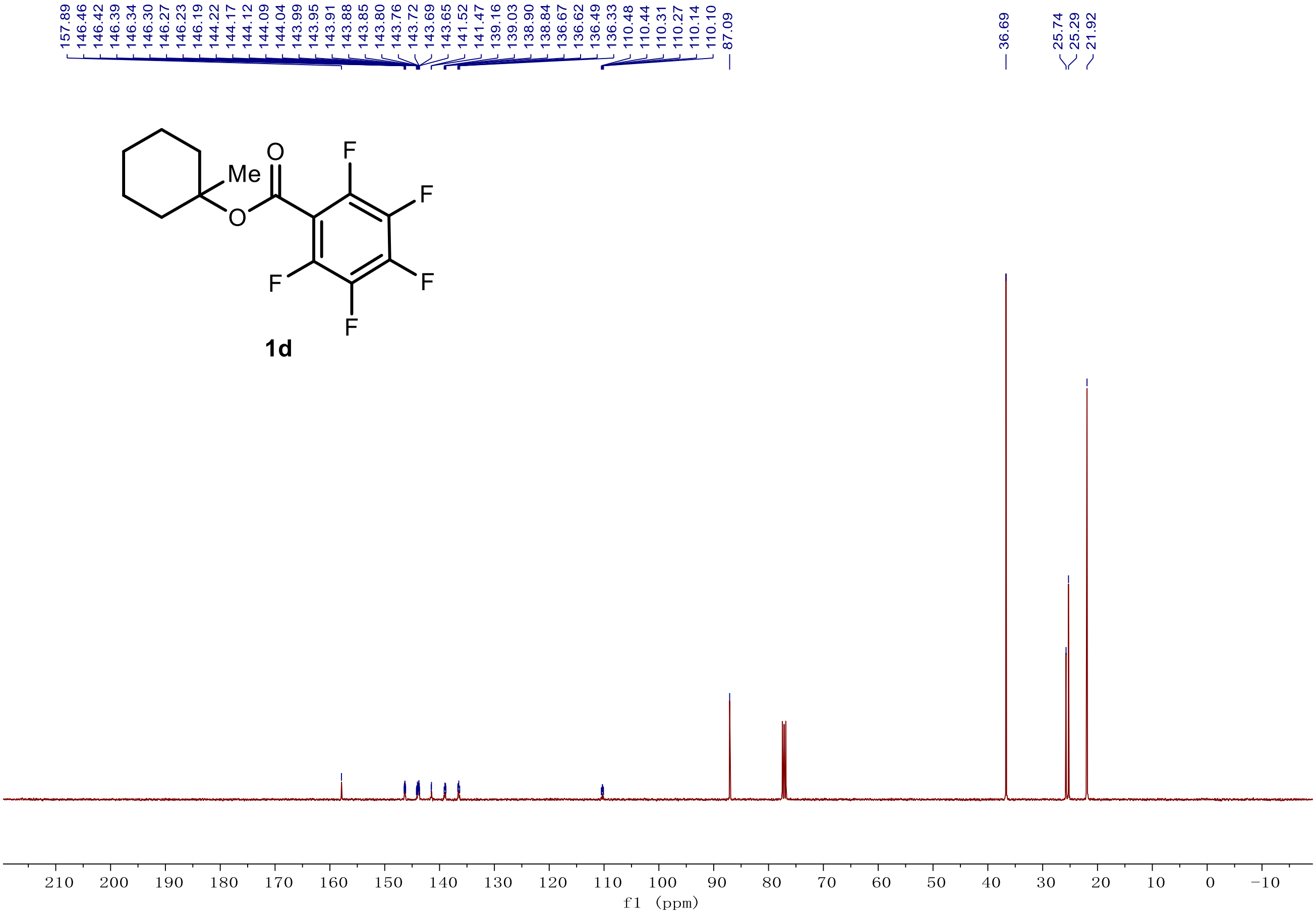

Figure S29 | ^{19}F NMR (471 MHz, $CDCl_3$) spectrum of **1d**.

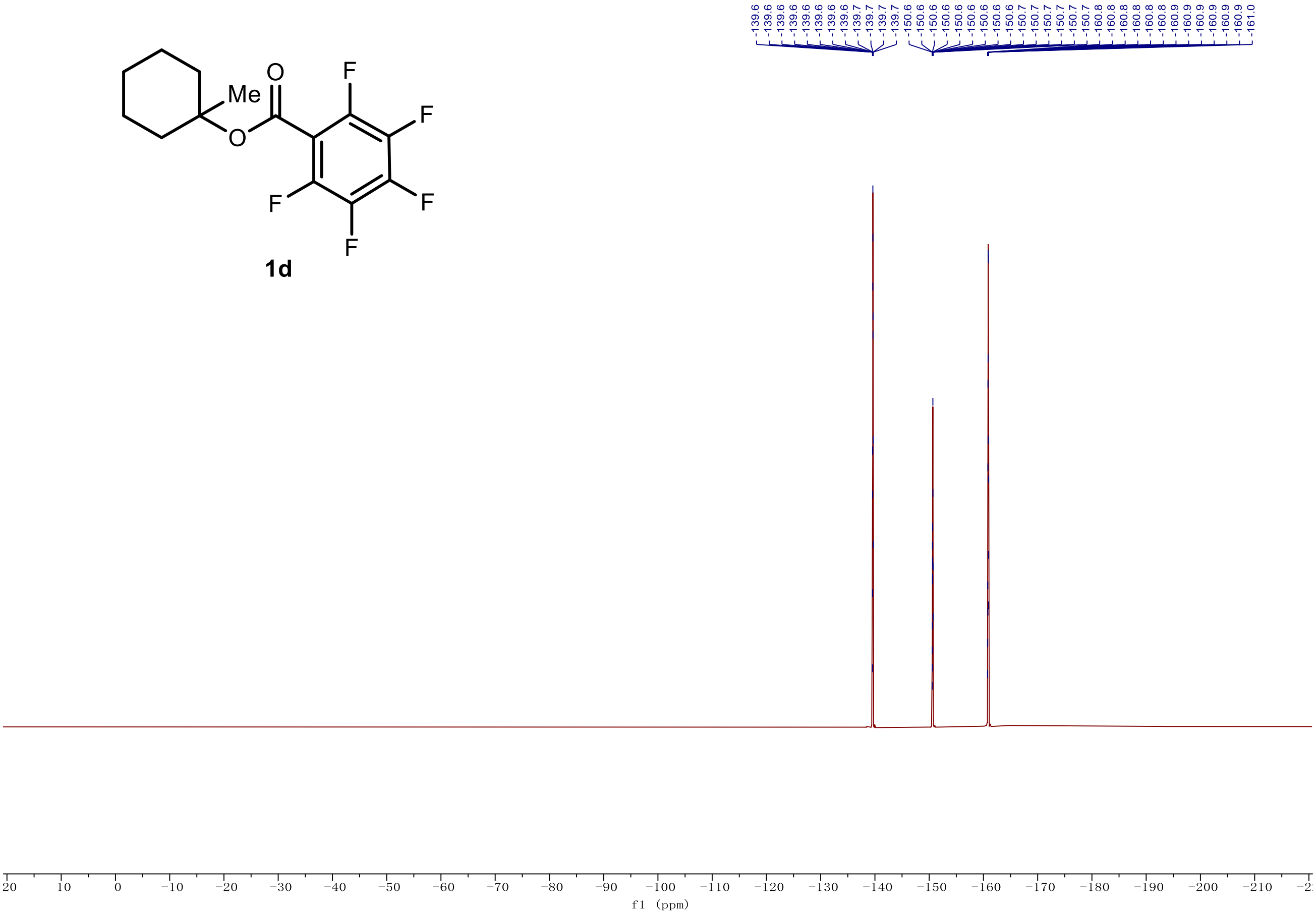

Figure S30 | ^{1}H NMR (400 MHz, $CDCl_3$) spectrum of **1e**.

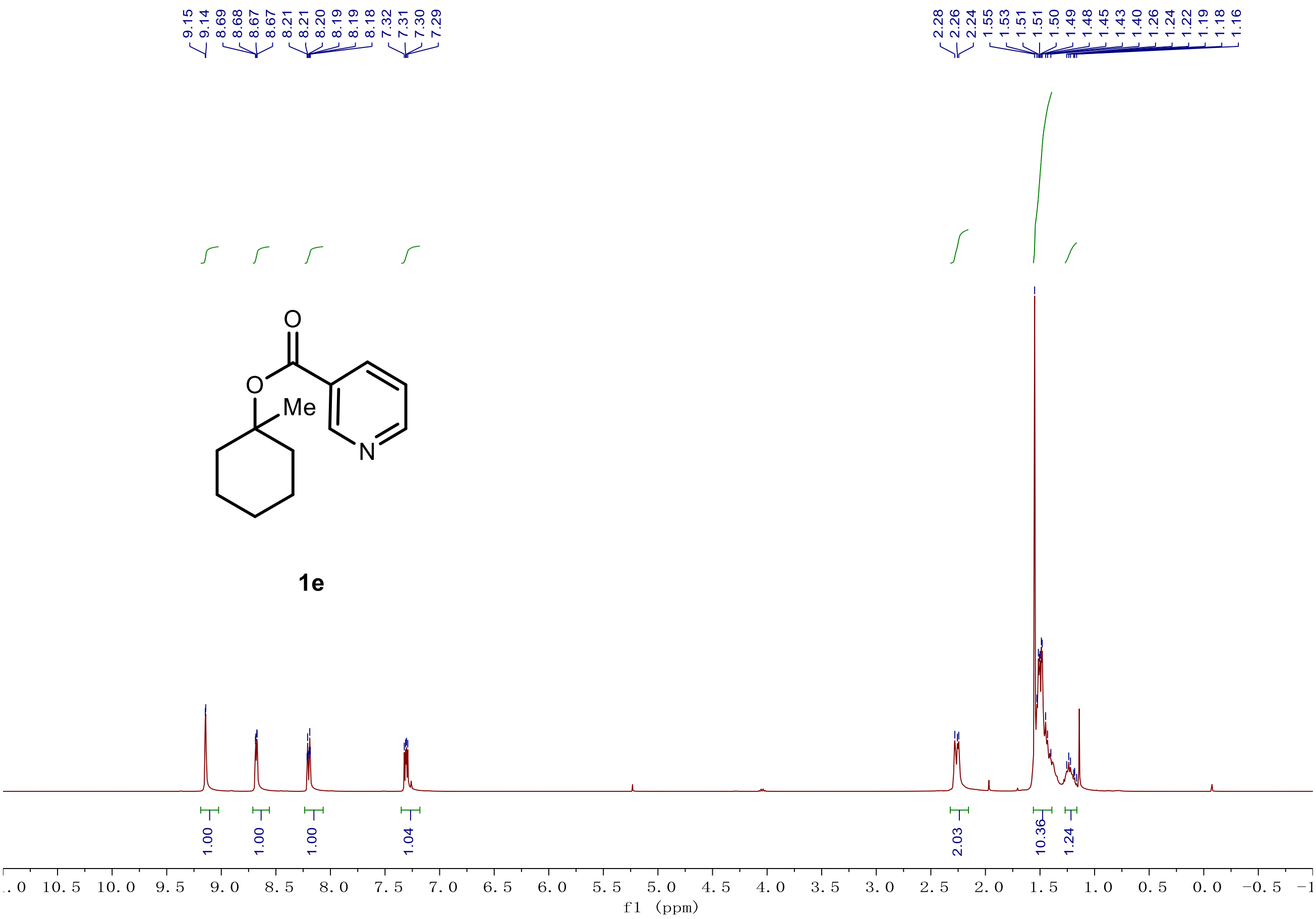

Figure S31 | ^{13}C NMR (101 MHz, $CDCl_3$) spectrum of **1e**.

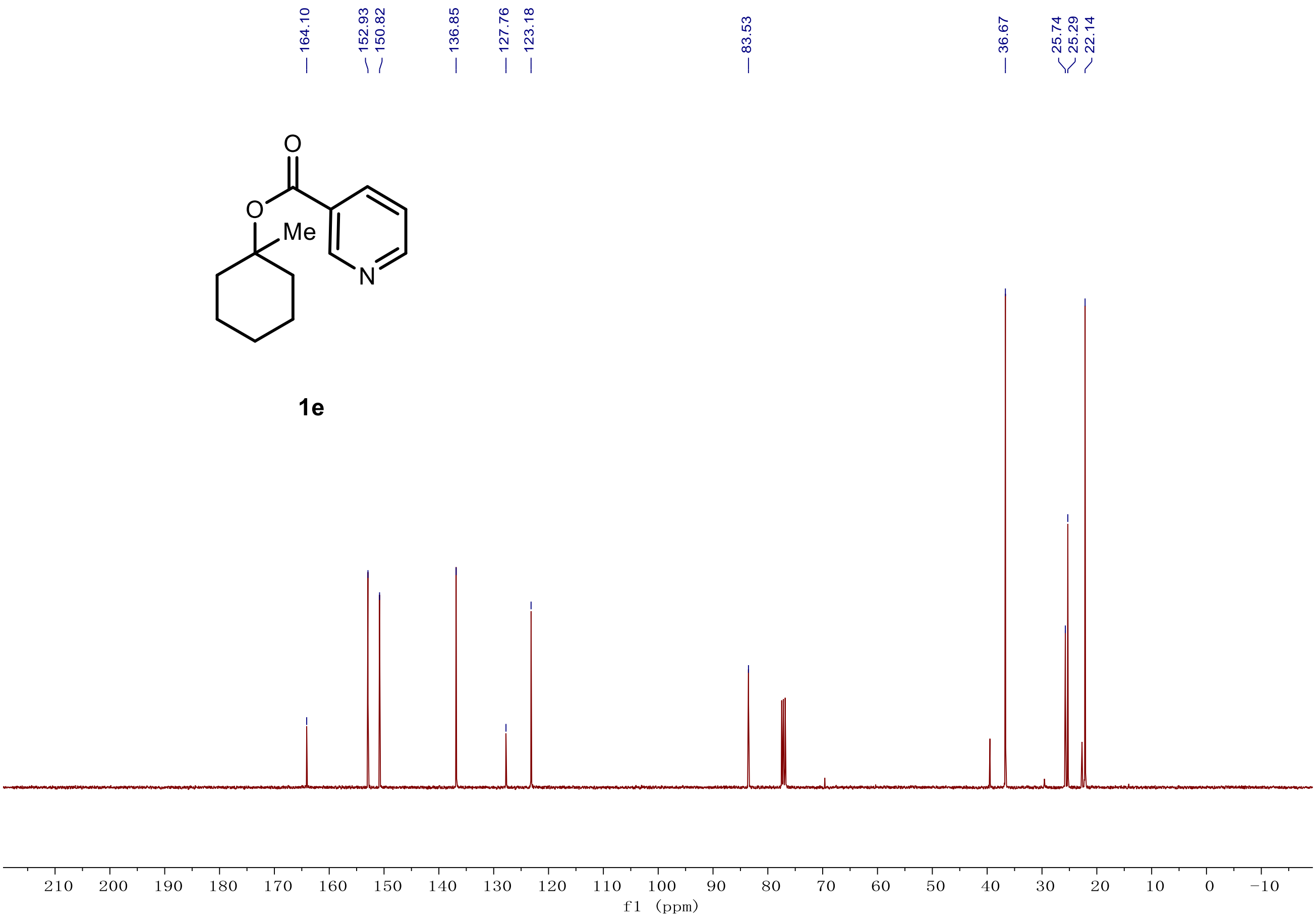

Figure S32 | ^{1}H NMR (400 MHz, $CDCl_3$) spectrum of **1h**.

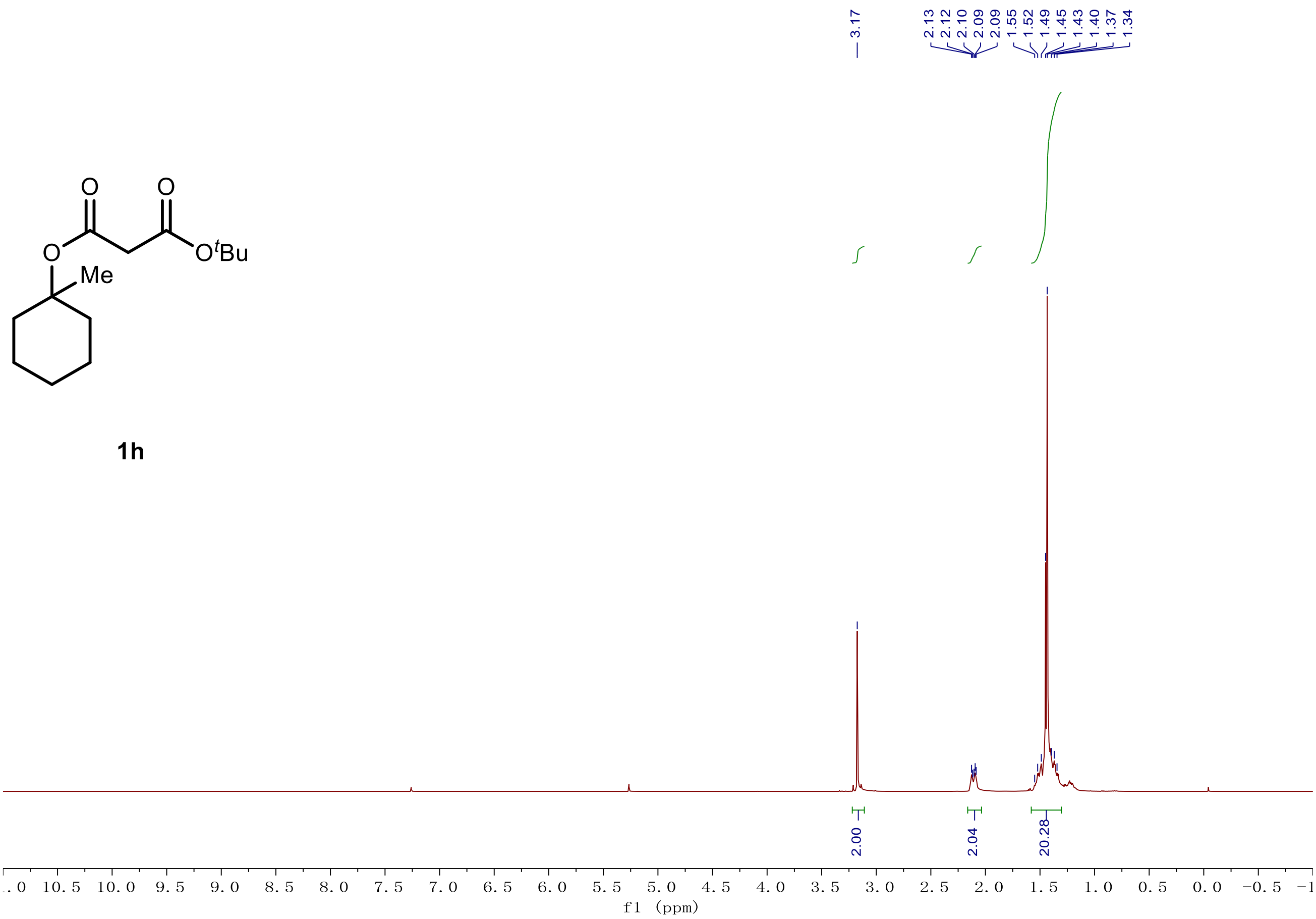

Figure S33 | ^{13}C NMR (101 MHz, $CDCl_3$) spectrum of **1h**.

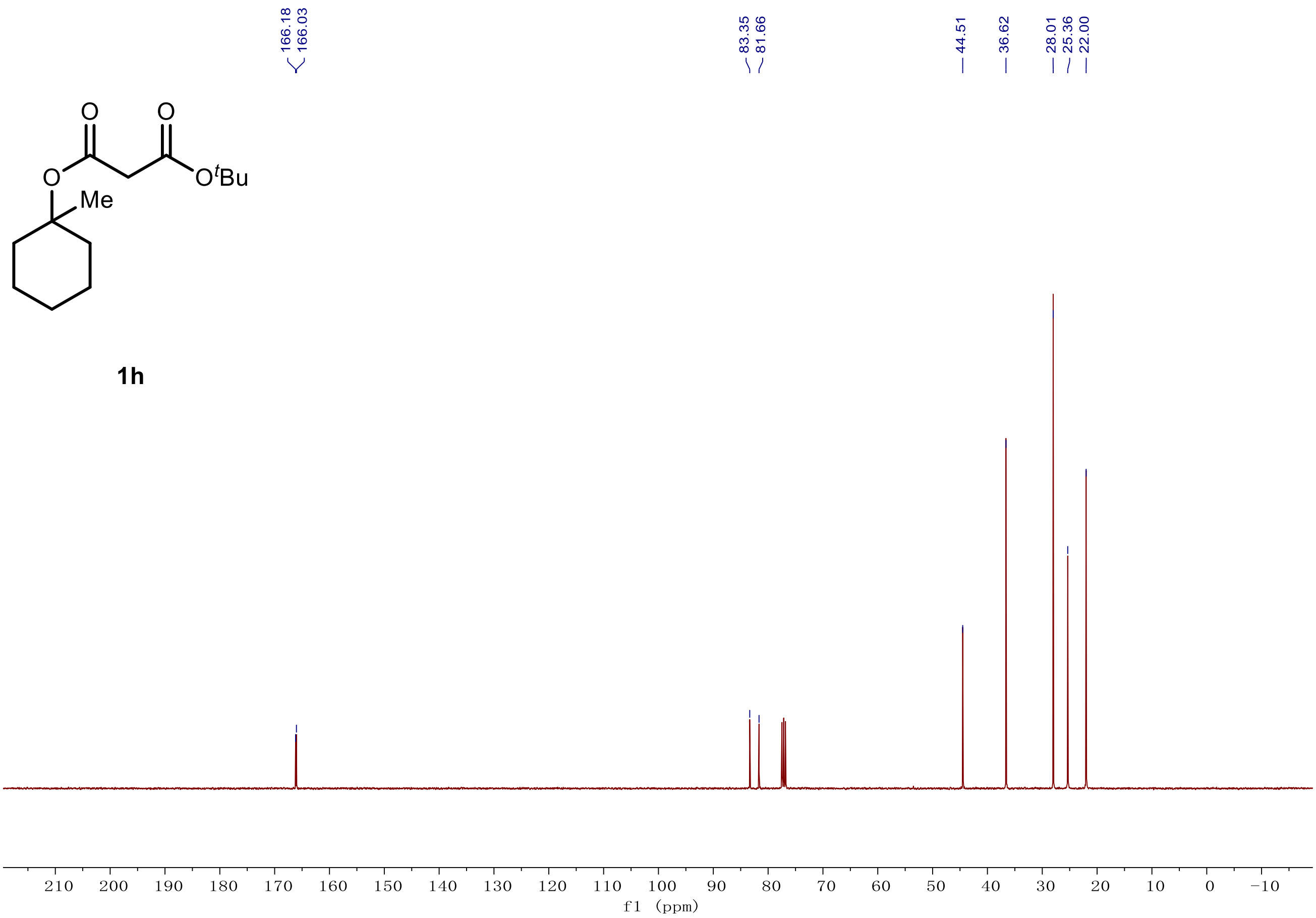

Figure S34 | ^{1}H NMR (400 MHz, $CDCl_3$) spectrum of **1i**.

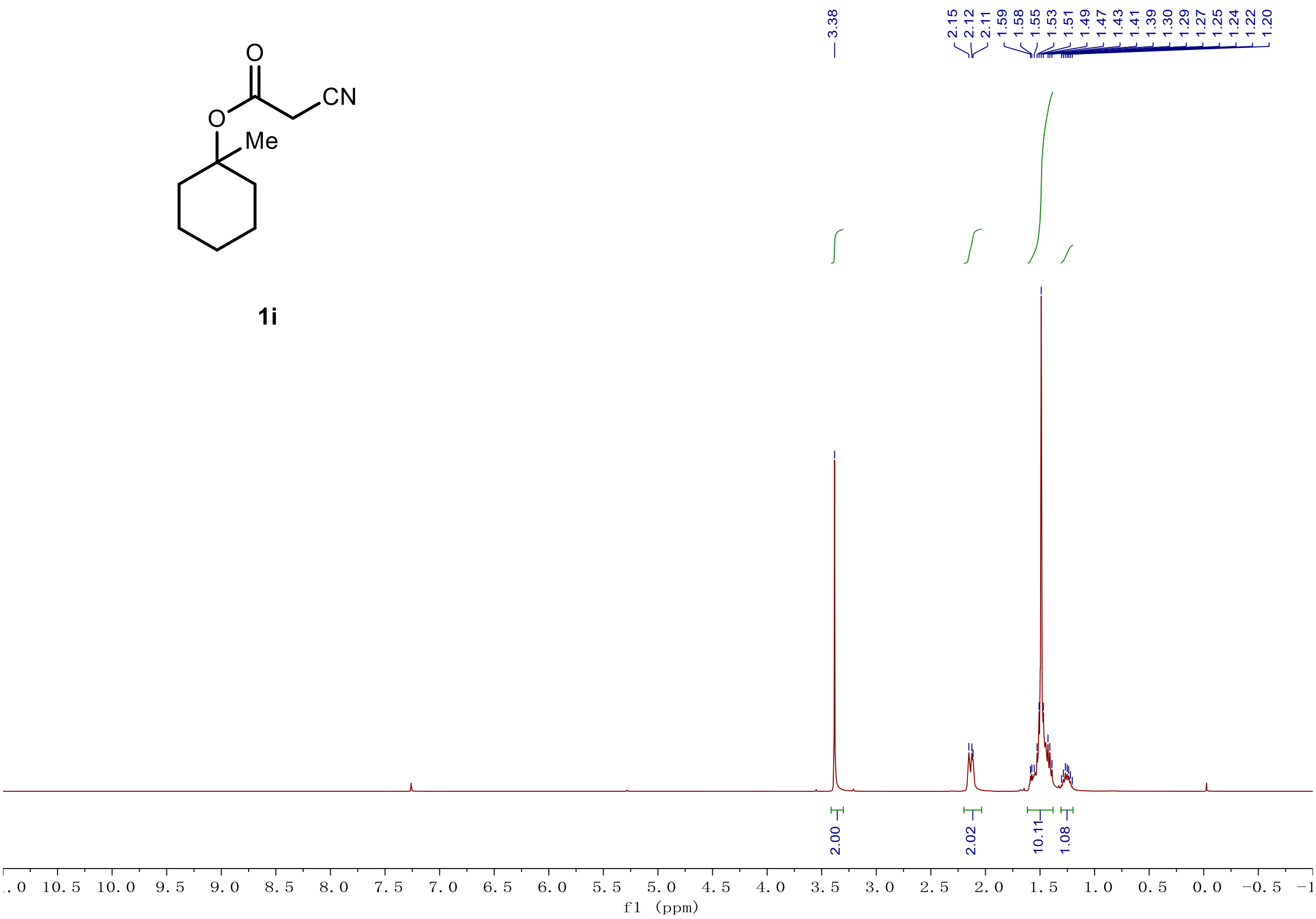

Figure S35 | ^{13}C NMR (101 MHz, $CDCl_3$) spectrum of **1i**.

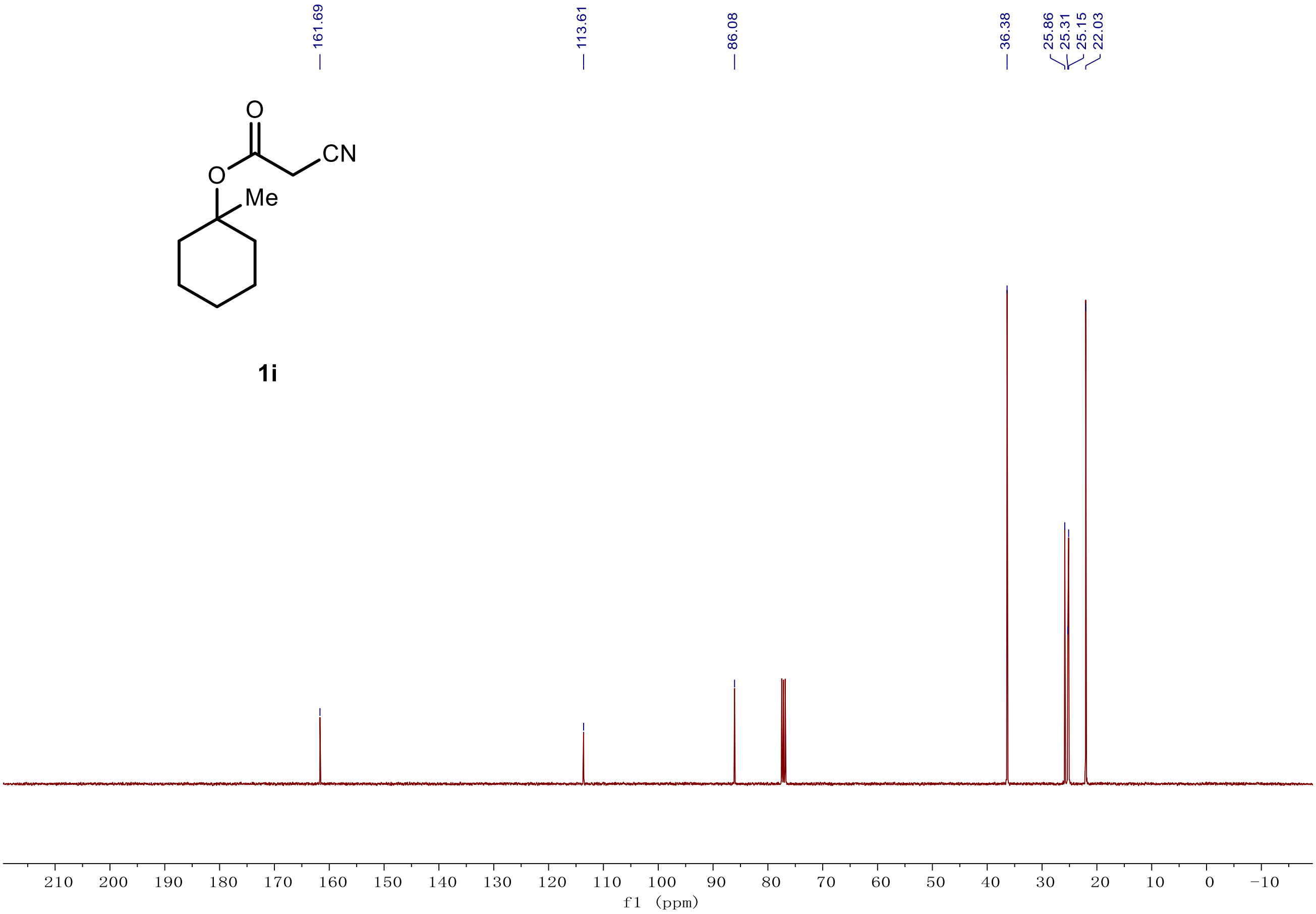

Figure S36 | ^{1}H NMR (400 MHz, $CDCl_3$) spectrum of **1j**.

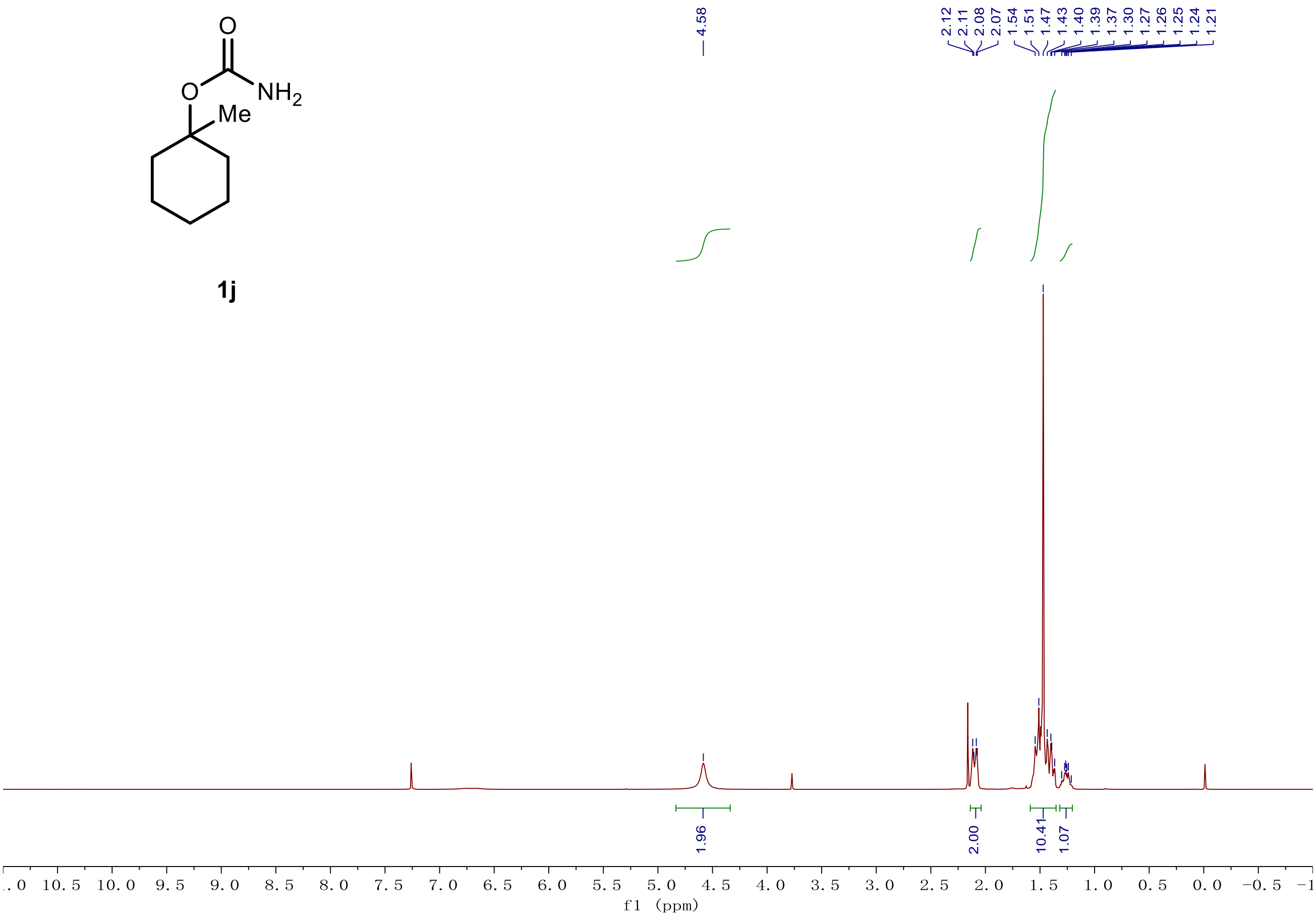

Figure S37 | ^{13}C NMR (101 MHz, $CDCl_3$) spectrum of **1j**.

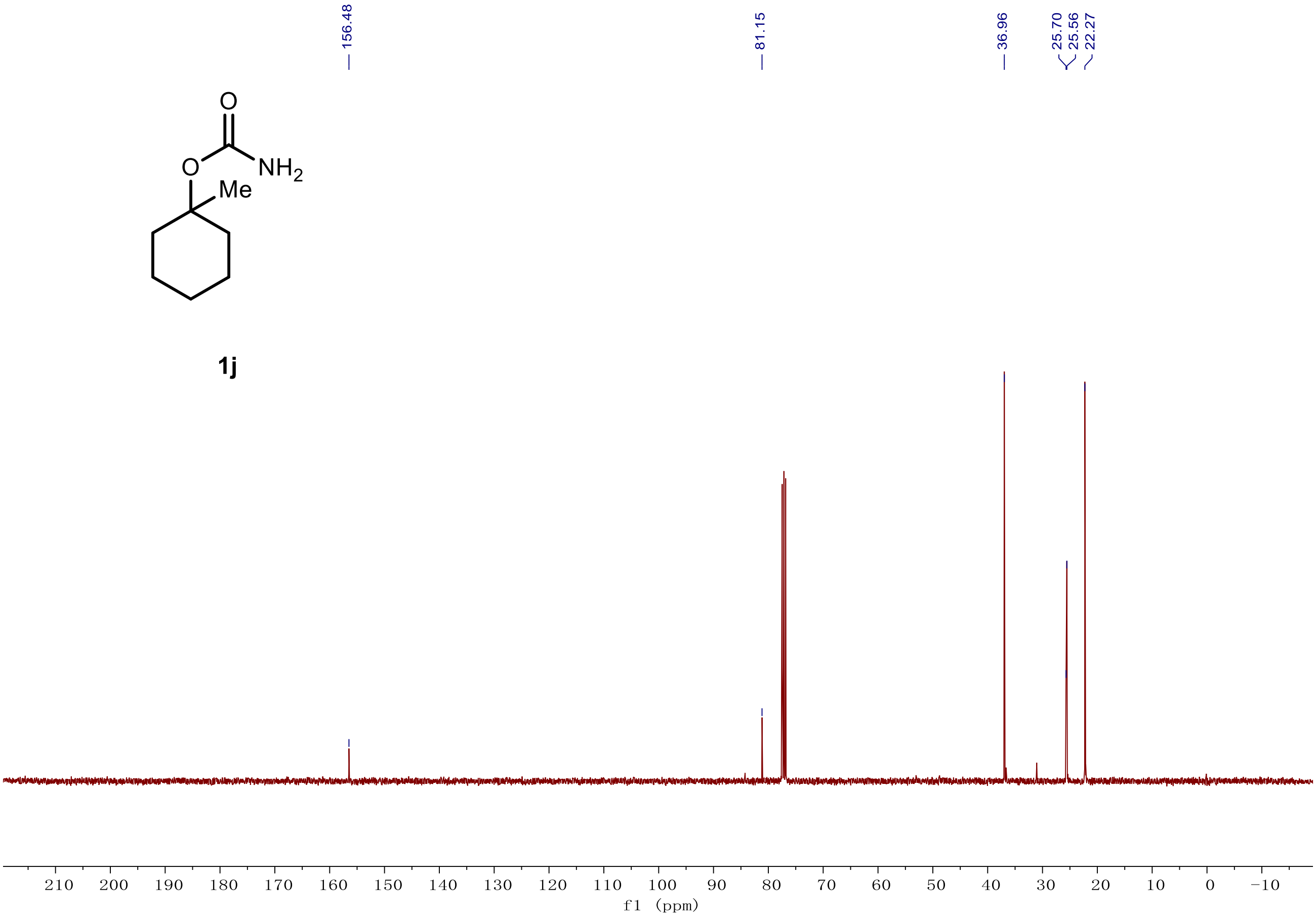

Figure S38 | ^{1}H NMR (500 MHz, $CDCl_3$) spectrum of **1k**.

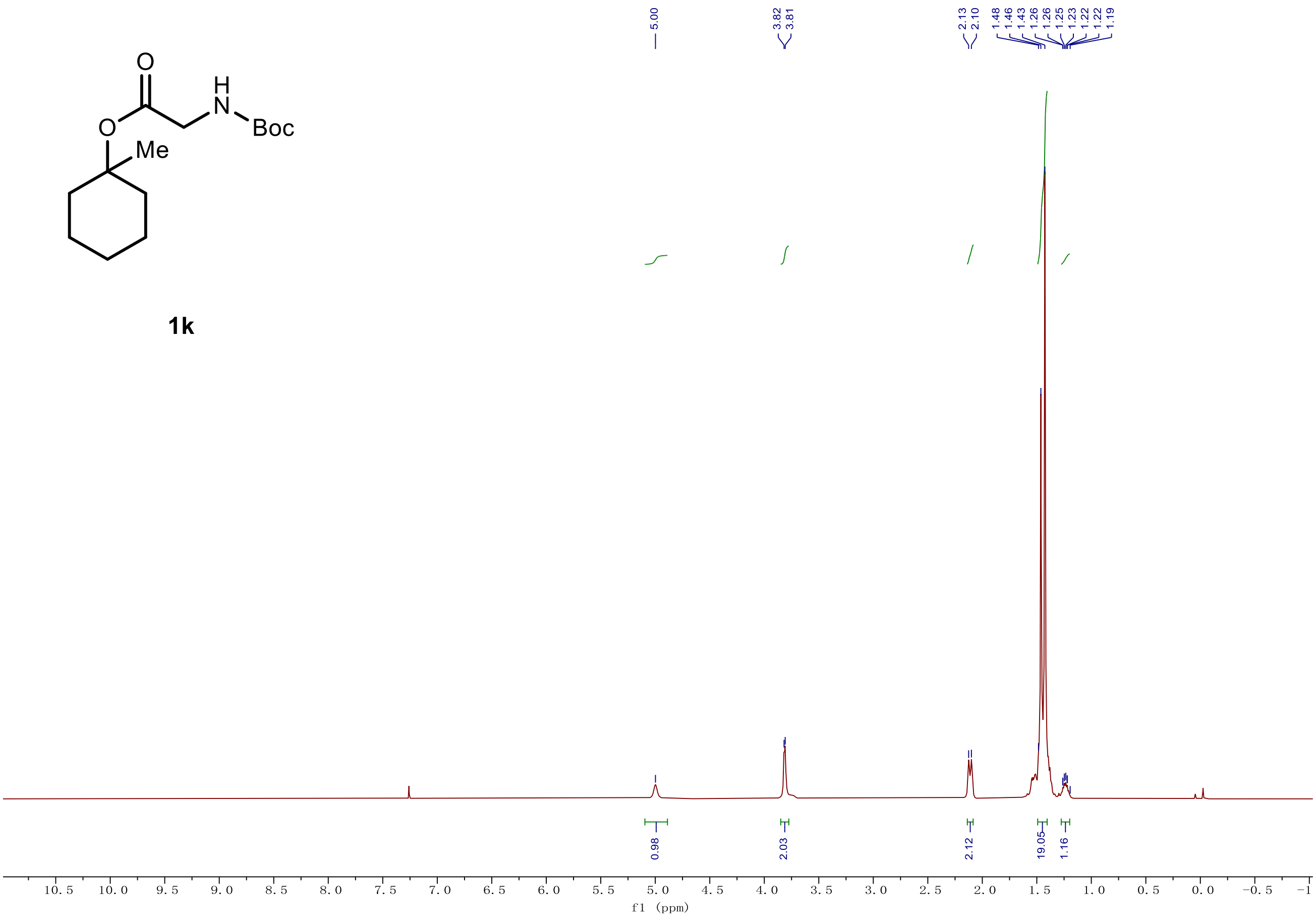

Figure S39 | ^{13}C NMR (126 MHz, $CDCl_3$) spectrum of **1k**.

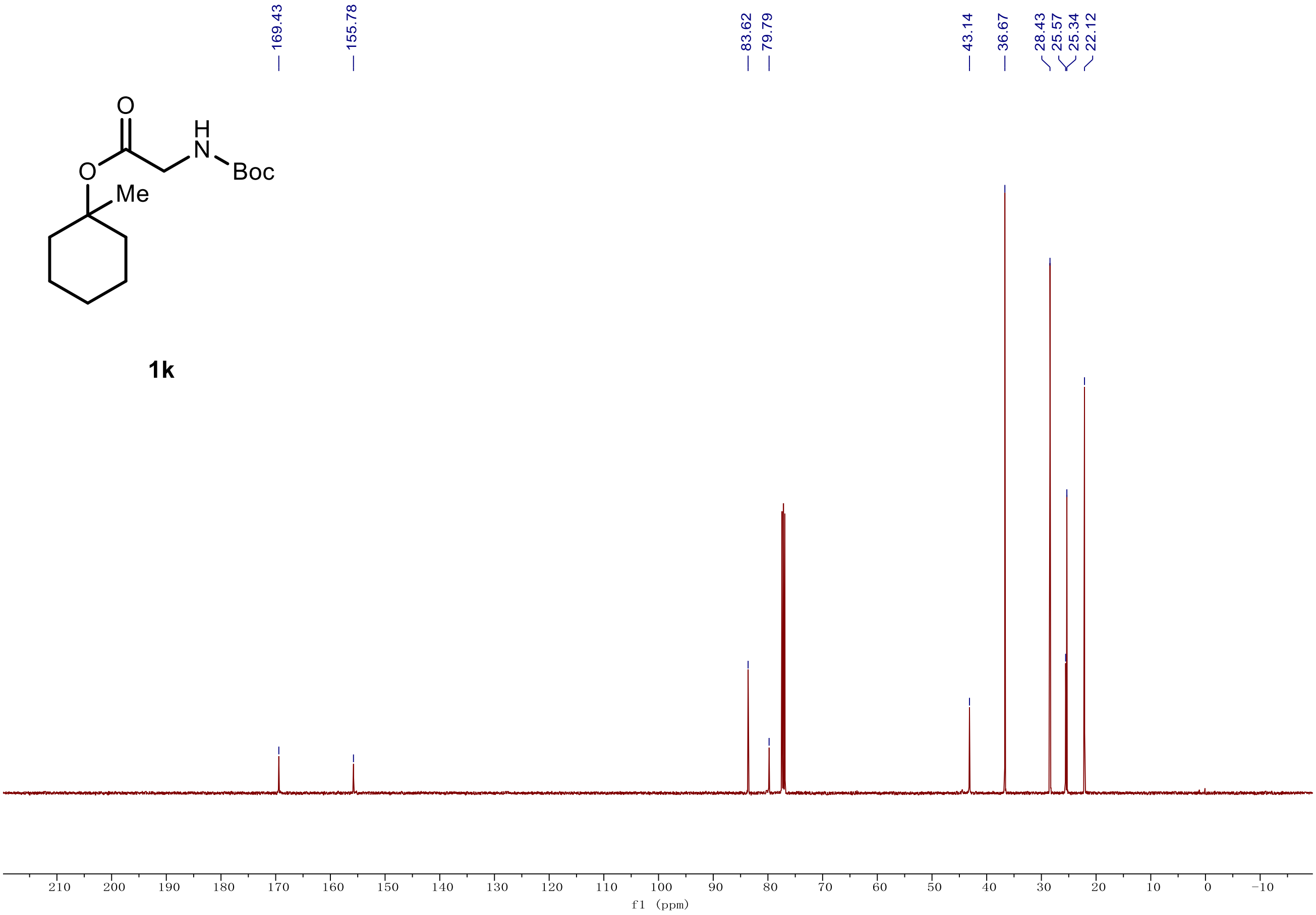

Figure S40 | ^{1}H NMR (500 MHz, $CDCl_3$) spectrum of **1l**.

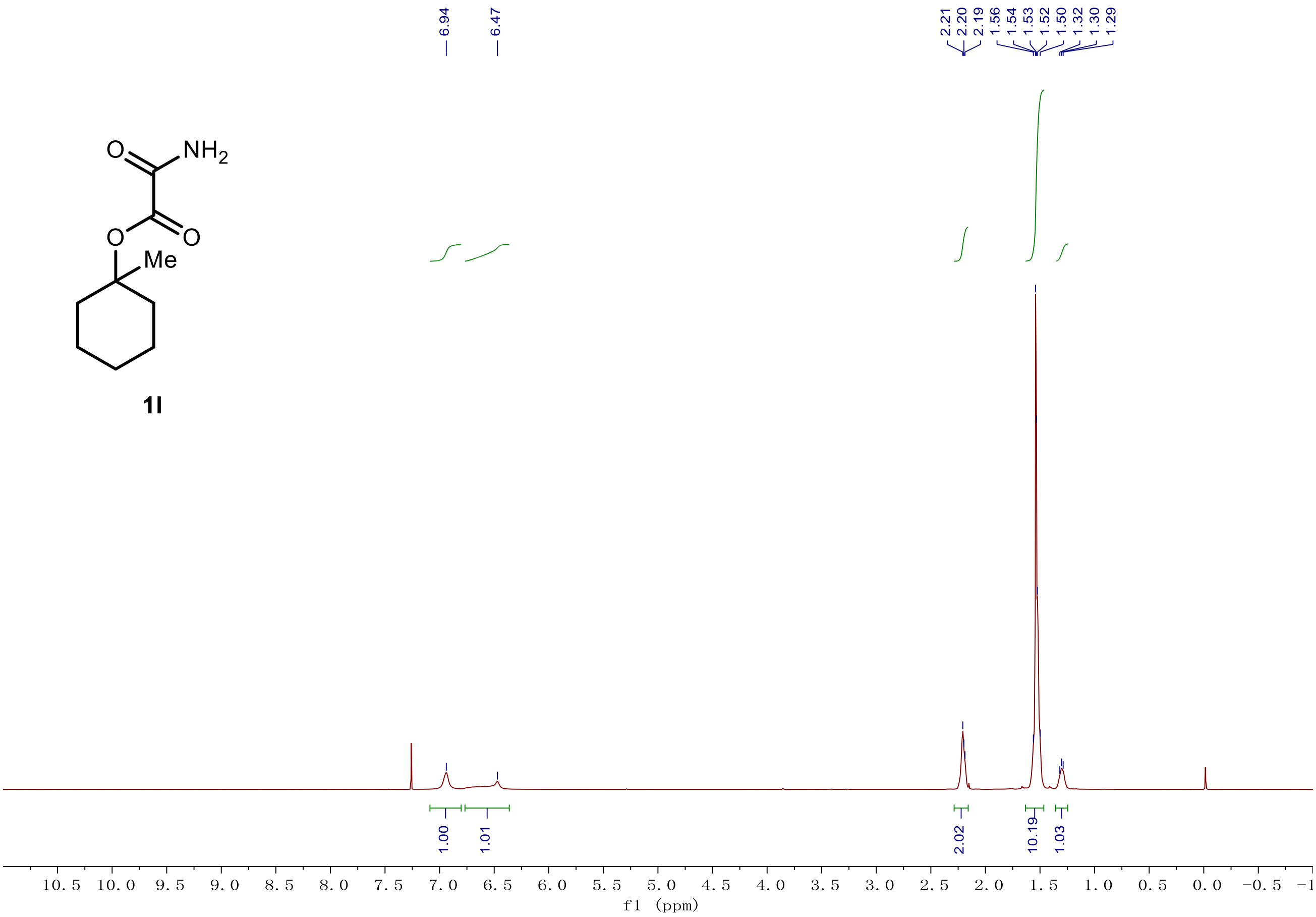

Figure S41 | ^{13}C NMR (126 MHz, $CDCl_3$) spectrum of **1l**.

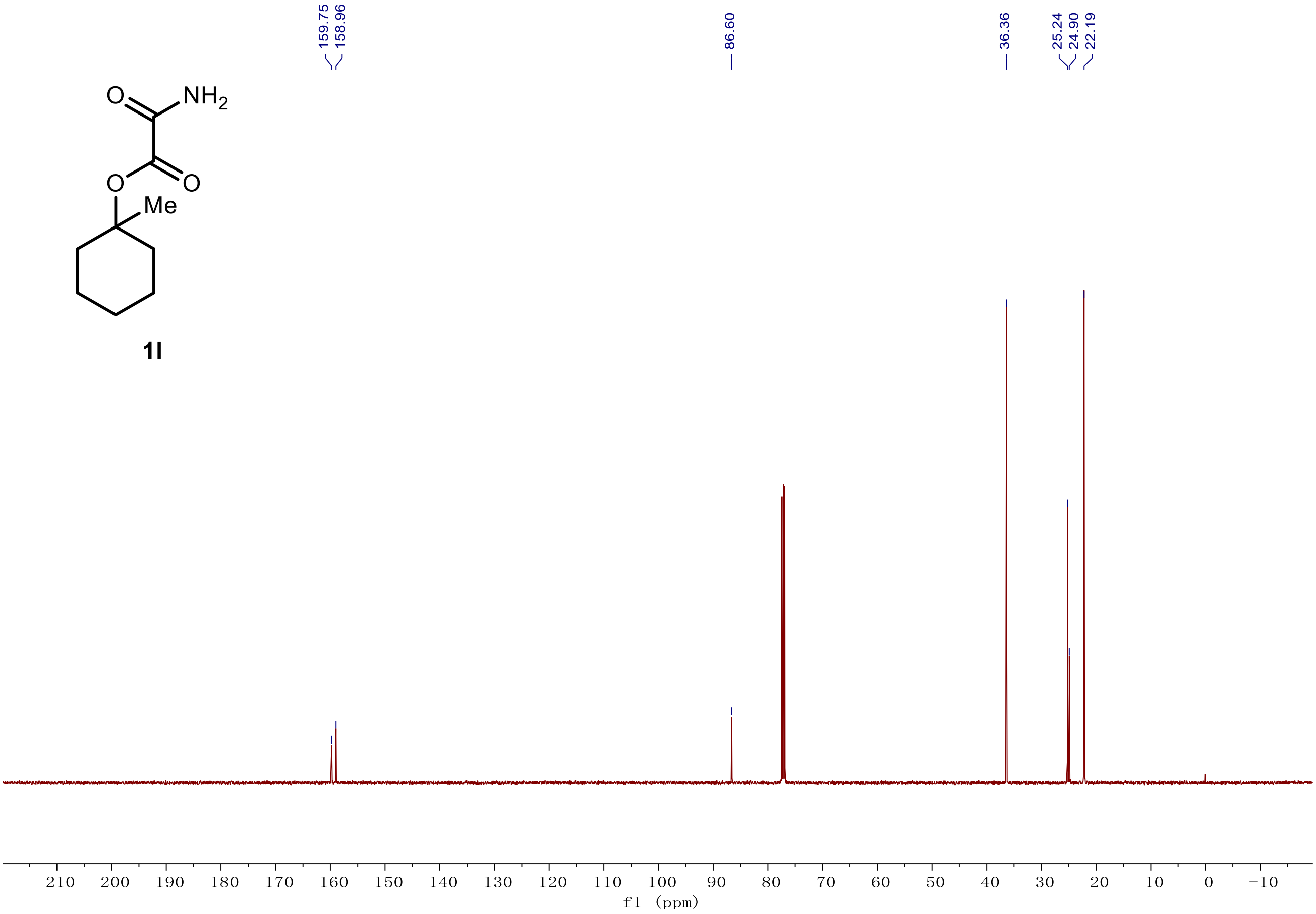

Figure S42 | ^{1}H NMR (500 MHz, $CDCl_3$) spectrum of **1n**.

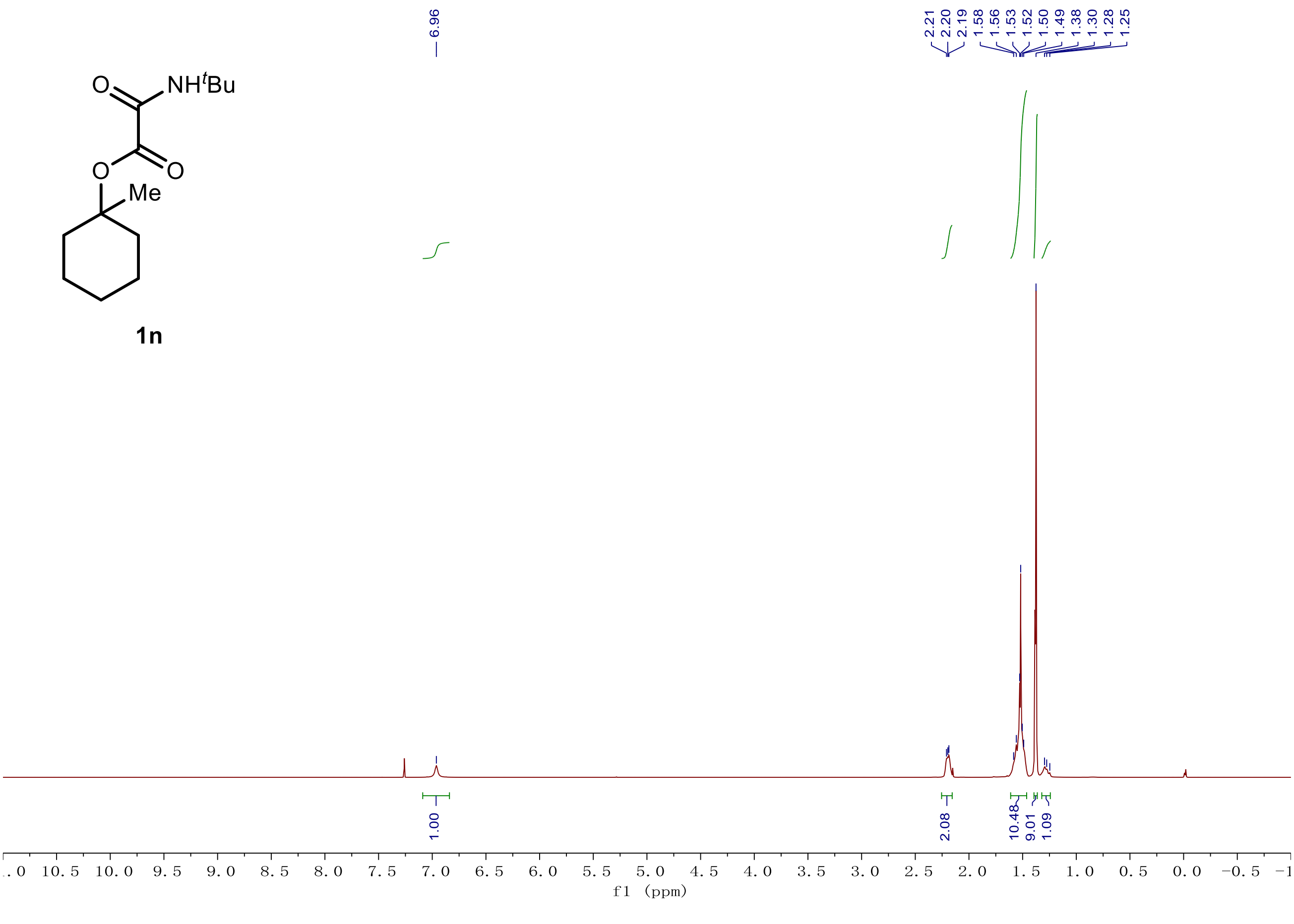

Figure S43 | ^{13}C NMR (126 MHz, $CDCl_3$) spectrum of **1n**.

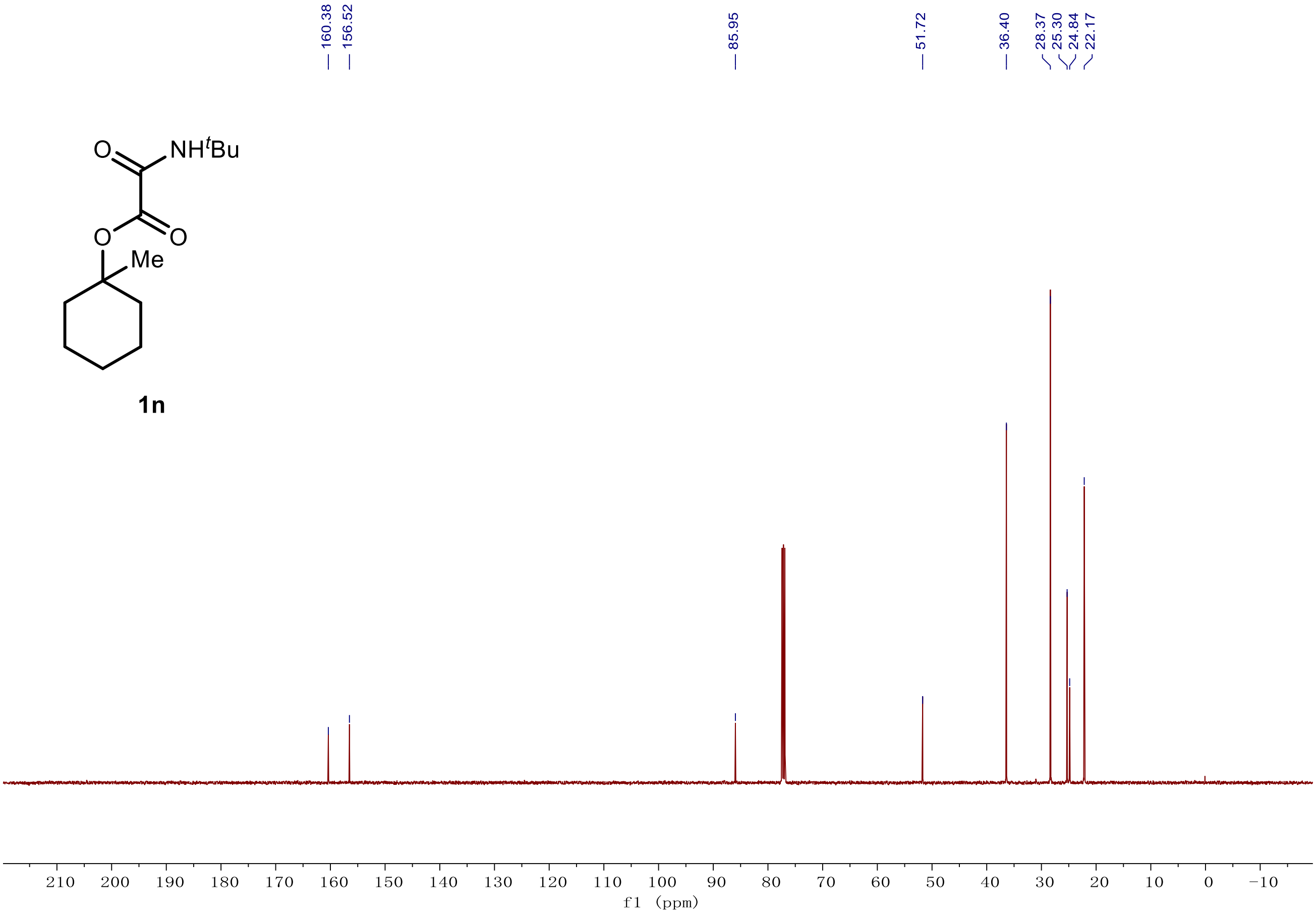

Figure S44 | ^{1}H NMR (400 MHz, $CDCl_3$) spectrum of **3**.

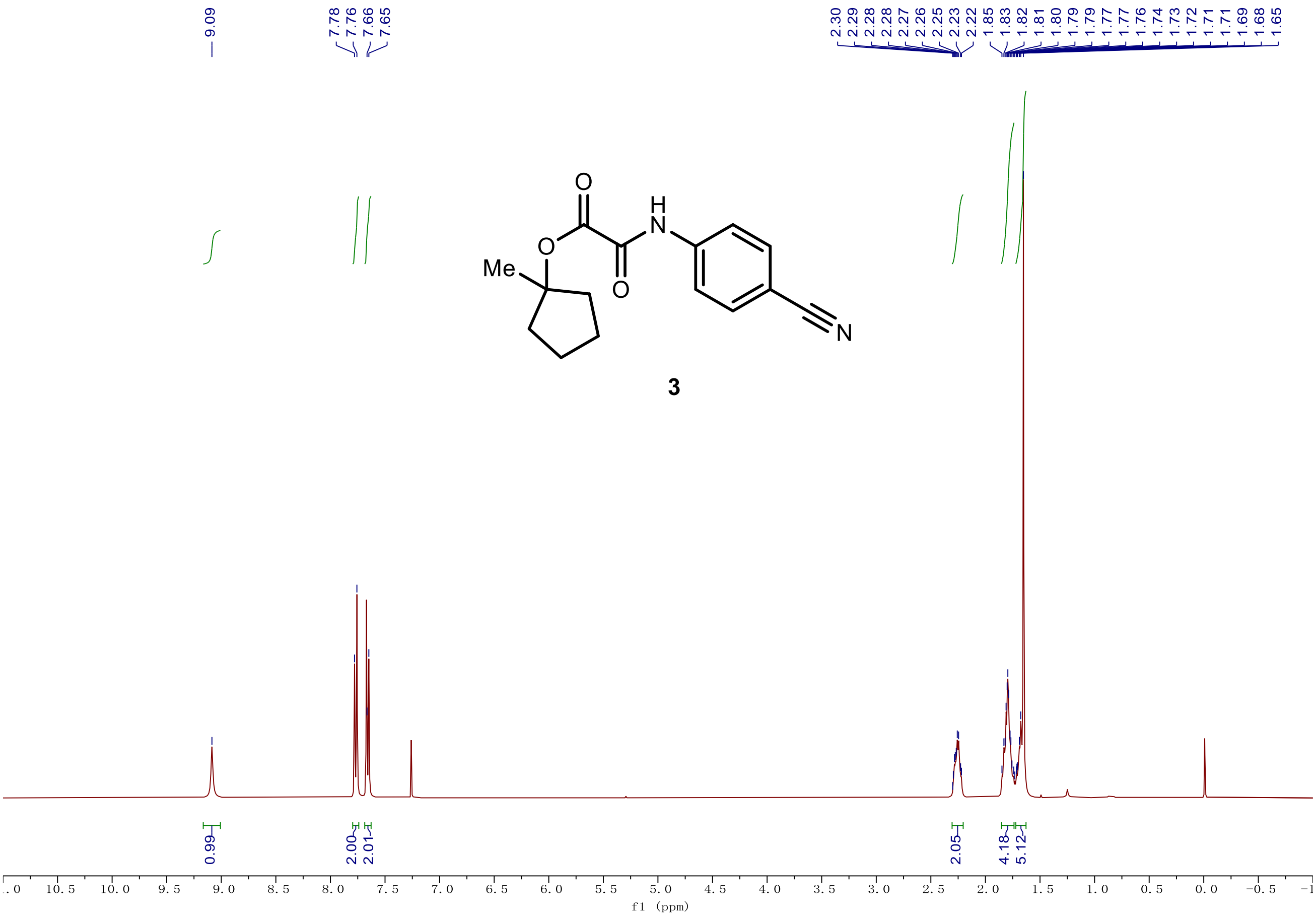

Figure S45 | ^{13}C NMR (101 MHz, $CDCl_3$) spectrum of **3**.

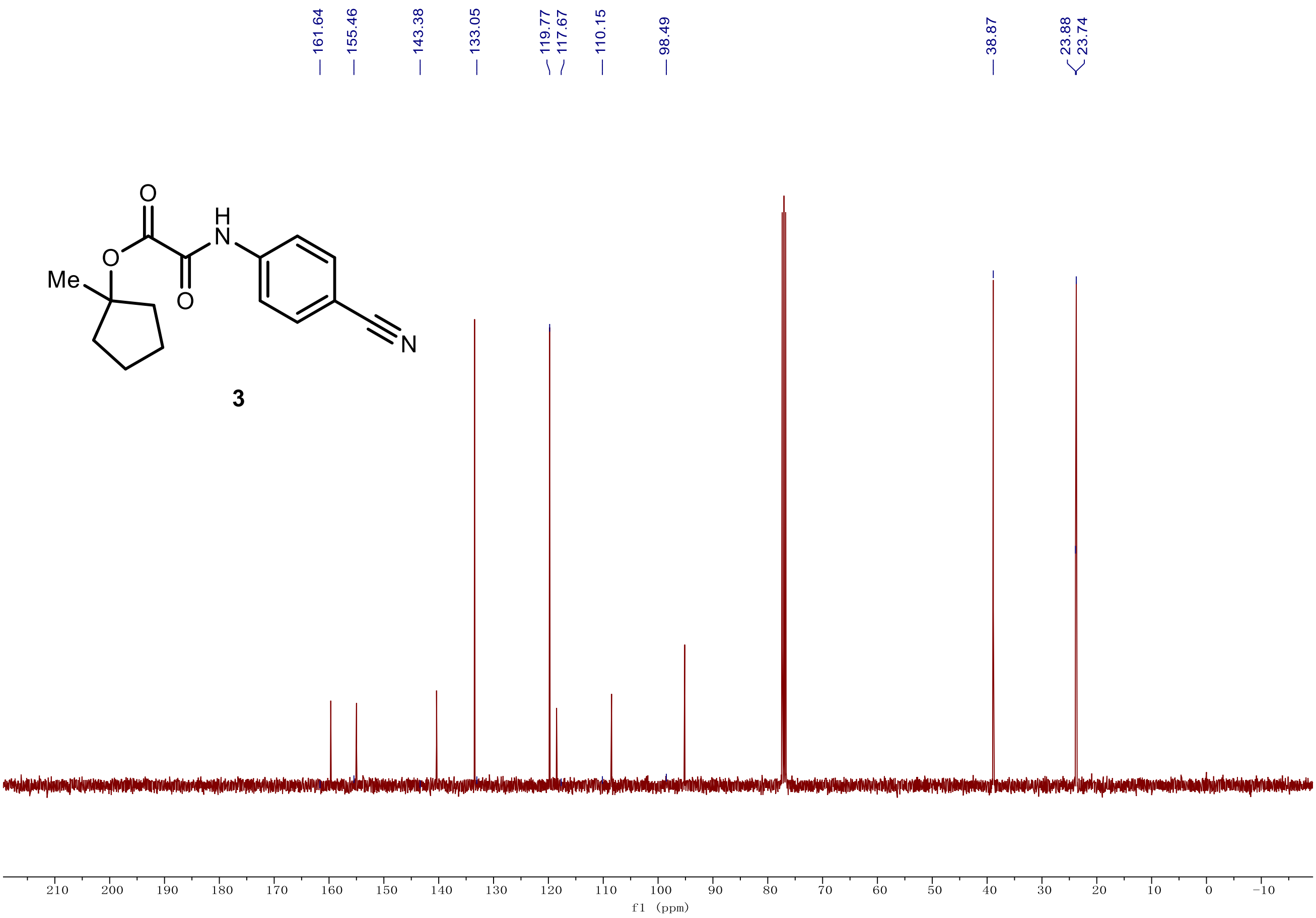

Figure S46 | ^{1}H NMR (400 MHz, $CDCl_3$) spectrum of **1m**.

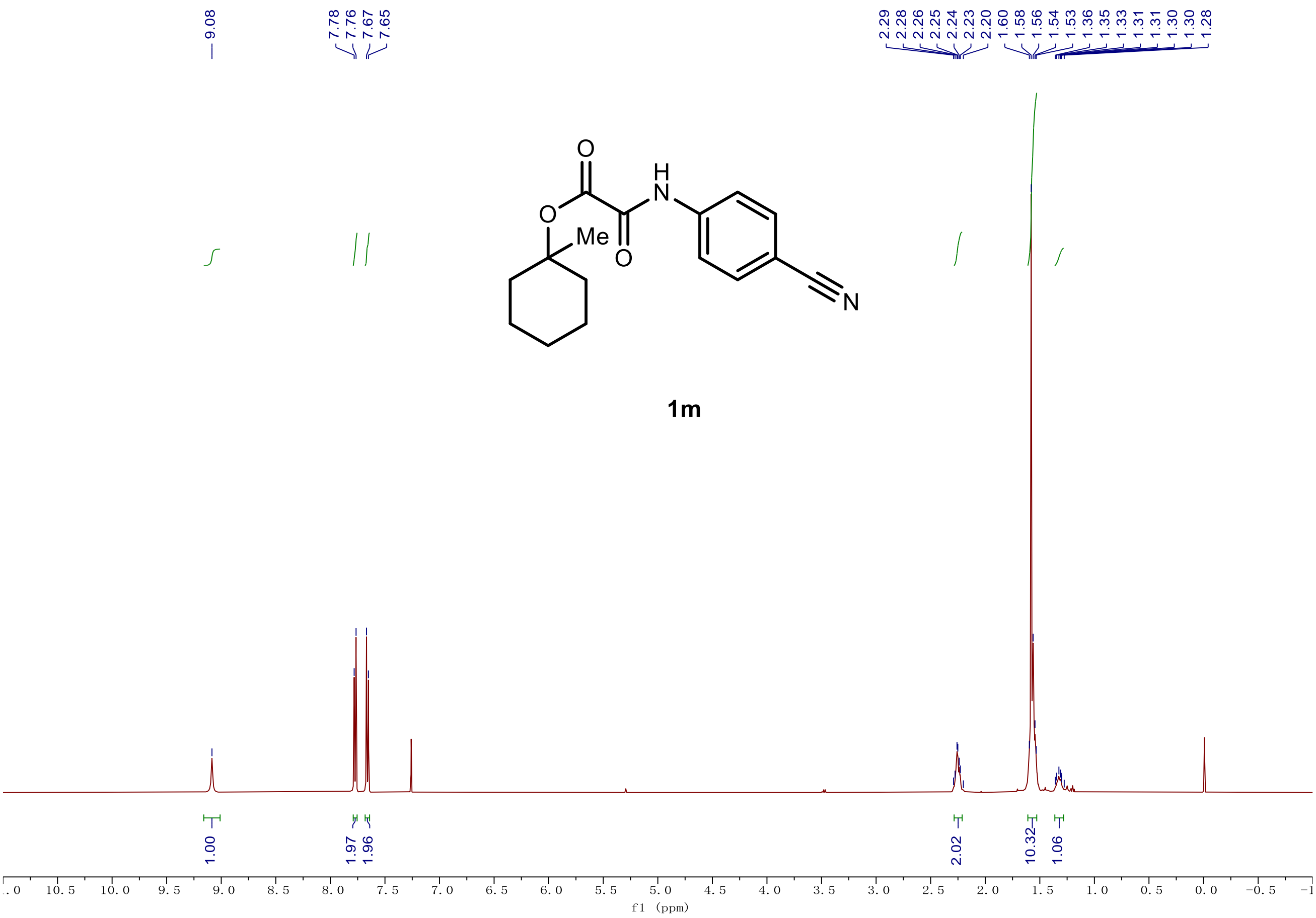

Figure S47 | ^{13}C NMR (101 MHz, $CDCl_3$) spectrum of **1m**.

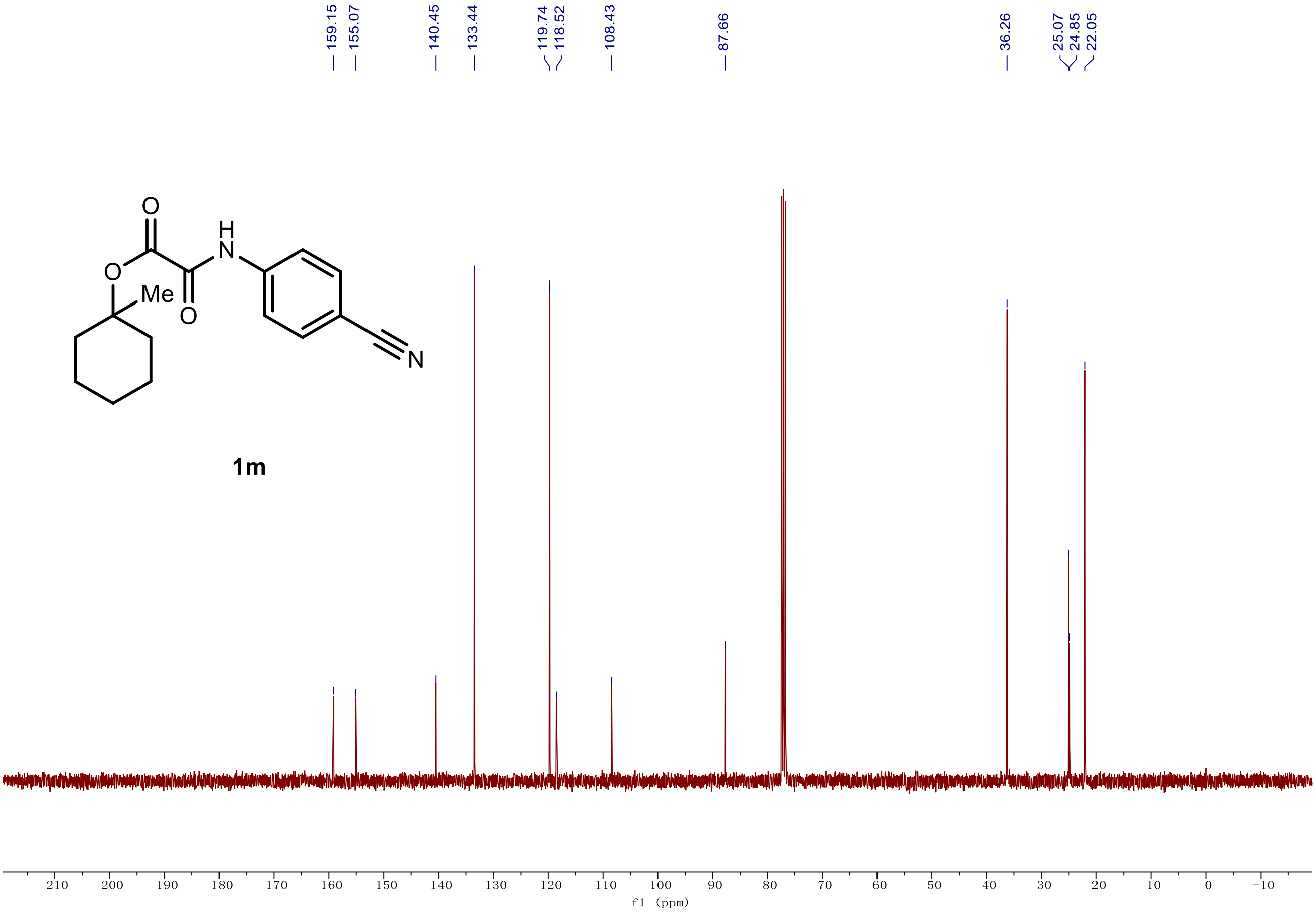

Figure S48 | ^{1}H NMR (400 MHz, $CDCl_3$) spectrum of **5**.

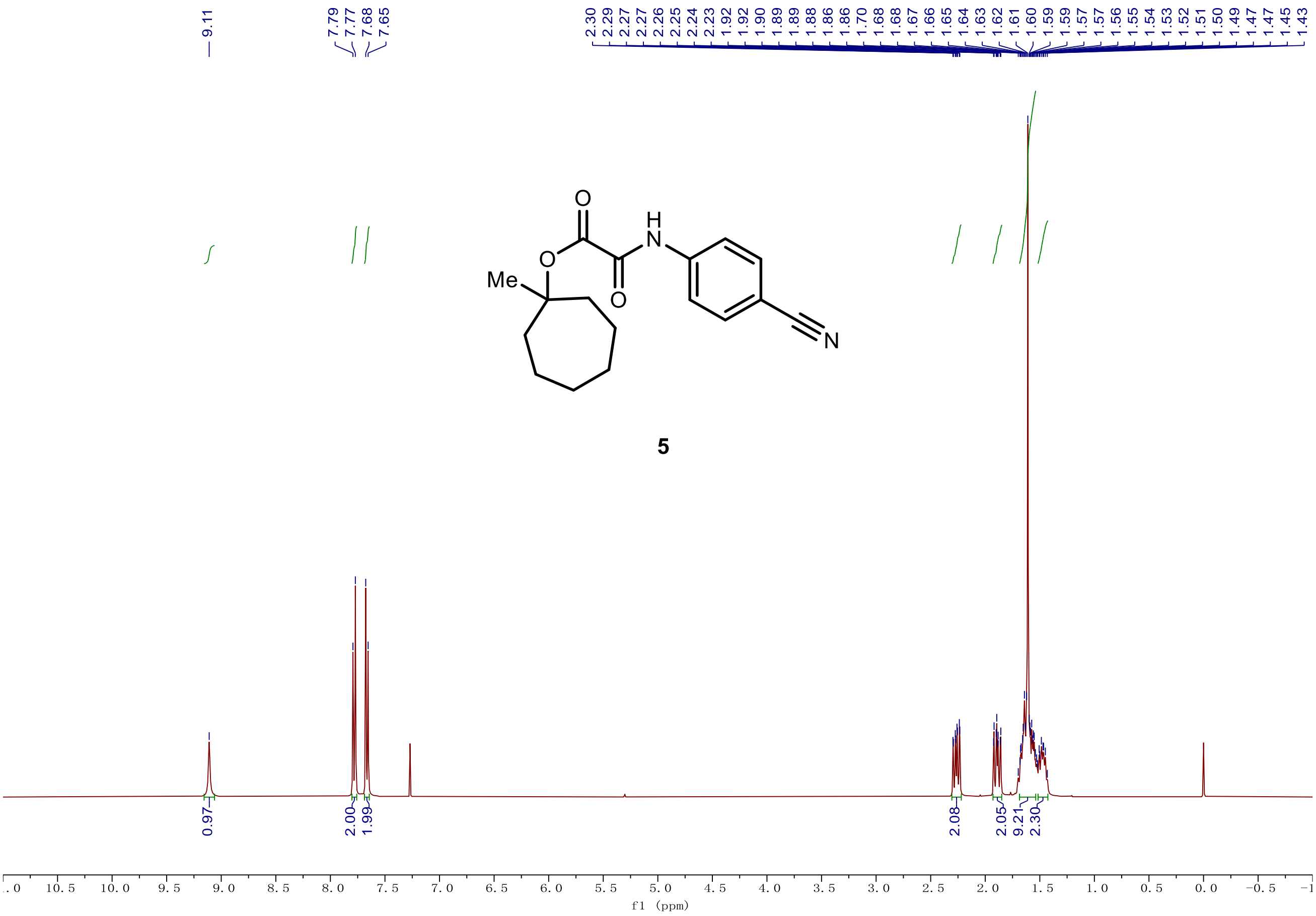

Figure S49 | ^{13}C NMR (101 MHz, $CDCl_3$) spectrum of **5**.

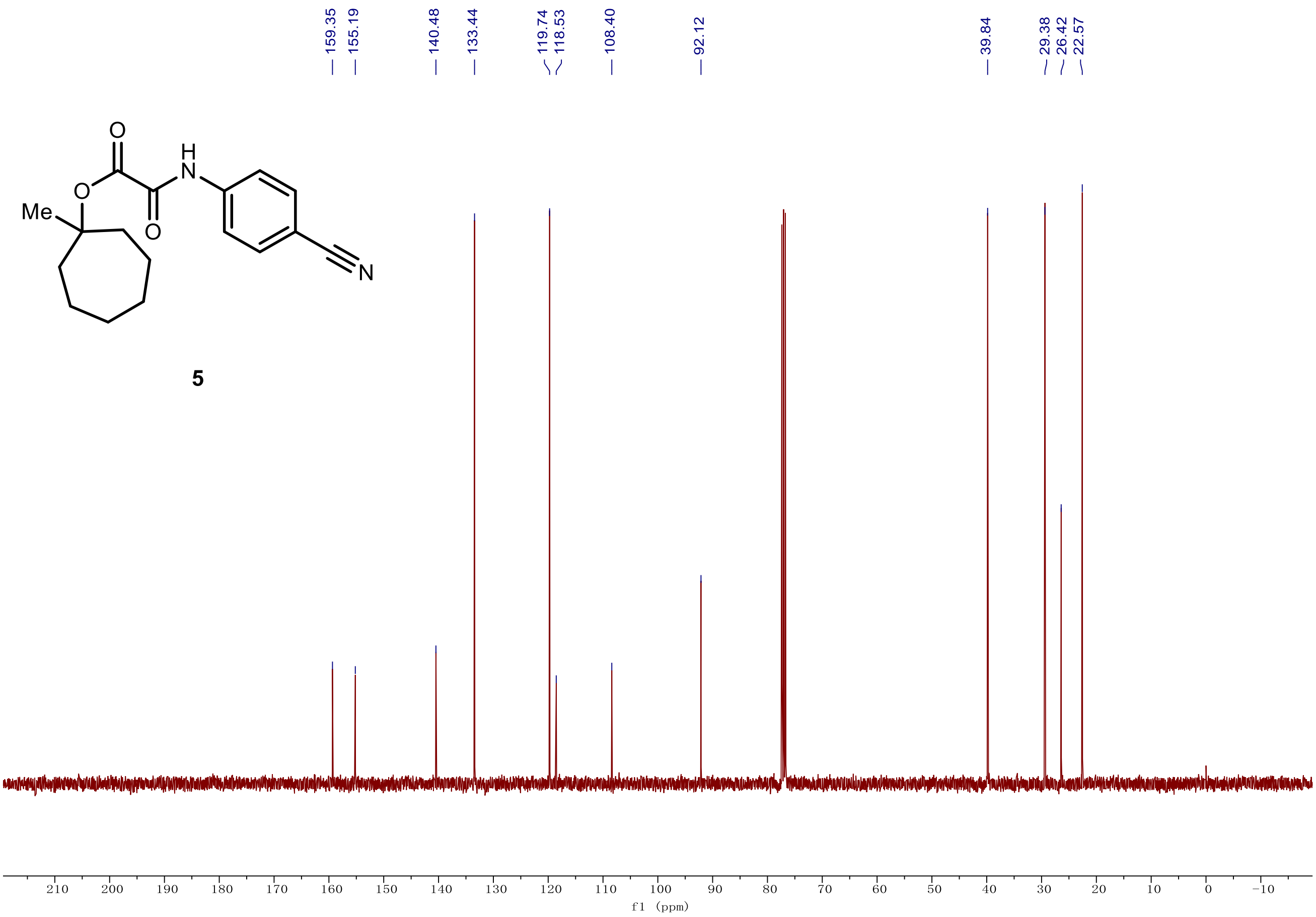

Figure S50 | ^{1}H NMR (400 MHz, $CDCl_3$) spectrum of **7**.

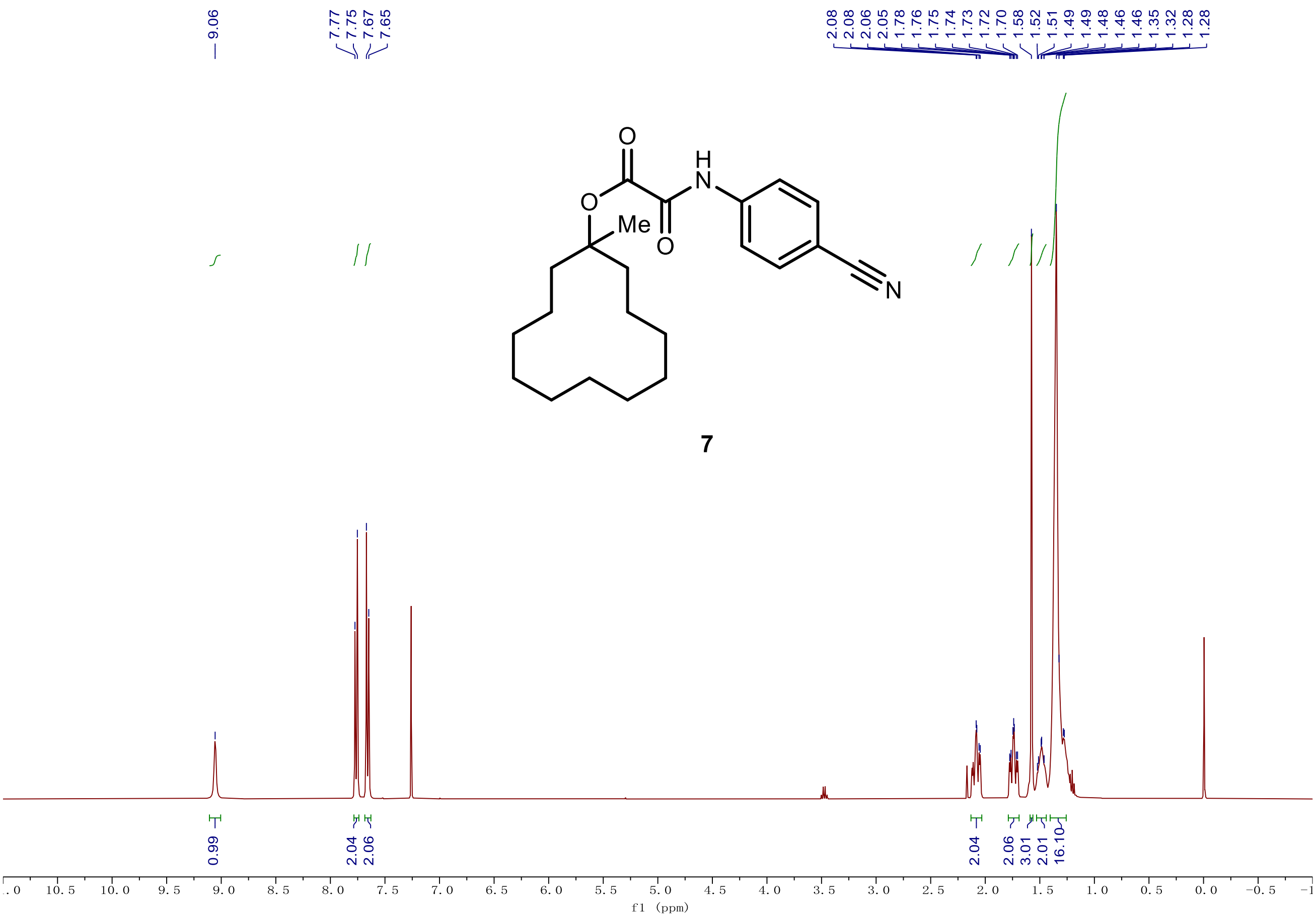

Figure S51 | ^{13}C NMR (101 MHz, $CDCl_3$) spectrum of **7**.

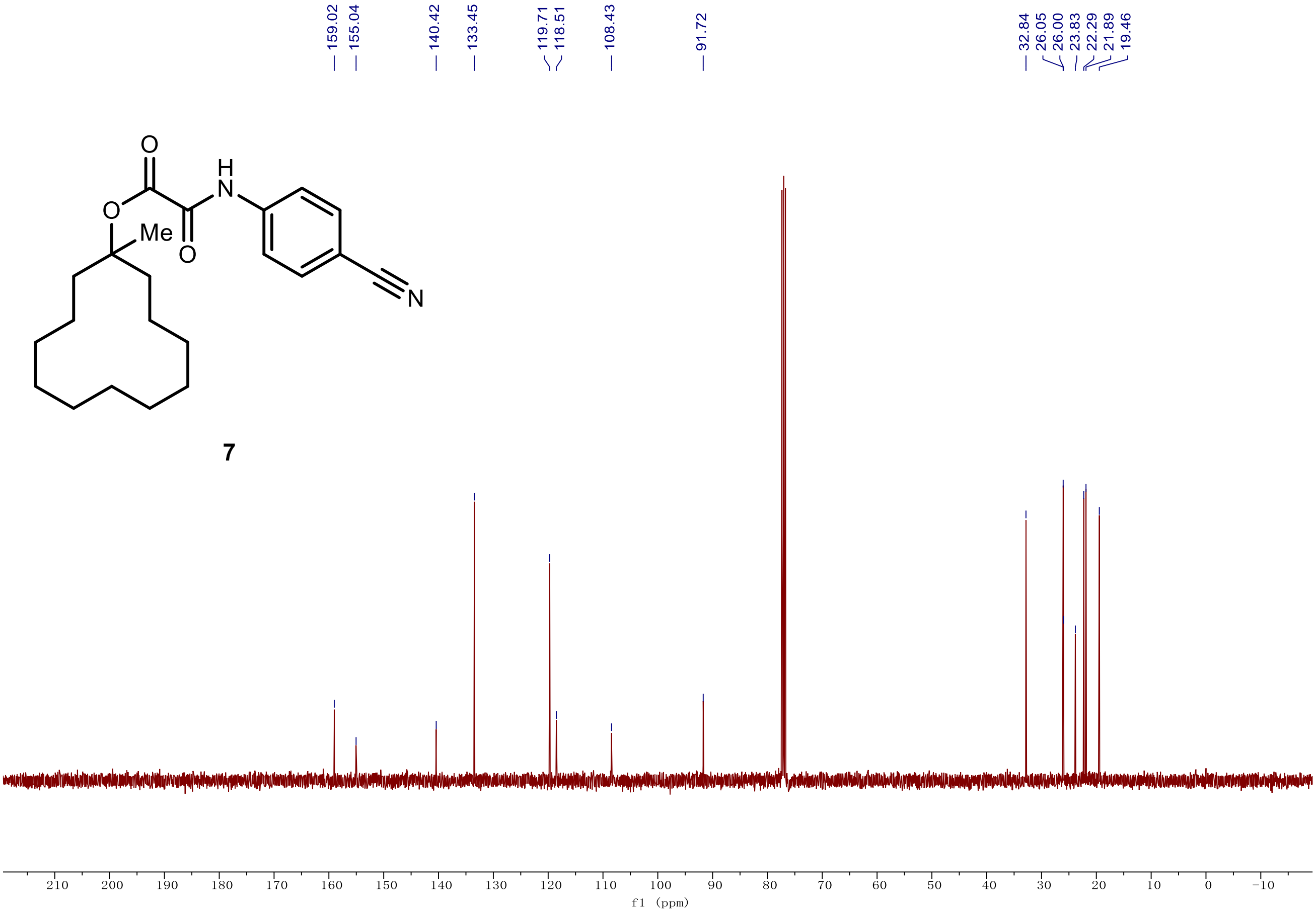

Figure S52 | ^{1}H NMR (400 MHz, $CDCl_3$) spectrum of **9**.

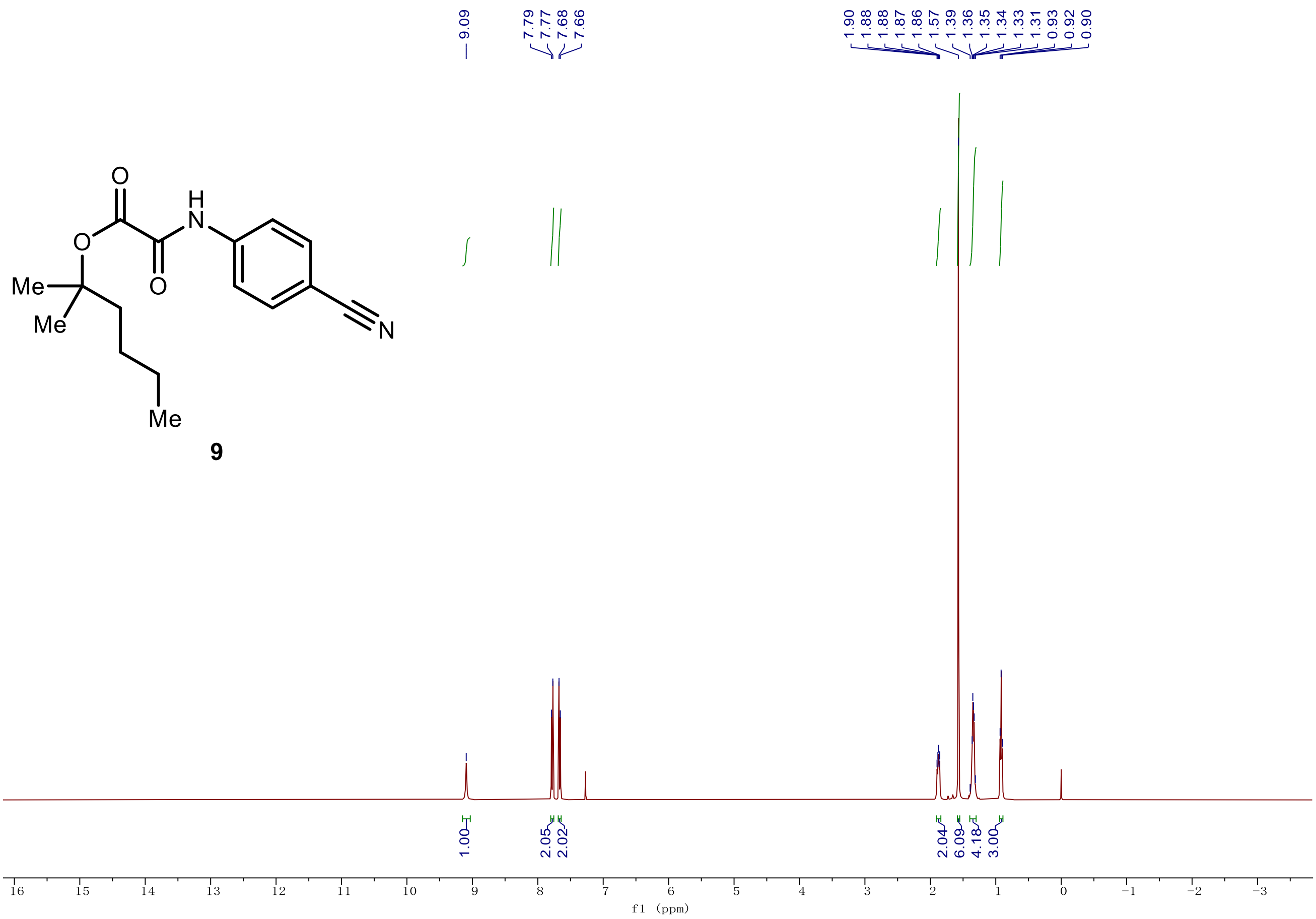

Figure S53 | ^{13}C NMR (101 MHz, $CDCl_3$) spectrum of **9**.

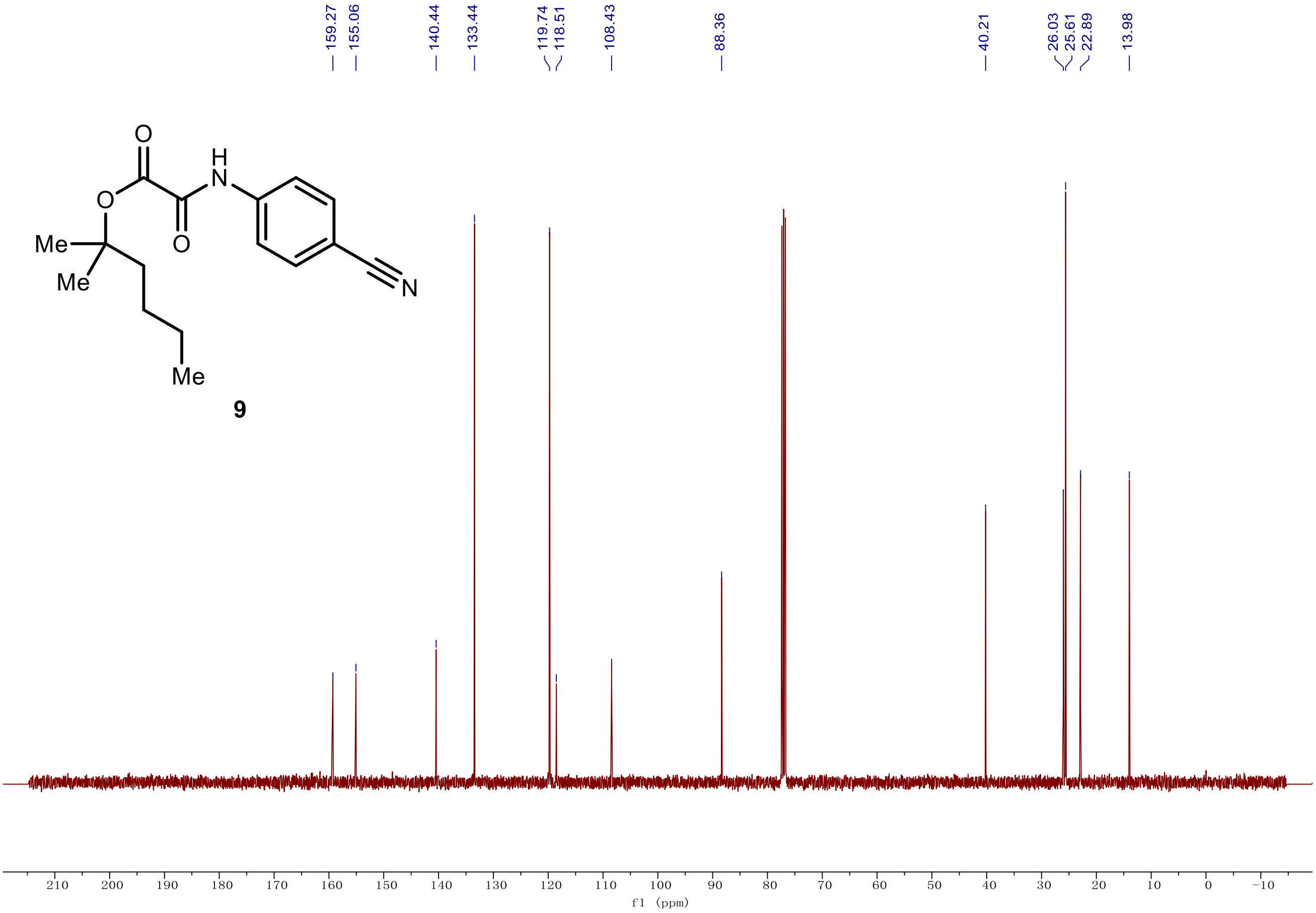

Figure S54 | ^{1}H NMR (400 MHz, $CDCl_3$) spectrum of **11**.

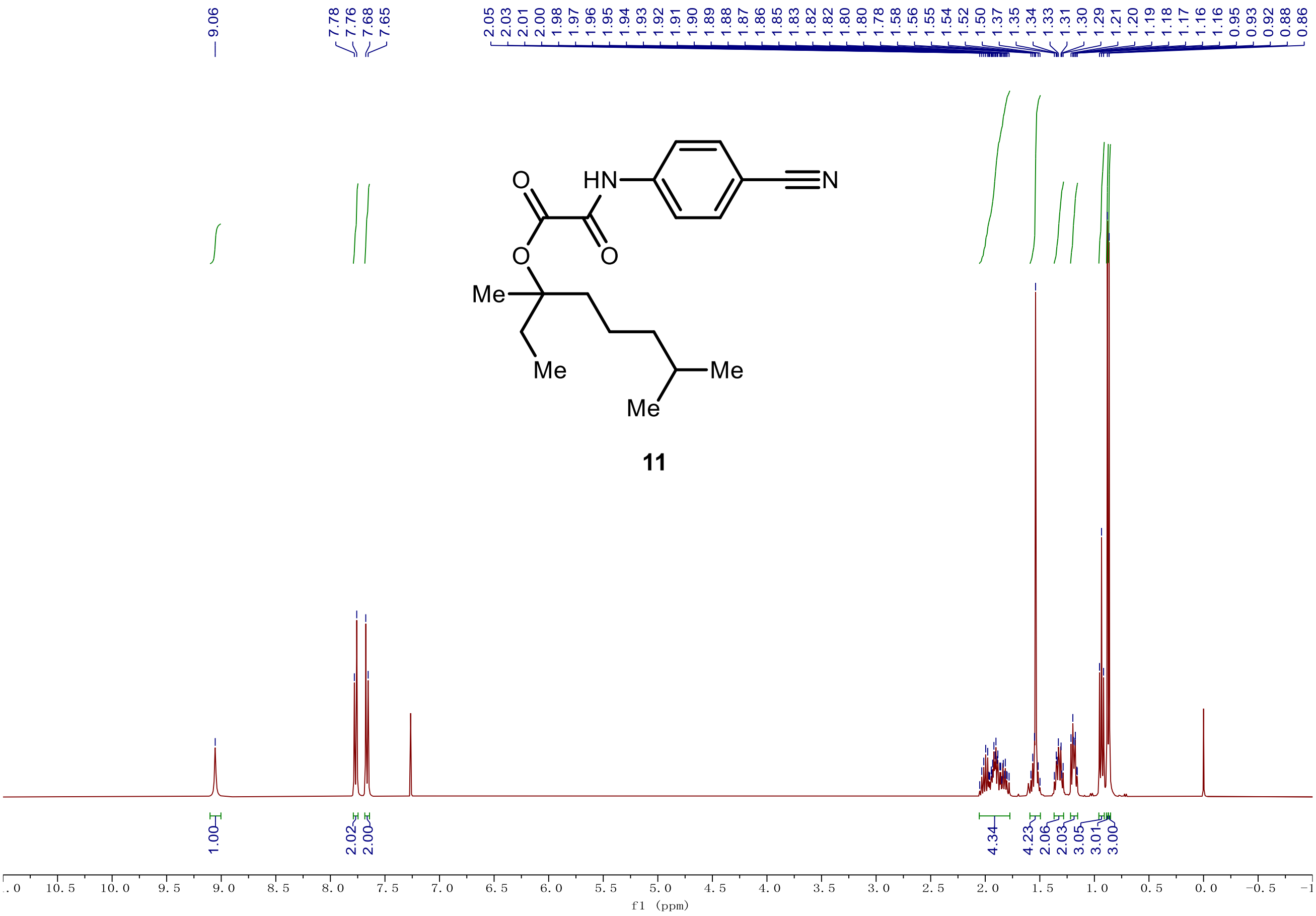

Figure S55 | ^{13}C NMR (101 MHz, $CDCl_3$) spectrum of **11**.

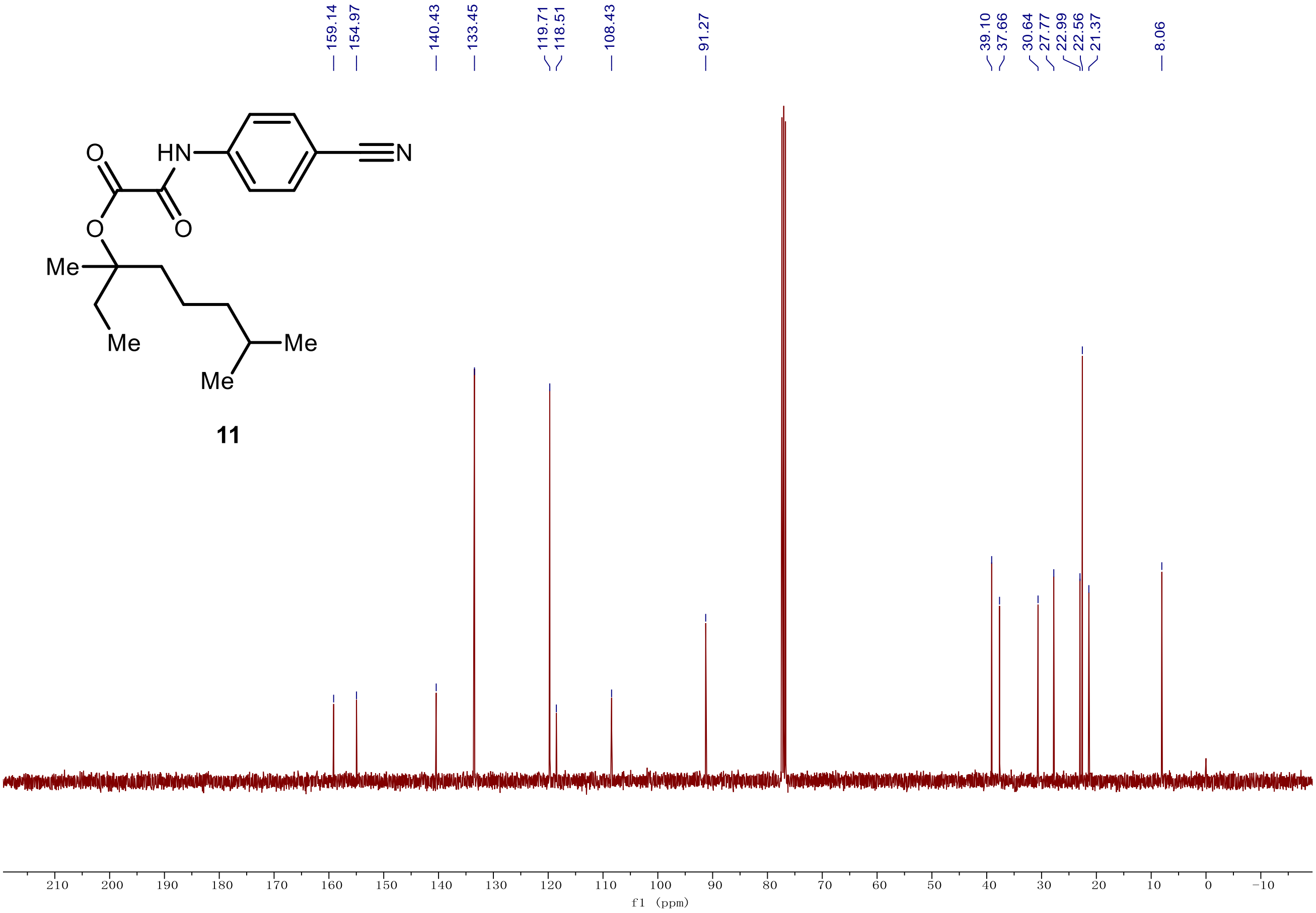

Figure S56 | ^{1}H NMR (400 MHz, $CDCl_3$) spectrum of **13**.

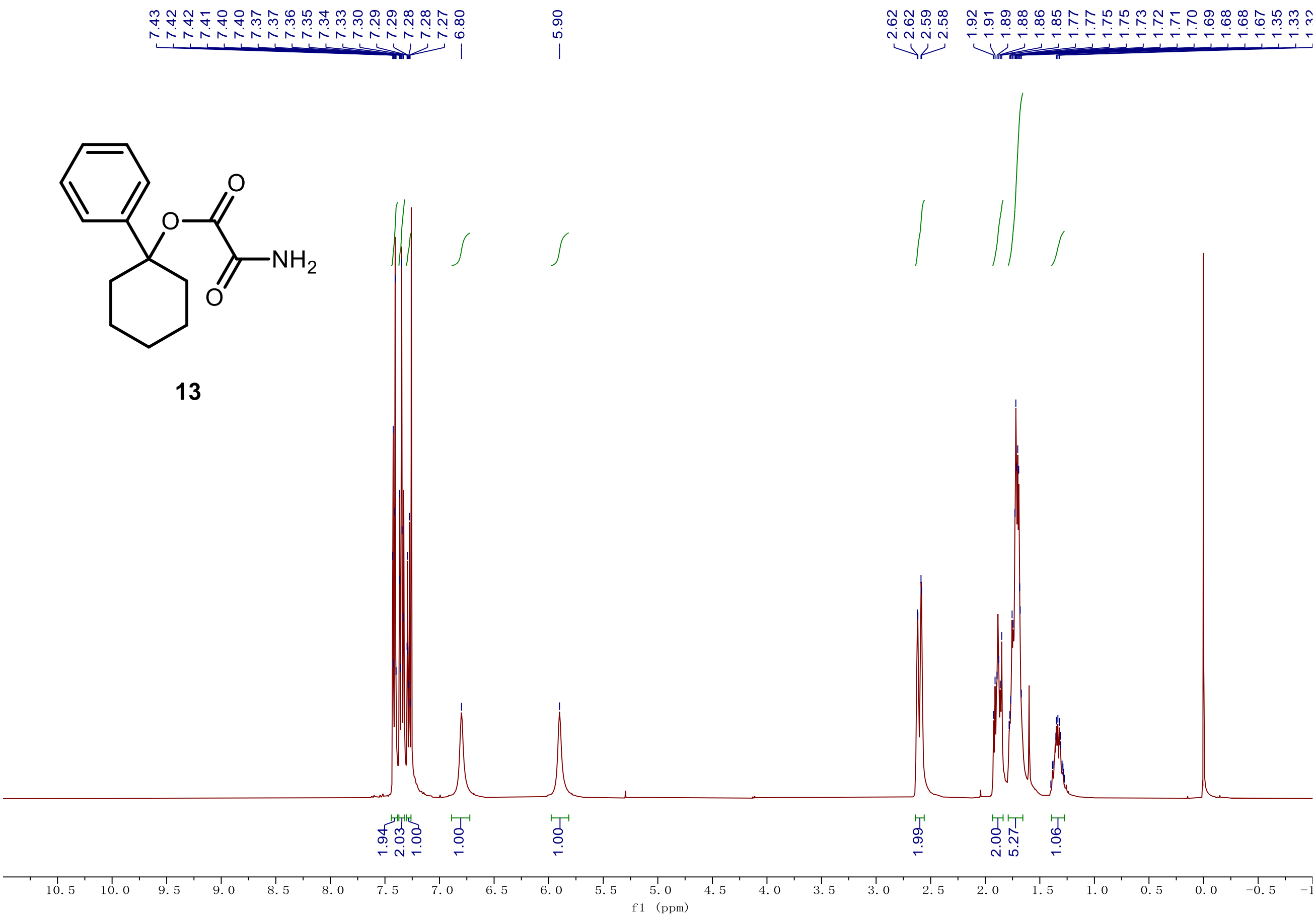

Figure S57 | ^{13}C NMR (101 MHz, $CDCl_3$) spectrum of **13**.

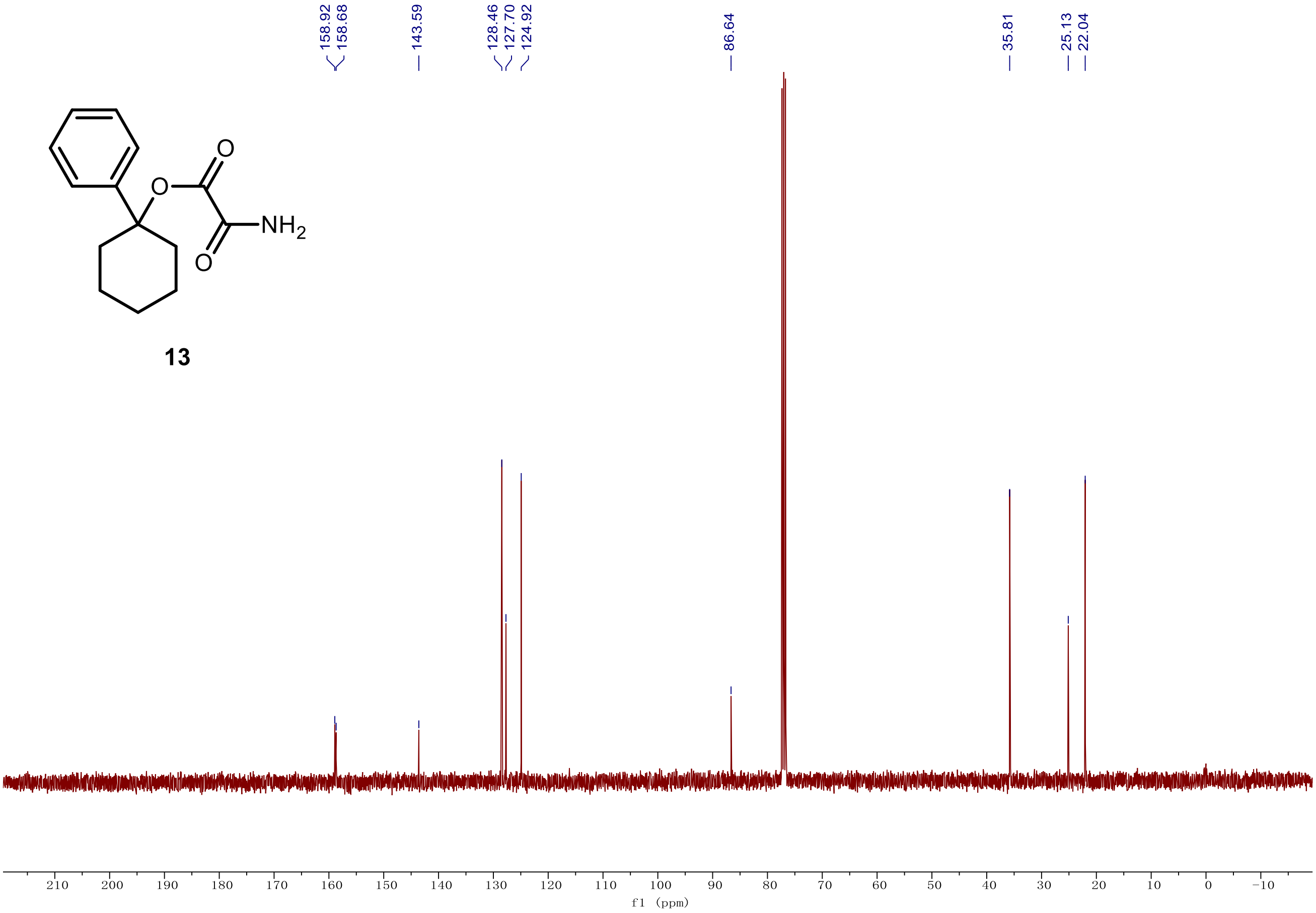

Figure S58 | ^{1}H NMR (400 MHz, $CDCl_3$) spectrum of **15**.

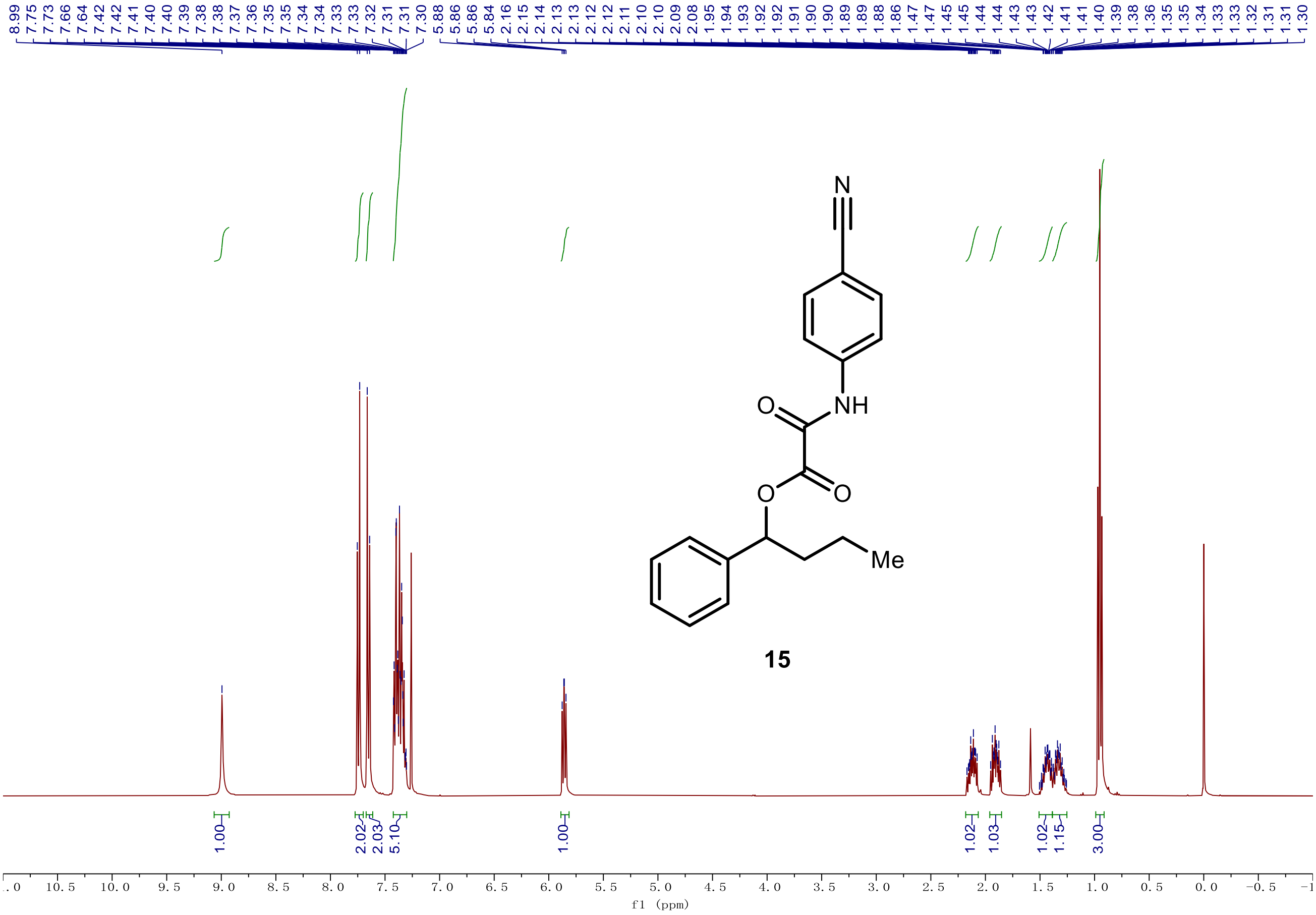

Figure S59 | ^{13}C NMR (101 MHz, $CDCl_3$) spectrum of **15**.

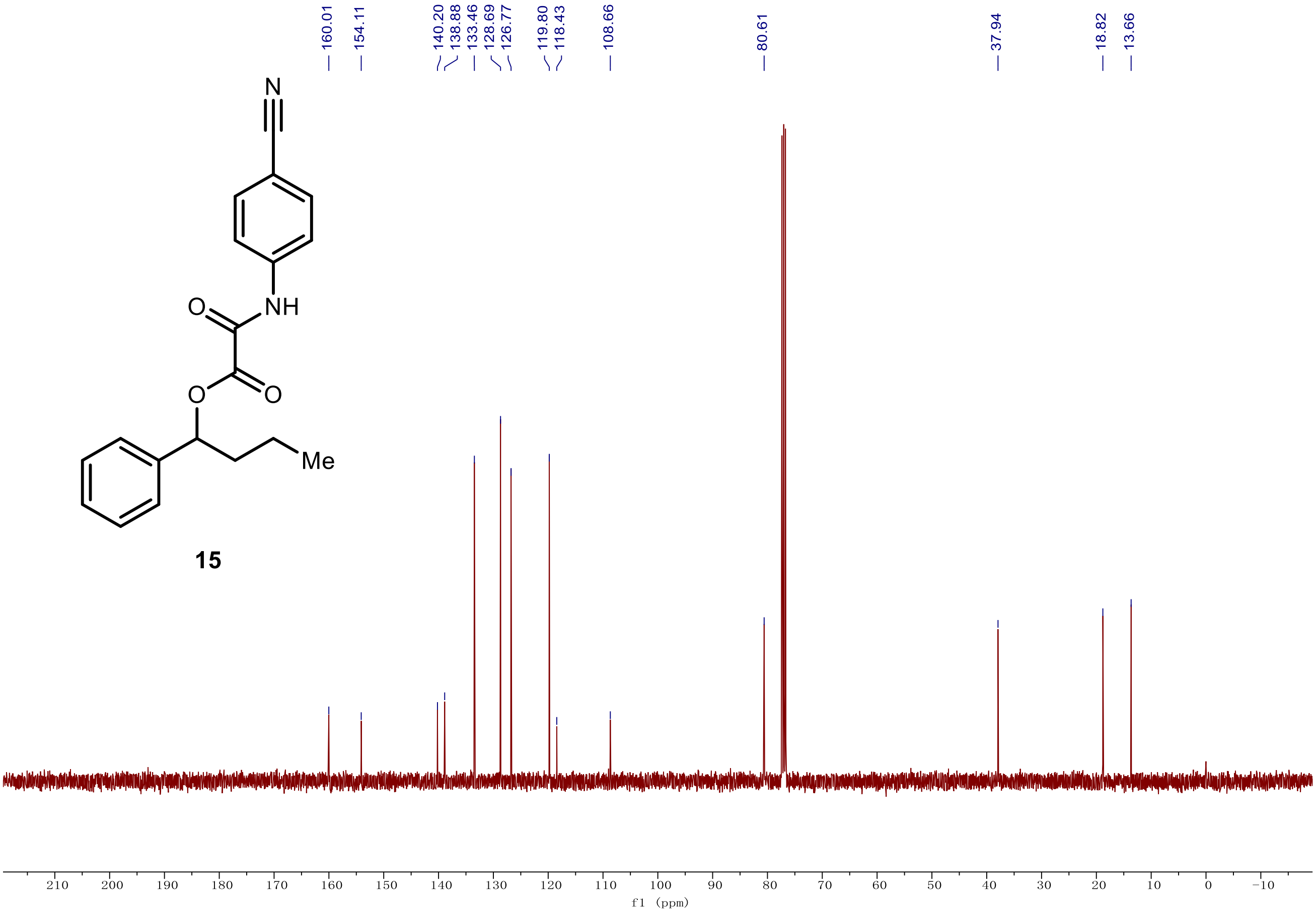

Figure S60 | ^{1}H NMR (400 MHz, $CDCl_3$) spectrum of **17**.

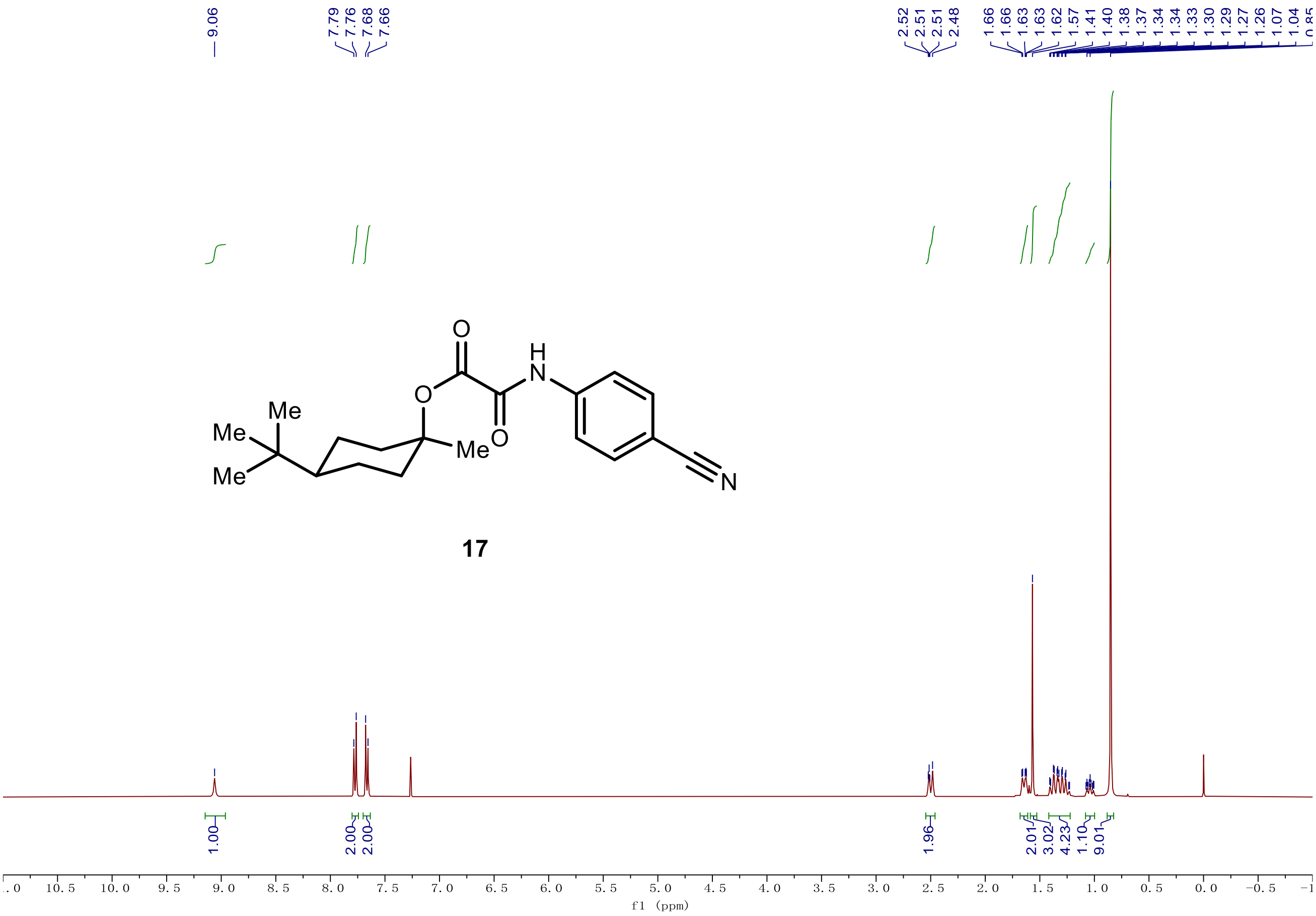

Figure S61 | ^{13}C NMR (101 MHz, $CDCl_3$) spectrum of **17**.

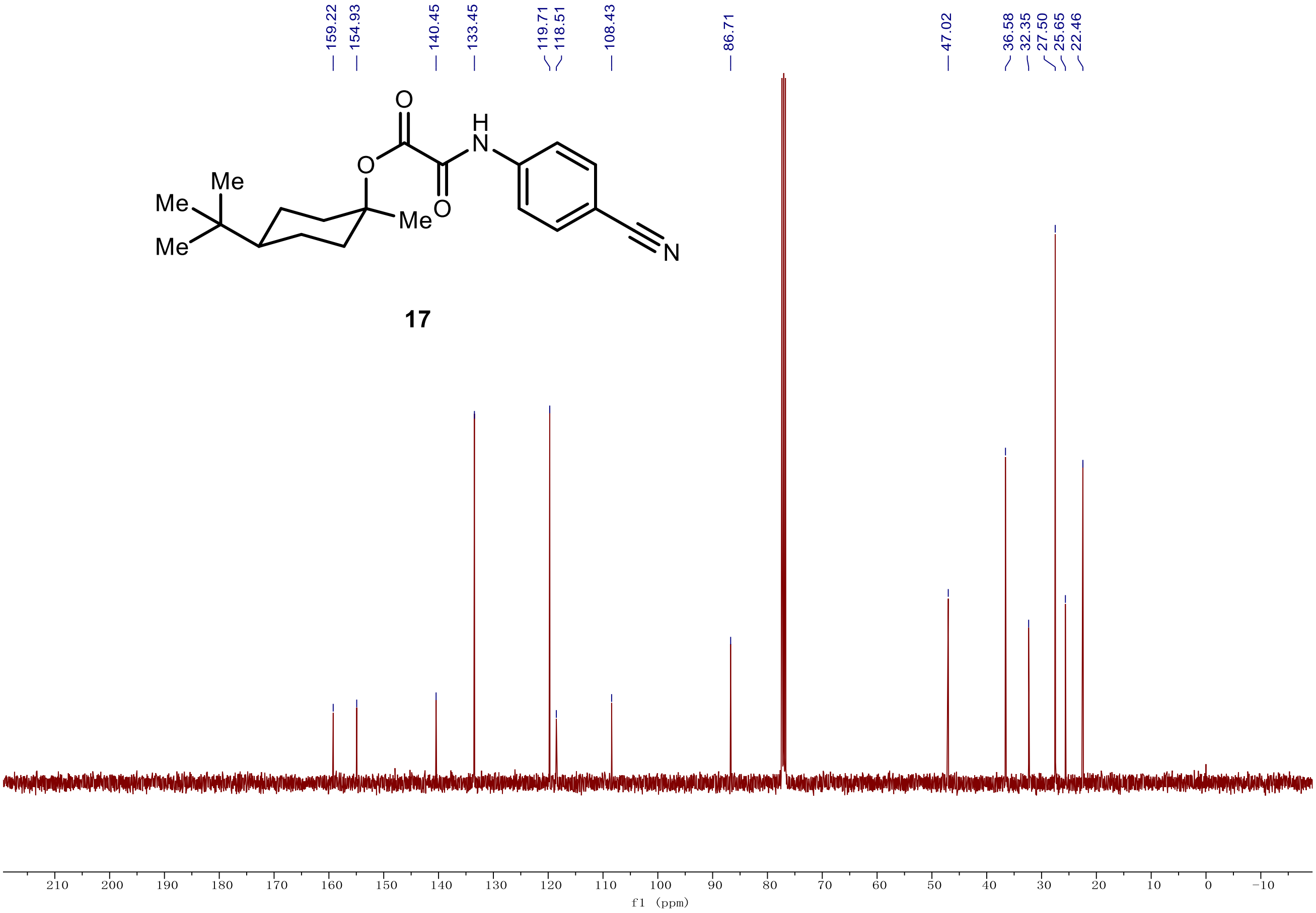

Figure S62 | ^{1}H NMR (400 MHz, $CDCl_3$) spectrum of **19**.

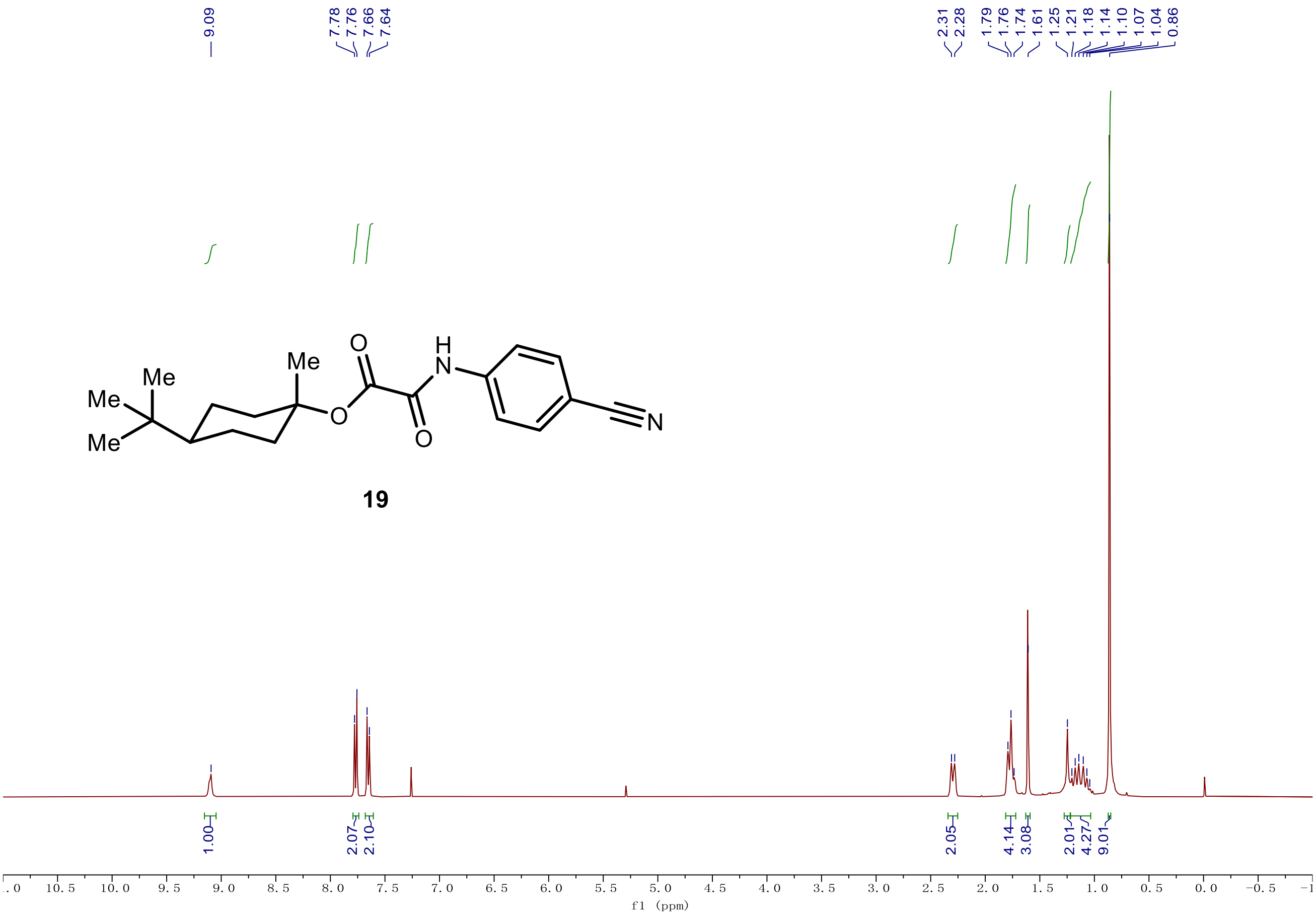

Figure S63 | ^{13}C NMR (101 MHz, $CDCl_3$) spectrum of **19**.

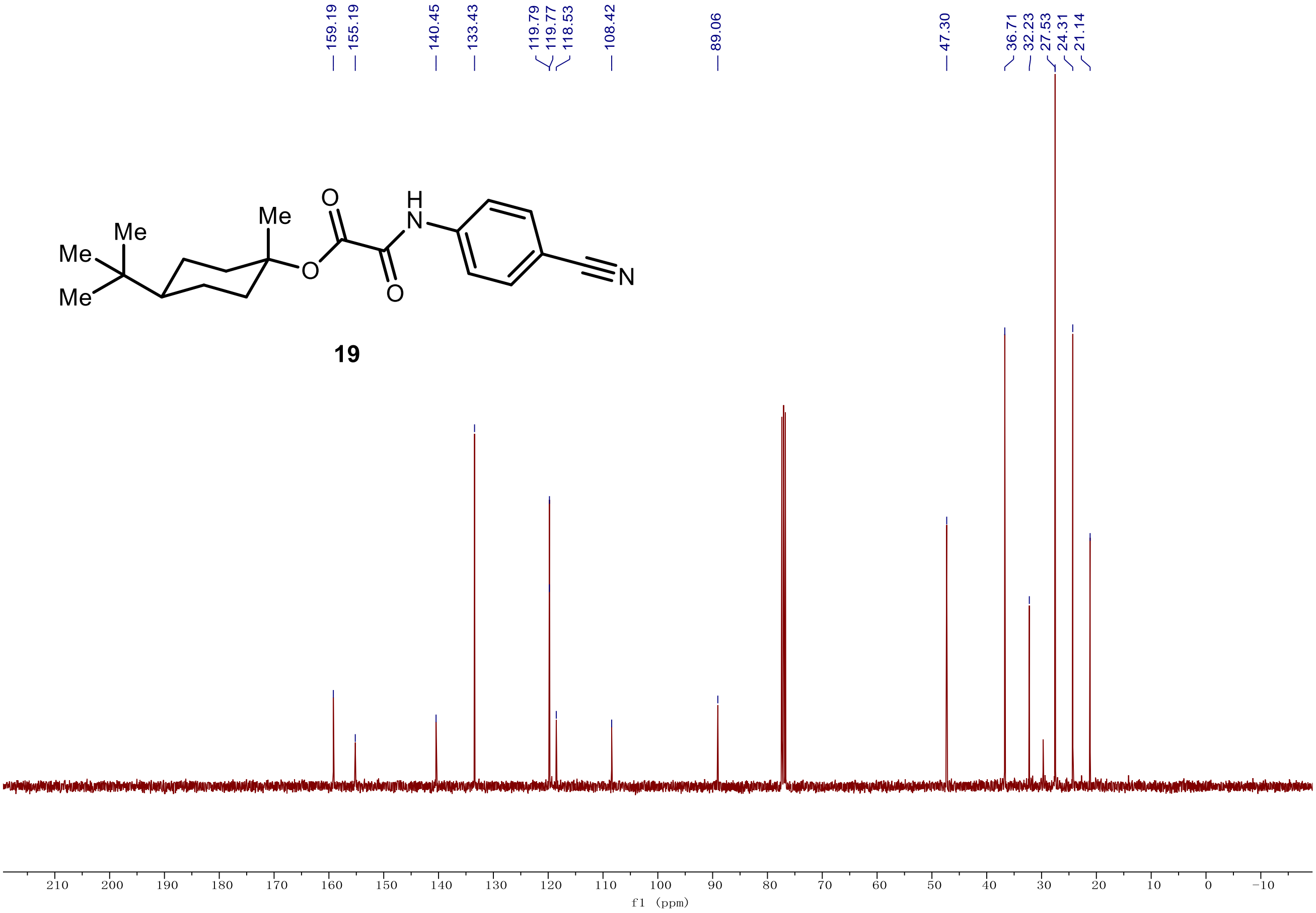

Figure S64 | ^{1}H NMR (400 MHz, $CDCl_3$) spectrum of **21**.

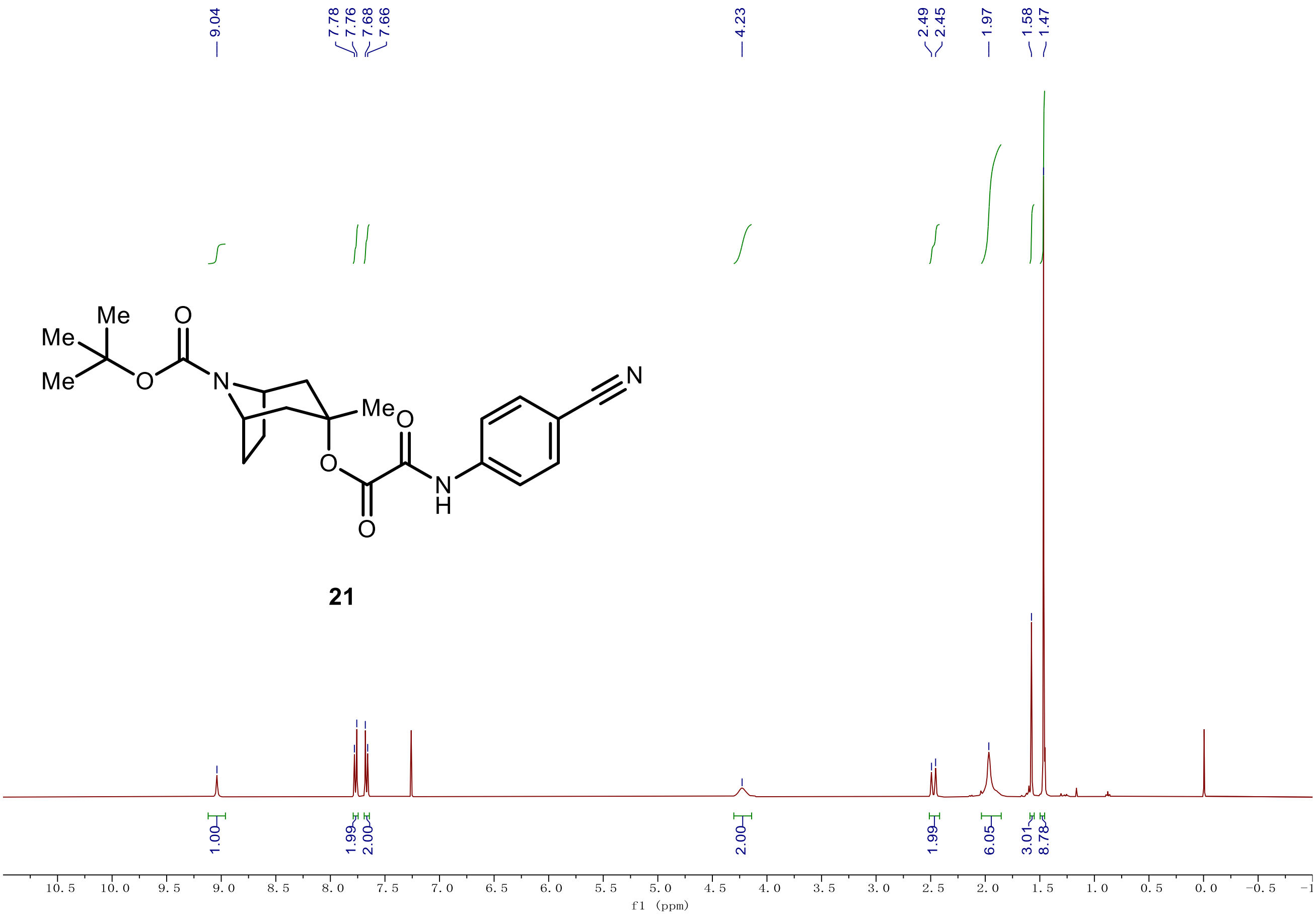

Figure S65 | ^{13}C NMR (101 MHz, CDCl$_3$) spectrum of **21**.

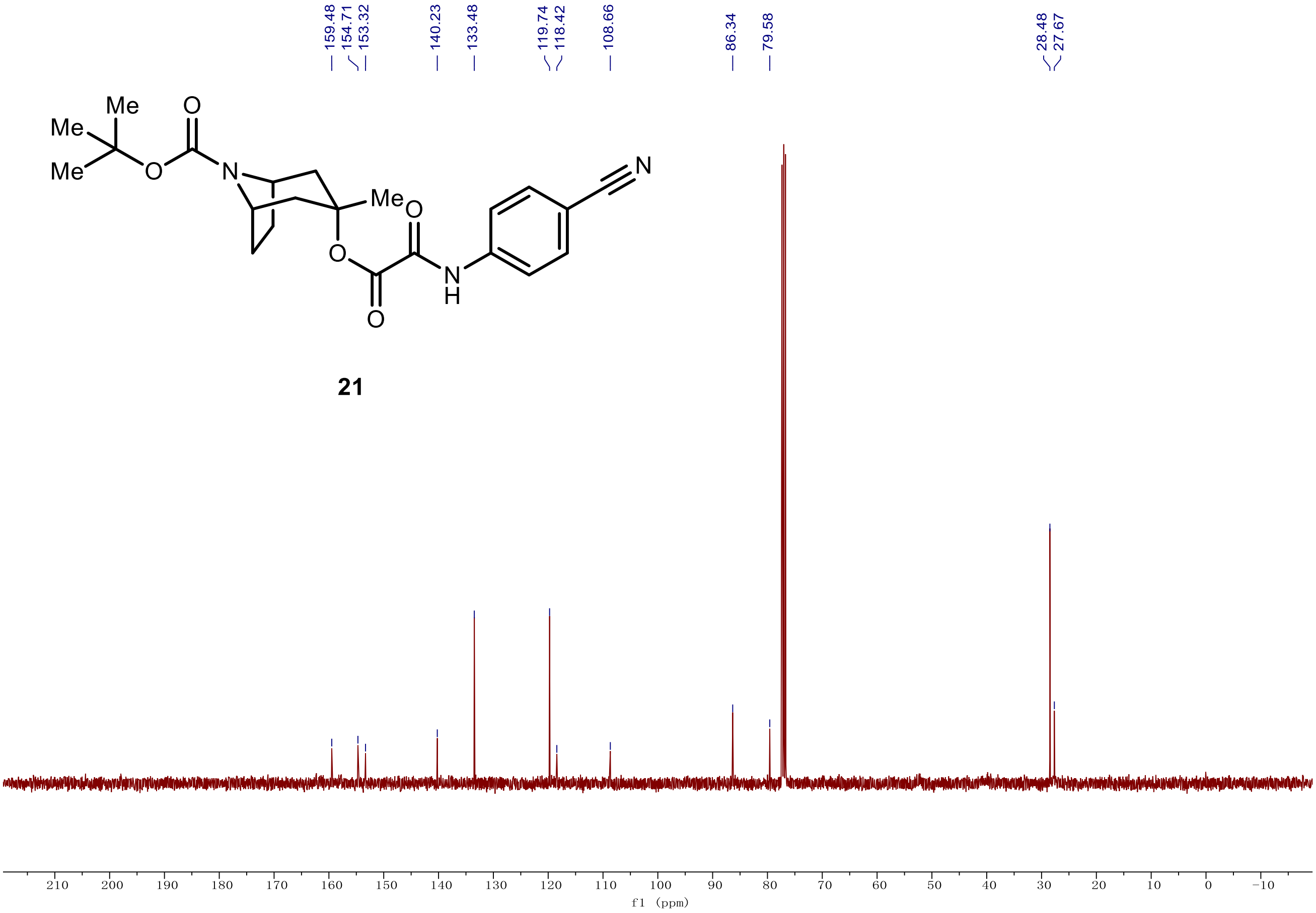

Figure S66 | ^{1}H NMR (400 MHz, CDCl$_3$) spectrum of **23**.

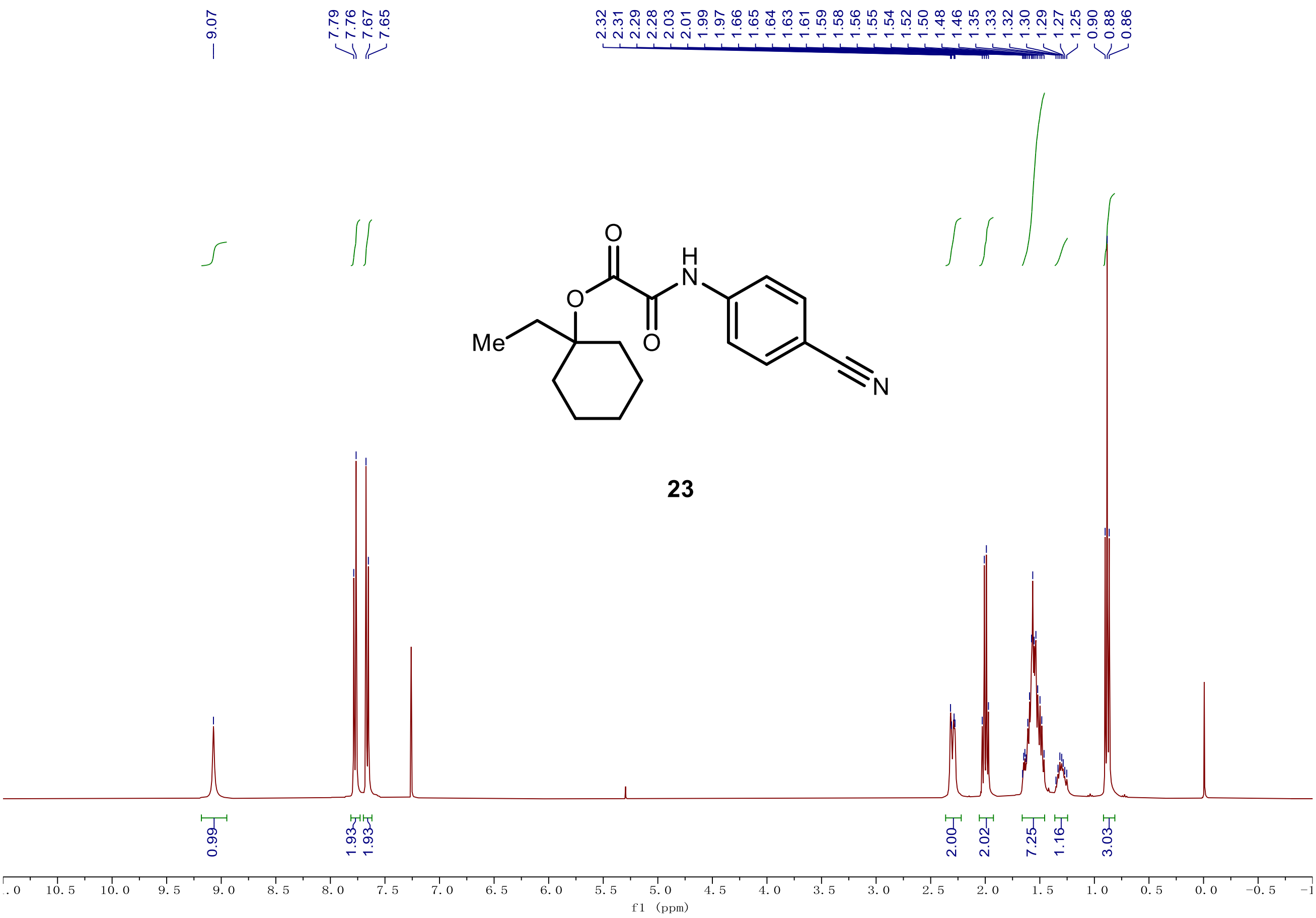

Figure S67 | ^{13}C NMR (101 MHz, $CDCl_3$) spectrum of **23**.

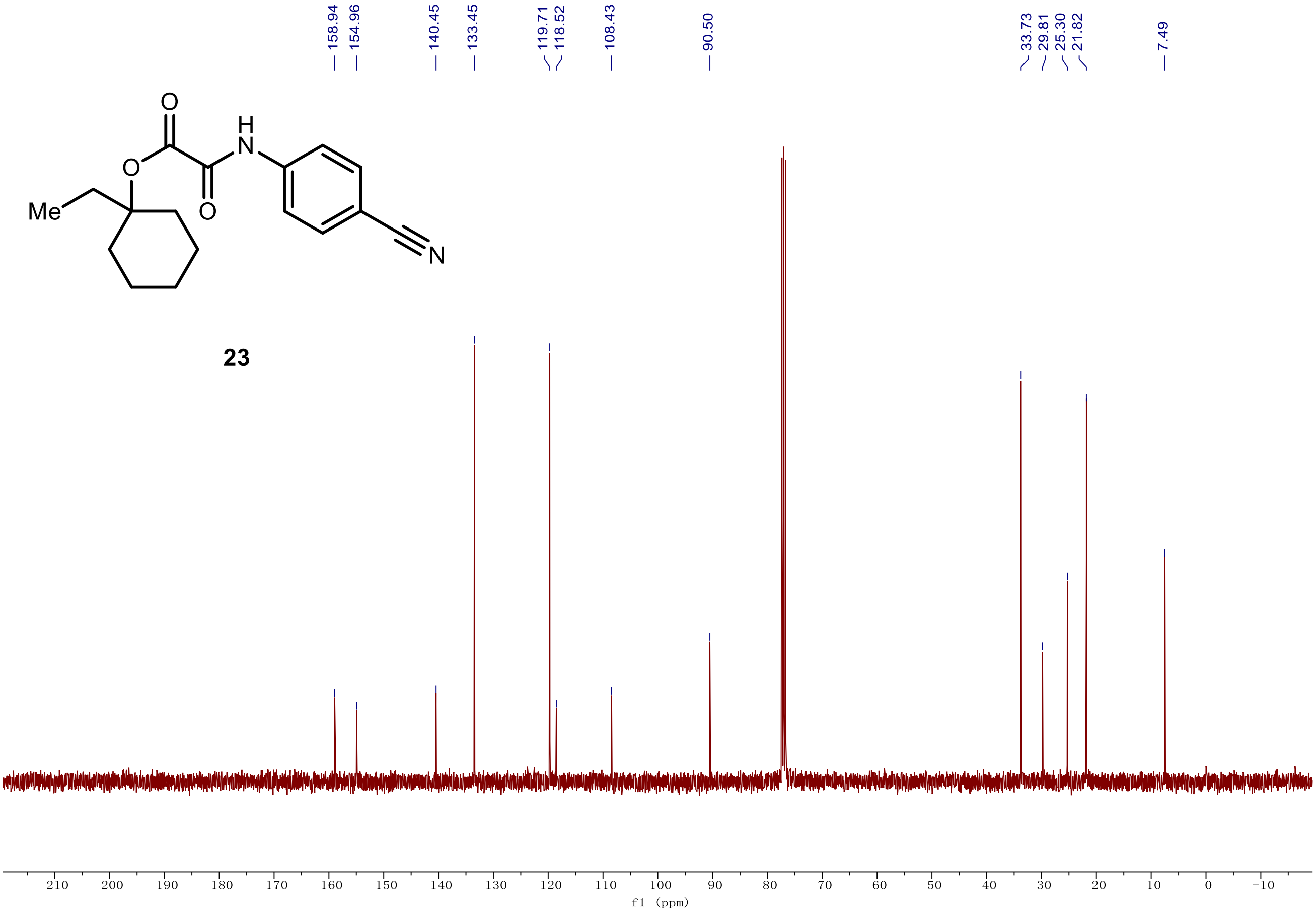

Figure S68 | ^{1}H NMR (400 MHz, $CDCl_3$) spectrum of **25**.

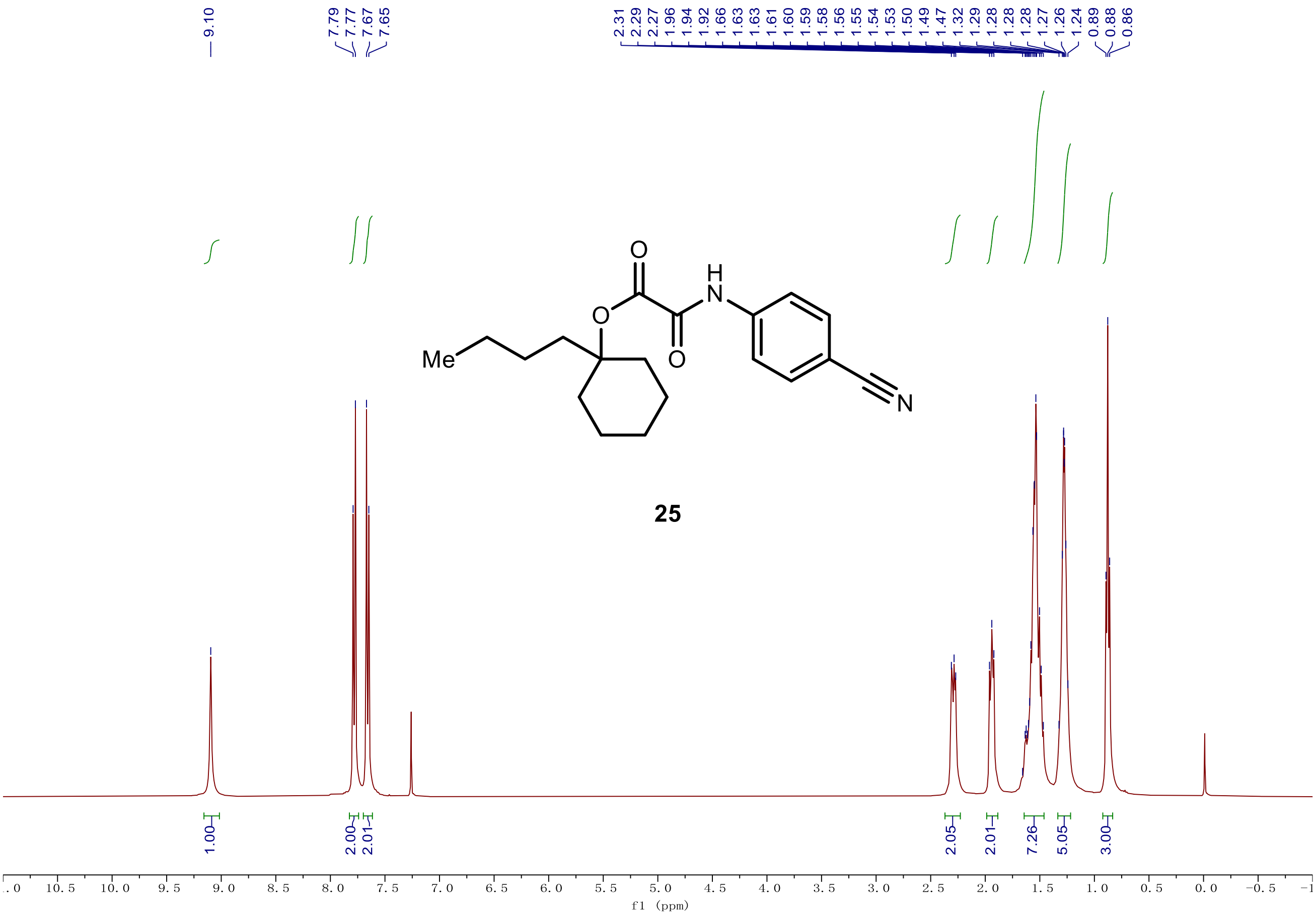

Figure S69 | ^{13}C NMR (101 MHz, $CDCl_3$) spectrum of **25**.

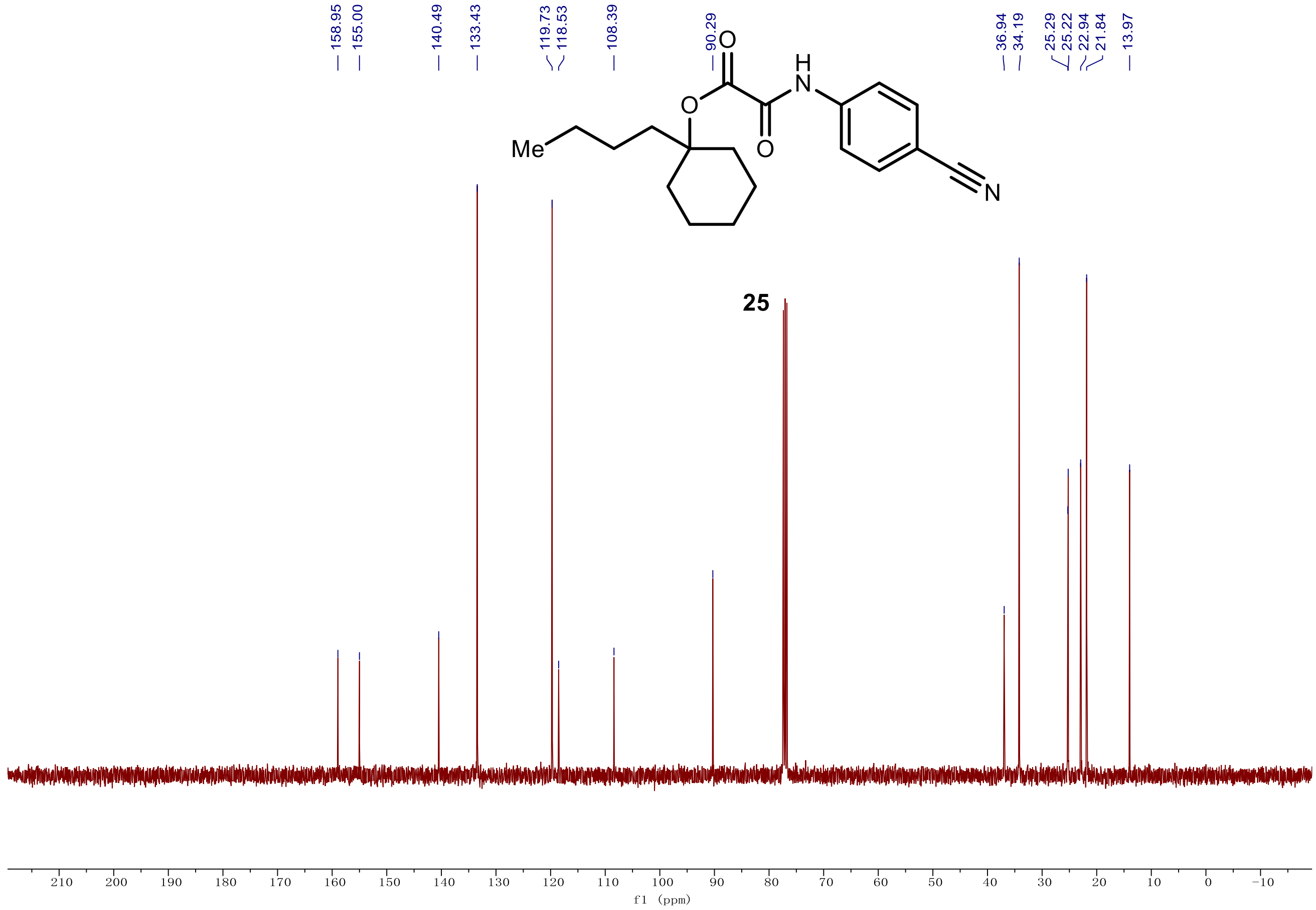

Figure S70 | ^{1}H NMR (400 MHz, $CDCl_3$) spectrum of **27**.

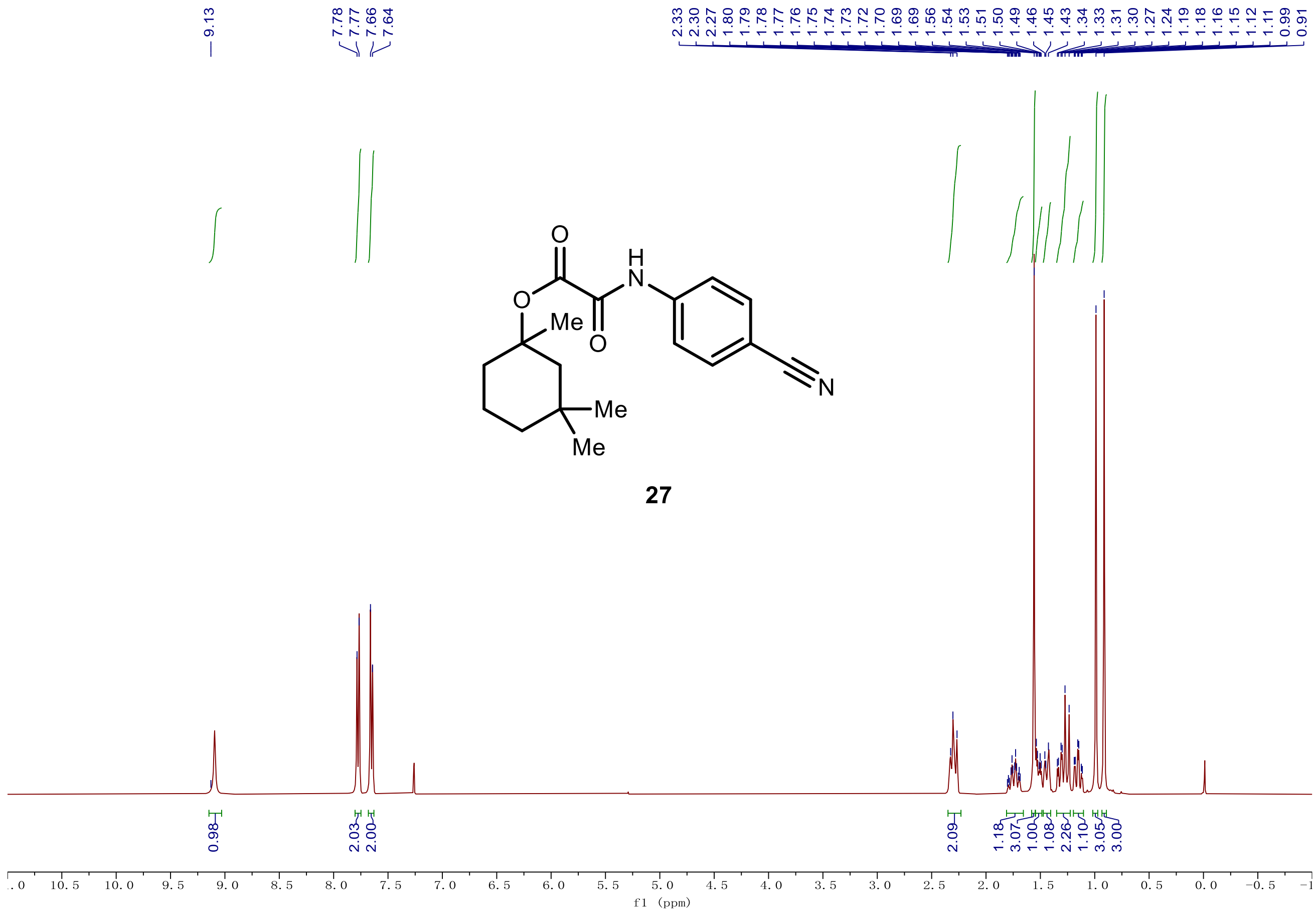

Figure S71 | ^{13}C NMR (101 MHz, $CDCl_3$) spectrum of **27**.

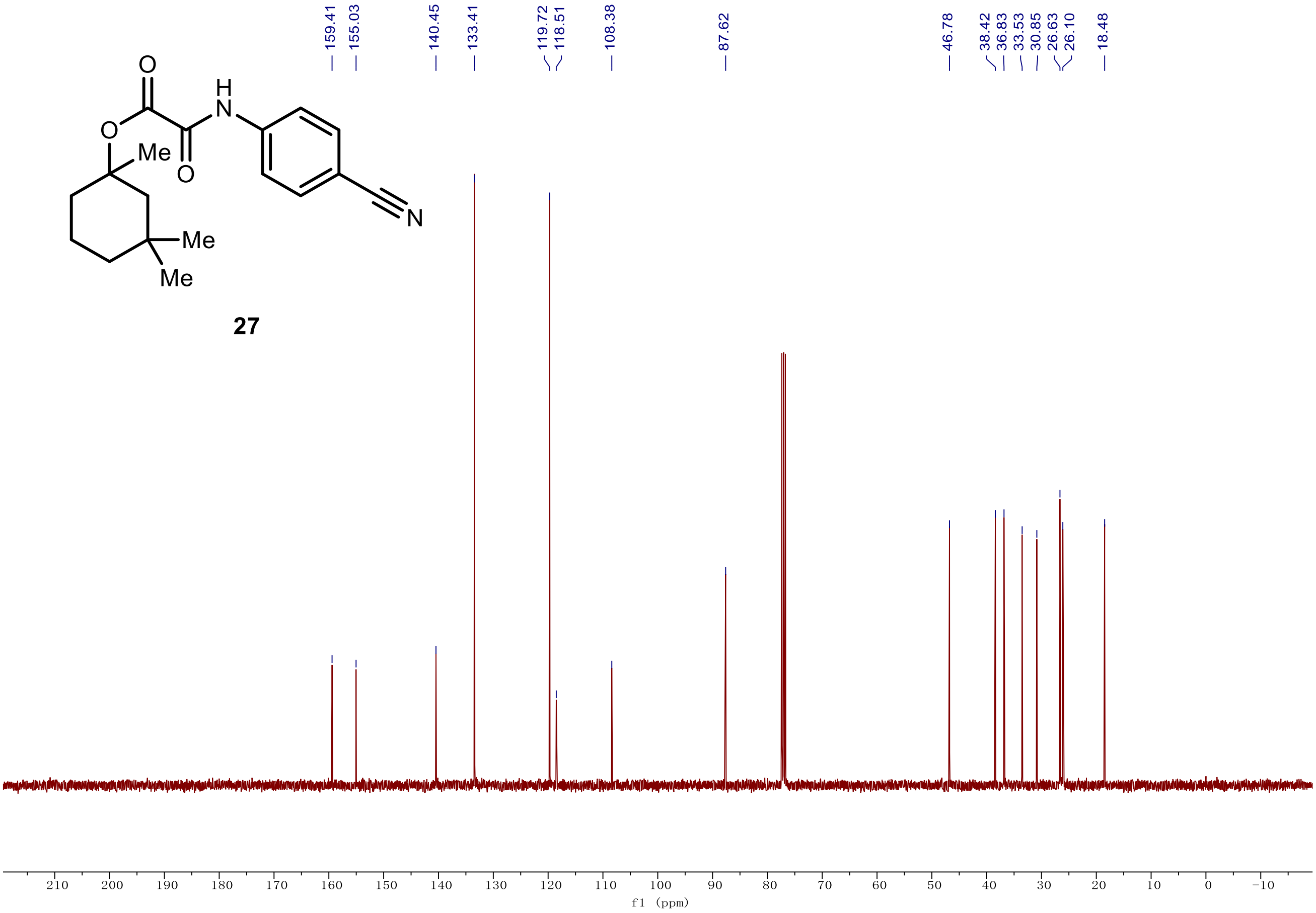

Figure S72 | ^{1}H NMR (400 MHz, $CDCl_3$) spectrum of **29**.

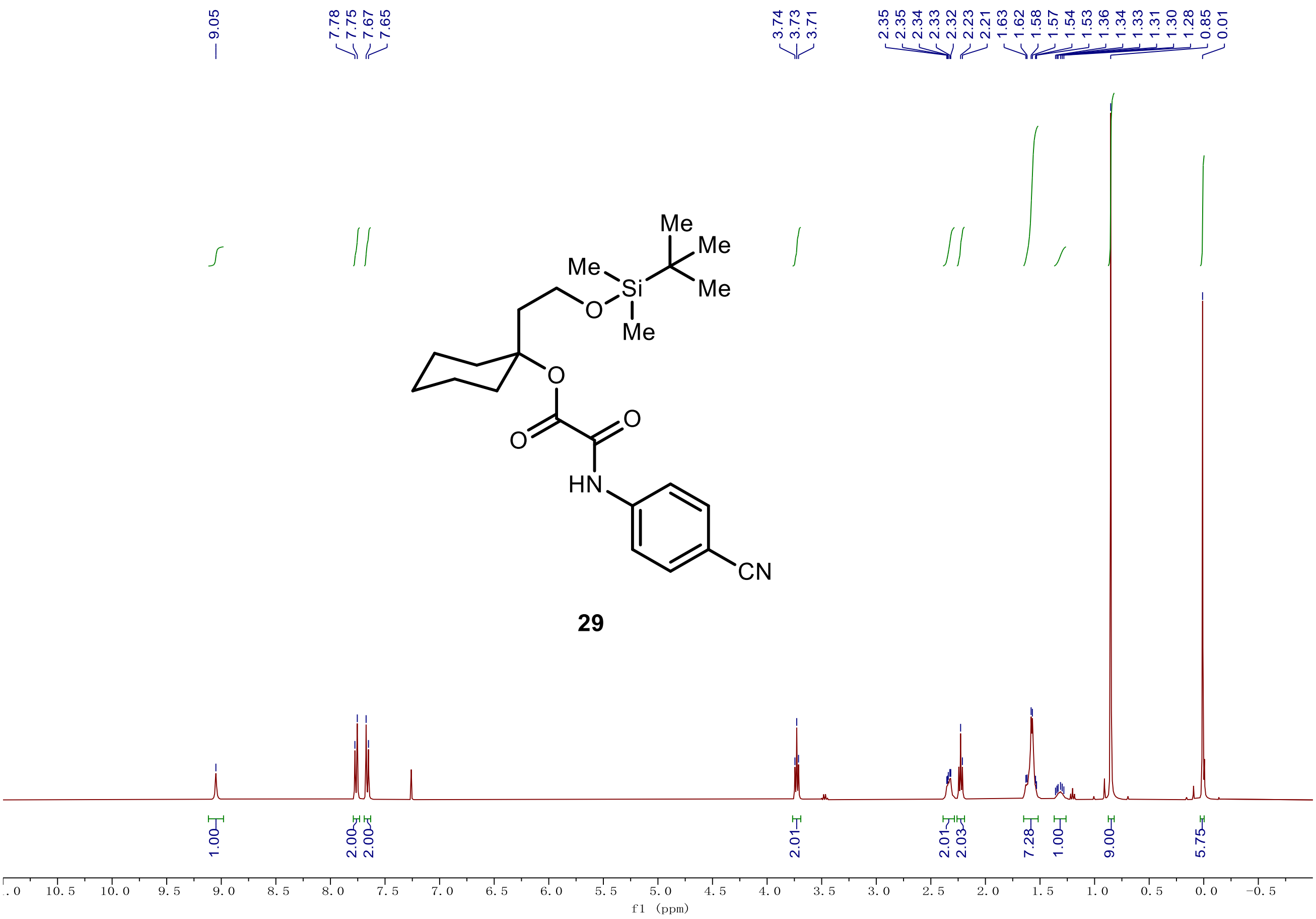

Figure S73 | ^{13}C NMR (101 MHz, $CDCl_3$) spectrum of **29**.

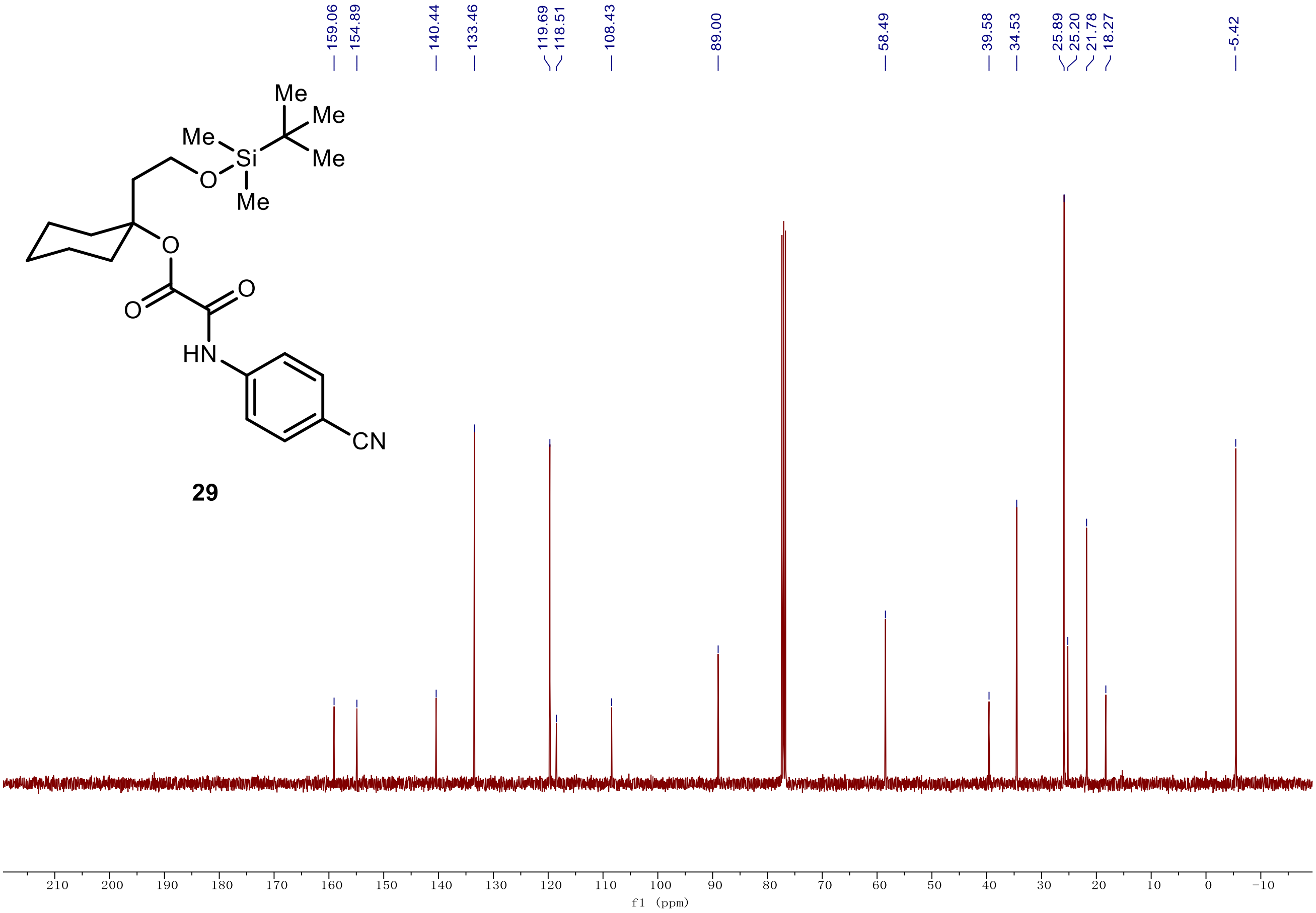

Figure S74 | ^{1}H NMR (400 MHz, $CDCl_3$) spectrum of **31**.

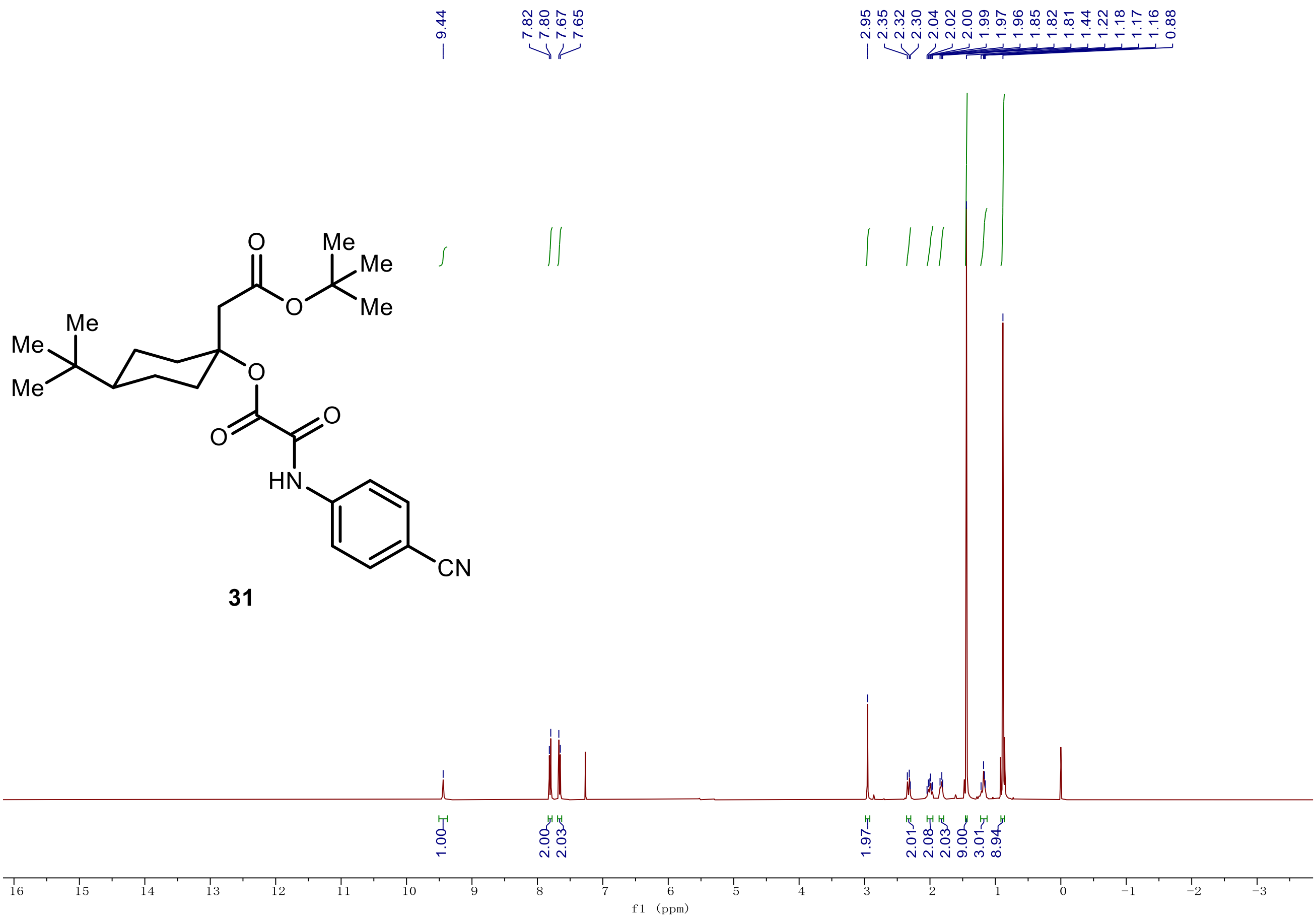

Figure S75 | ^{13}C NMR (101 MHz, $CDCl_3$) spectrum of **31**.

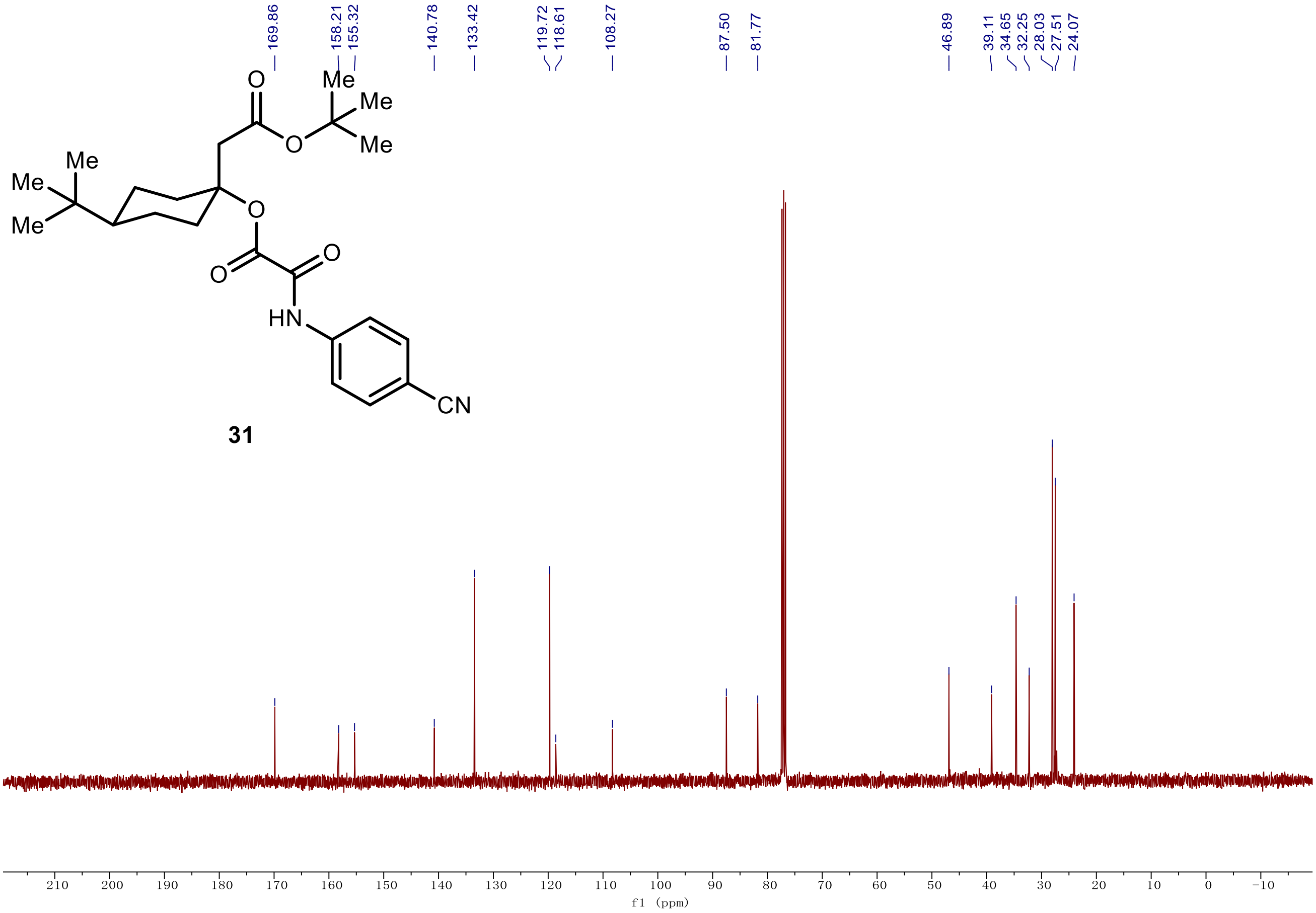

Figure S76 | ^{1}H NMR (400 MHz, $CDCl_3$) spectrum of **33**.

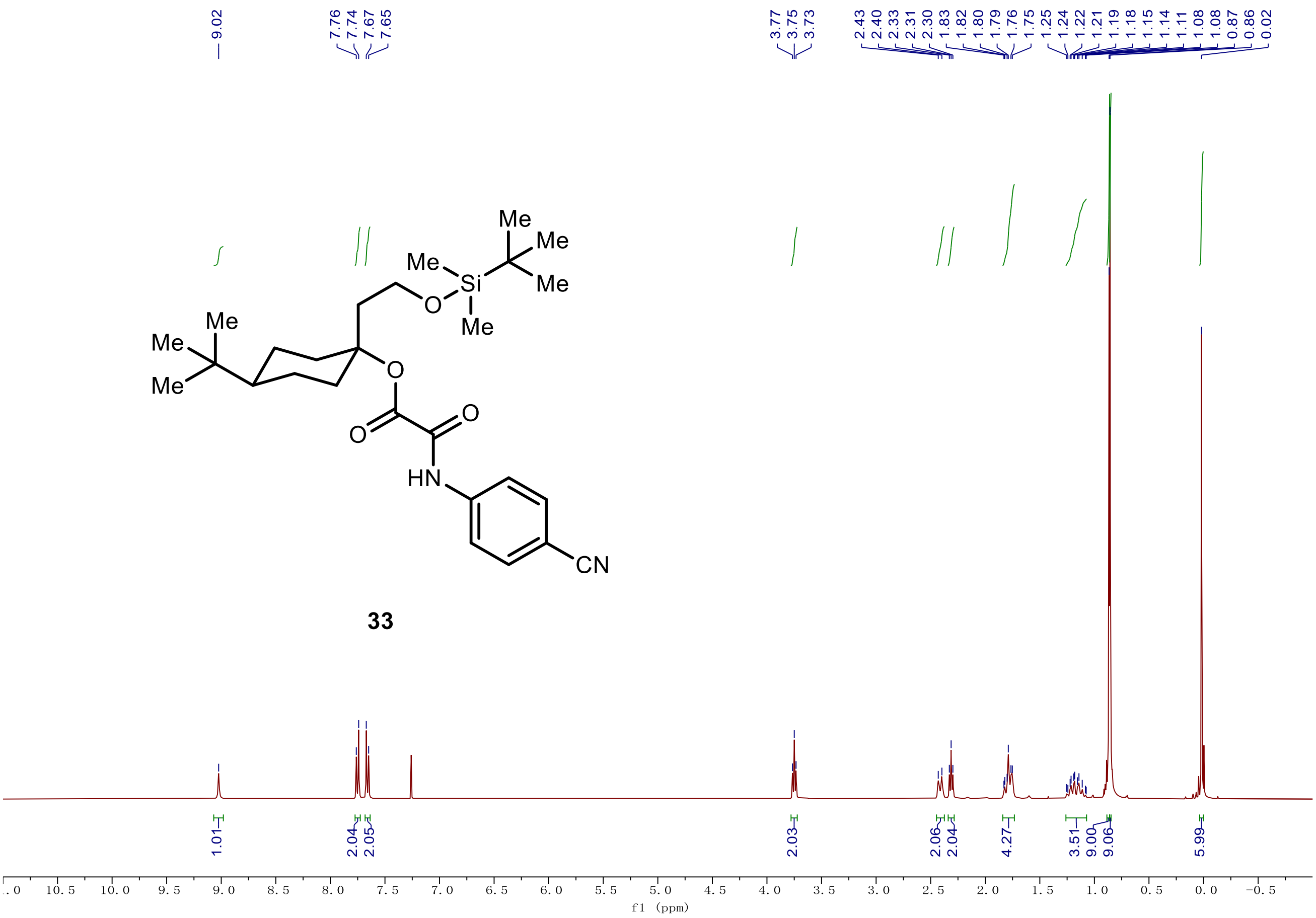

Figure S77 | ^{13}C NMR (101 MHz, $CDCl_3$) spectrum of **33**.

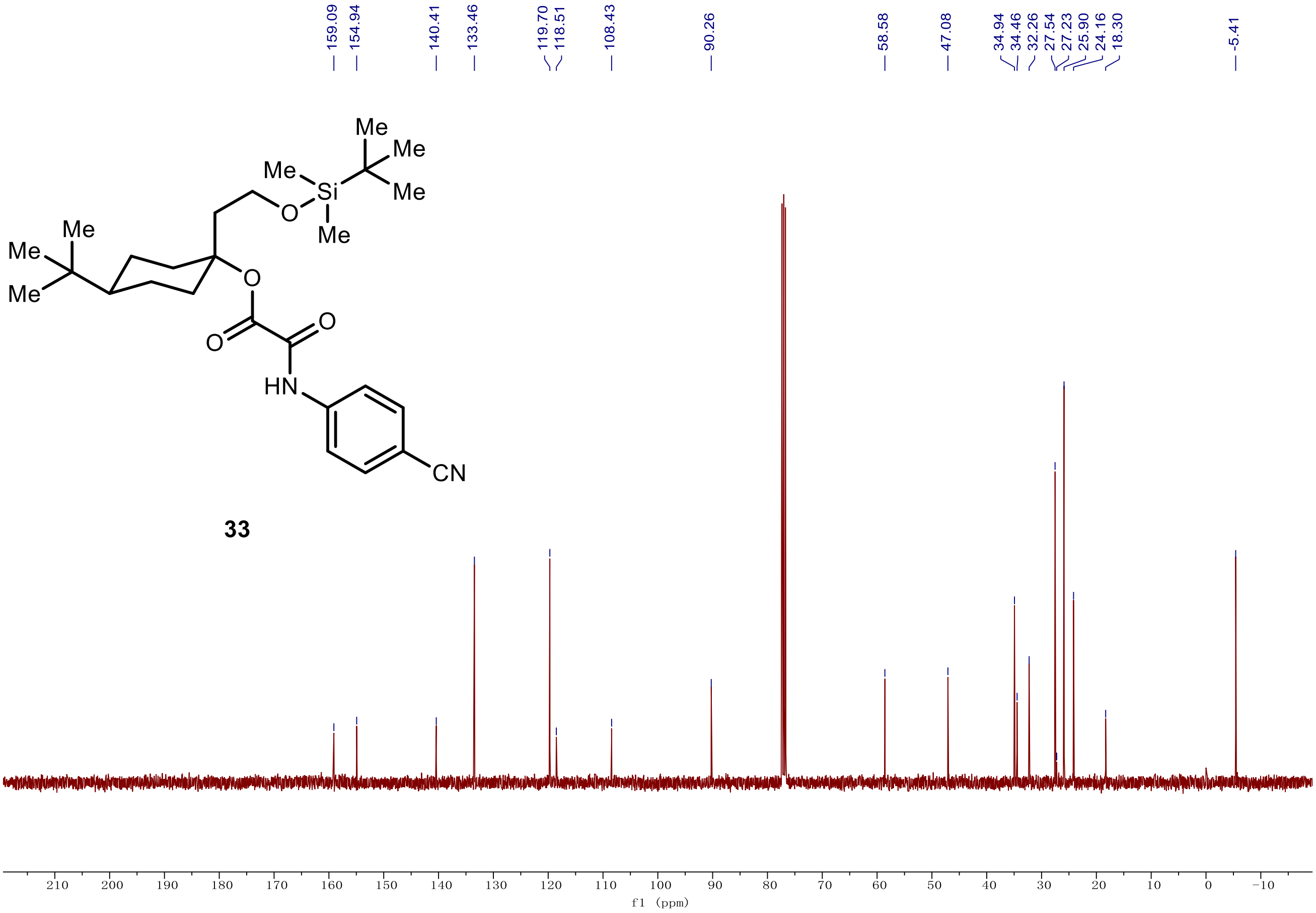

Figure S78 | ^{1}H NMR (400 MHz, $CDCl_3$) spectrum of **35**.

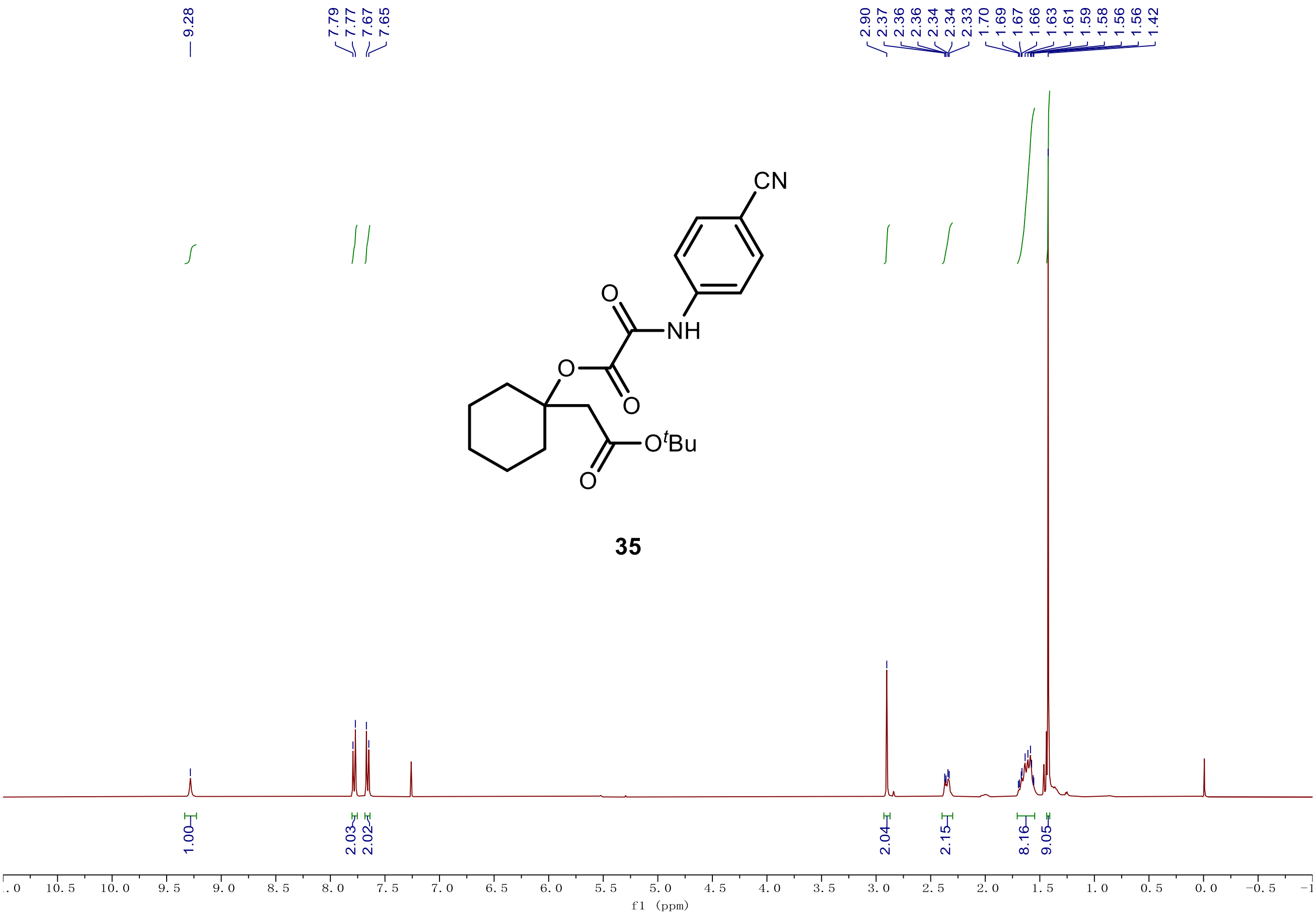

Figure S79 | ^{13}C NMR (101 MHz, CDCl$_3$) spectrum of **35**.

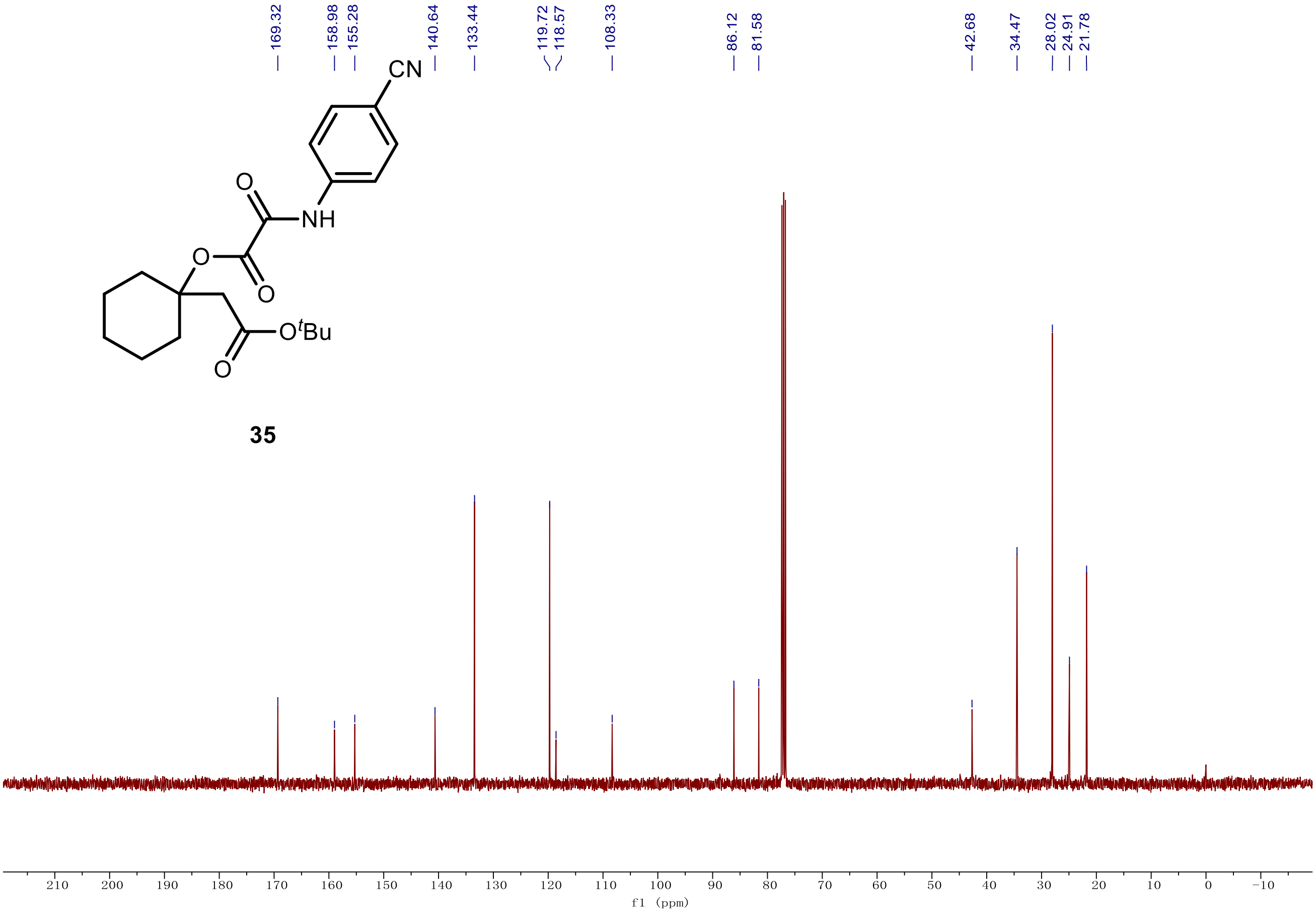

Figure S80 | ^{1}H NMR (400 MHz, CDCl$_3$) spectrum of **37**.

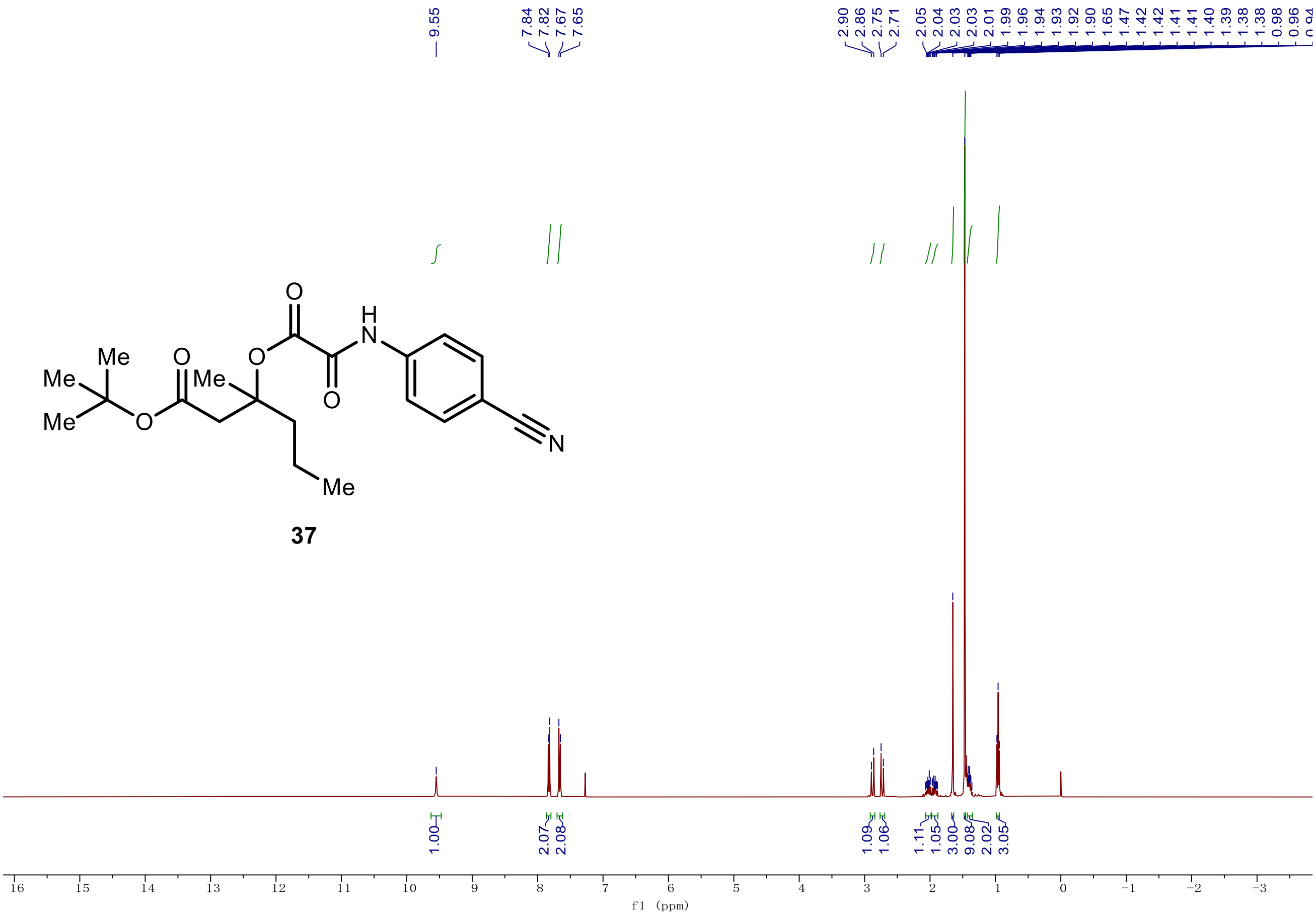

Figure S81 | ^{13}C NMR (101 MHz, CDCl$_3$) spectrum of **37**.

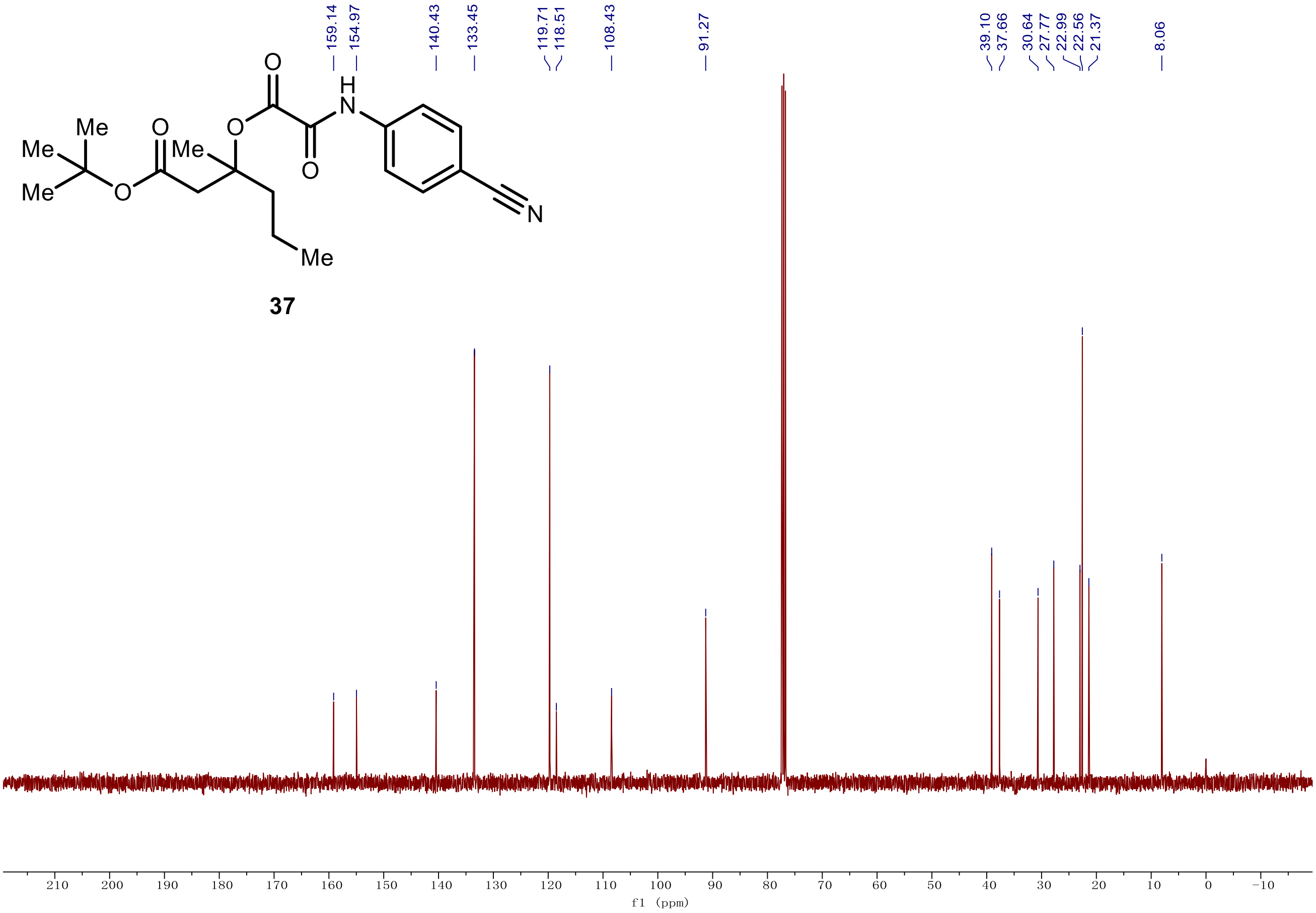

Figure S82 | ^{1}H NMR (400 MHz, CDCl$_3$) spectrum of **39**.

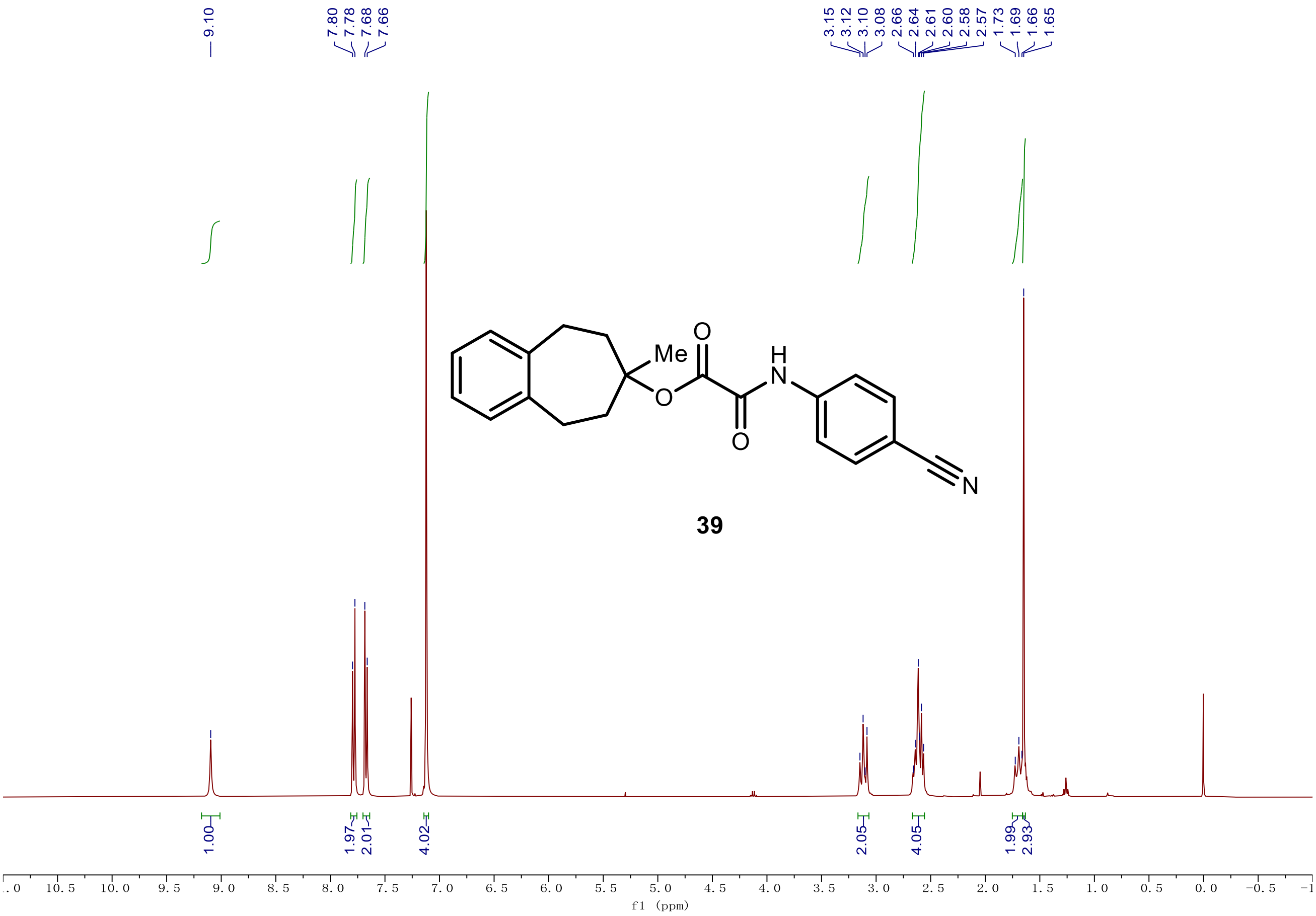

Figure S83 | ^{13}C NMR (101 MHz, CDCl$_3$) spectrum of **39**.

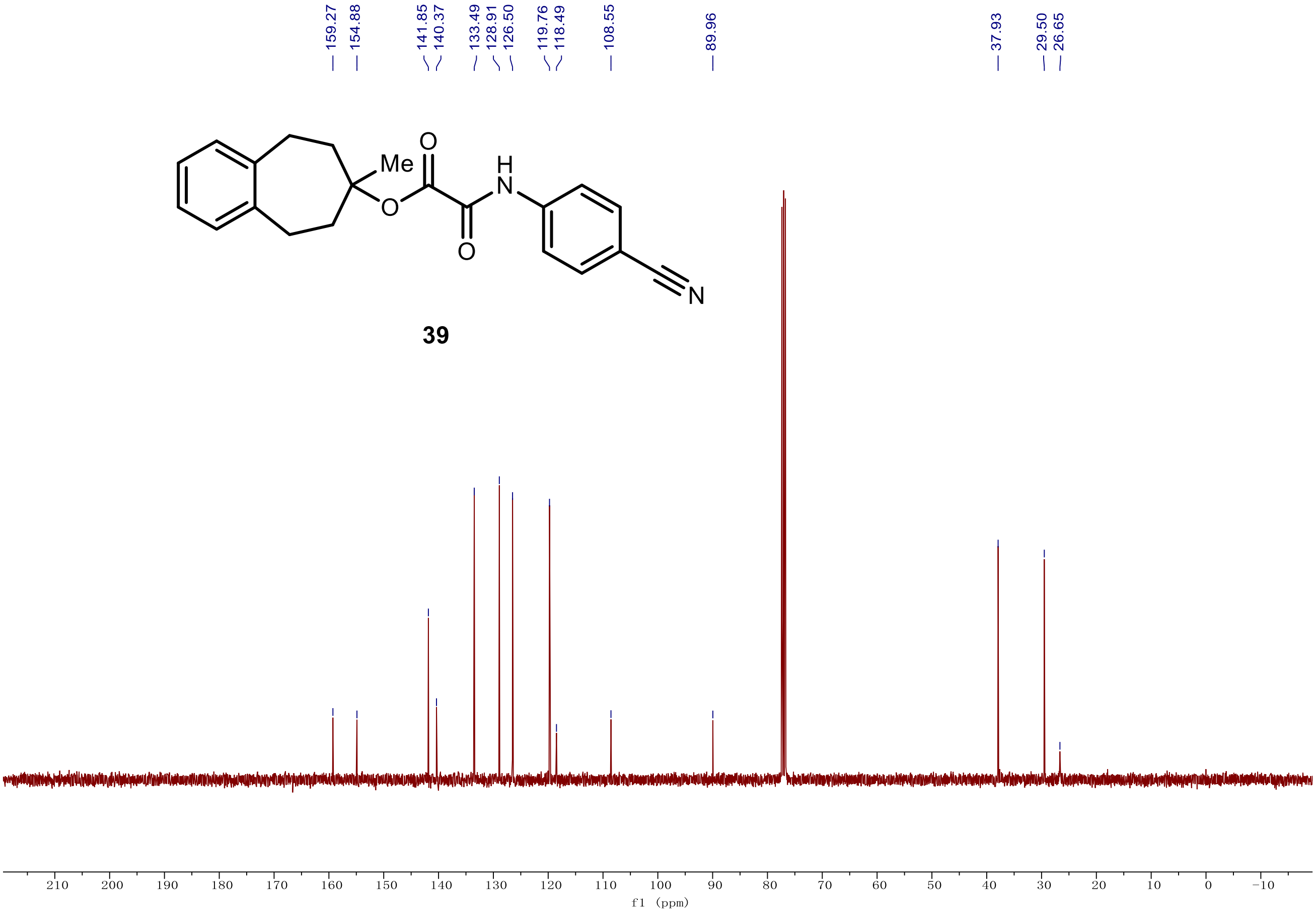

Figure S84 | ^{1}H NMR (400 MHz, CDCl$_3$) spectrum of **41**.

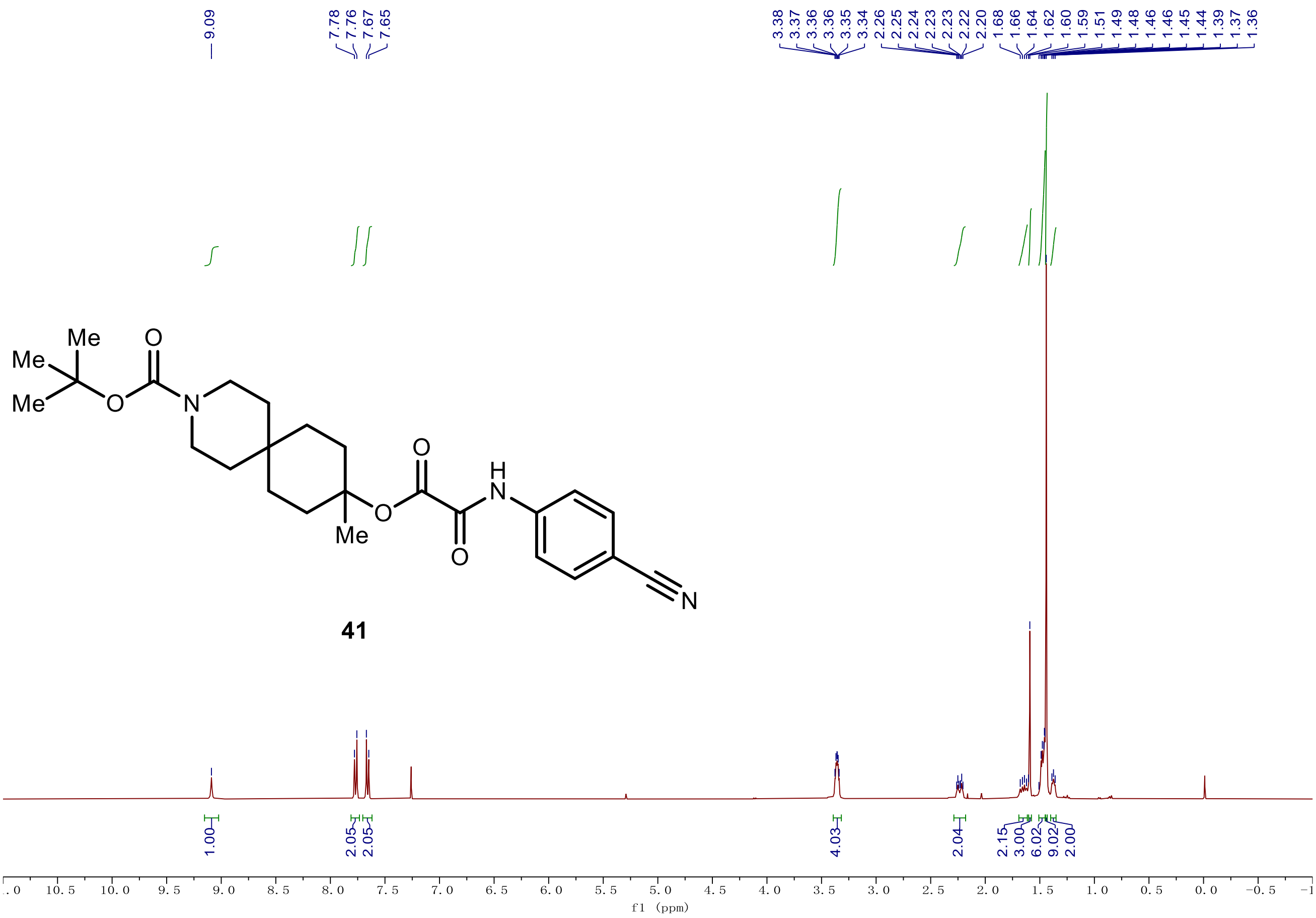

Figure S85 | ^{13}C NMR (101 MHz, $CDCl_3$) spectrum of **41**.

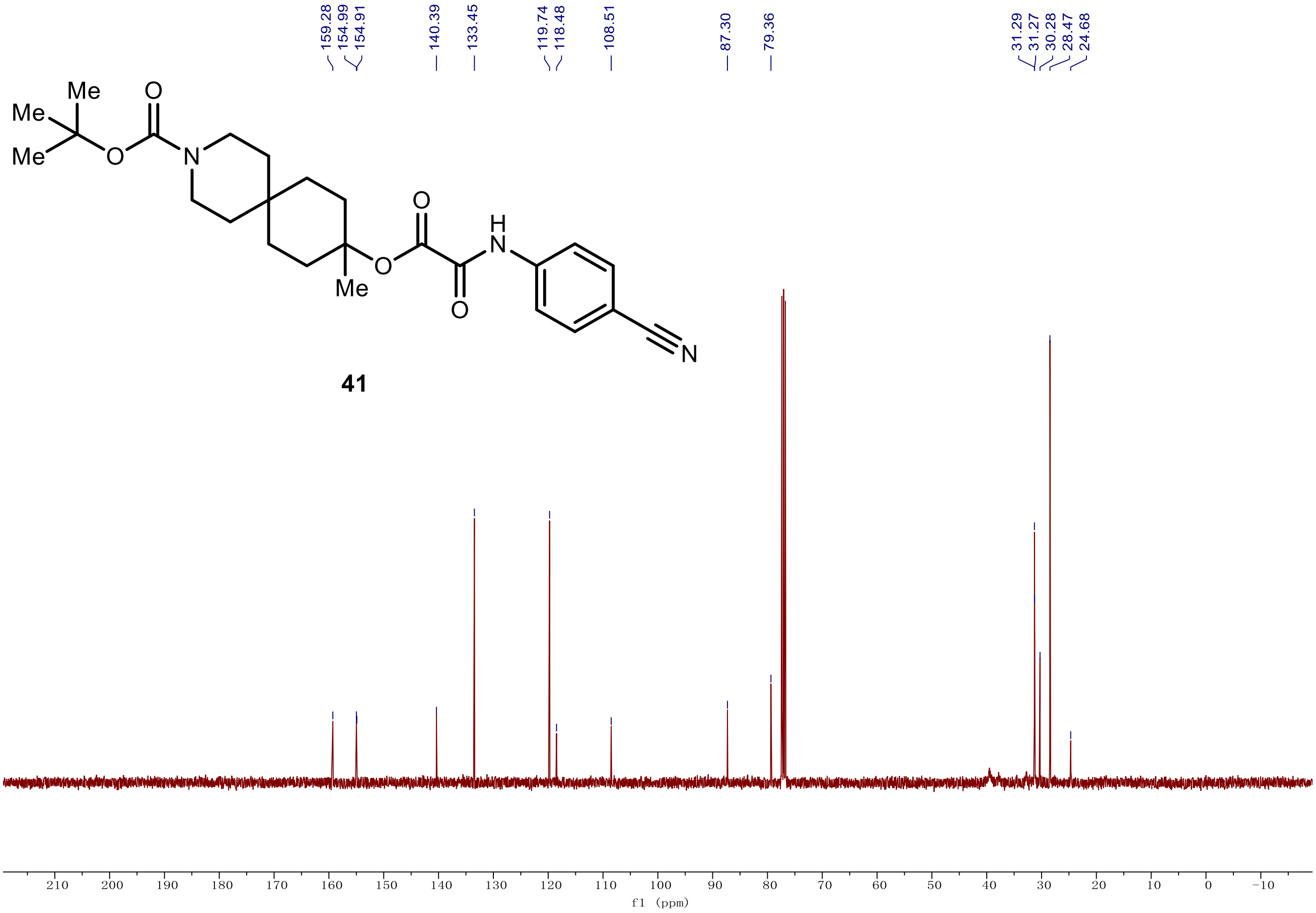

Figure S86 | ^{1}H NMR (500 MHz, $CDCl_3$) spectrum of **49**.

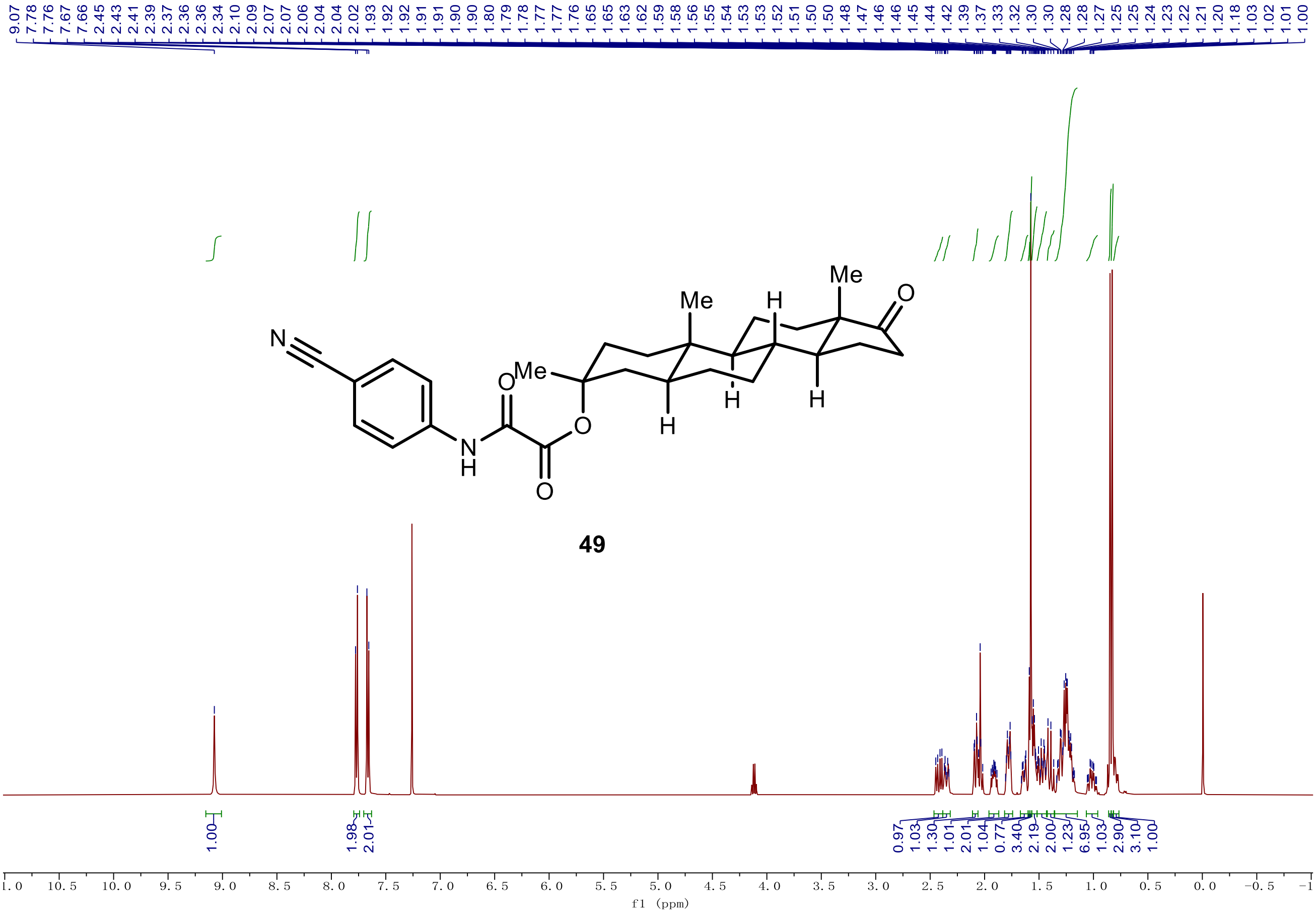

Figure S87 | ^{13}C NMR (126 MHz, CDCl$_3$) spectrum of **49**.

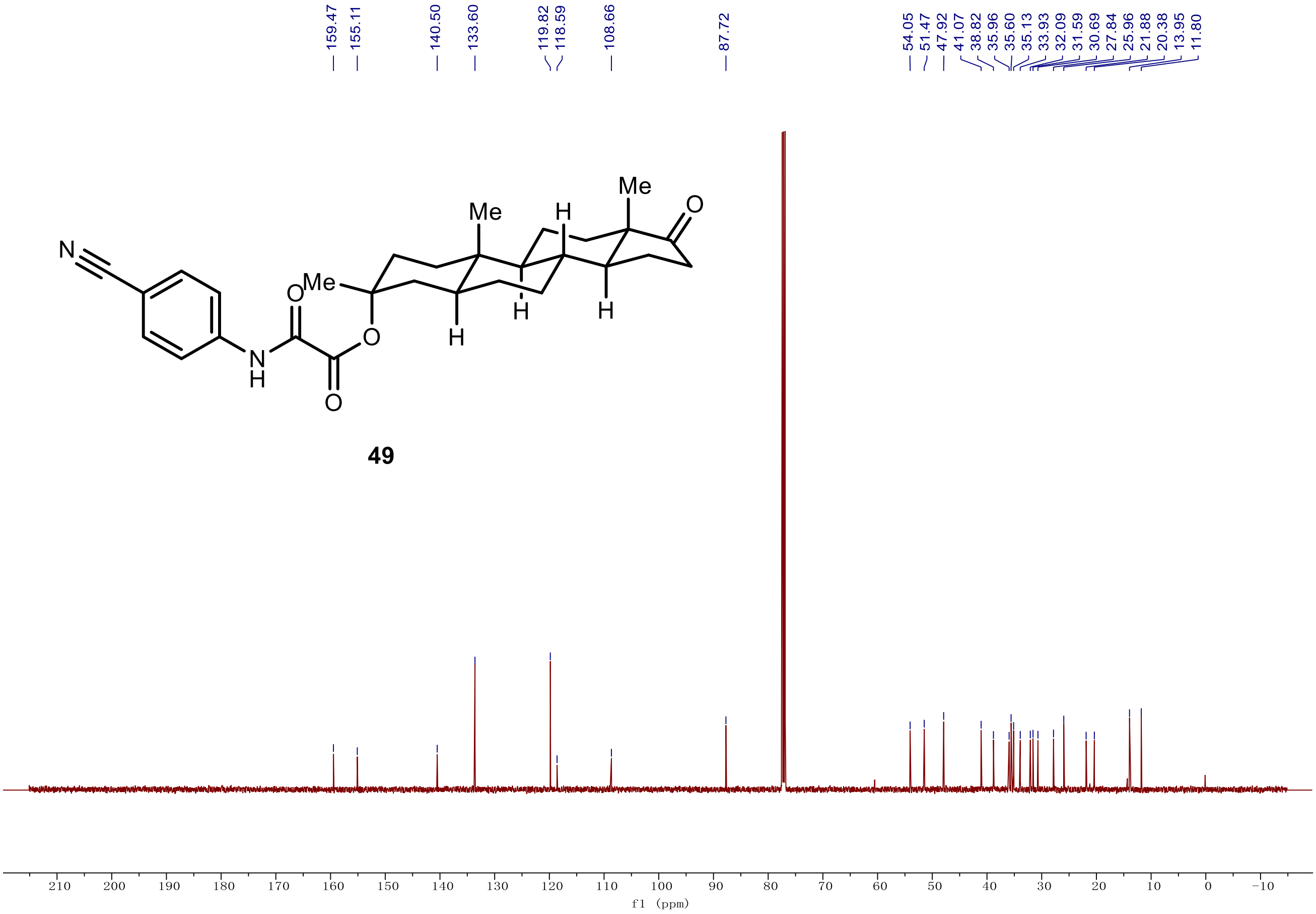

Figure S88 | ^{1}H NMR (600 MHz, CDCl$_3$) spectrum of **2ma**.

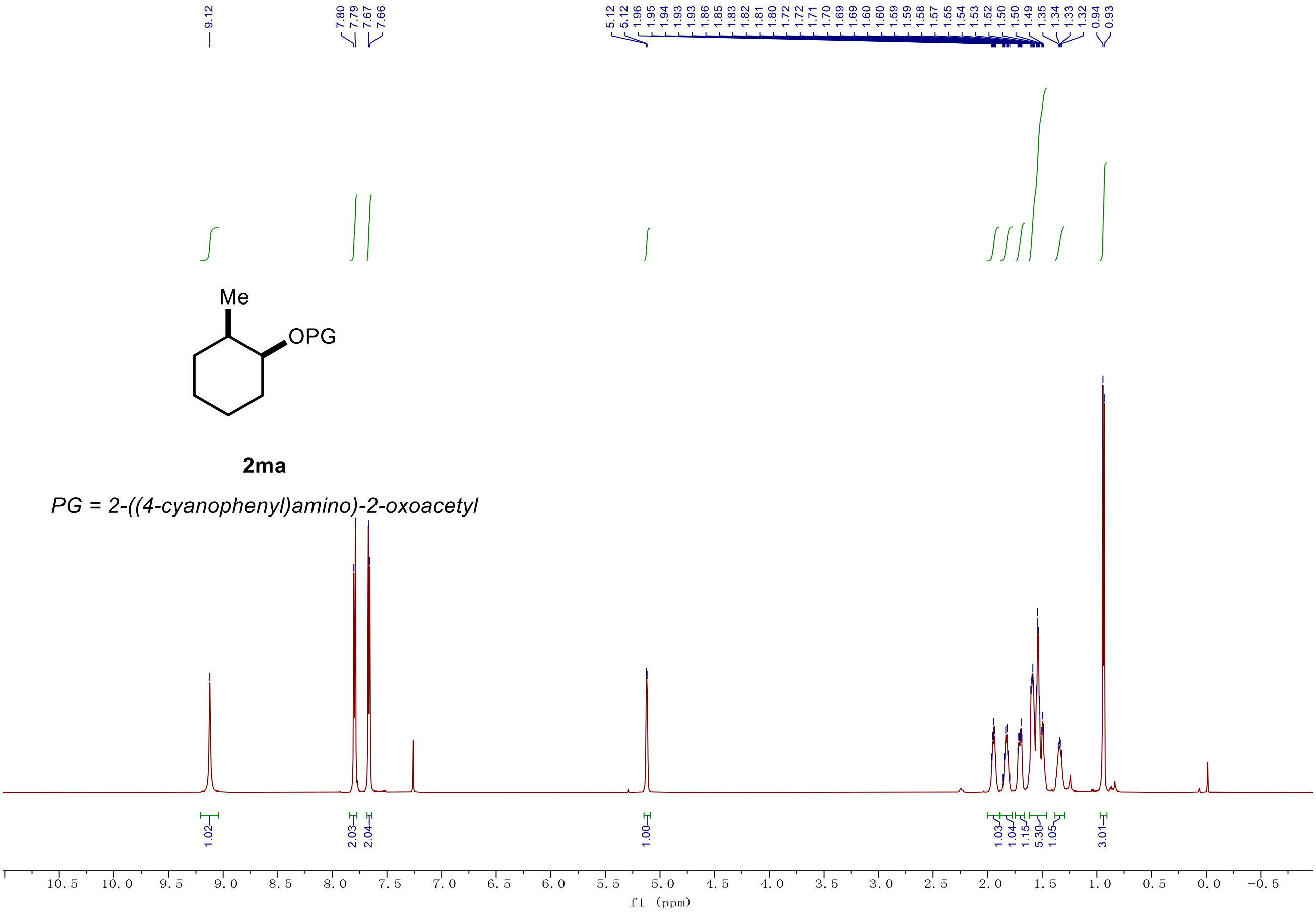

Figure S89 | ^{13}C NMR (151 MHz, $CDCl_3$) spectrum of **2ma**.

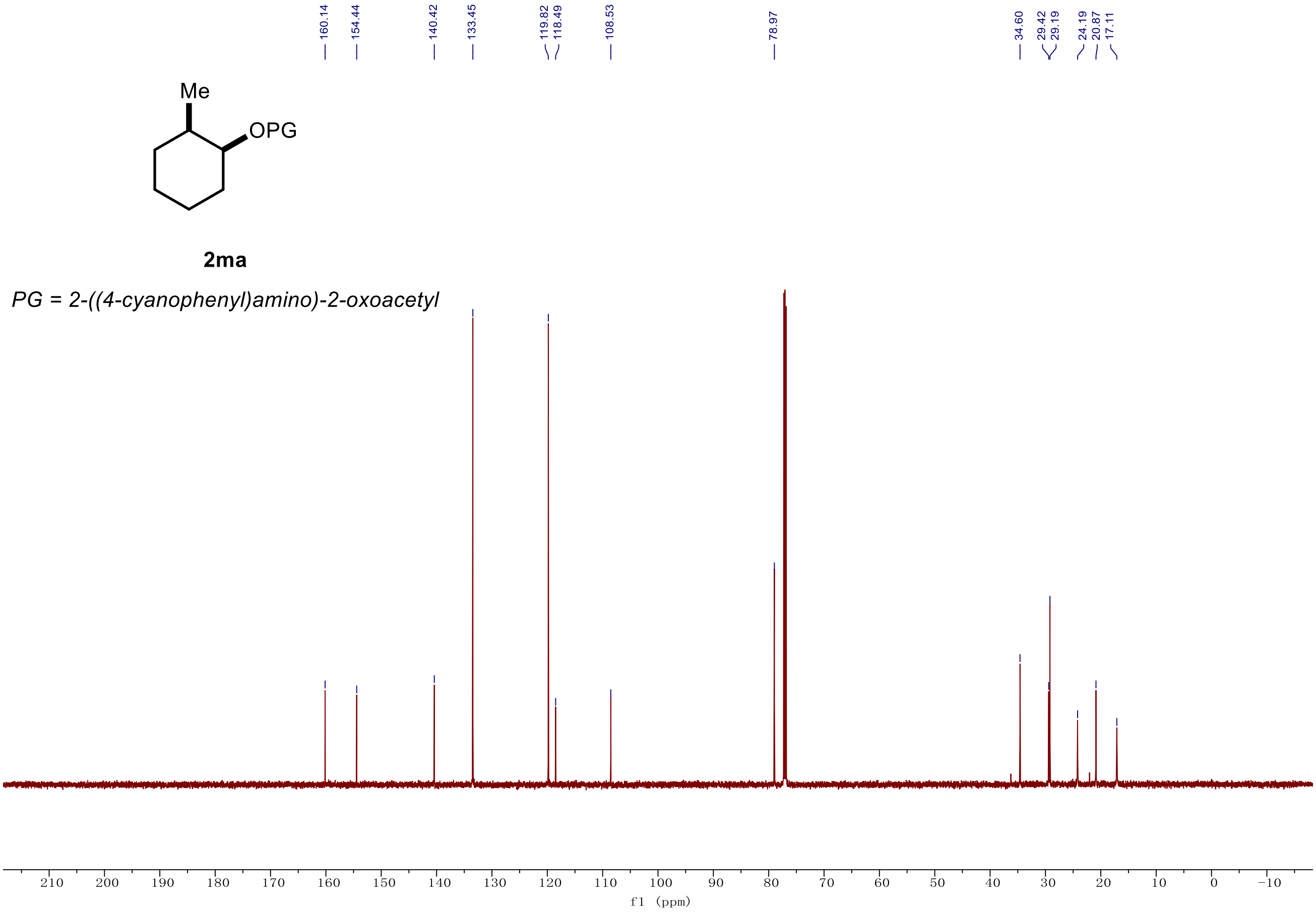

Figure S90 | ^{1}H NMR (600 MHz, $CDCl_3$) spectrum of **2mb**.

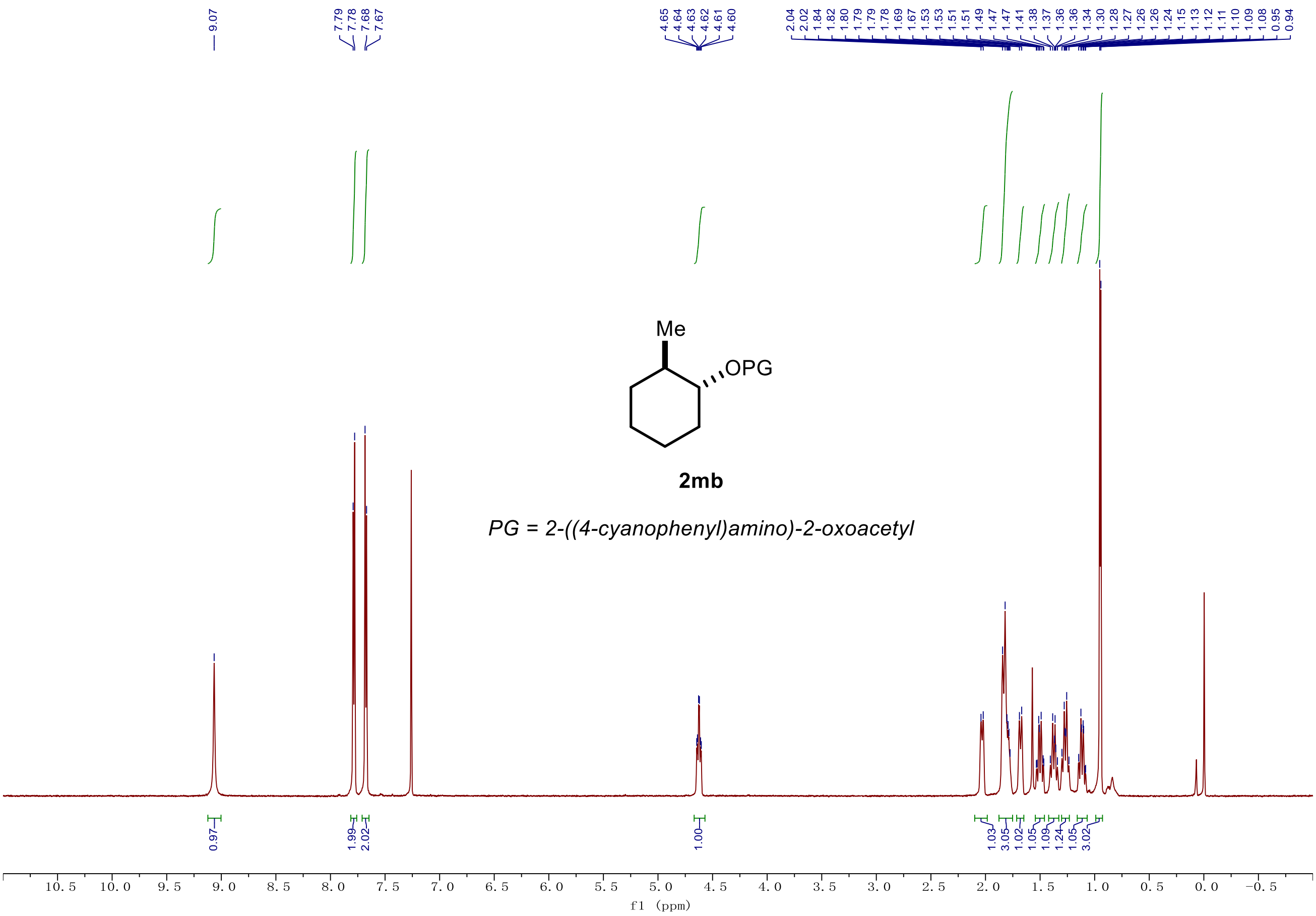

Figure S91 | ^{13}C NMR (151 MHz, $CDCl_3$) spectrum of **2mb**.

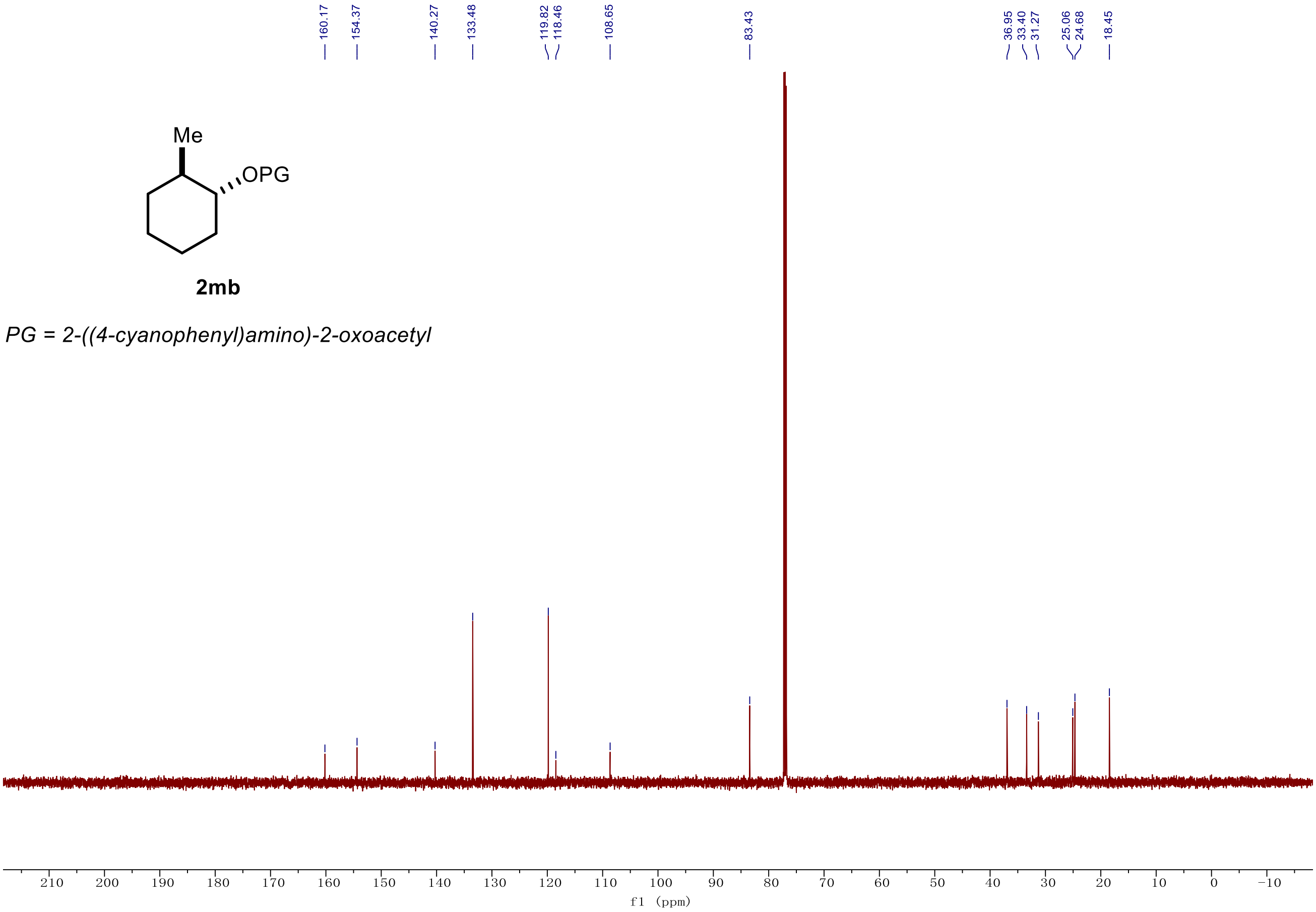

Figure S92 | ^{1}H NMR (400 MHz, $CDCl_3$) spectrum of **4**.

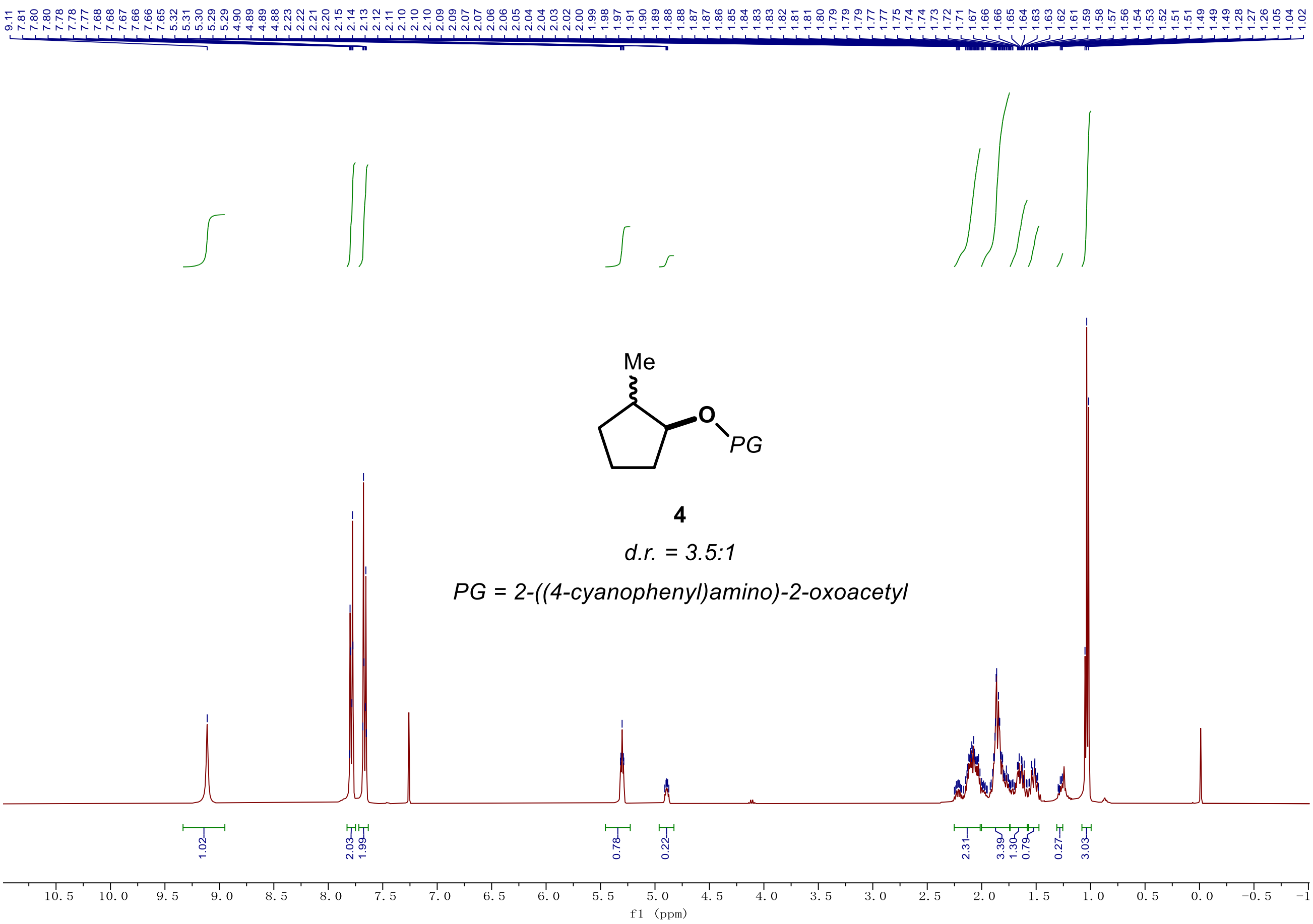

Figure S93 | ^{13}C NMR (101 MHz, $CDCl_3$) spectrum of **4**.

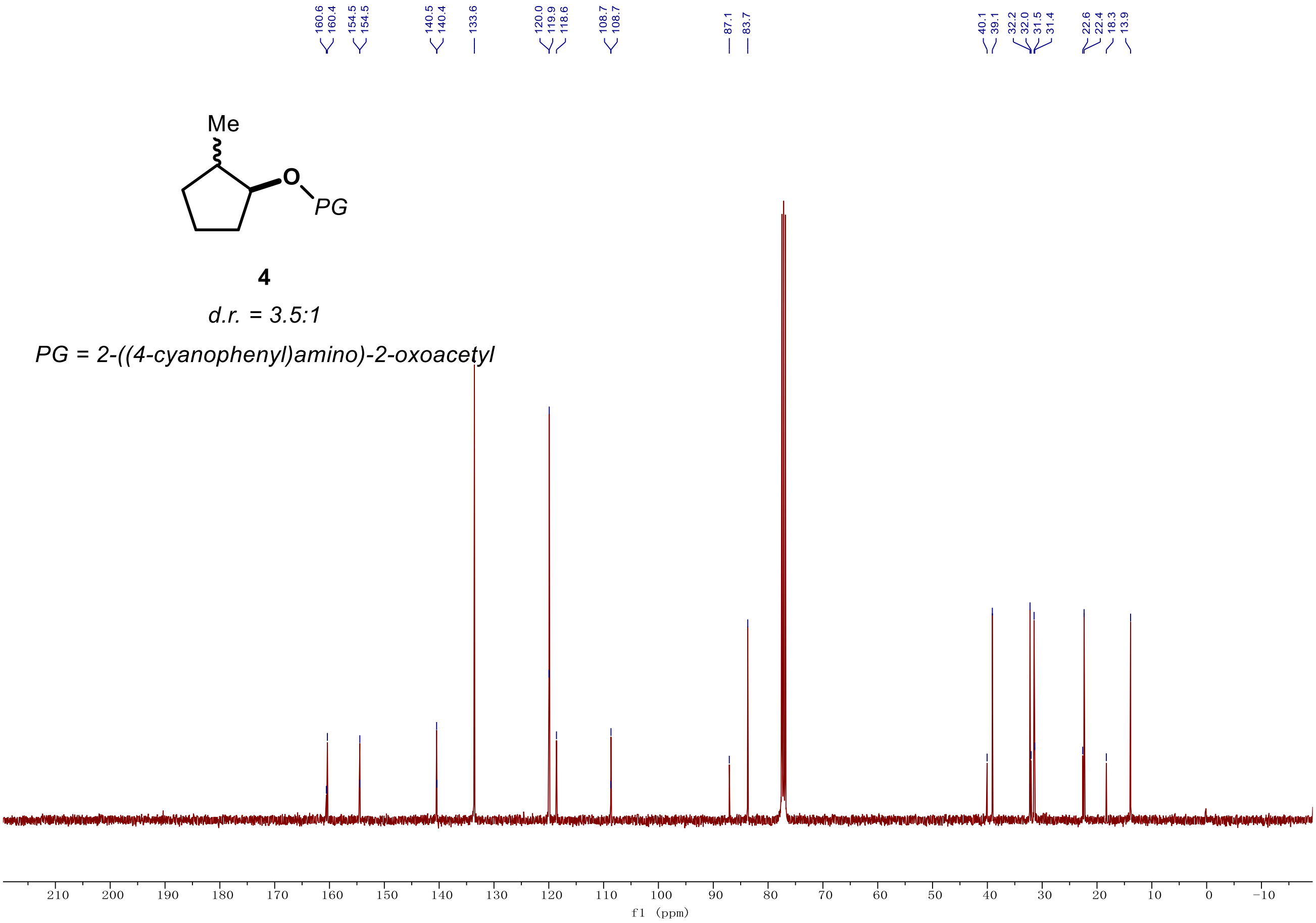

Figure S94 | ^{1}H NMR (400 MHz, $CDCl_3$) spectrum of **6-*diastereomer 1***.

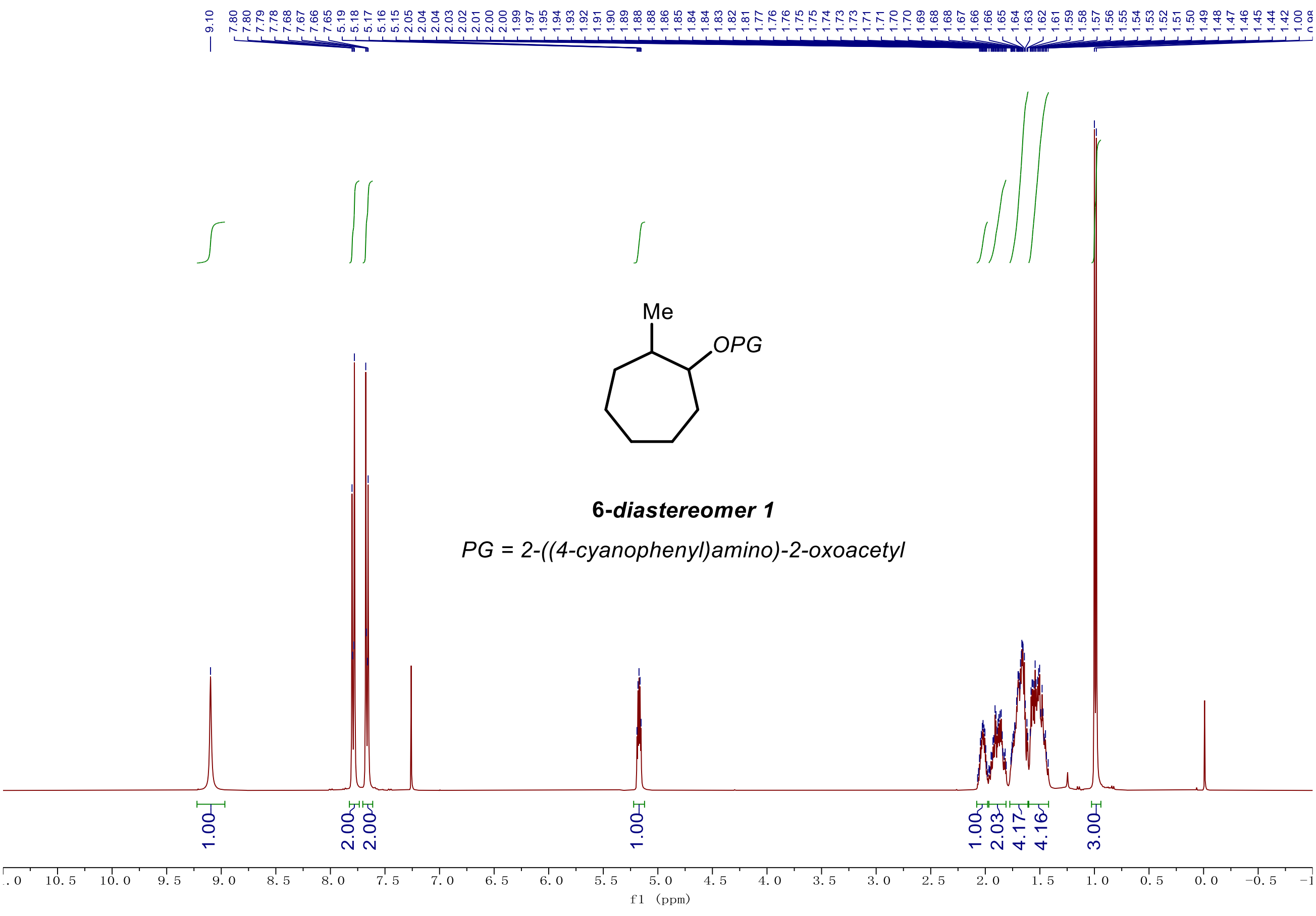

Figure S95 | ^{13}C NMR (101 MHz, $CDCl_3$) spectrum of **6-*diastereomer 1***.

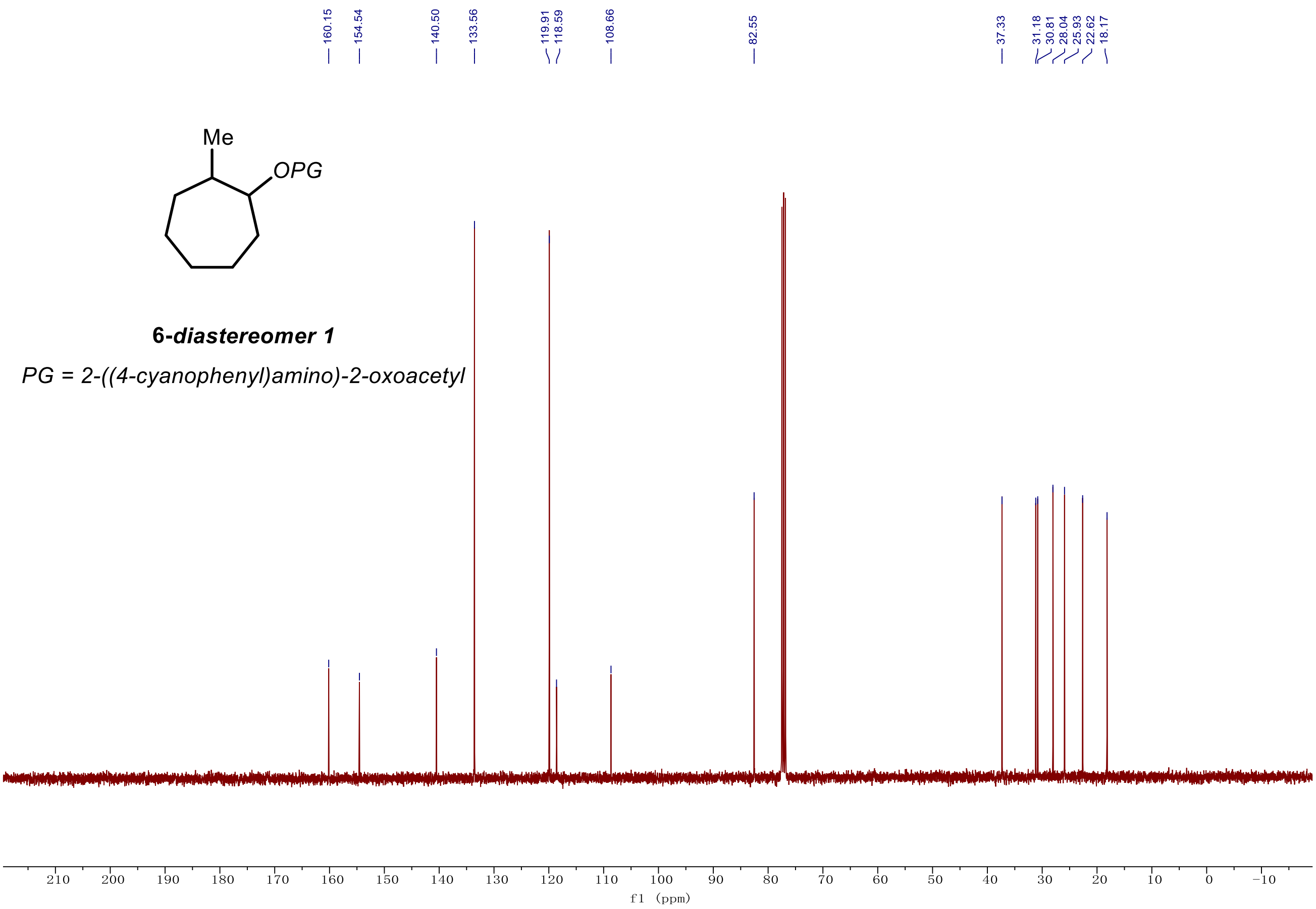

Figure S96 | ^{1}H NMR (400 MHz, $CDCl_3$) spectrum of **6-*diastereomer 2*** (mixed with 6% of **6'**).

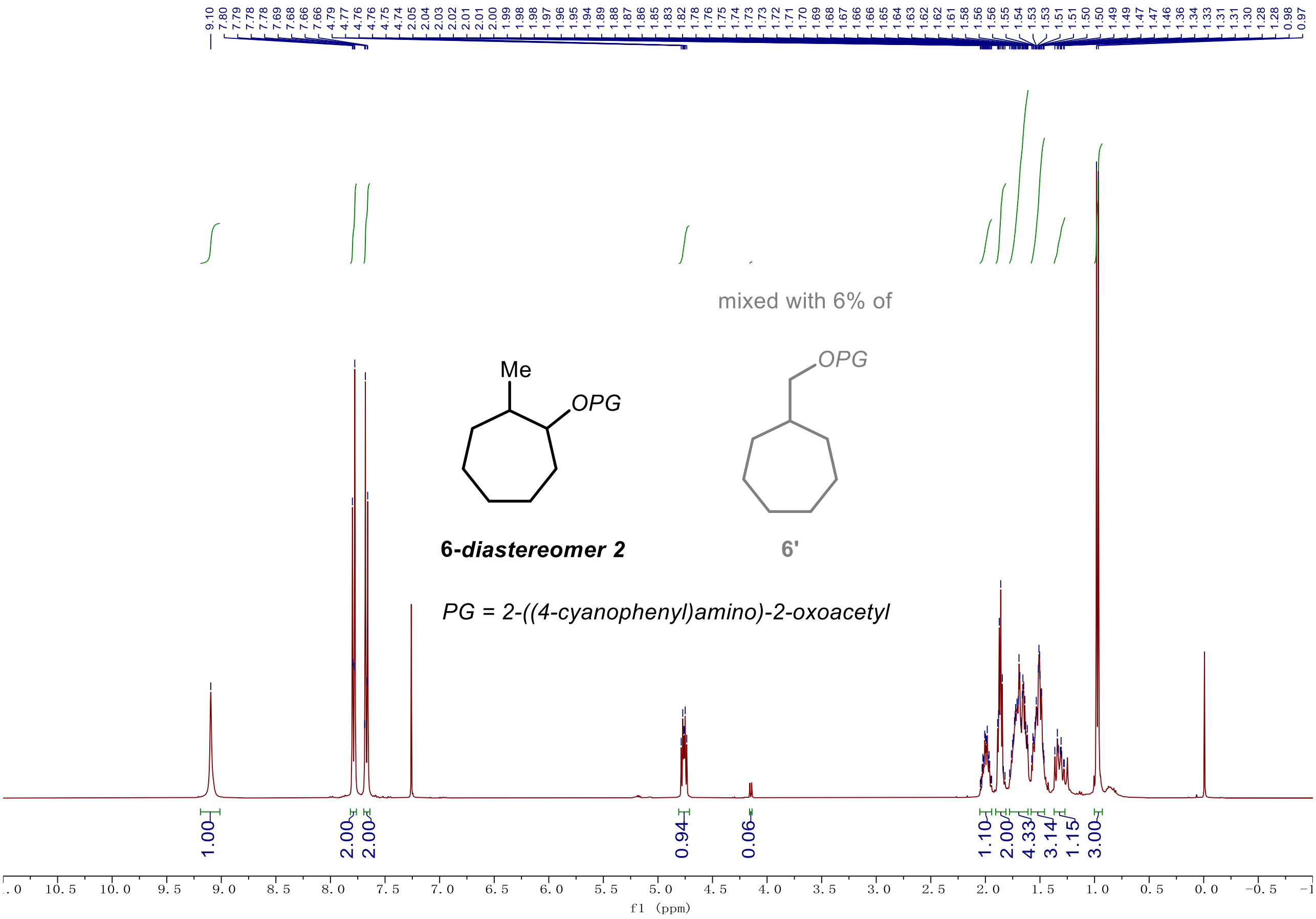

Figure S97 | ^{13}C NMR (101 MHz, $CDCl_3$) spectrum of **6-*diastereomer 2*** (mixed with 6% of **6'**).

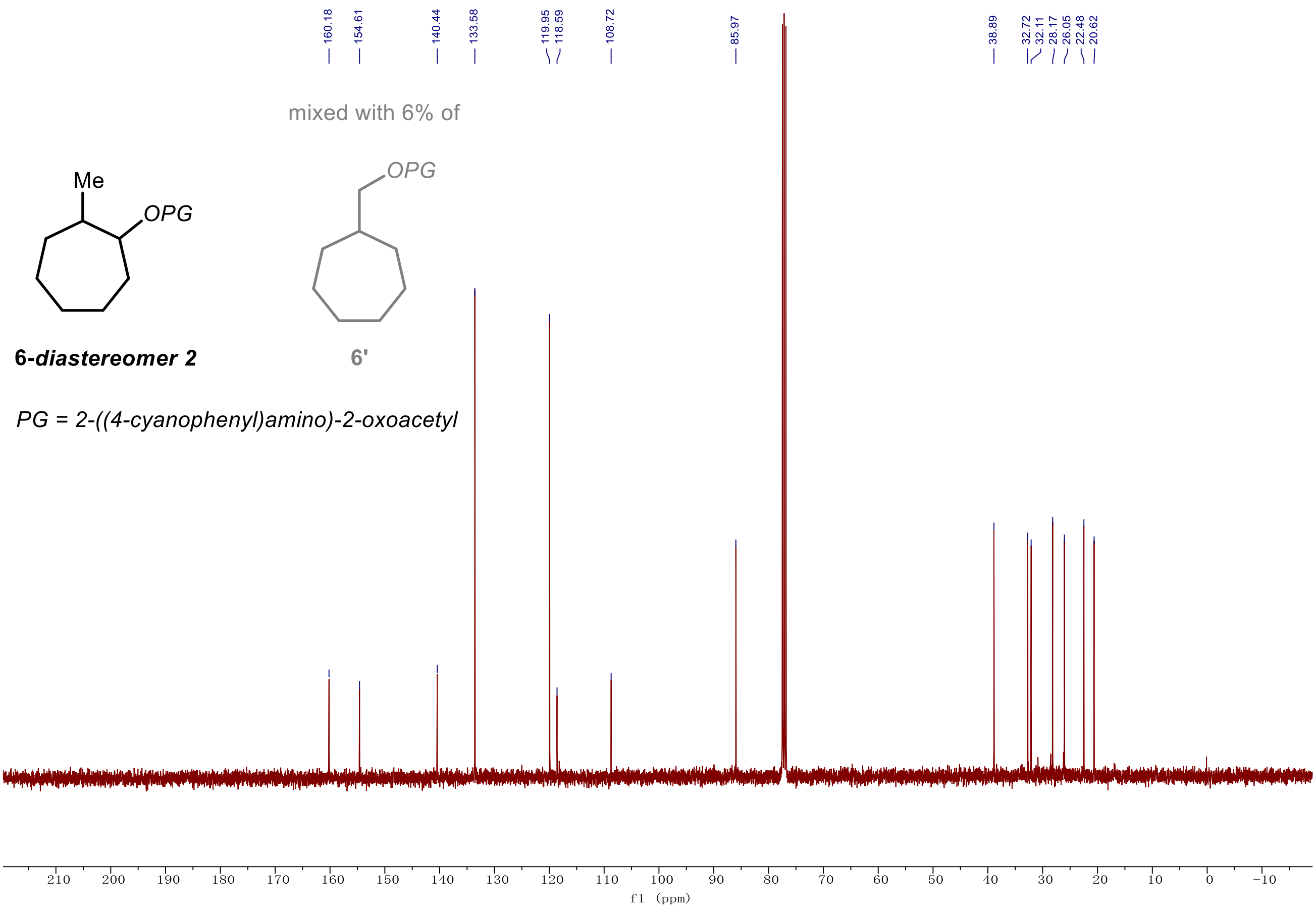

Figure S98 | ^{1}H NMR (400 MHz, $CDCl_3$) spectrum of **8.**

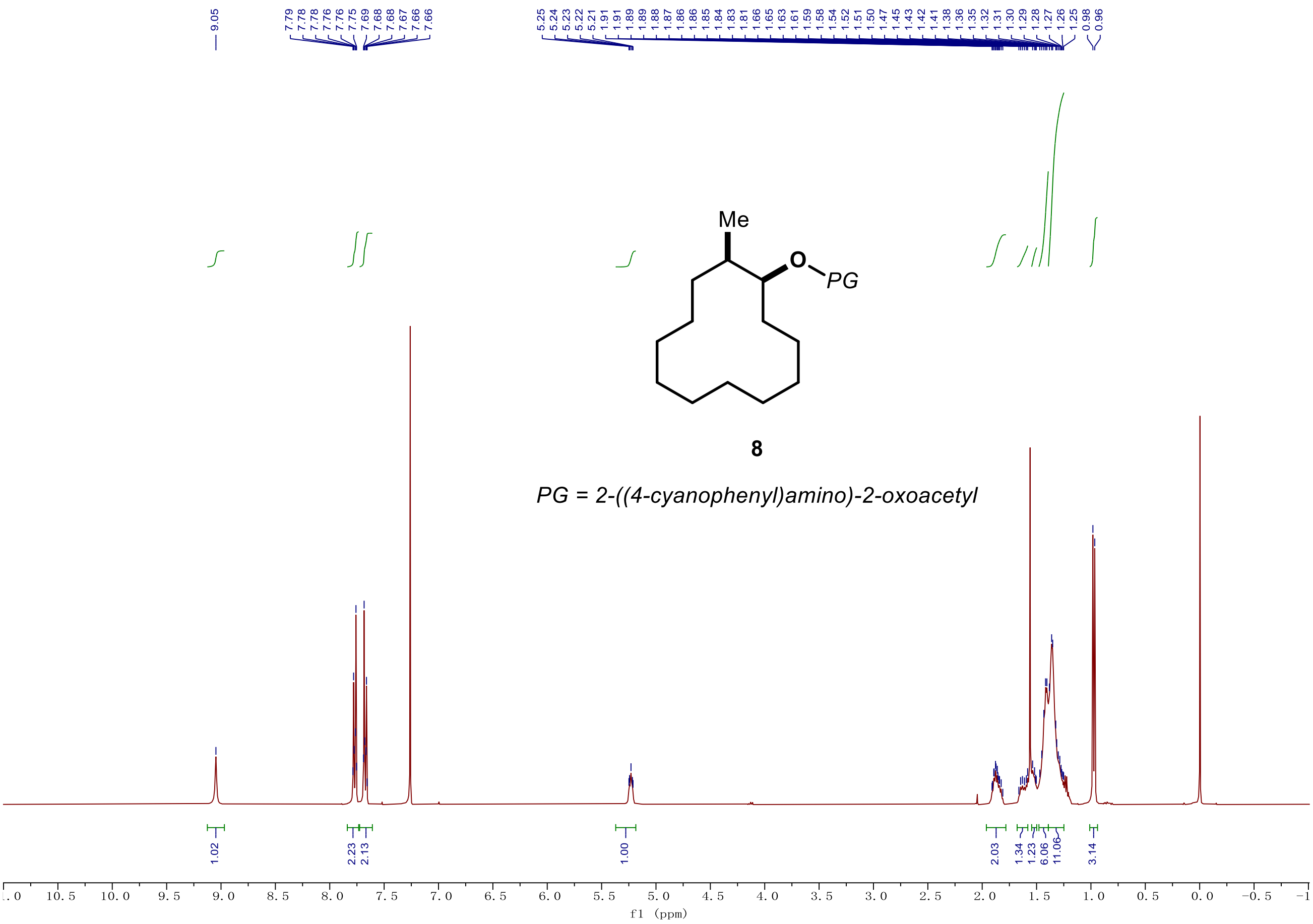

Figure S99 | ^{13}C NMR (101 MHz, $CDCl_3$) spectrum of **8**.

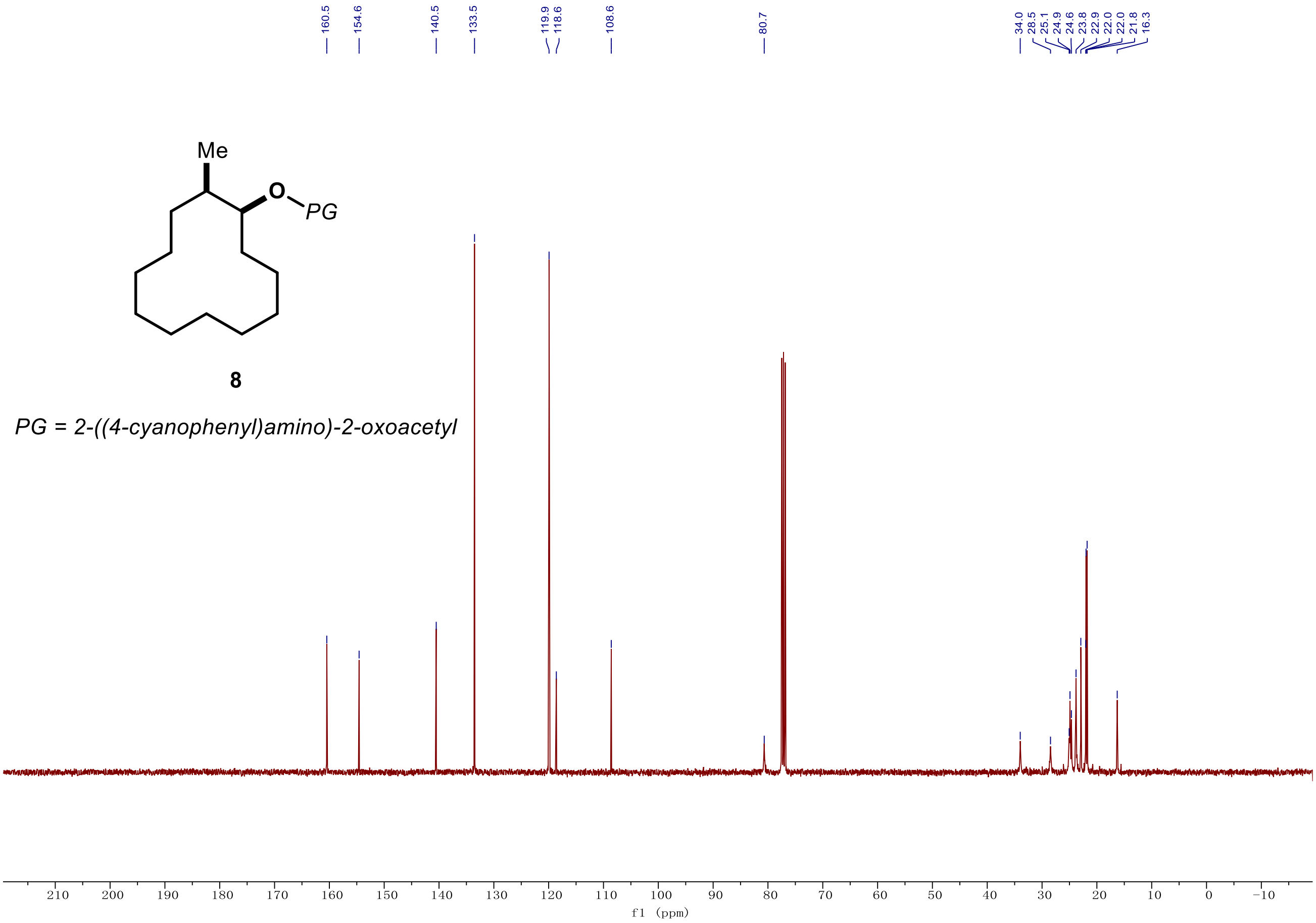

Figure S100 | ^{1}H NMR (600 MHz, $CDCl_3$) spectrum of **10a.**

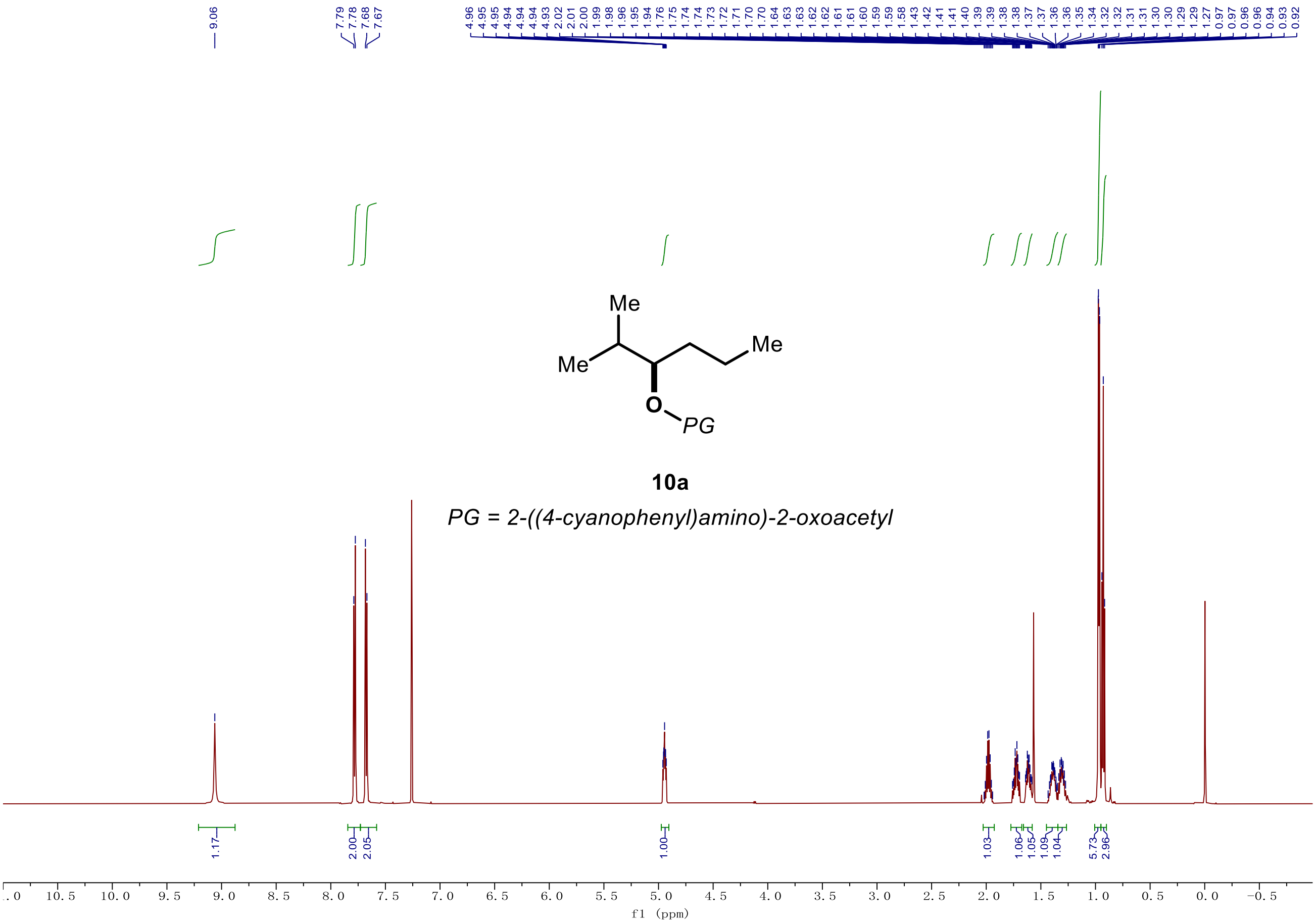

Figure S101 | ^{13}C NMR (151 MHz, CDCl$_3$) spectrum of **10a**.

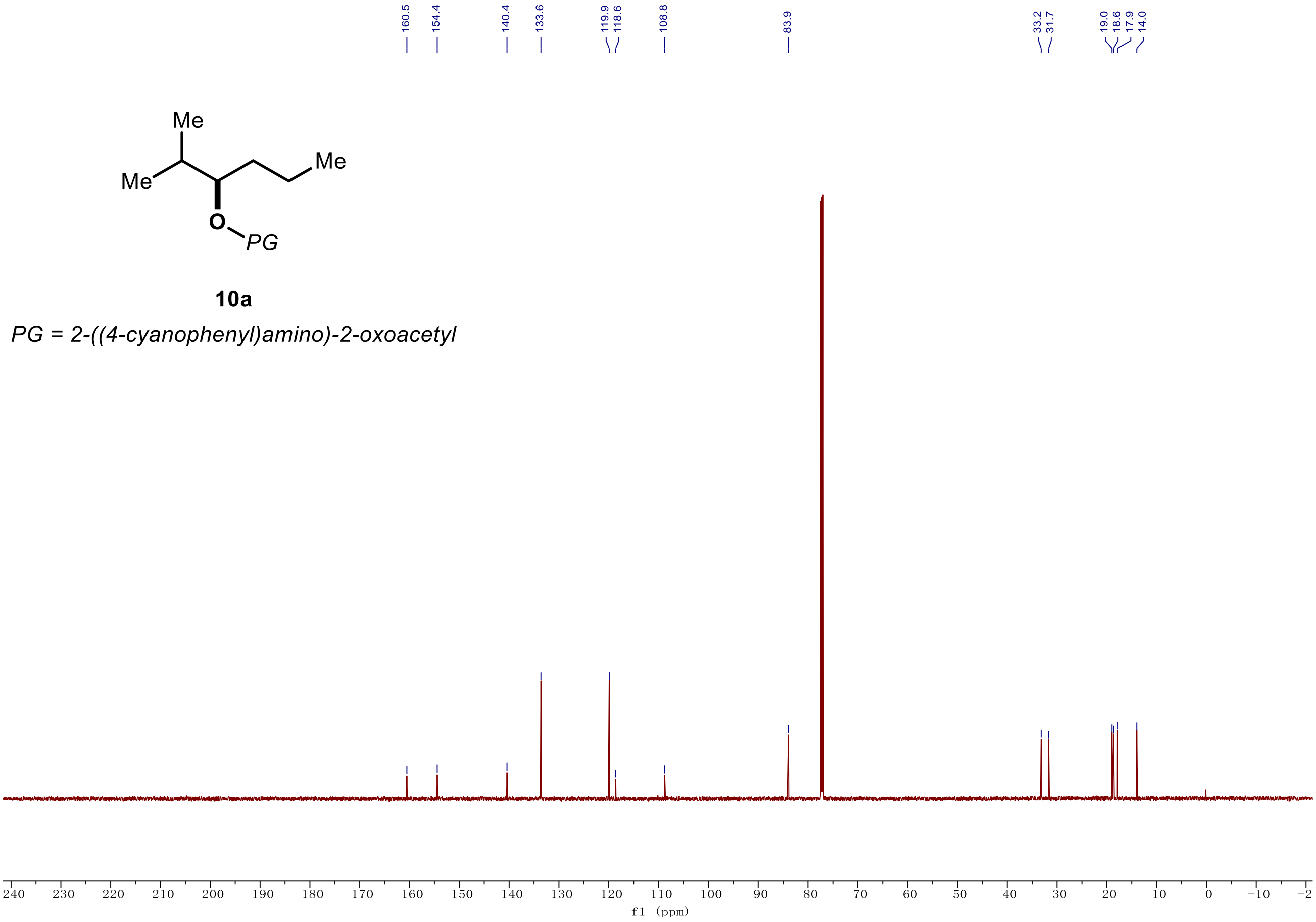

Figure S102 | ^{1}H NMR (400 MHz, CDCl$_3$) spectrum of **10b.**

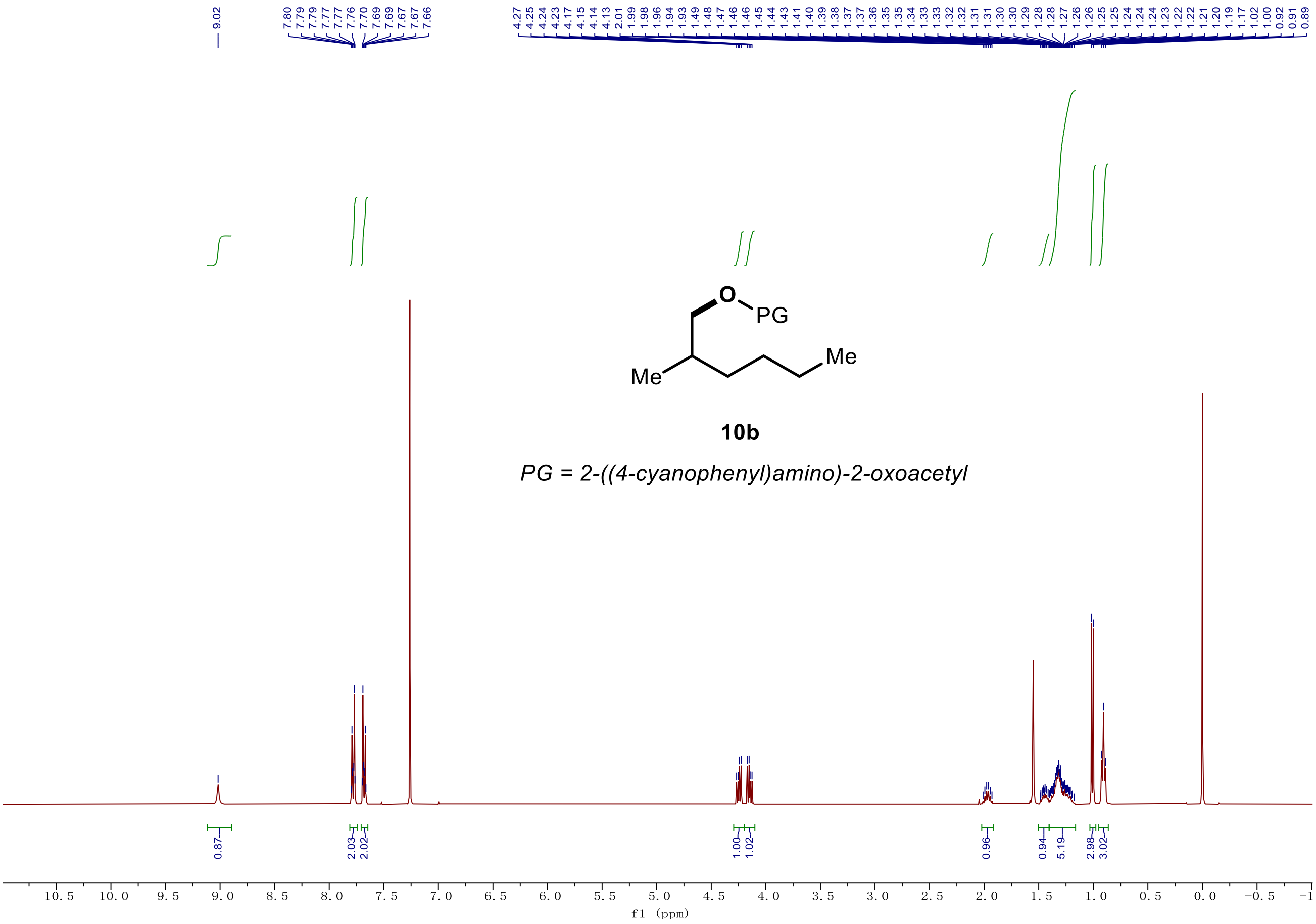

Figure S103 | ^{13}C NMR (101 MHz, $CDCl_3$) spectrum of **10b**.

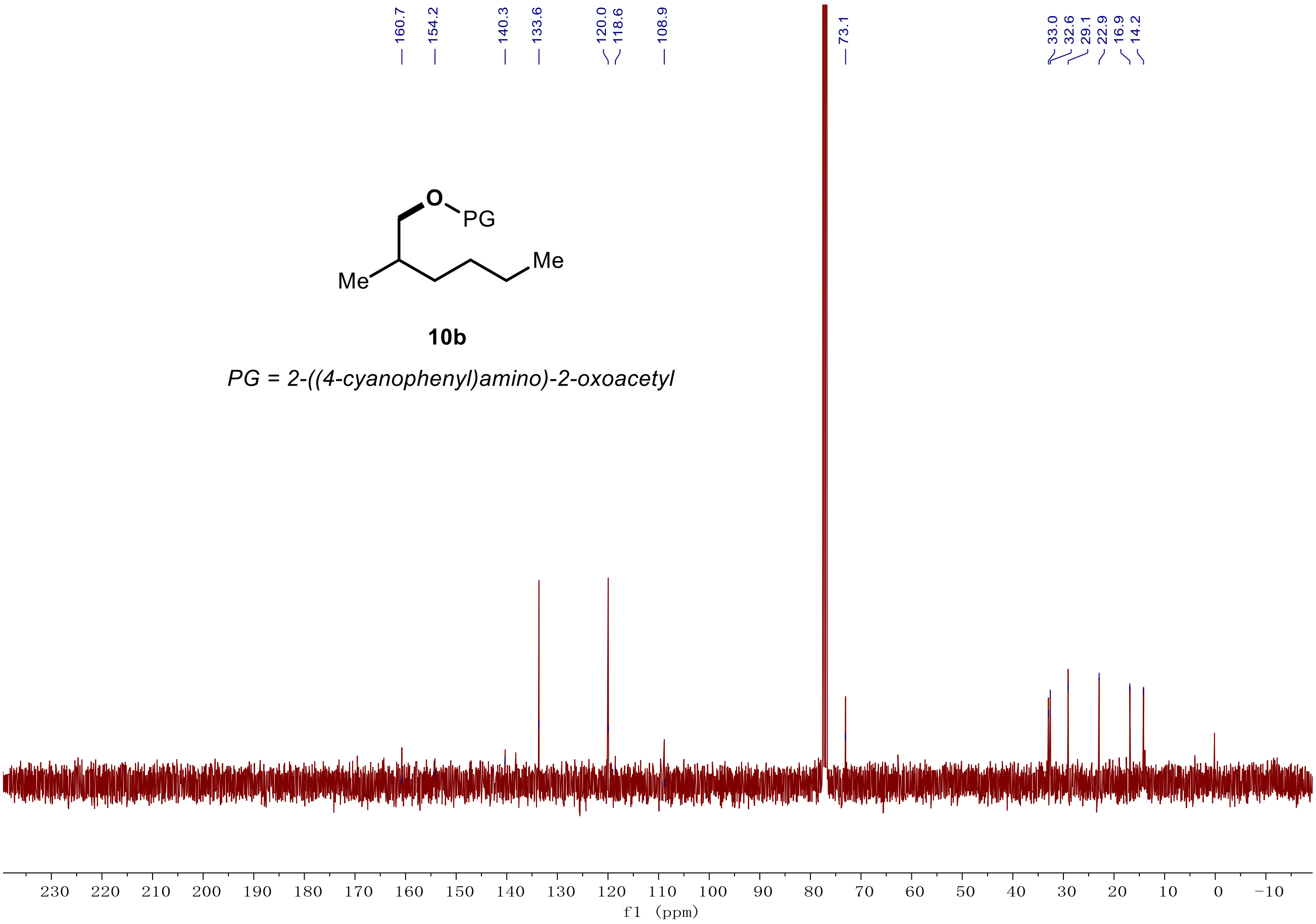

Figure S104 | ^{1}H NMR (400 MHz, $CDCl_3$) spectrum of **12a.**

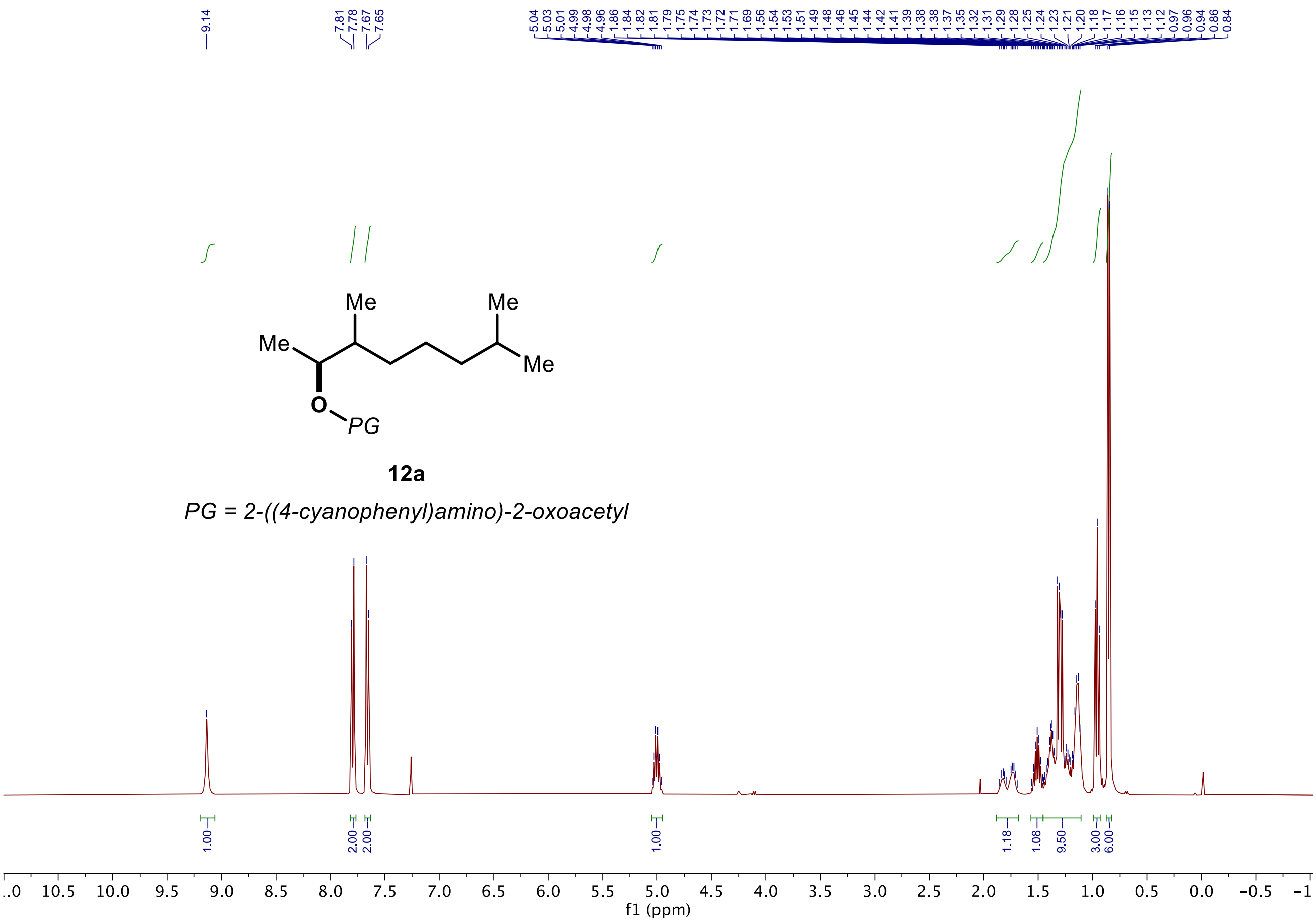

Figure S105 | ^{13}C NMR (101 MHz, $CDCl_3$) spectrum of **12a**.

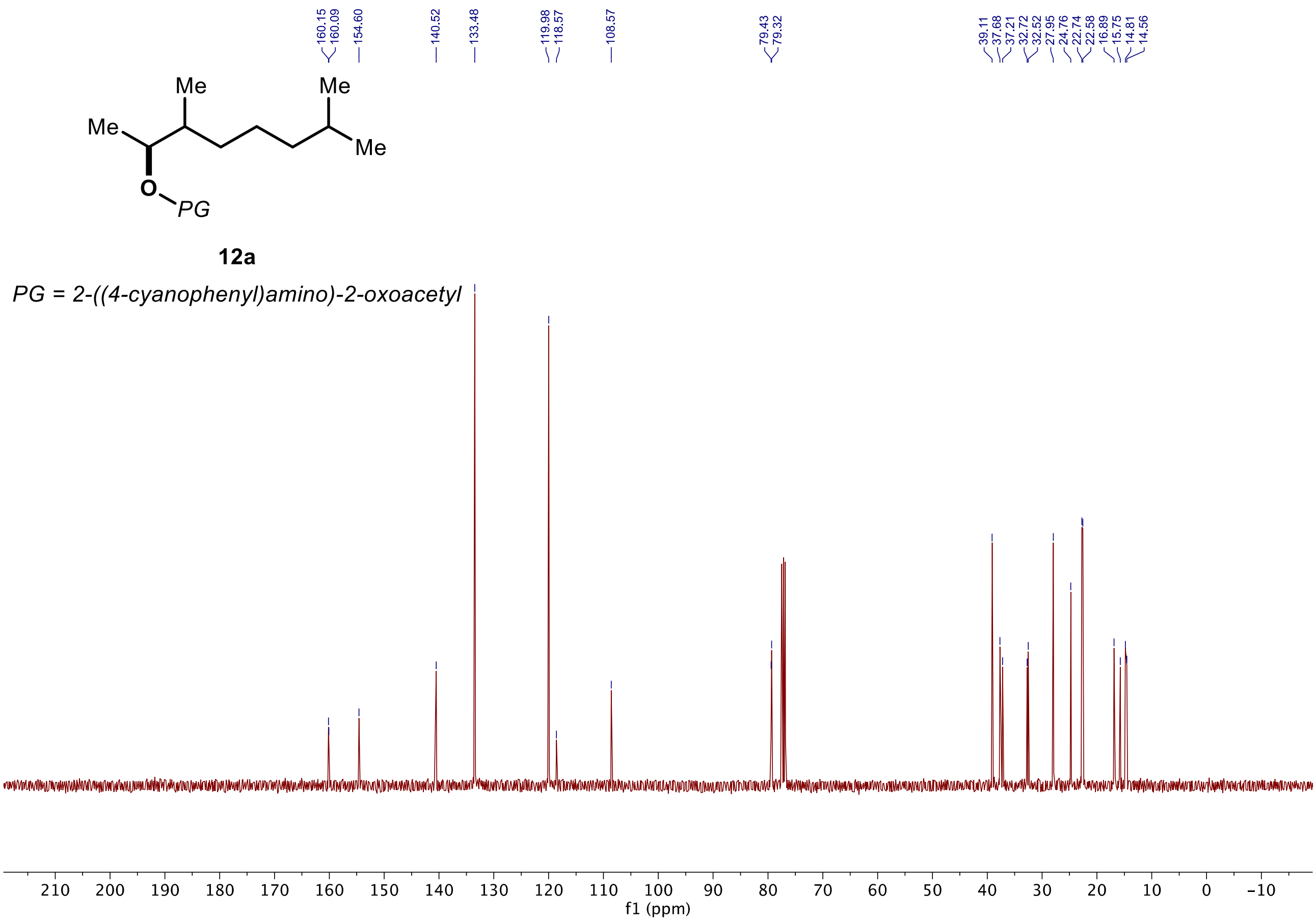

Figure S106 | ^{1}H NMR (400 MHz, $CDCl_3$) spectrum of **12b**.

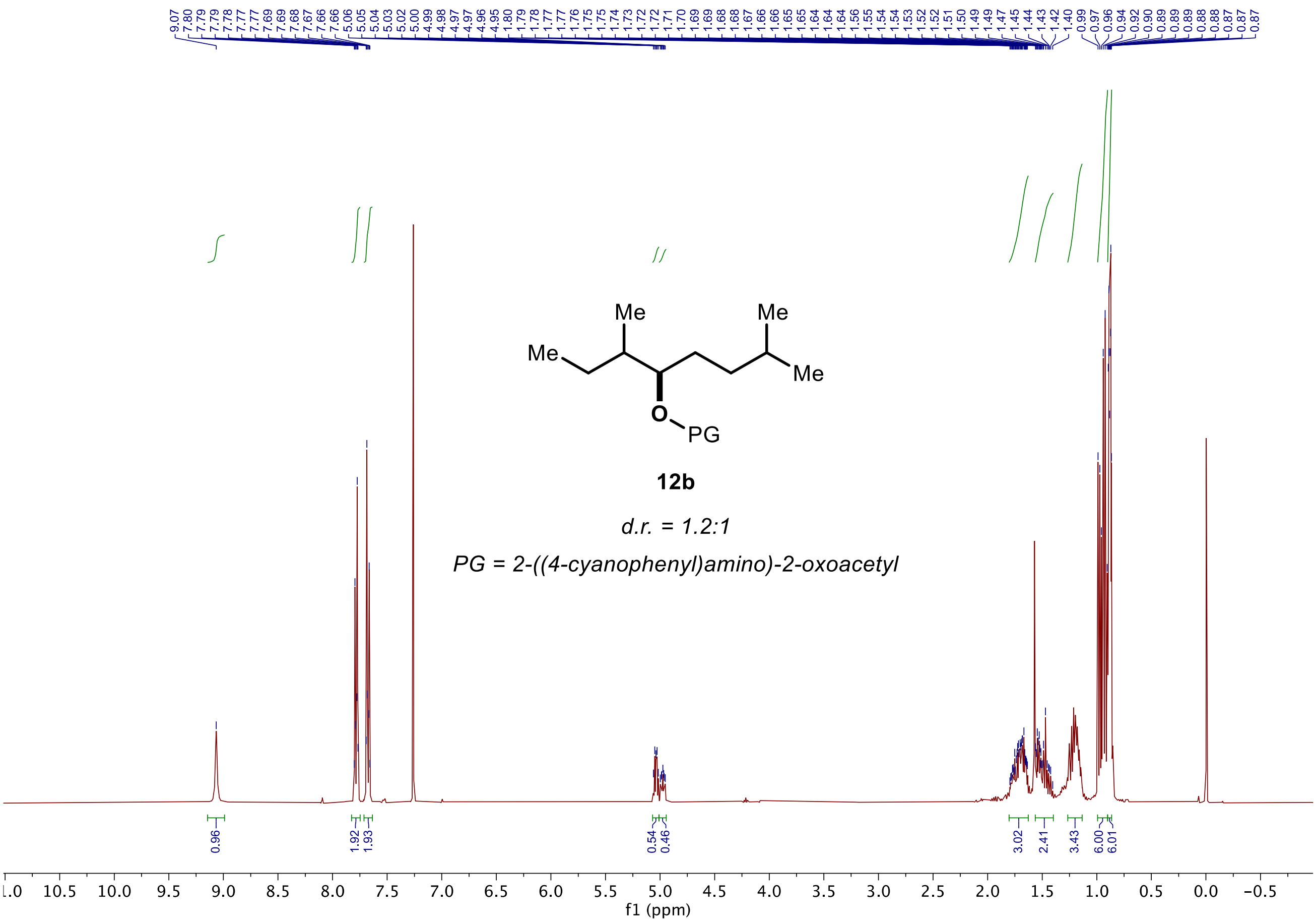

Figure S107 | ^{13}C NMR (101 MHz, CDCl$_3$) spectrum of **12b**.

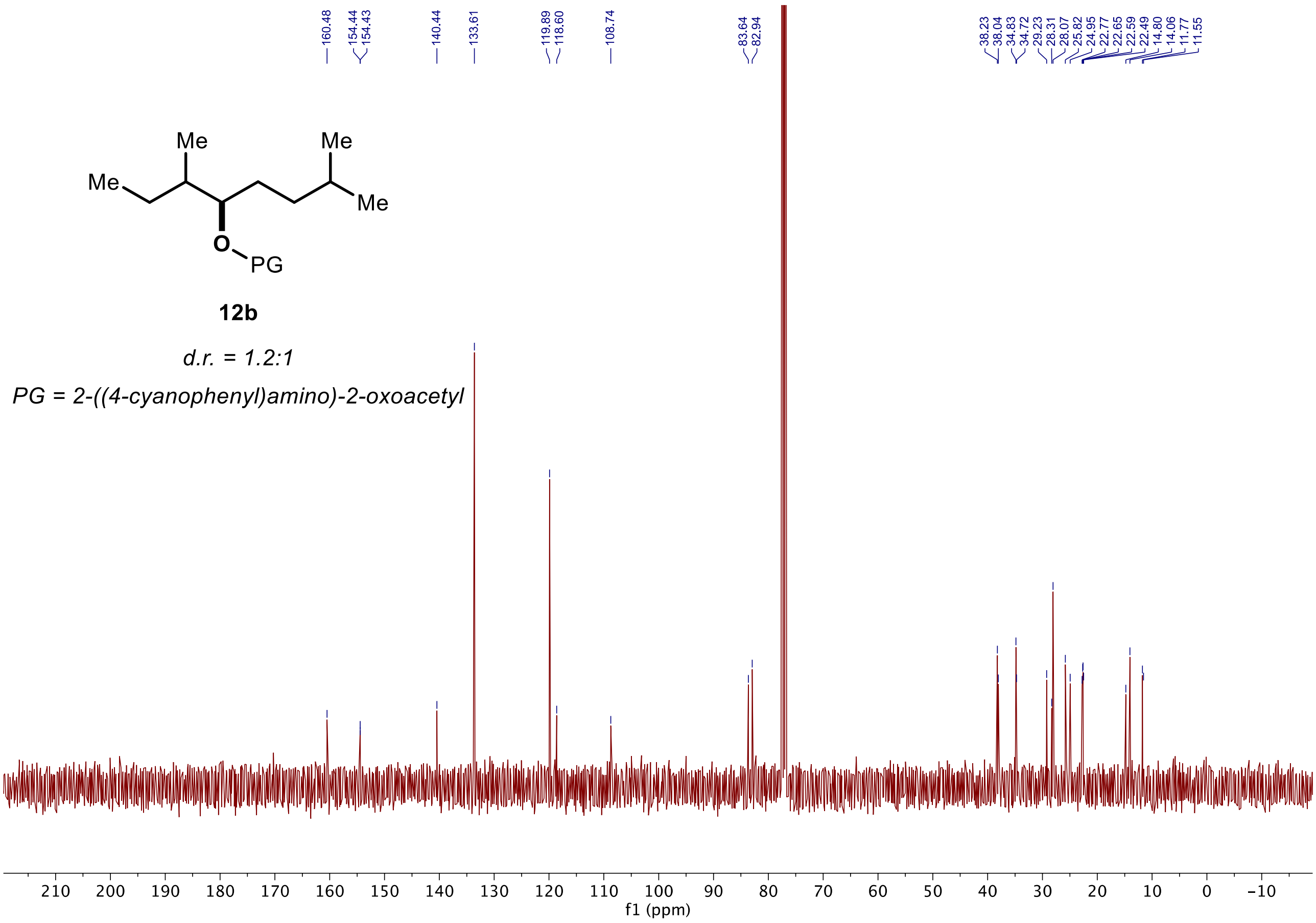

Figure S108 | ^{1}H NMR (400 MHz, CDCl$_3$) spectrum of **14a.**

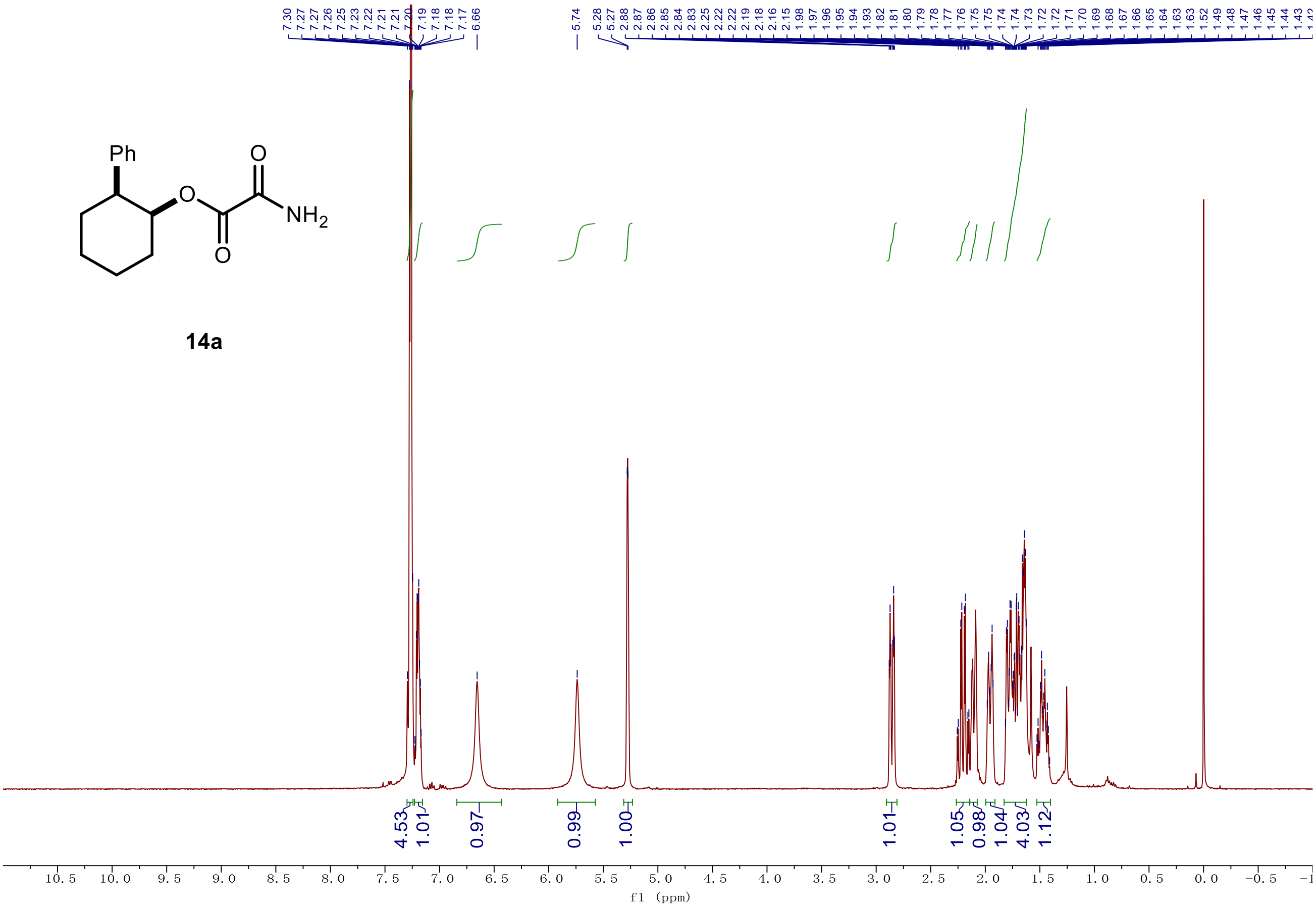

Figure S109 | ^{13}C NMR (101 MHz, CDCl$_3$) spectrum of **14a**.

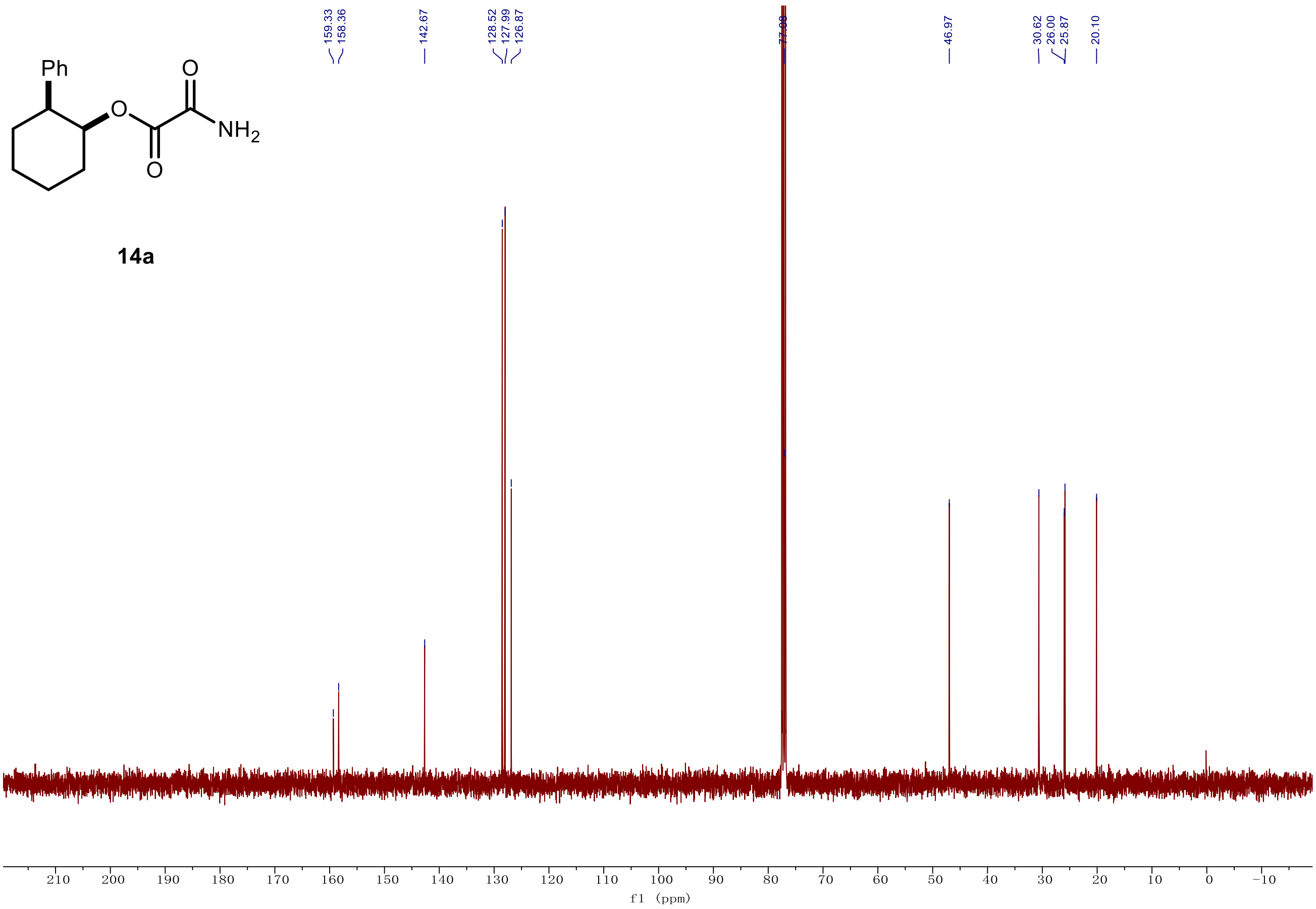

Figure S110 | ^{1}H NMR (400 MHz, CDCl$_3$) spectrum of **14b.**

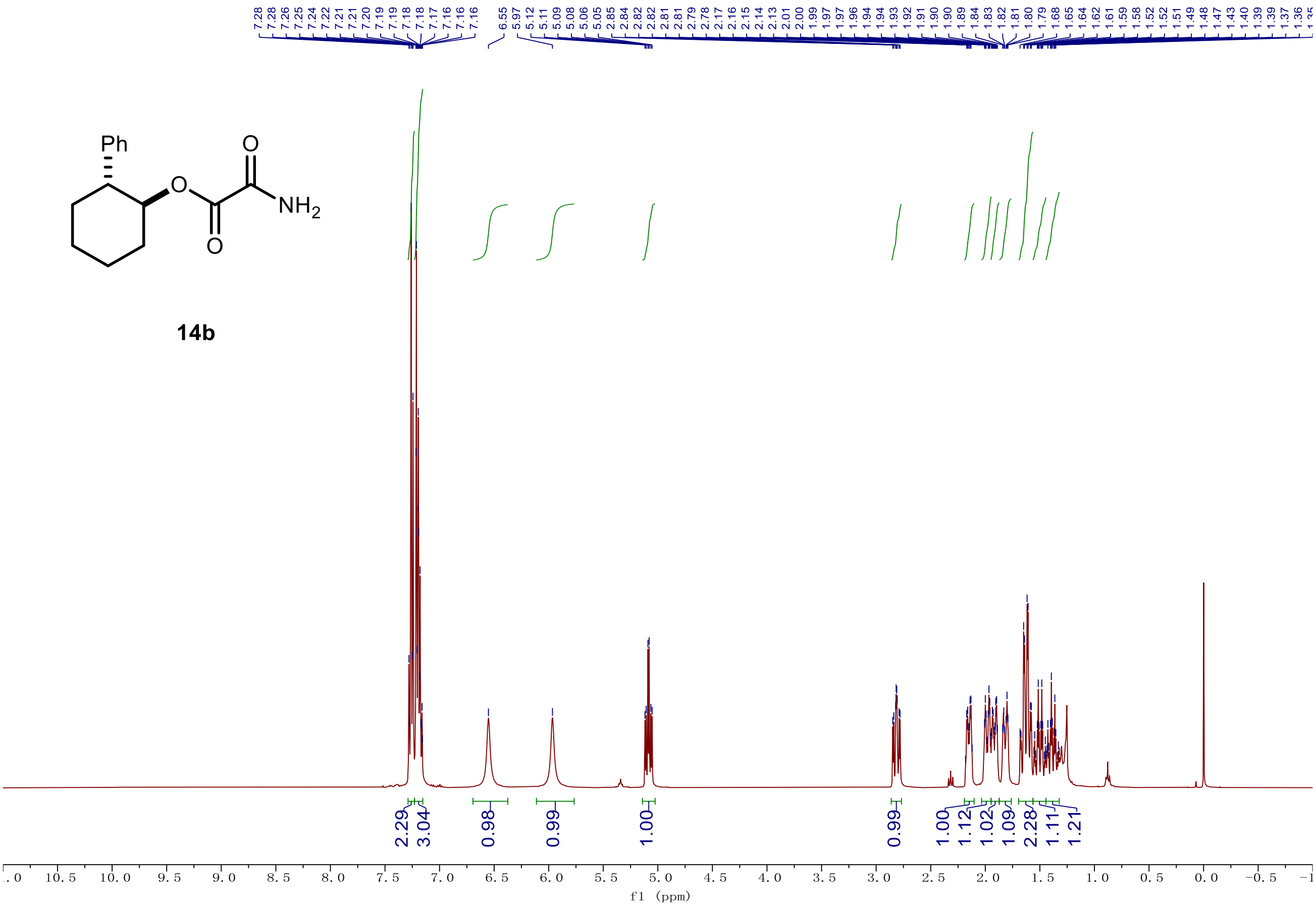

Figure S111 | ^{13}C NMR (101 MHz, $CDCl_3$) spectrum of **14b**.

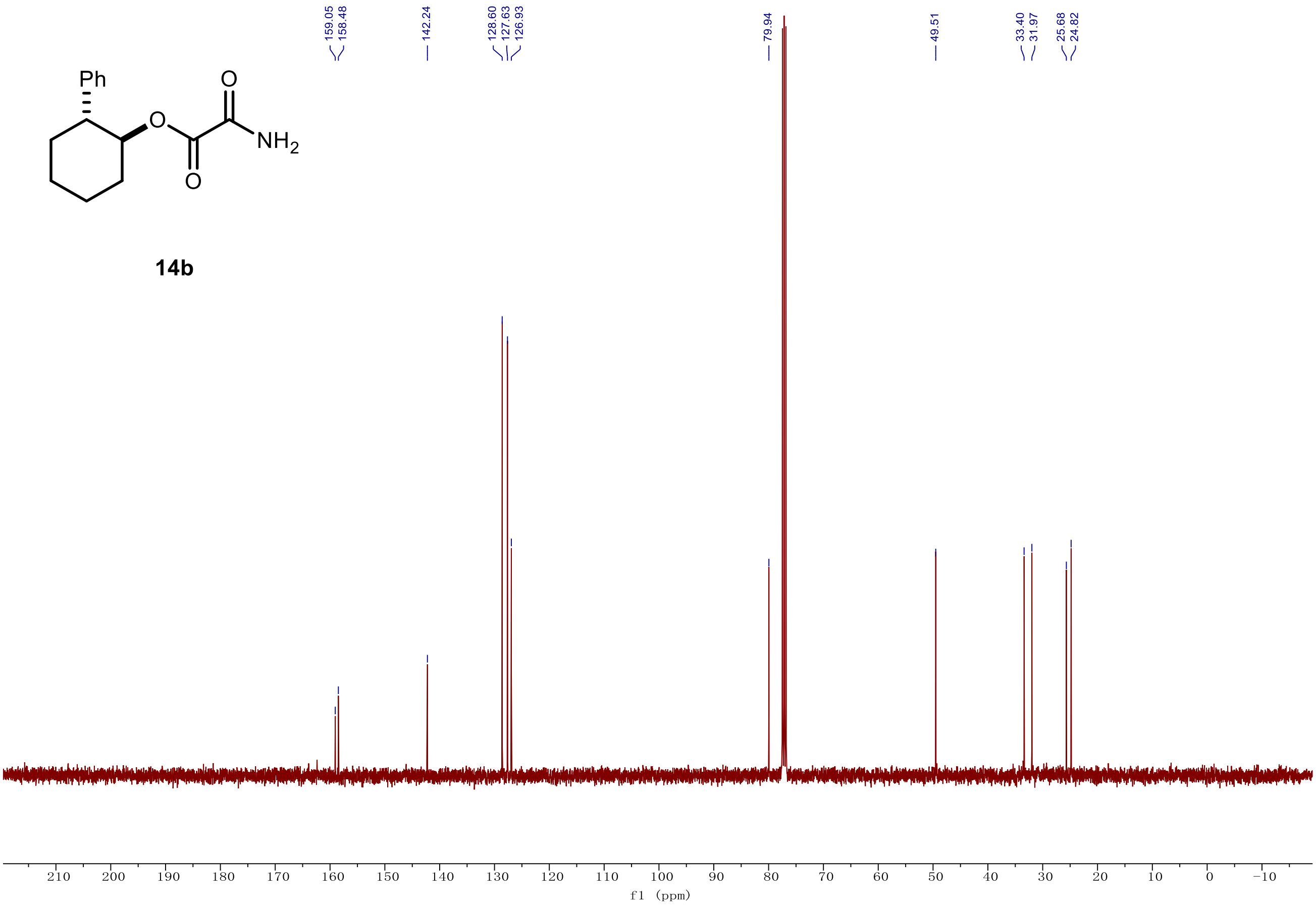

Figure S112 | ^{1}H NMR (400 MHz, $CDCl_3$) spectrum of **16.**

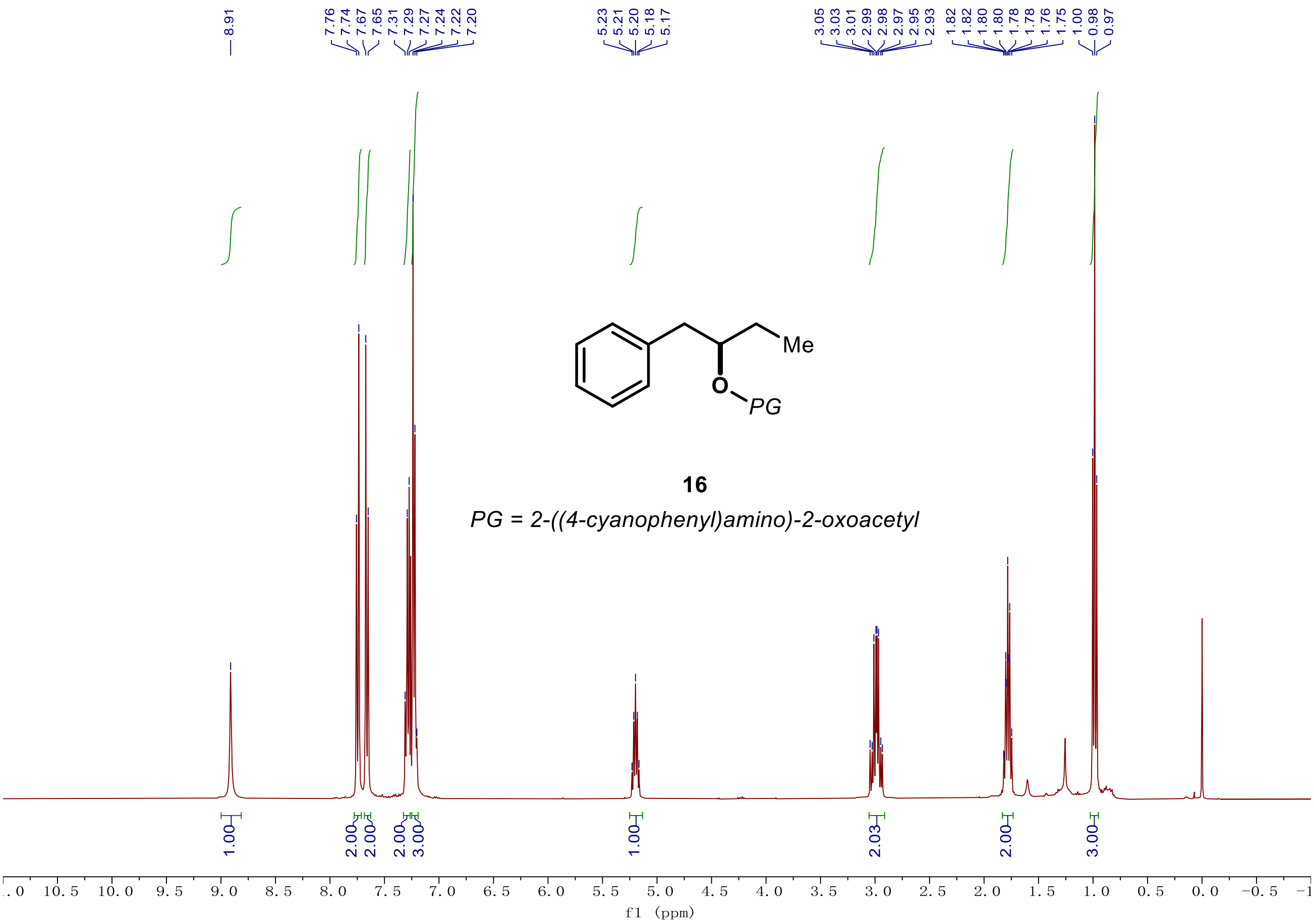

Figure S113 | ^{13}C NMR (101 MHz, $CDCl_3$) spectrum of **16**.

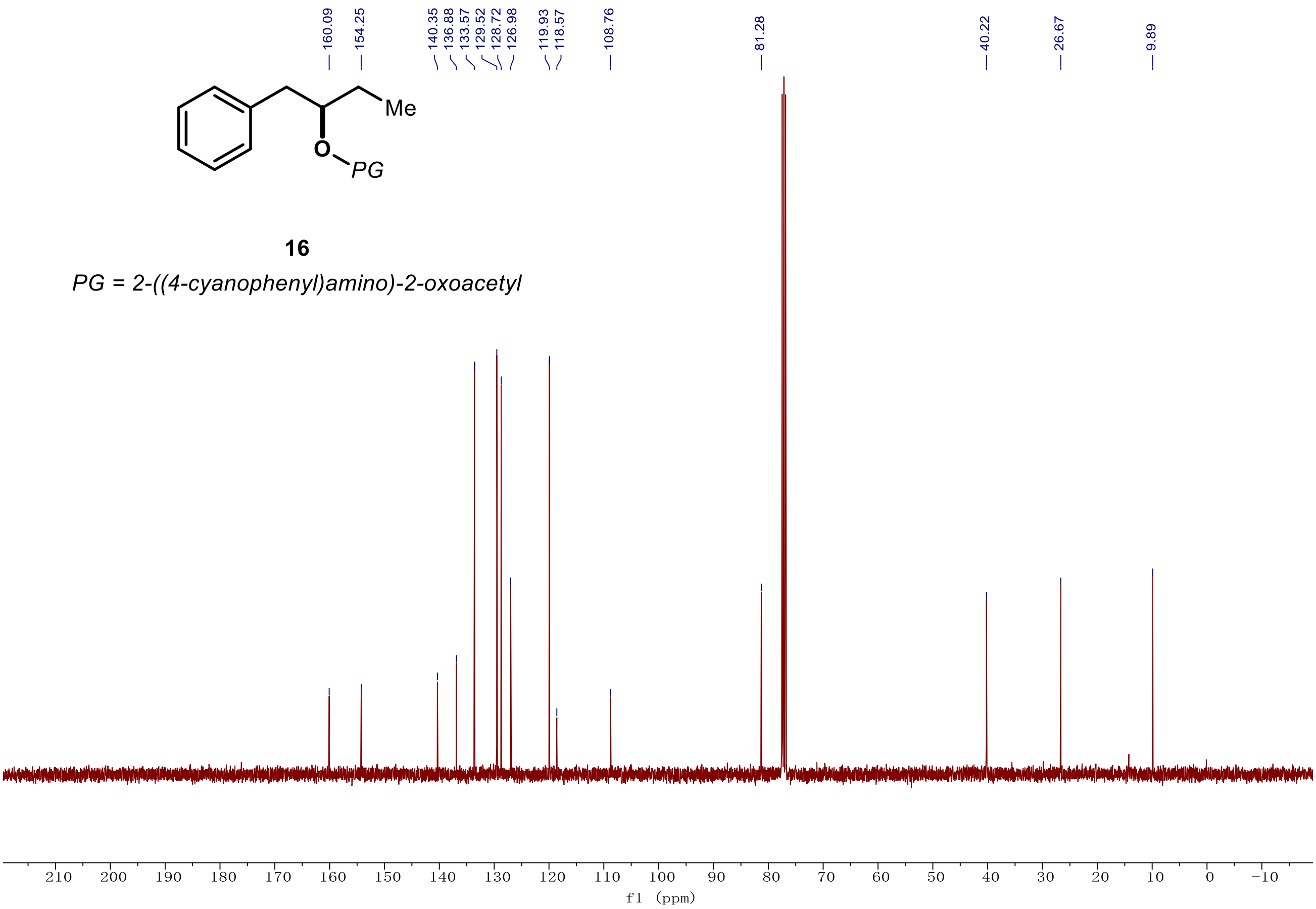

Figure S114 | ^{1}H NMR (400 MHz, $CDCl_3$) spectrum of **18a**.

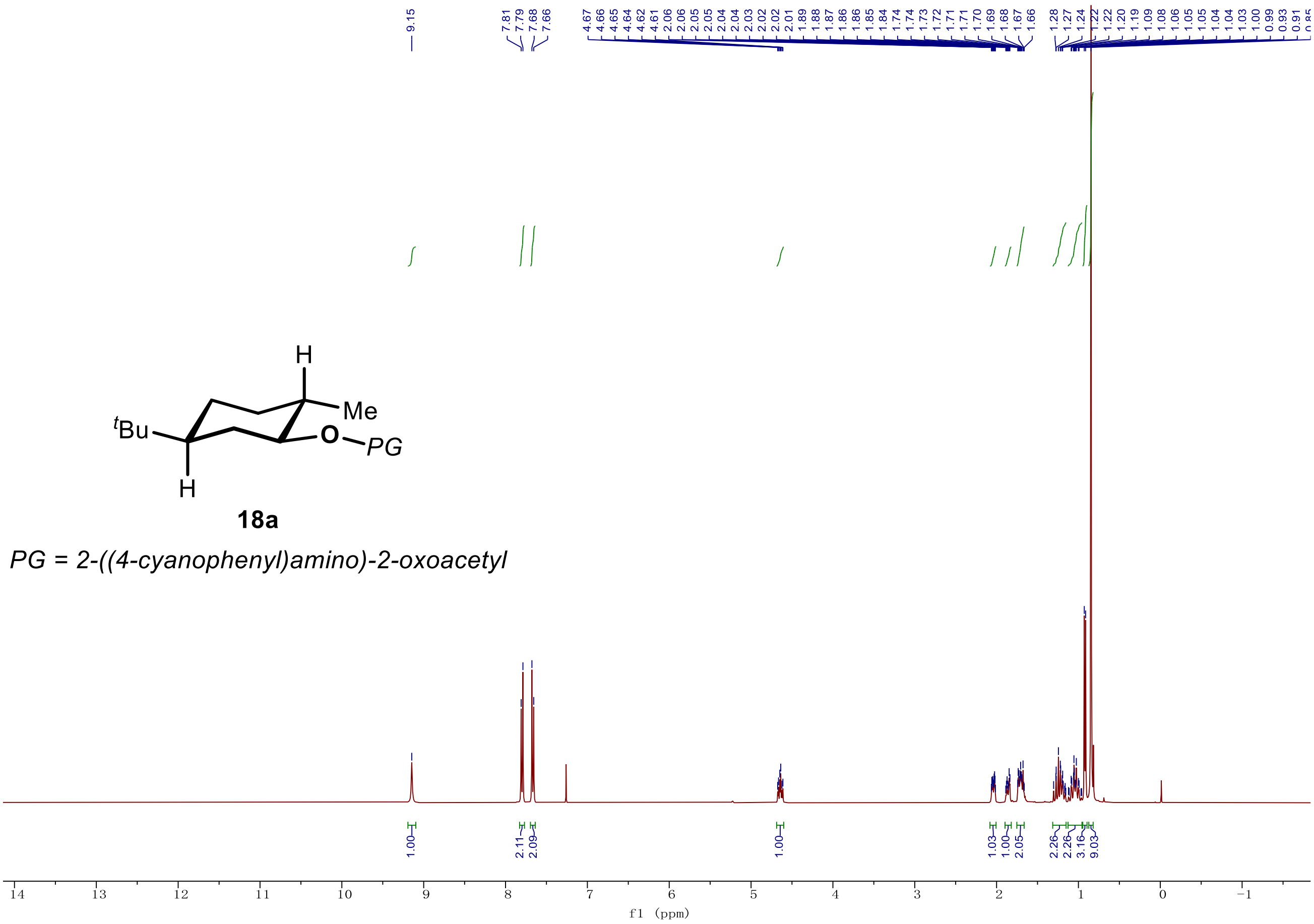

Figure S115 | ^{13}C NMR (101 MHz, $CDCl_3$) spectrum of **18a**.

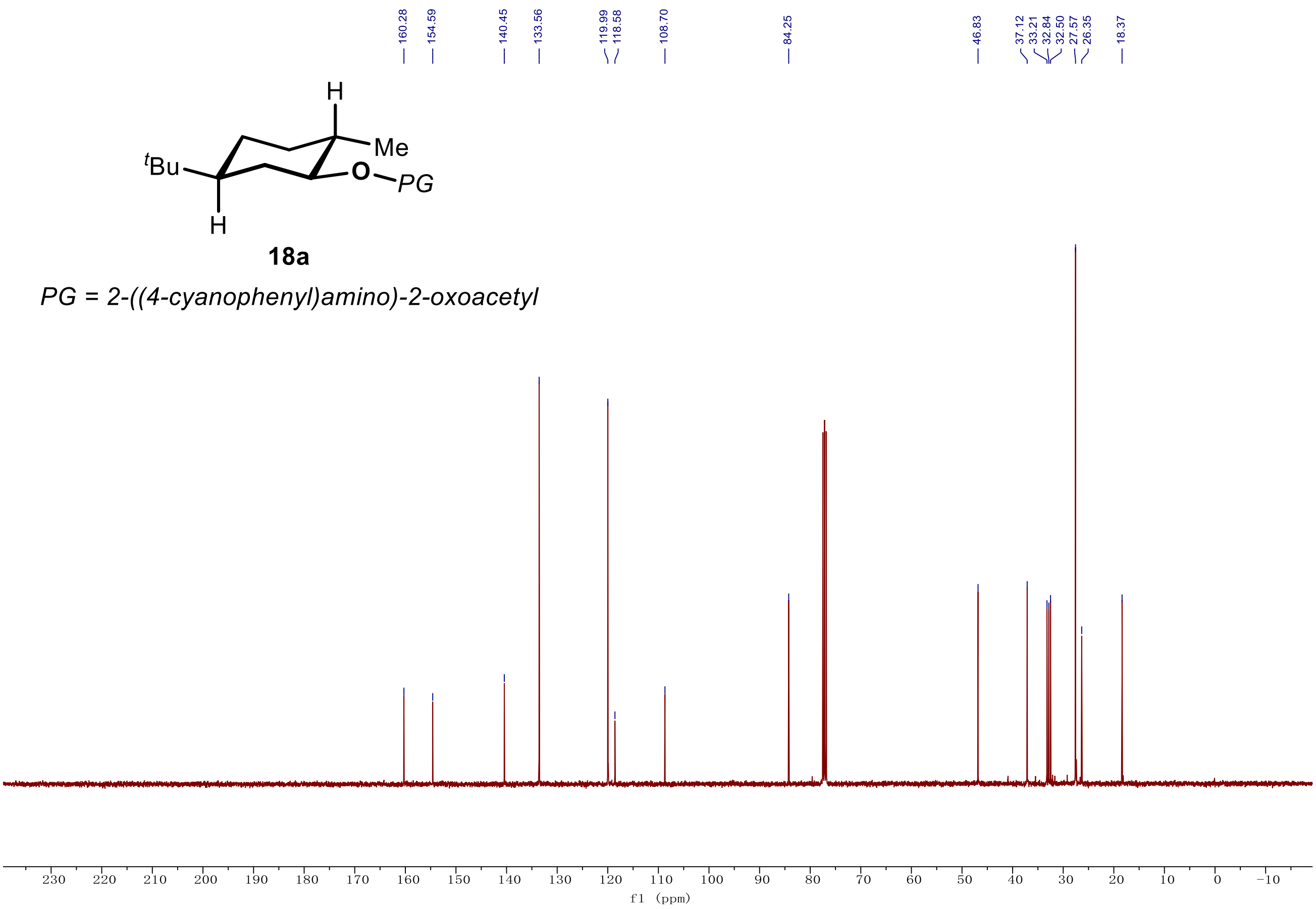

Figure S116 | ^{1}H NMR (600 MHz, $CDCl_3$) spectrum of **20**.

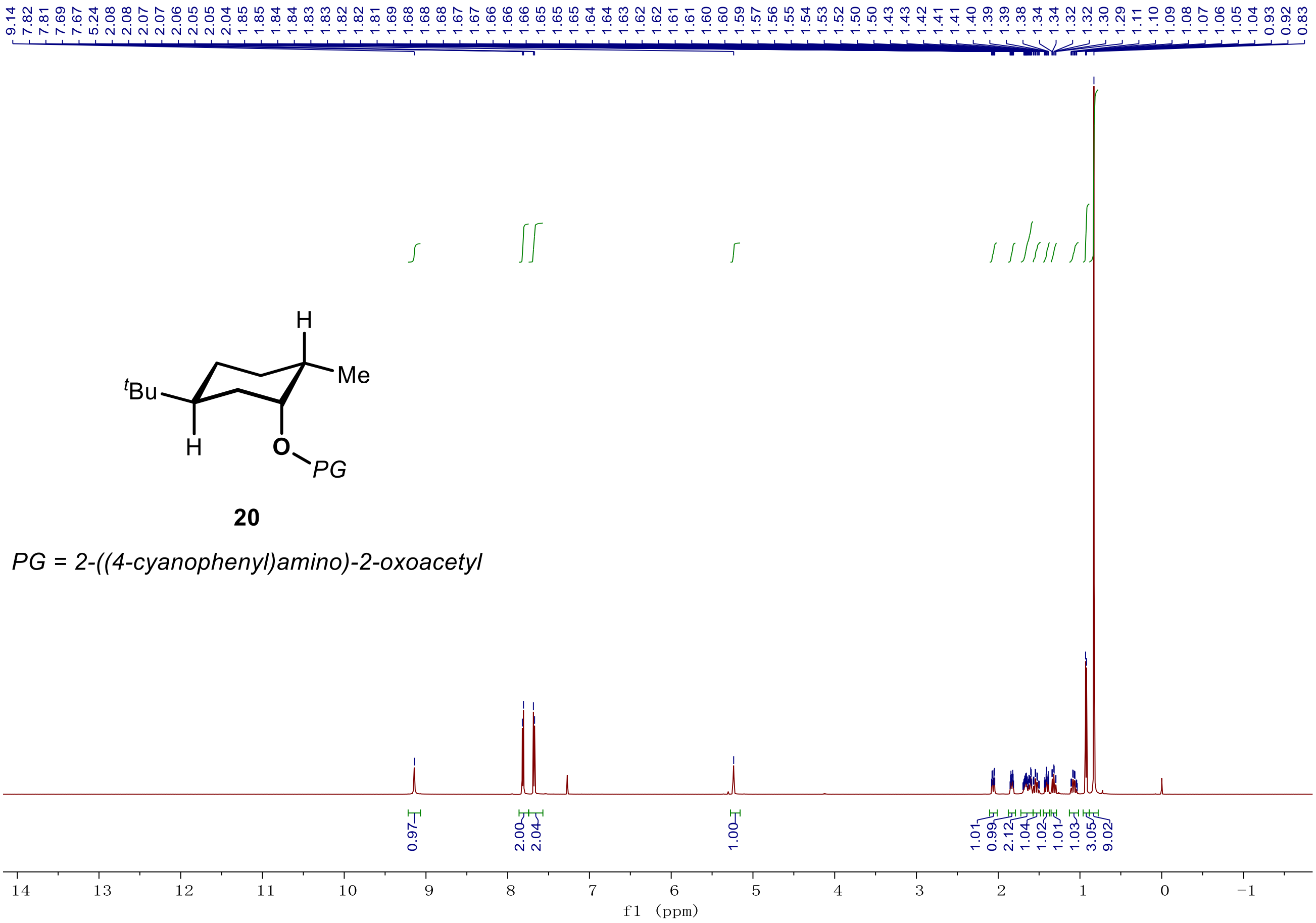

Figure S117 | ^{13}C NMR (151 MHz, $CDCl_3$) spectrum of **20**.

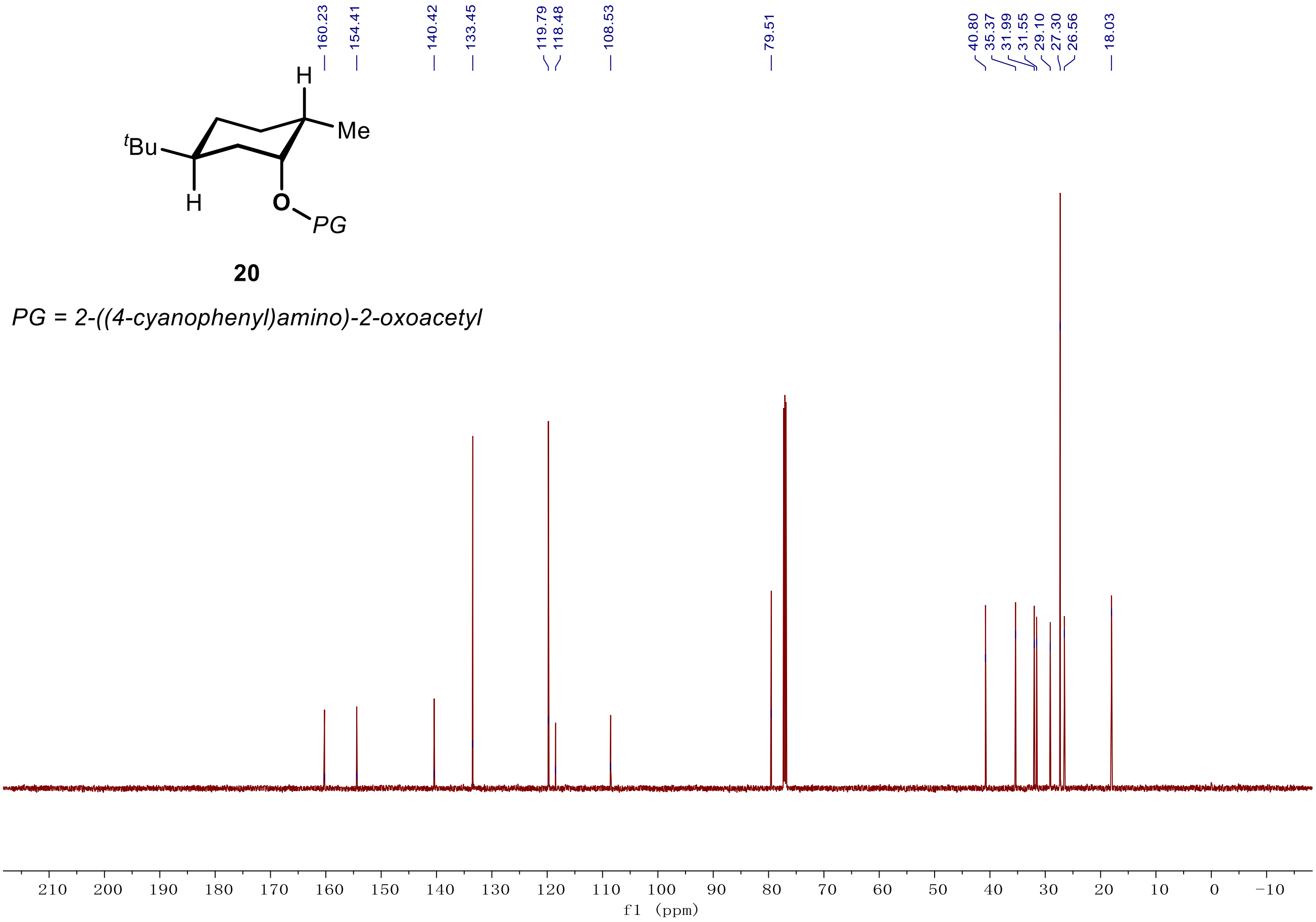

Figure S118 | ^{1}H NMR (500 MHz, $CDCl_3$) spectrum of **22**.

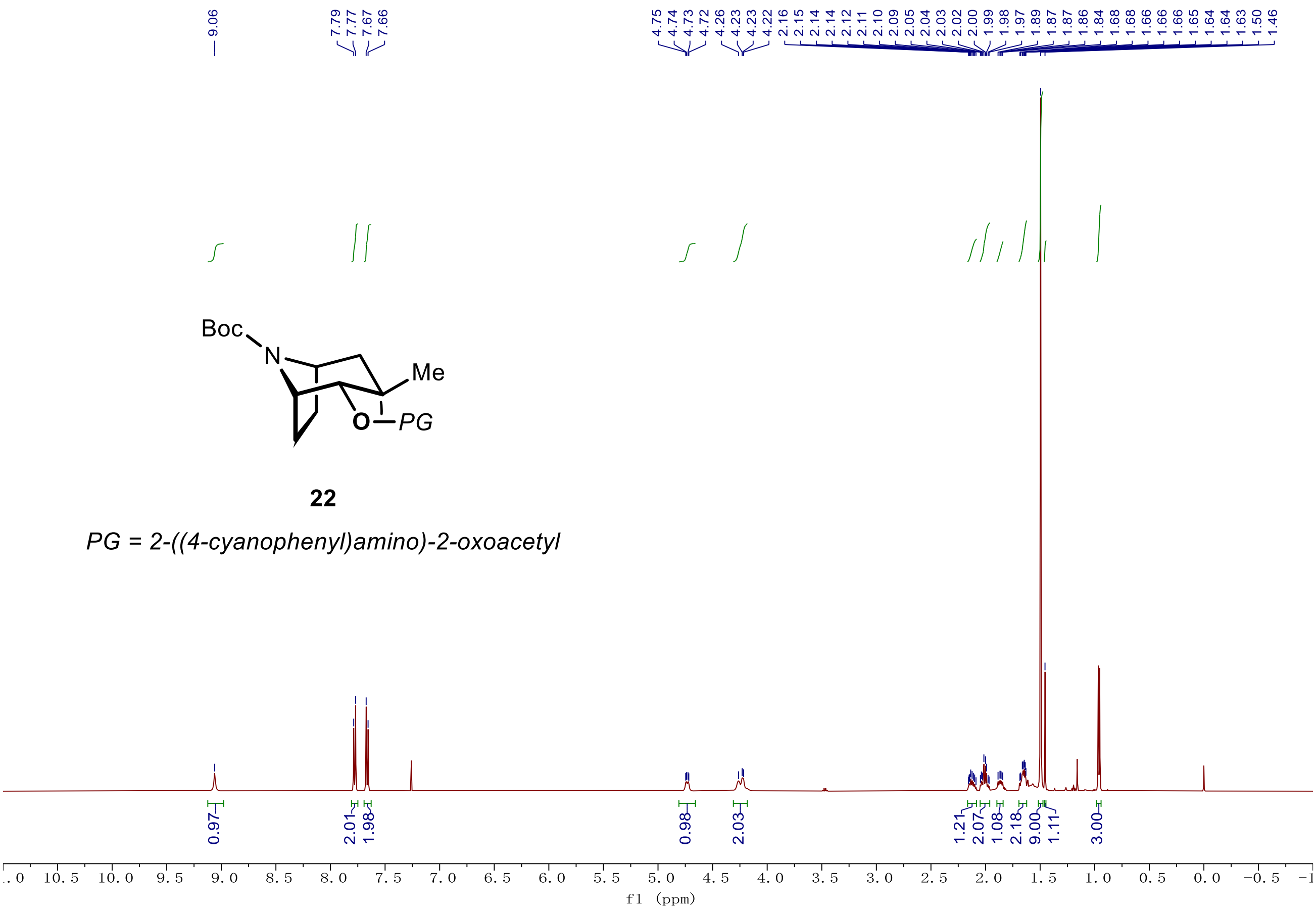

Figure S119 | ^{13}C NMR (126 MHz, $CDCl_3$) spectrum of **22**.

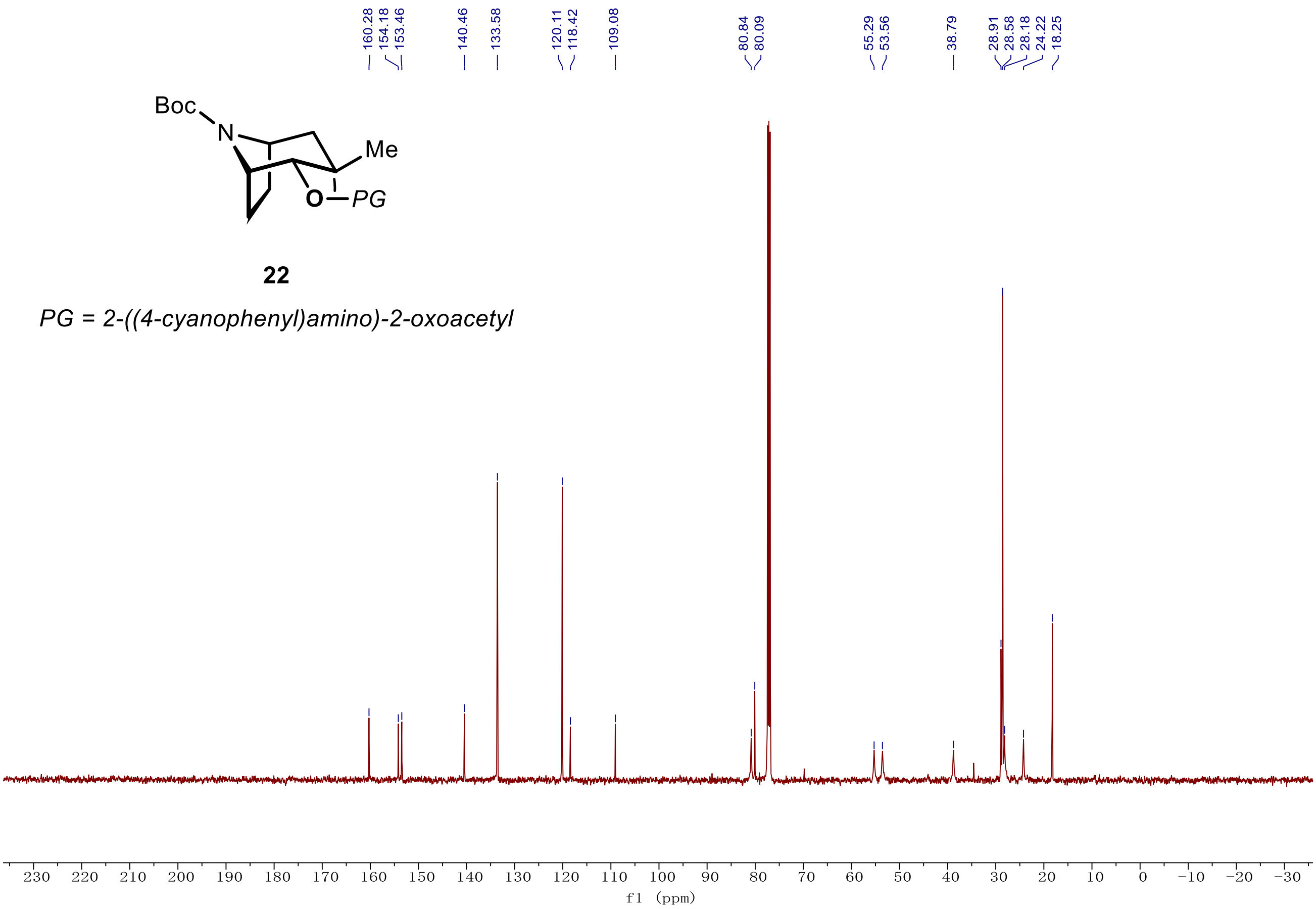

Figure S120 | ^{1}H NMR (500 MHz, $CDCl_3$) spectrum of **24a**.

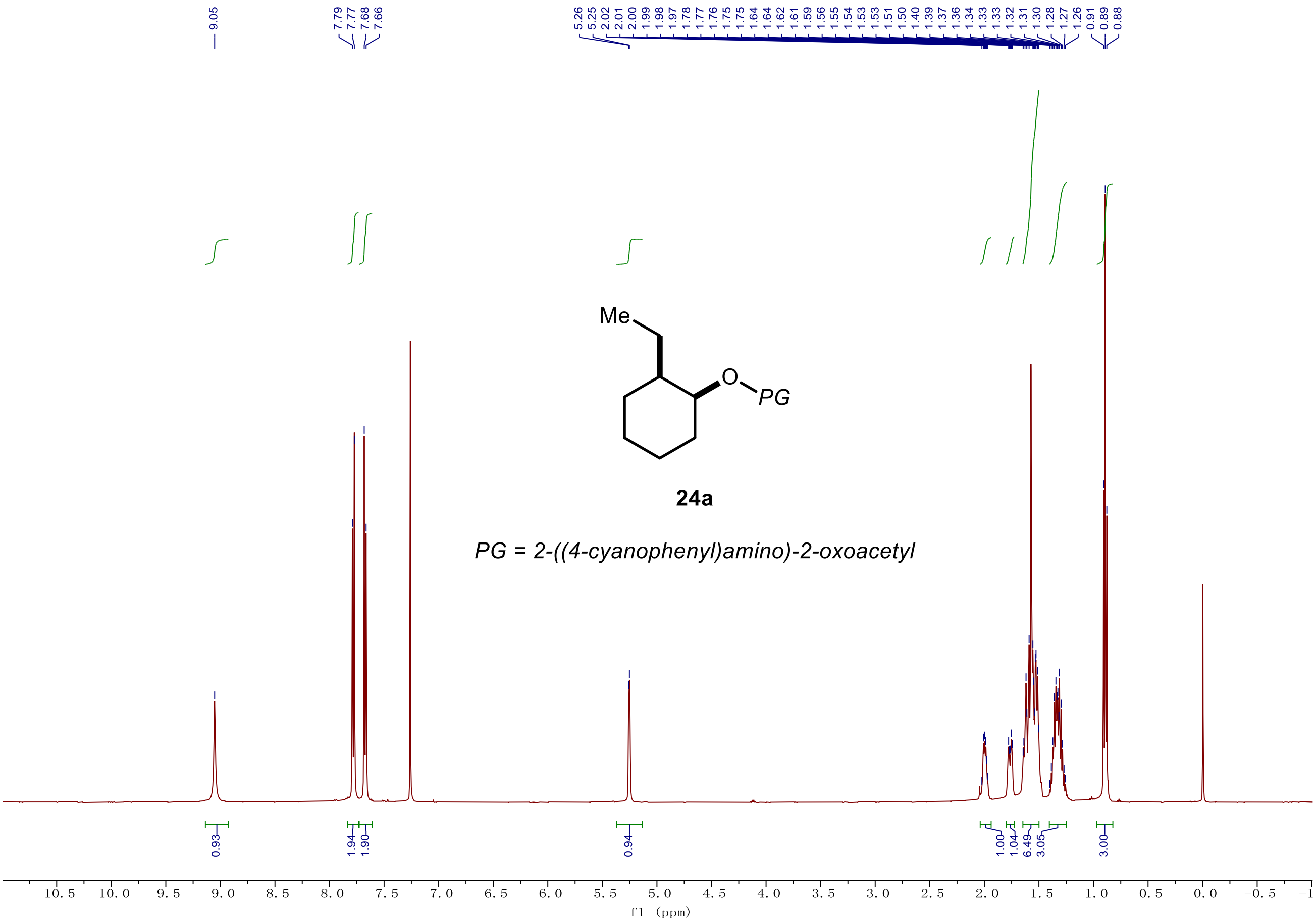

Figure S121 | ^{13}C NMR (101 MHz, $CDCl_3$) spectrum of **24a**.

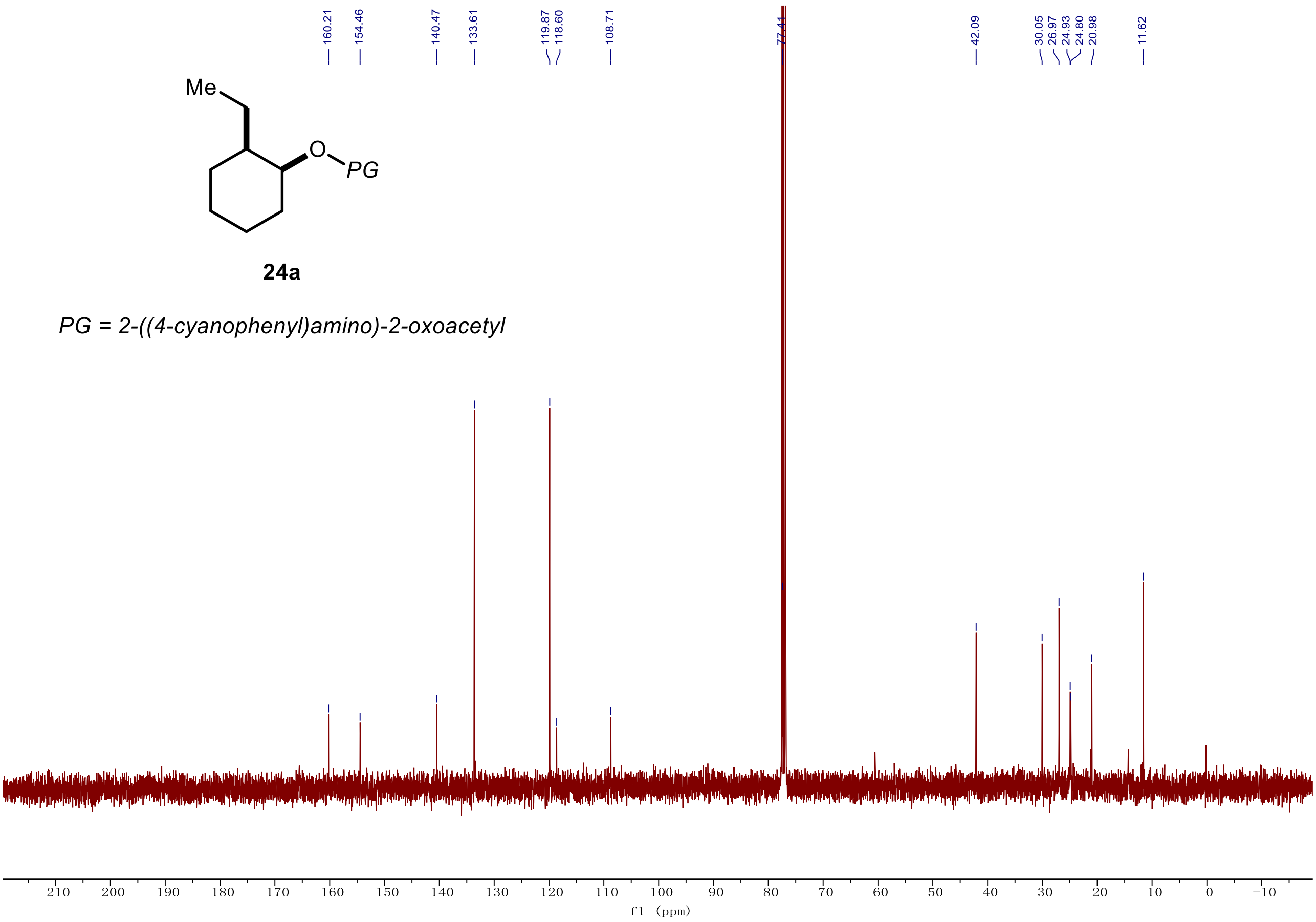

Figure S122 | ^{1}H NMR (500 MHz, $CDCl_3$) spectrum of a mixture of **24b** and **24′** (0.24:0.76).

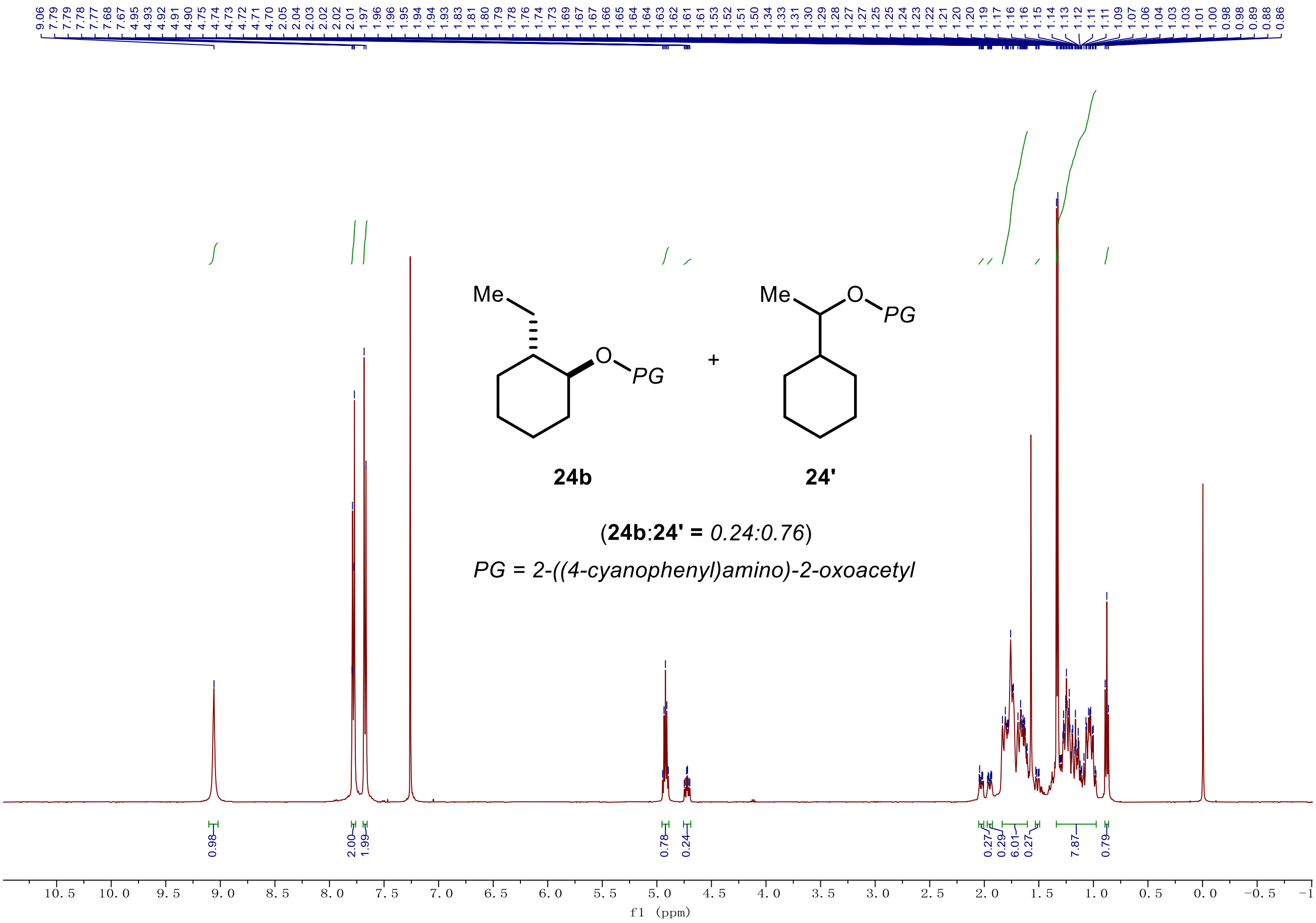

Figure S123 | ^{13}C NMR (101 MHz, $CDCl_3$) spectrum of a mixture of **24b** and **24'** (0.24:0.76).

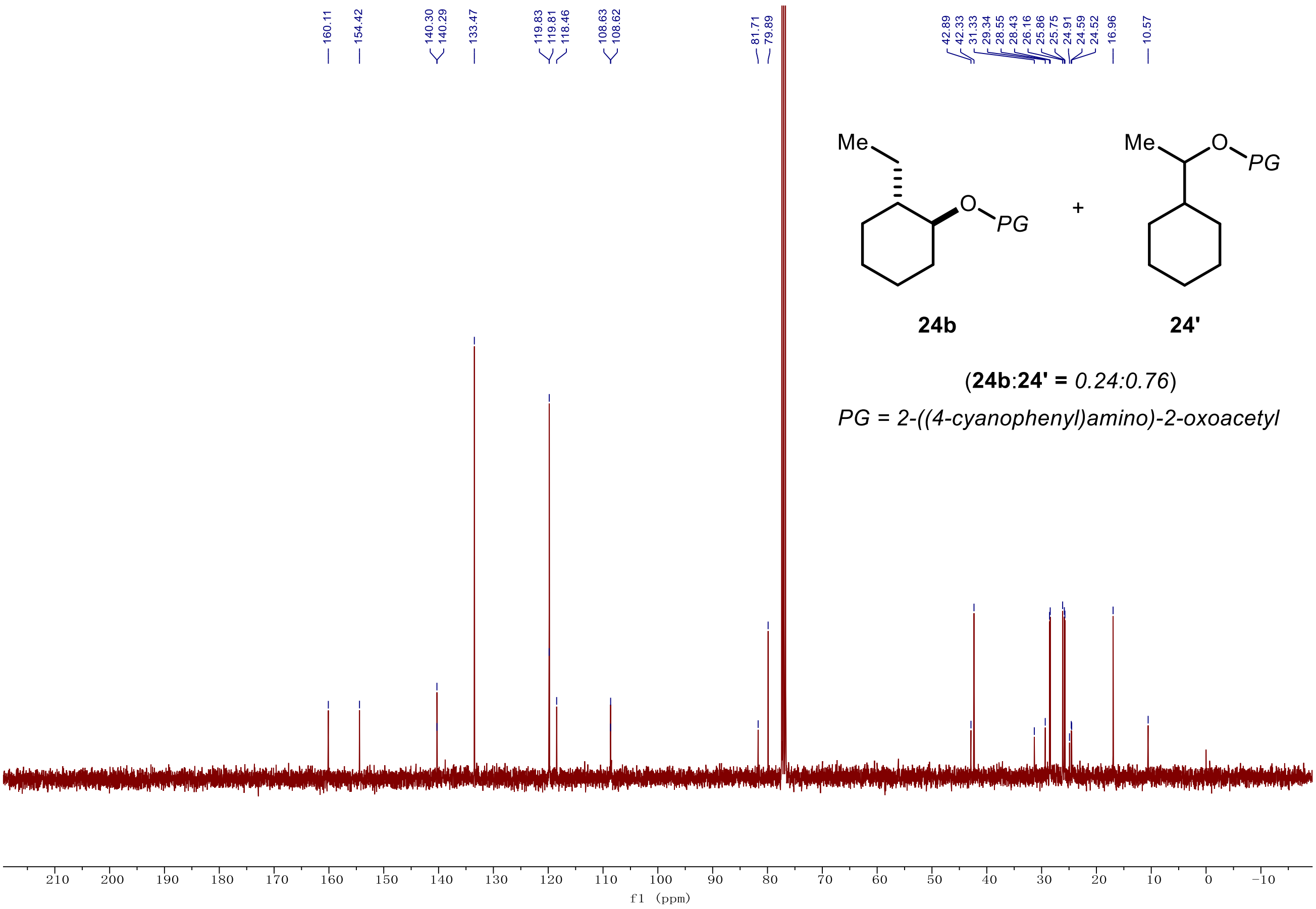

Figure S124 | ^{1}H NMR (400 MHz, $CDCl_3$) spectrum of **26a**.

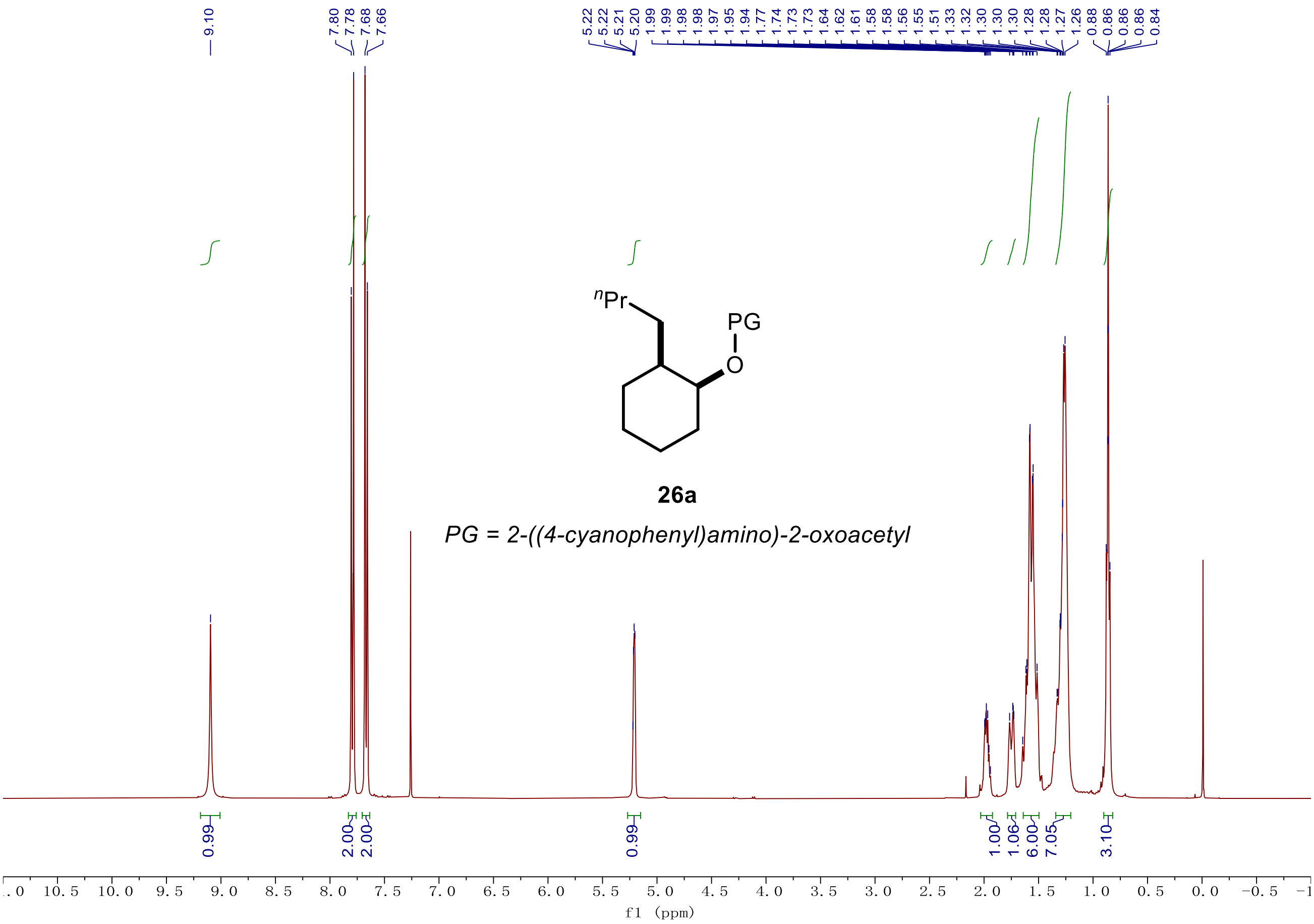

Figure S125 | ^{13}C NMR (101 MHz, $CDCl_3$) spectrum of **26a**.

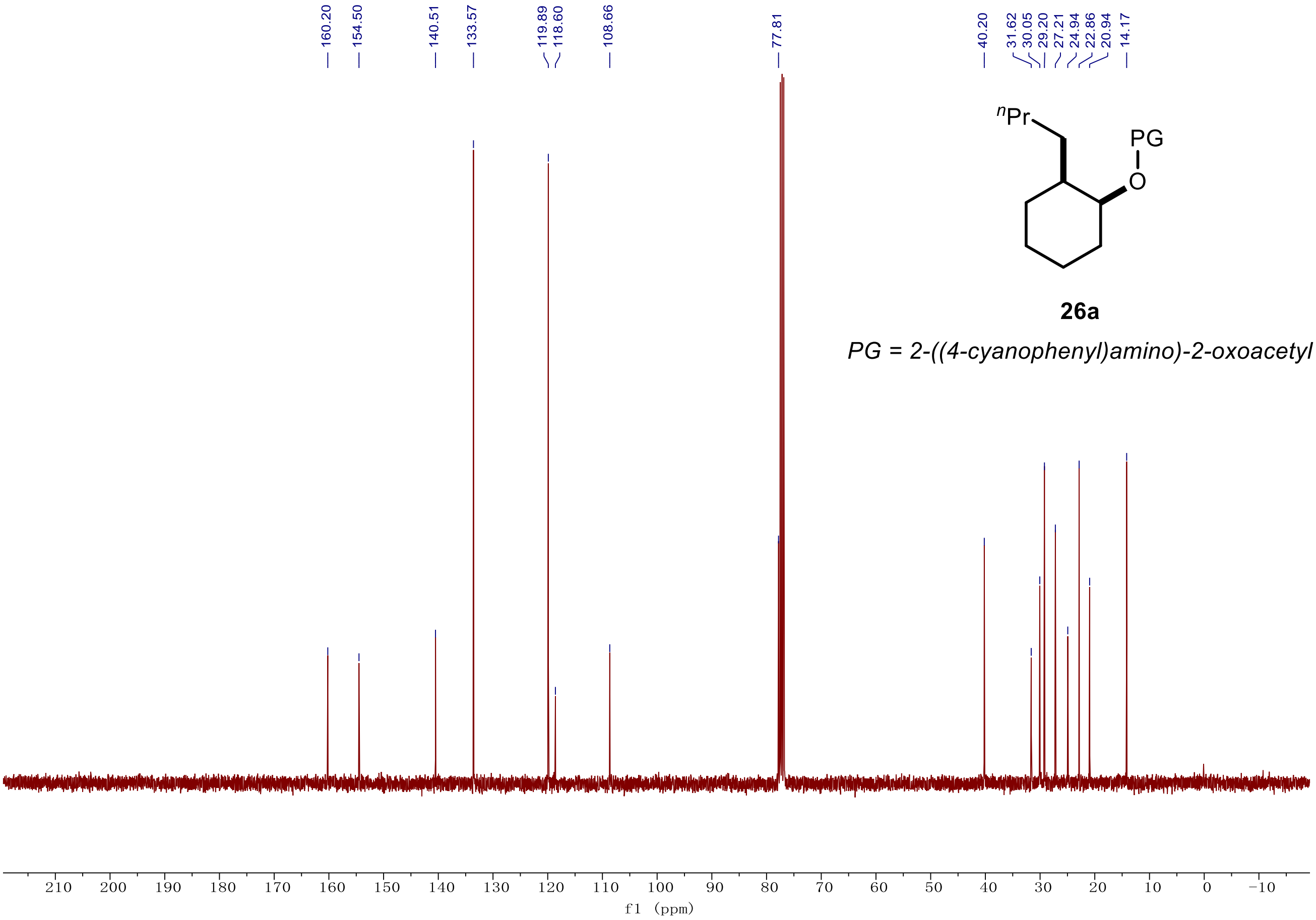

Figure S126 | ^{1}H NMR (400 MHz, $CDCl_3$) spectrum of **28**.

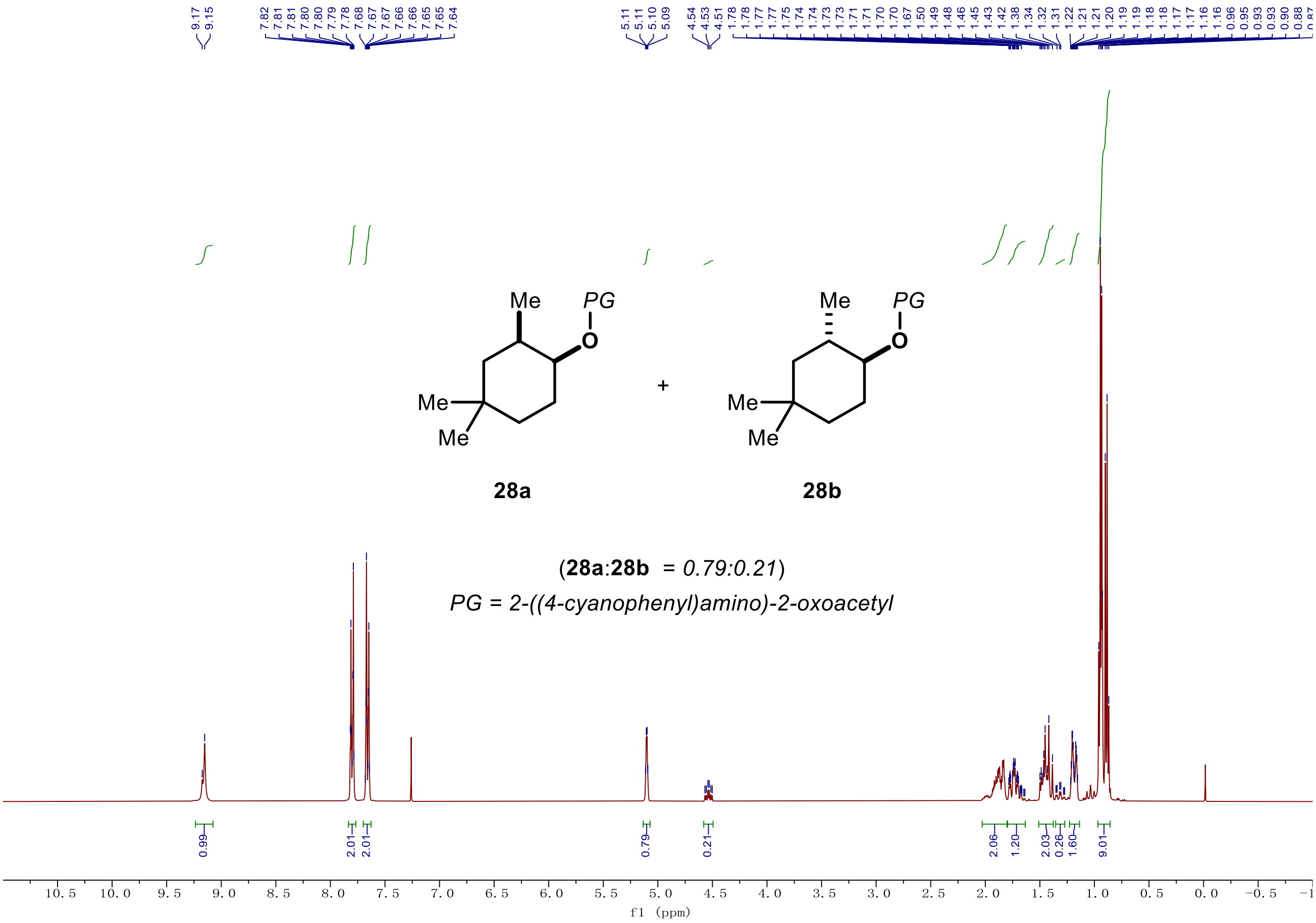

Figure S127 | ^{13}C NMR (101 MHz, $CDCl_3$) spectrum of **28**.

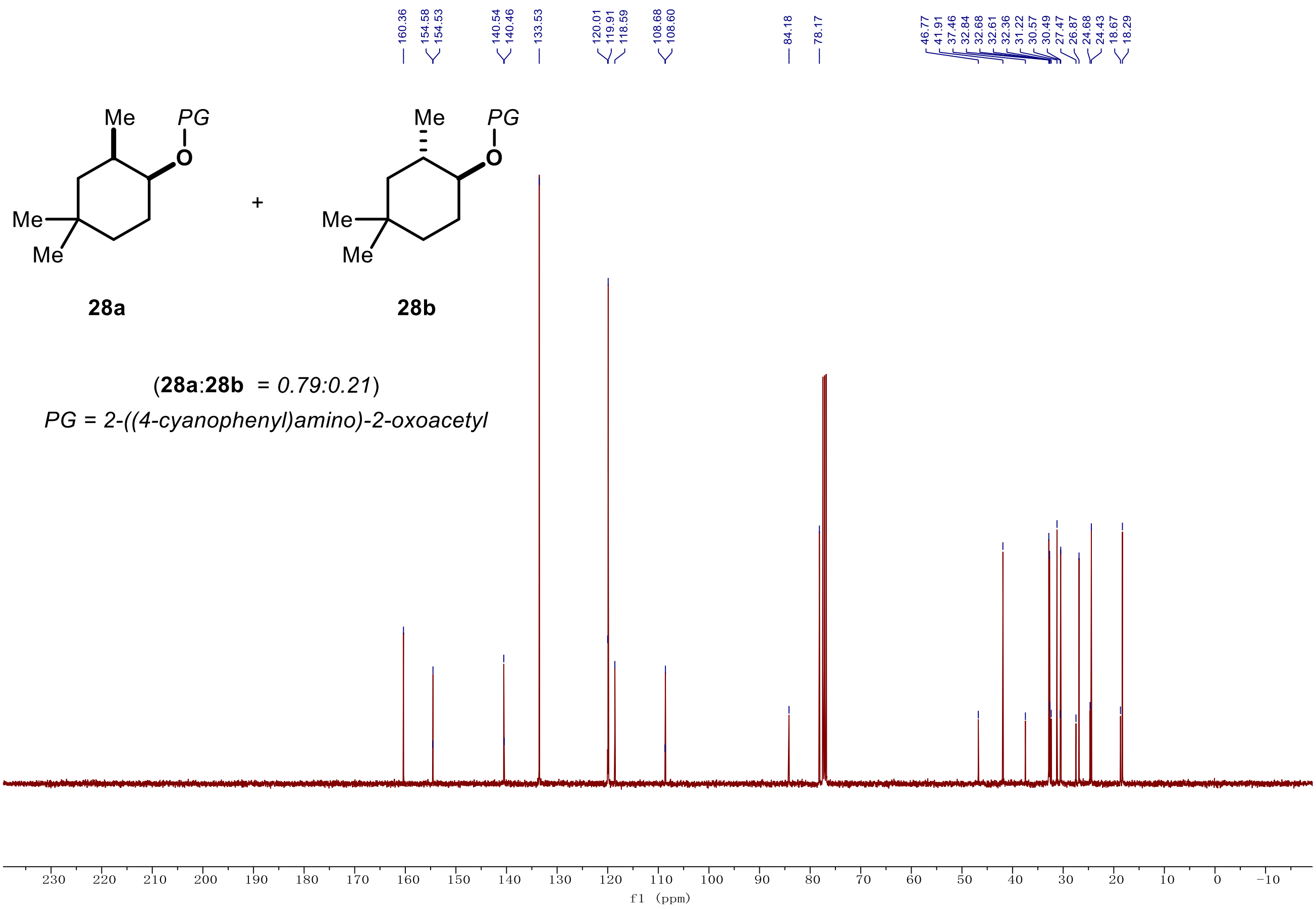

Figure S128 | ^{1}H NMR (400 MHz, $CDCl_3$) spectrum of **30a**.

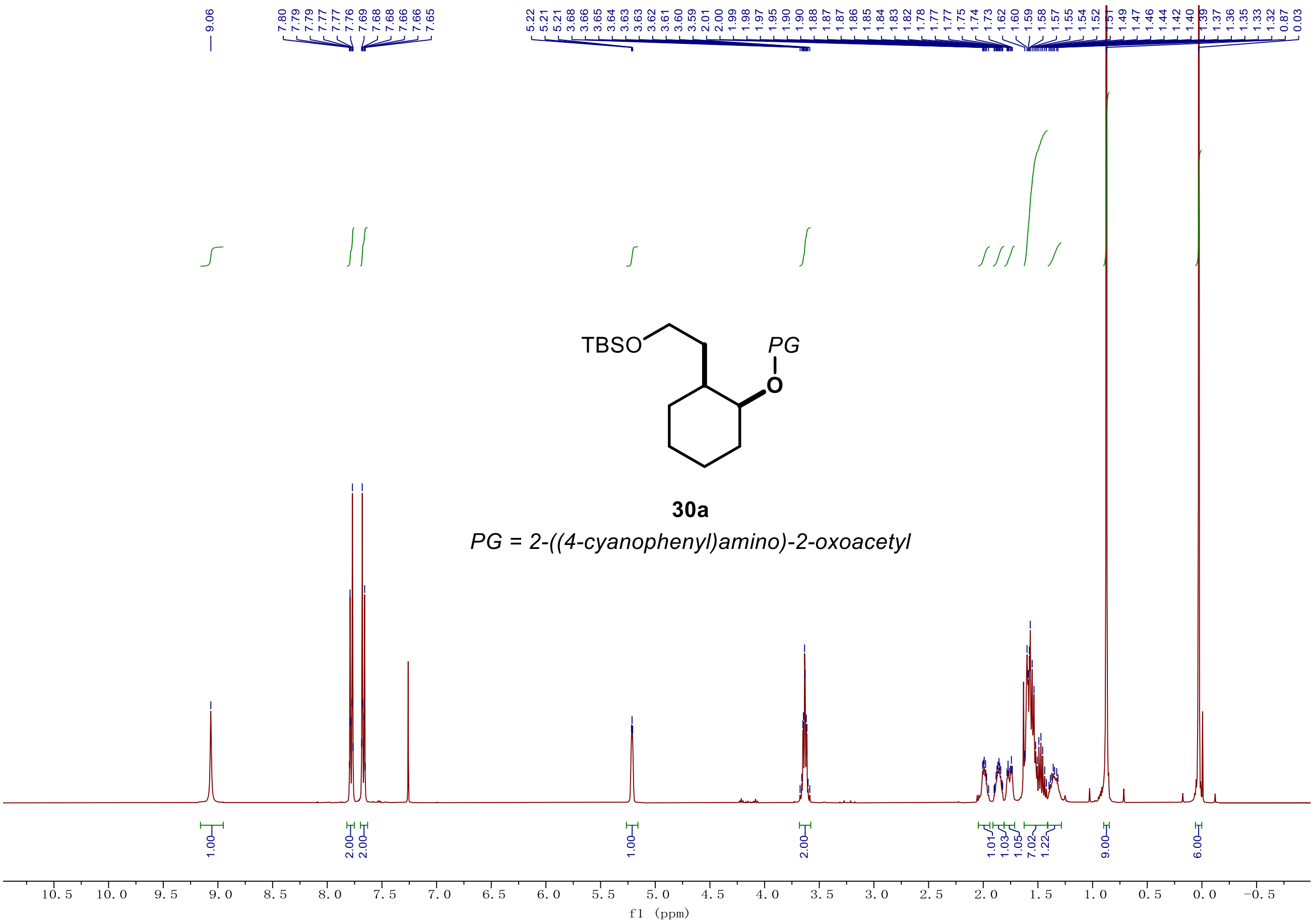

Figure S129 | ^{13}C NMR (101 MHz, $CDCl_3$) spectrum of **30a**.

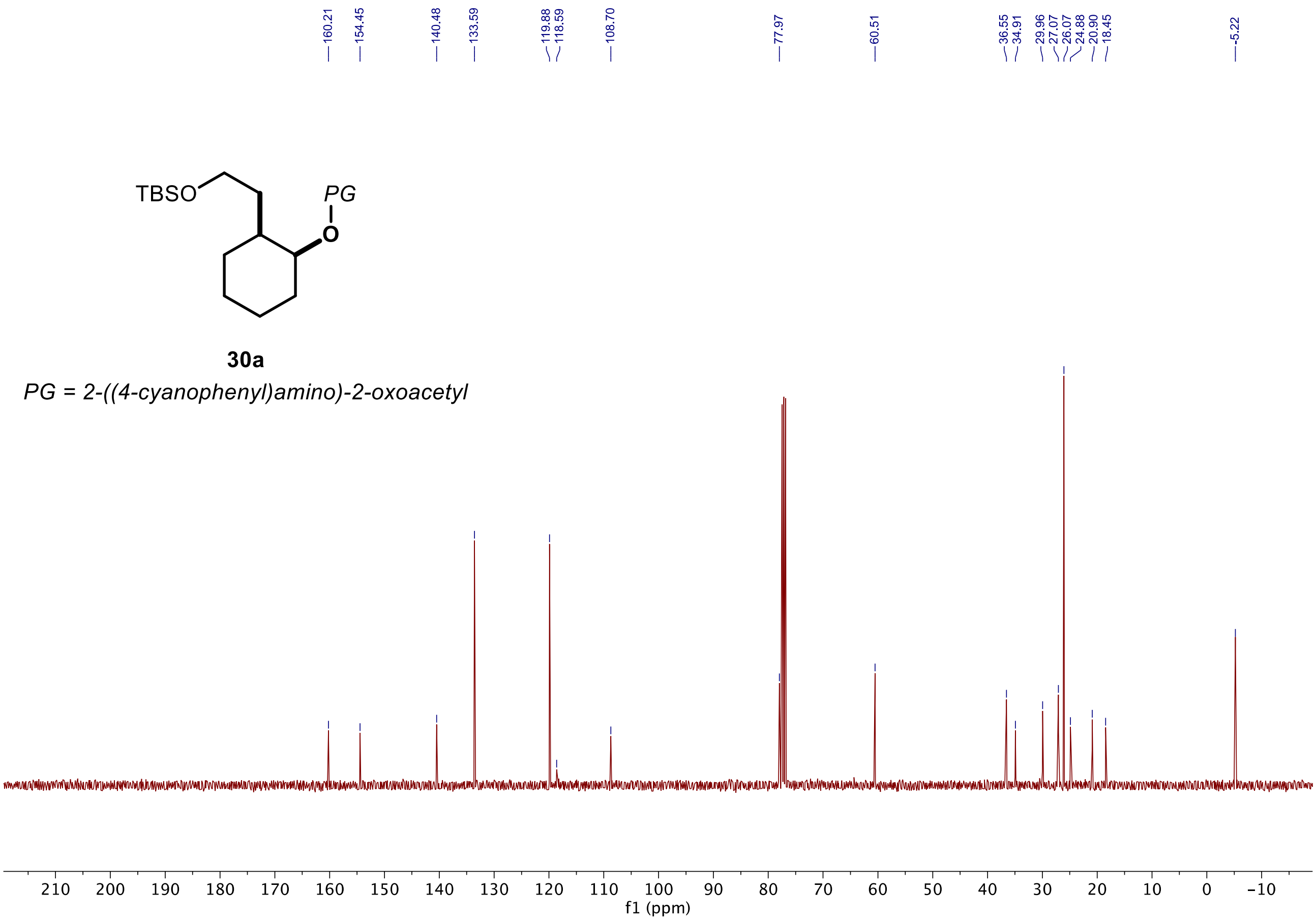

Figure S130 | ^{1}H NMR (400 MHz, $CDCl_3$) spectrum of **30b**.

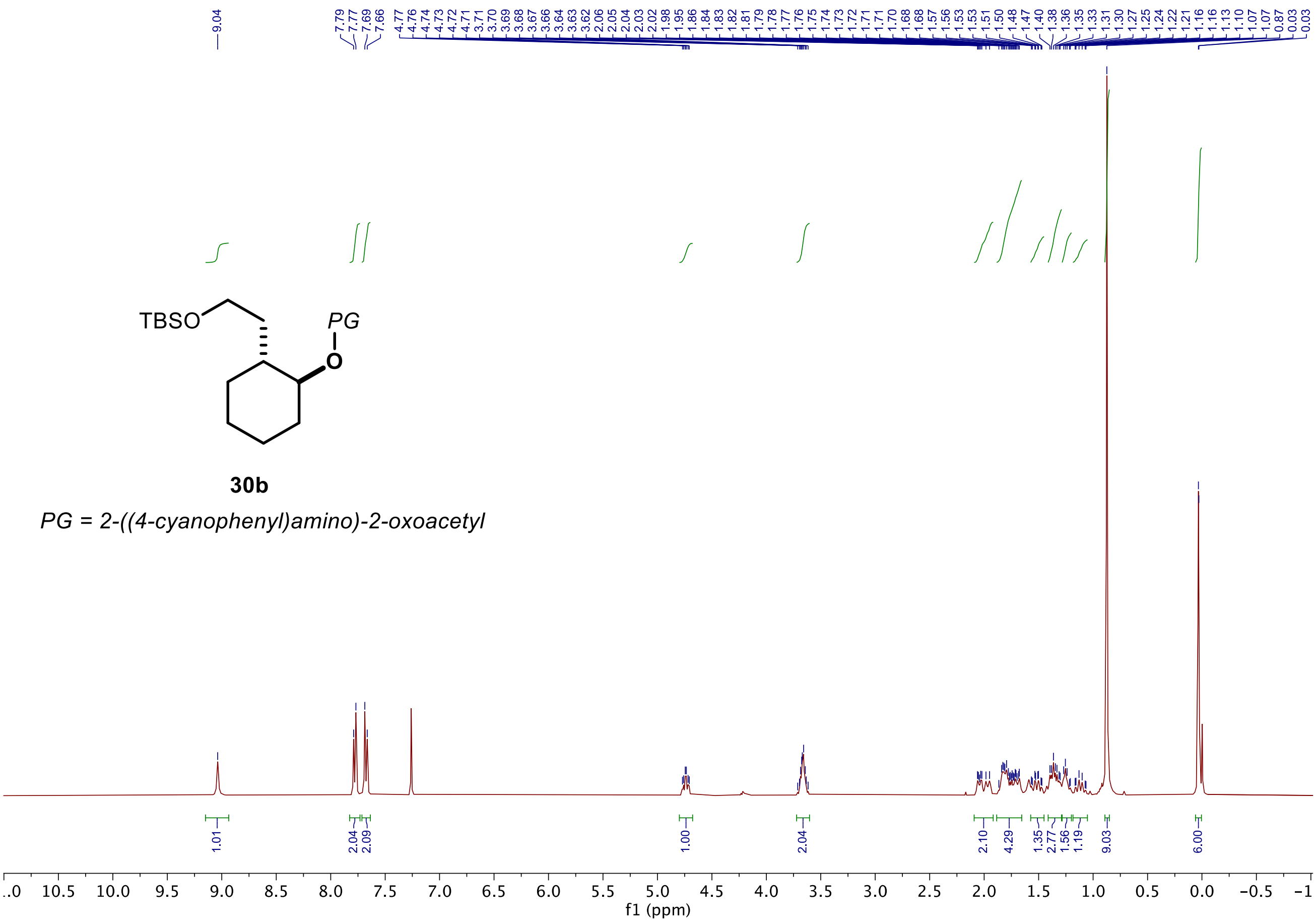

Figure S131 | ^{13}C NMR (101 MHz, $CDCl_3$) spectrum of **30b**.

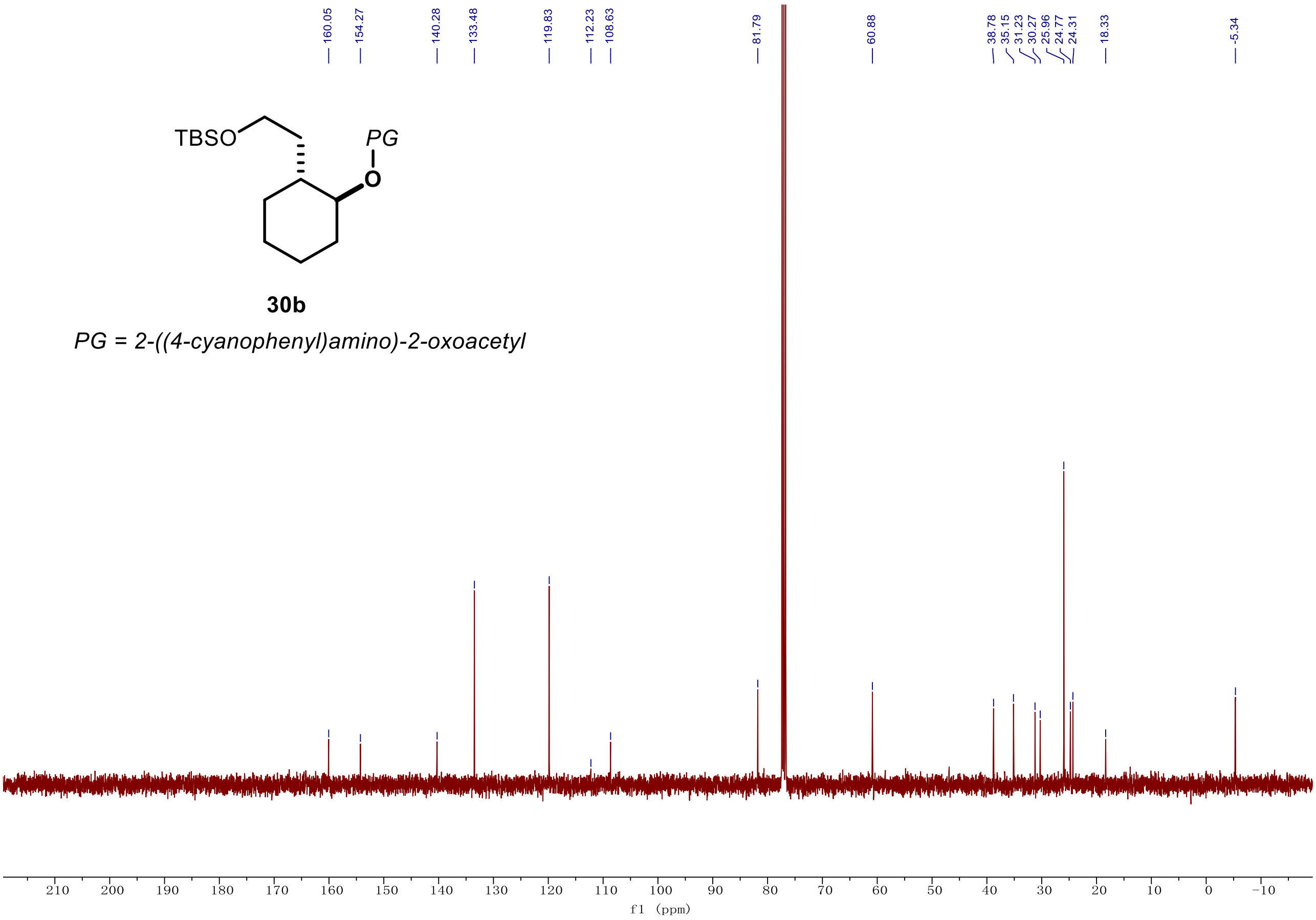

Figure S132 | ^{1}H NMR (400 MHz, $CDCl_3$) spectrum of **32a**.

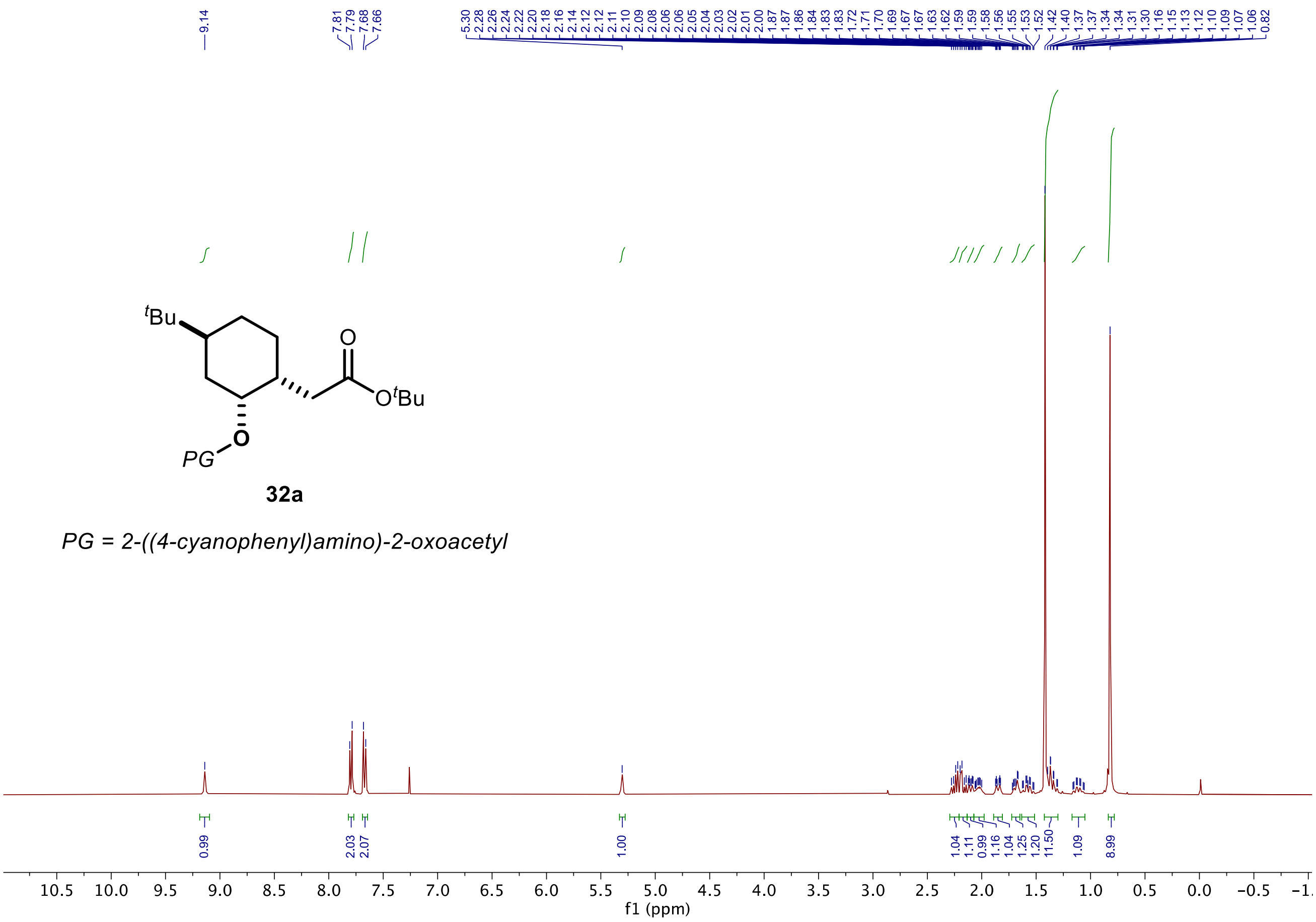

Figure S133 | ^{13}C NMR (101 MHz, $CDCl_3$) spectrum of **32a**.

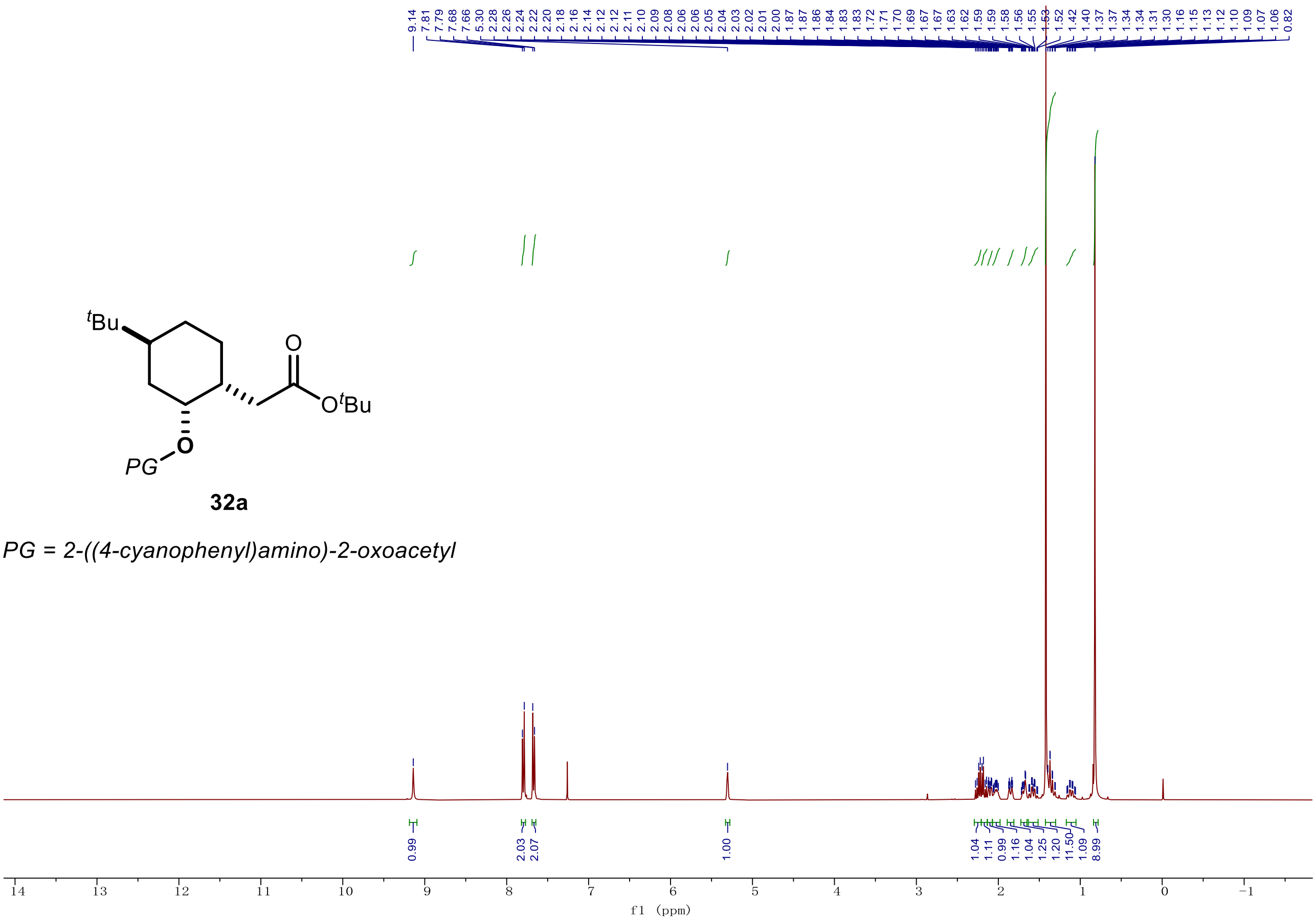

Figure S134 | ^{1}H NMR (500 MHz, $CDCl_3$) spectrum of a mixture of **32b**, **32c**, and **32d** (0.64:0.19:0.17).

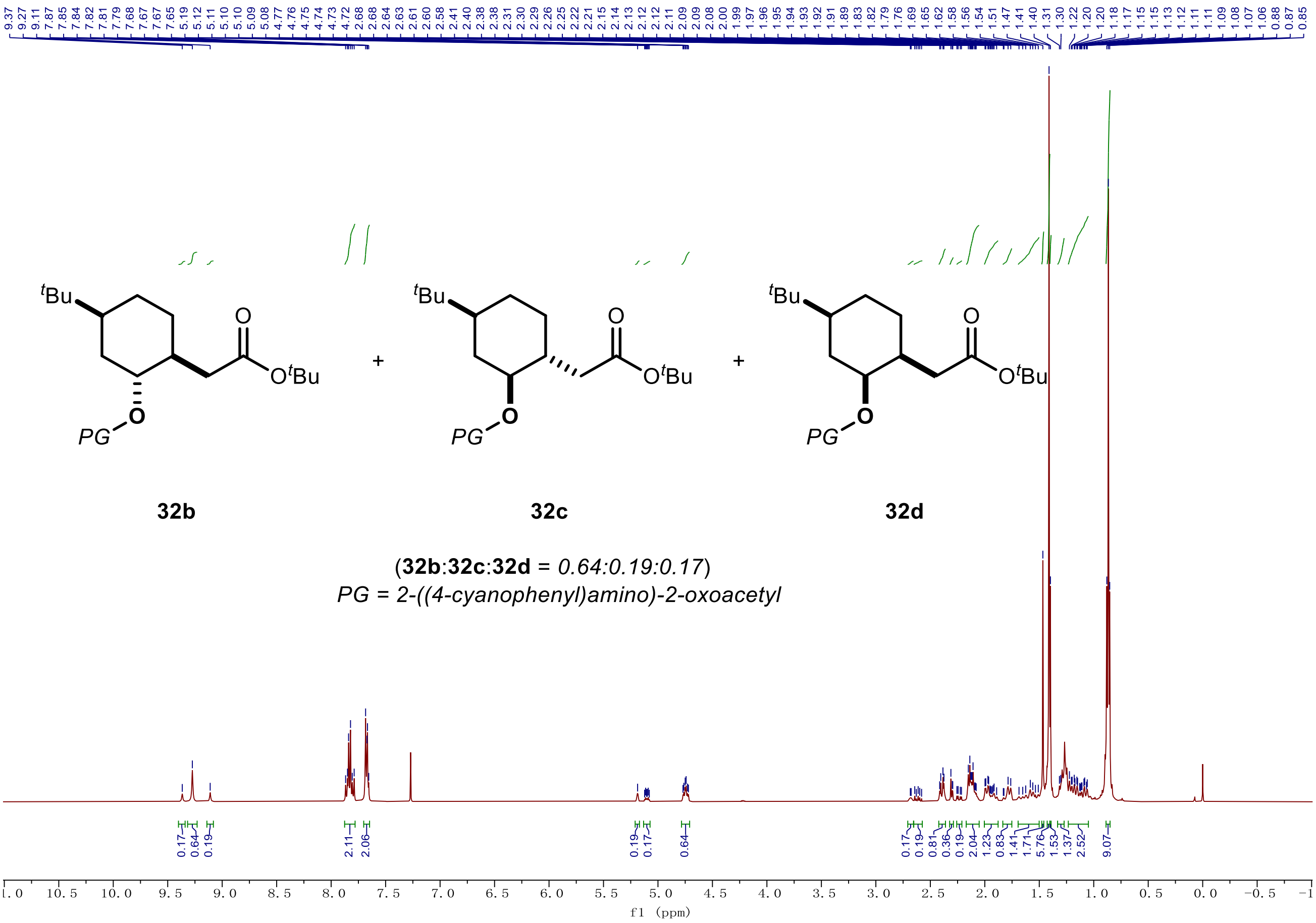

Figure S135 | ^{13}C NMR (101 MHz, $CDCl_3$) spectrum of a mixture of **32b**, **32c**, and **32d** (0.64:0.19:0.17).

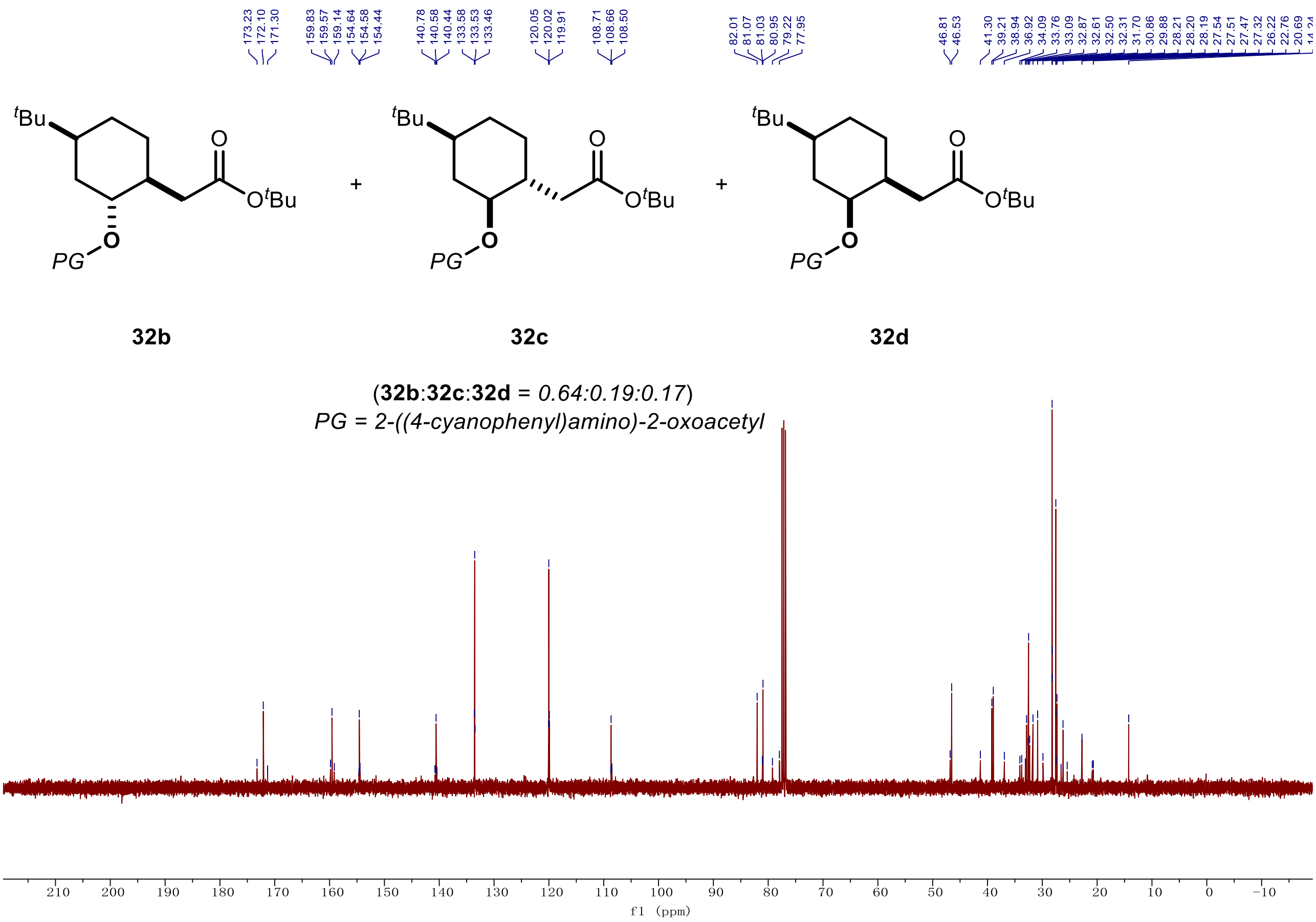

Figure S136 | ^{1}H NMR (600 MHz, $CDCl_3$) spectrum of **34a**.

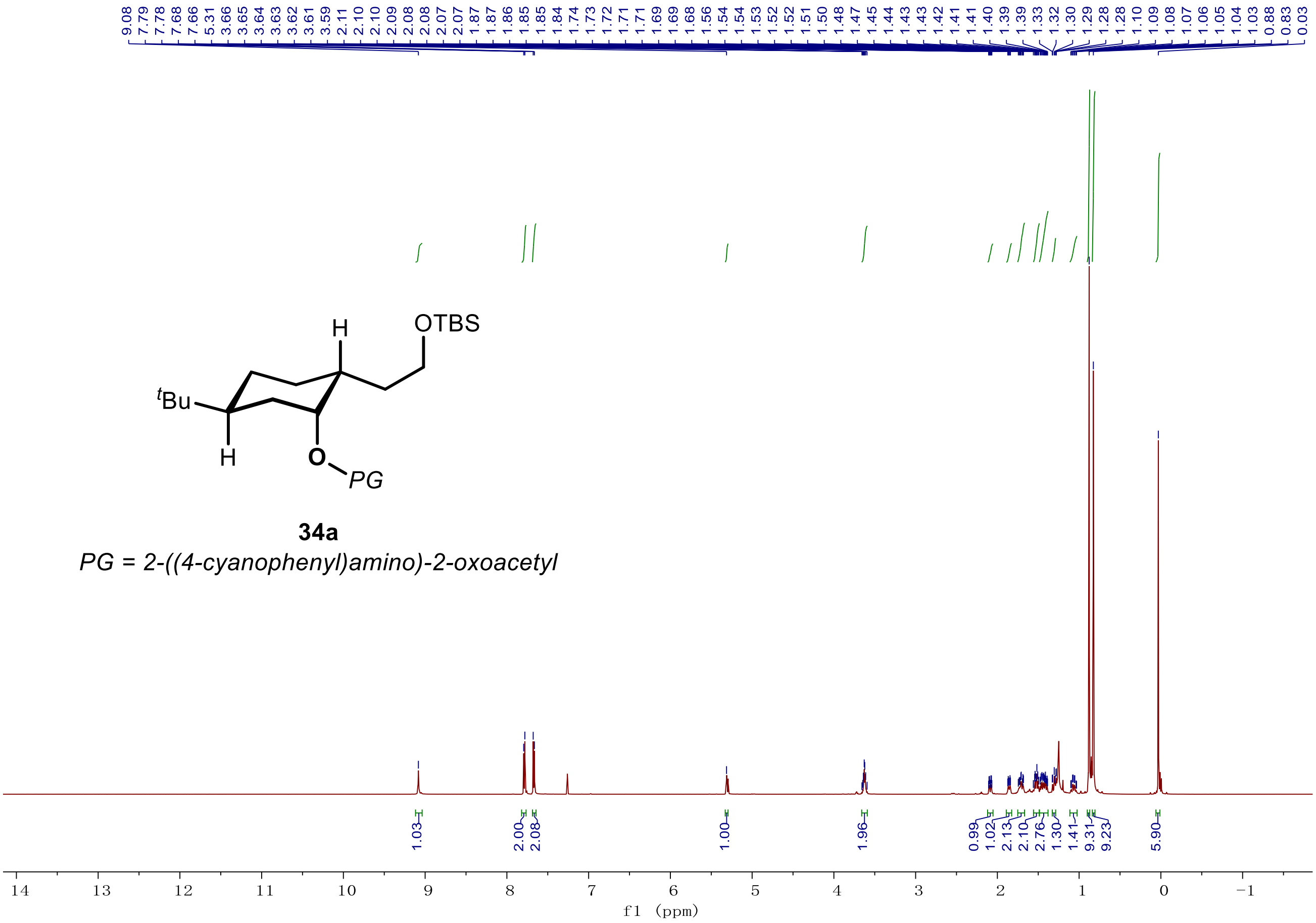

Figure S137 | ^{13}C NMR (151 MHz, CDCl$_3$) spectrum of **34a**.

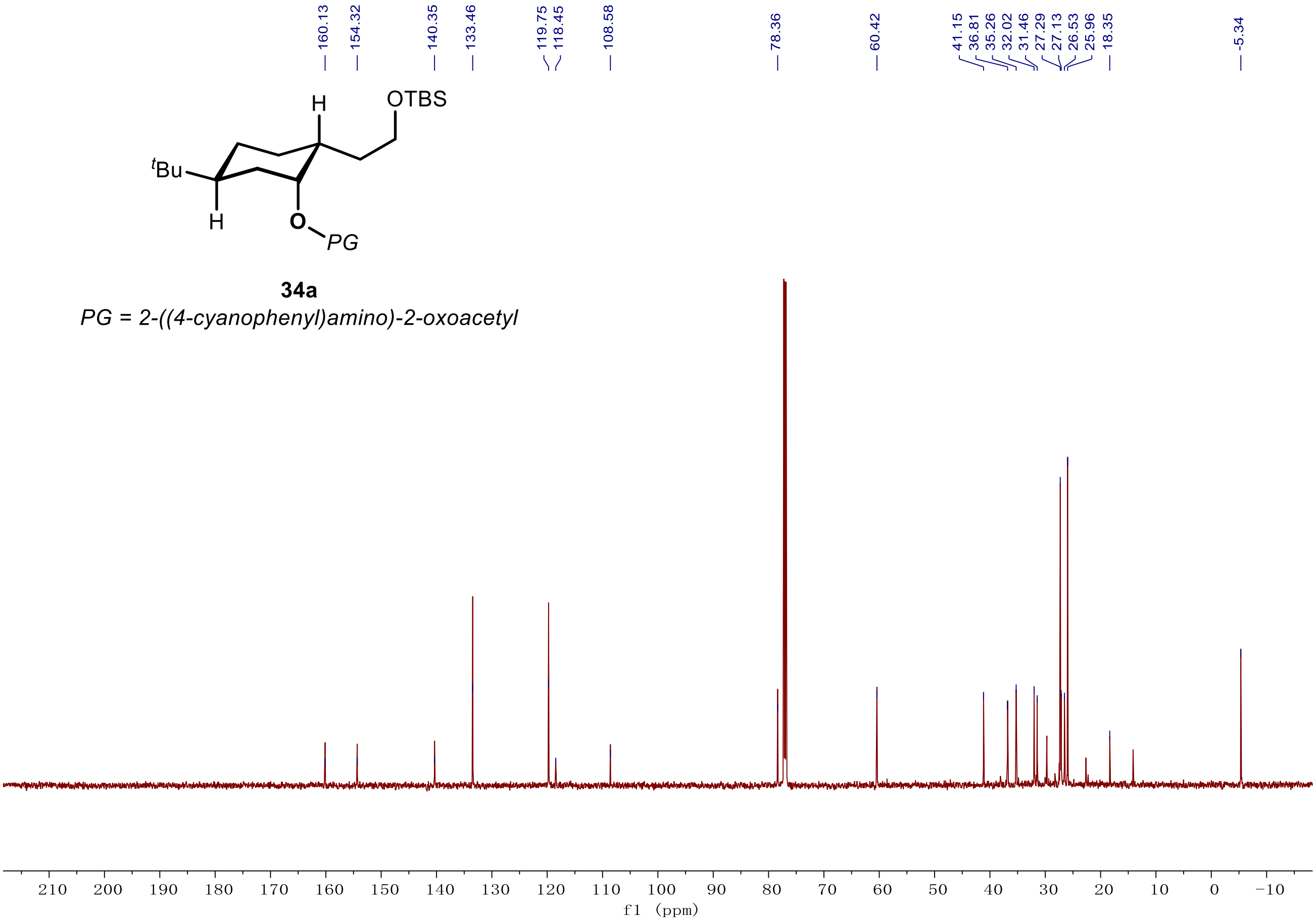

Figure S138 | ^{1}H NMR (600 MHz, CDCl$_3$) spectrum of a mixture of **34b** and **33**.

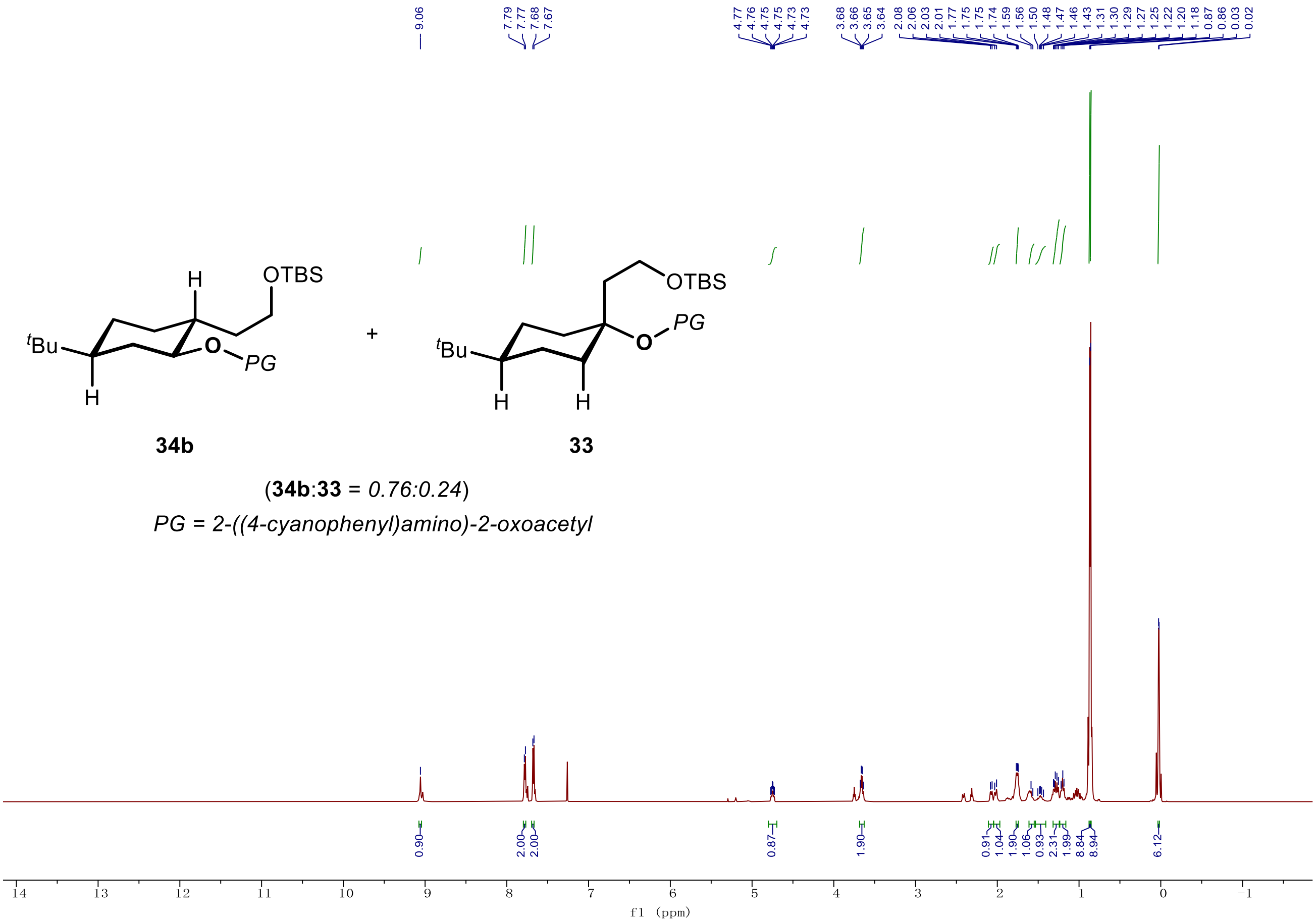

Figure S139 | ^{13}C NMR (151 MHz, $CDCl_3$) spectrum of a mixture of **34b** and **33**.

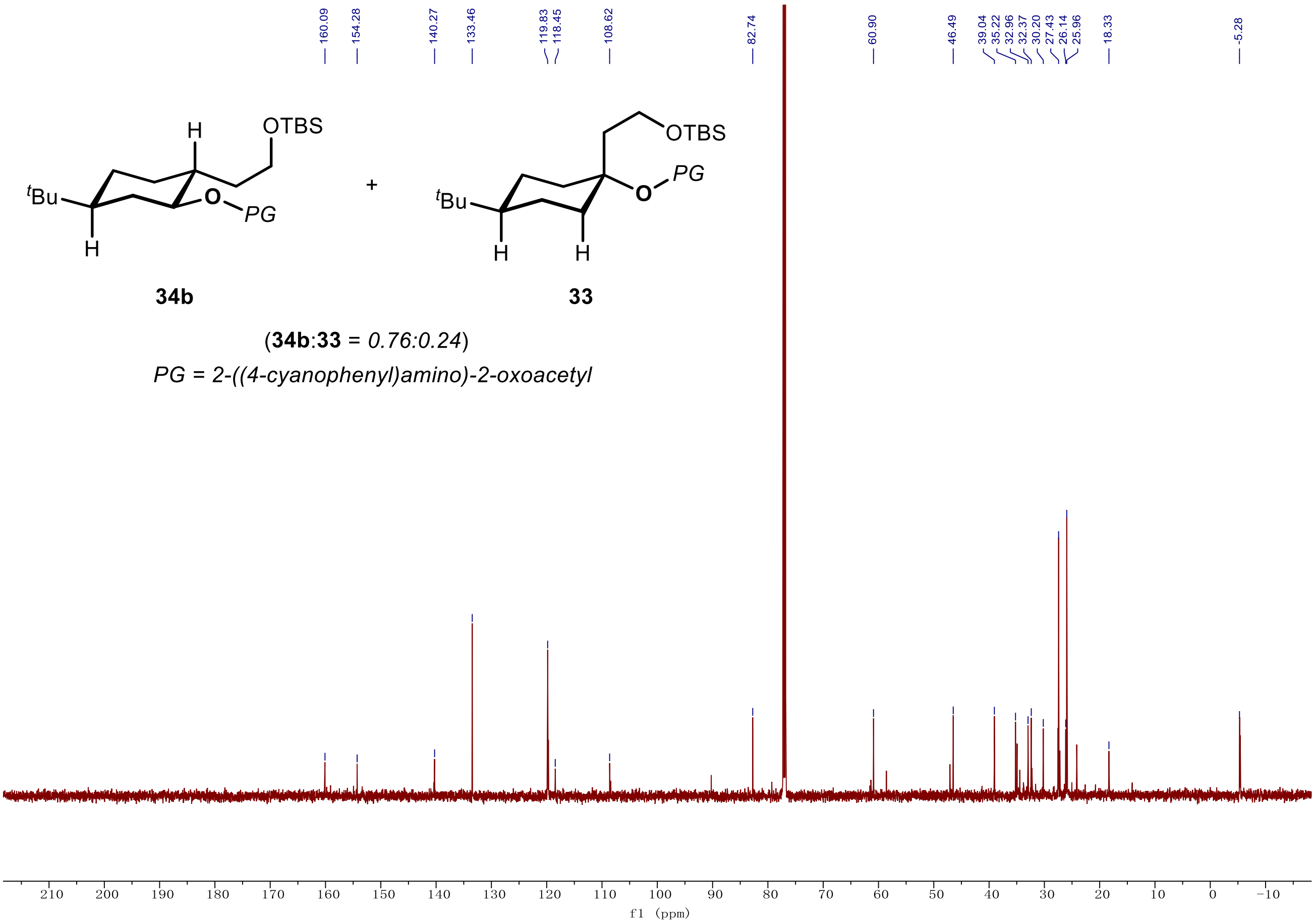

Figure S140 | ^{1}H NMR (400 MHz, $CDCl_3$) spectrum of **36a** (*mixed with 5.5% of 4-aminobenzonitrile*).

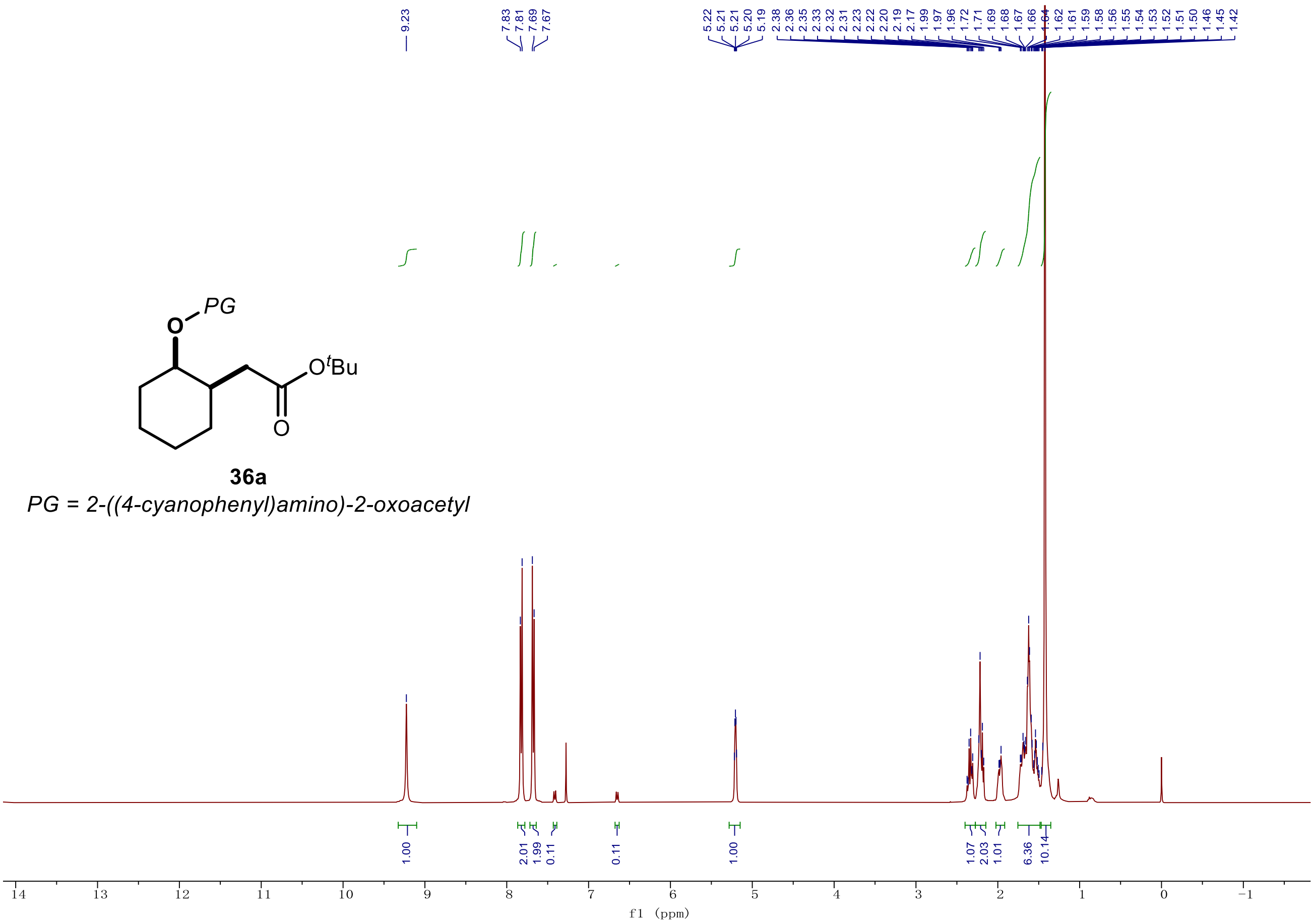

Figure S141 | ^{13}C NMR (101 MHz, $CDCl_3$) spectrum of **36a** (*mixed with 5.5% of 4-aminobenzonitrile*).

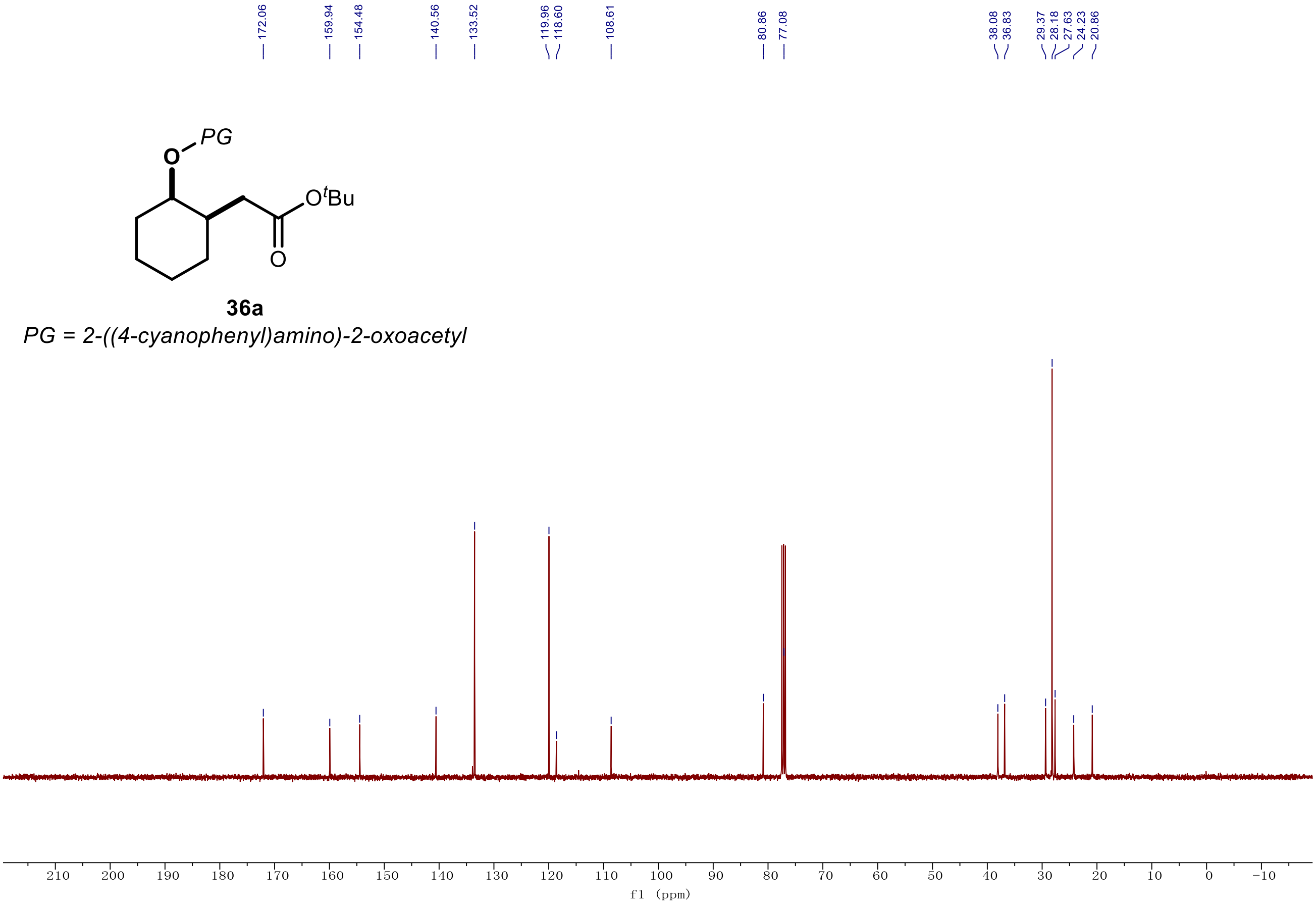

Figure S142 | ^{1}H NMR (400 MHz, $CDCl_3$) spectrum of **36b**.

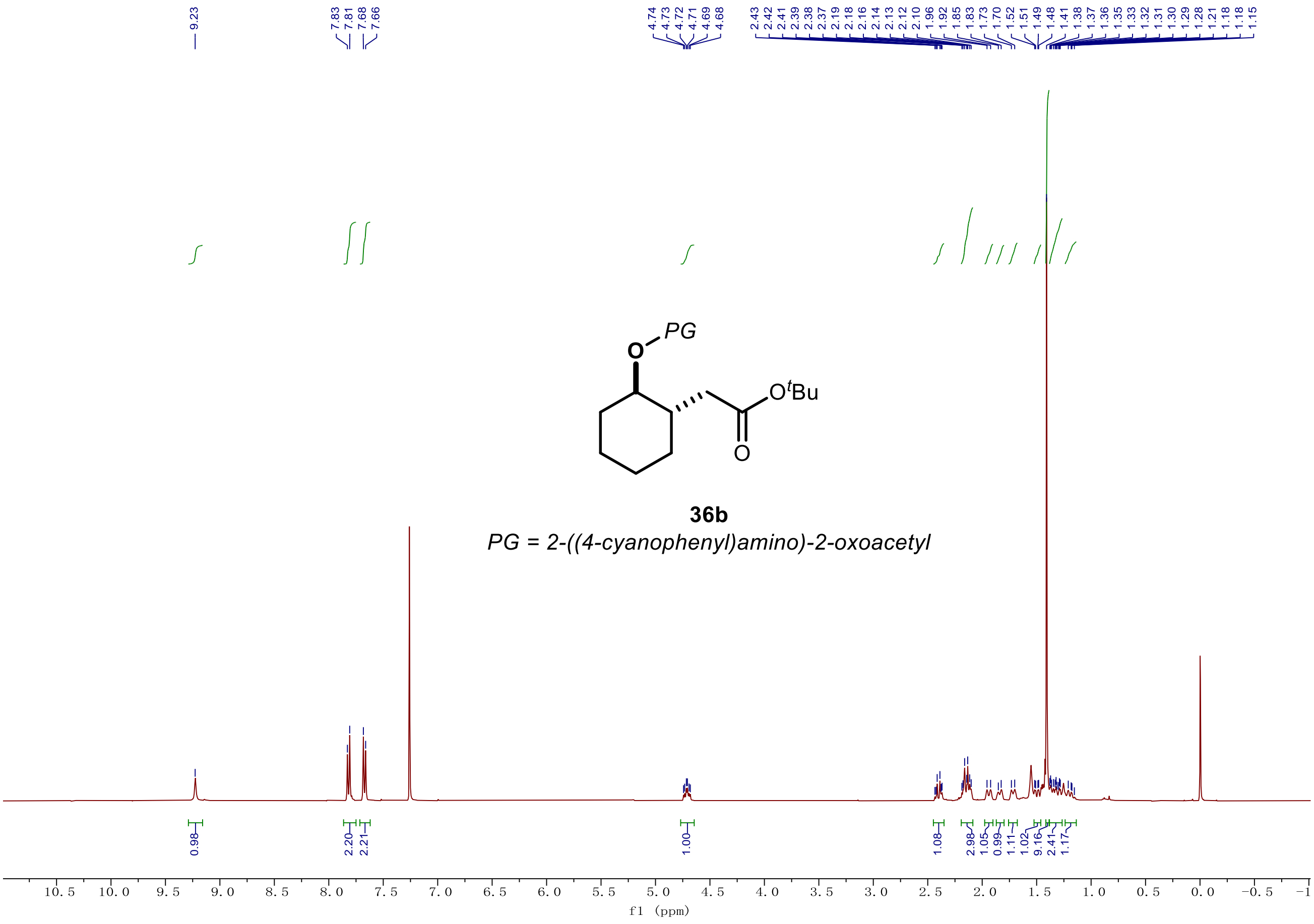

Figure S143 | ^{13}C NMR (101 MHz, $CDCl_3$) spectrum of **36b**.

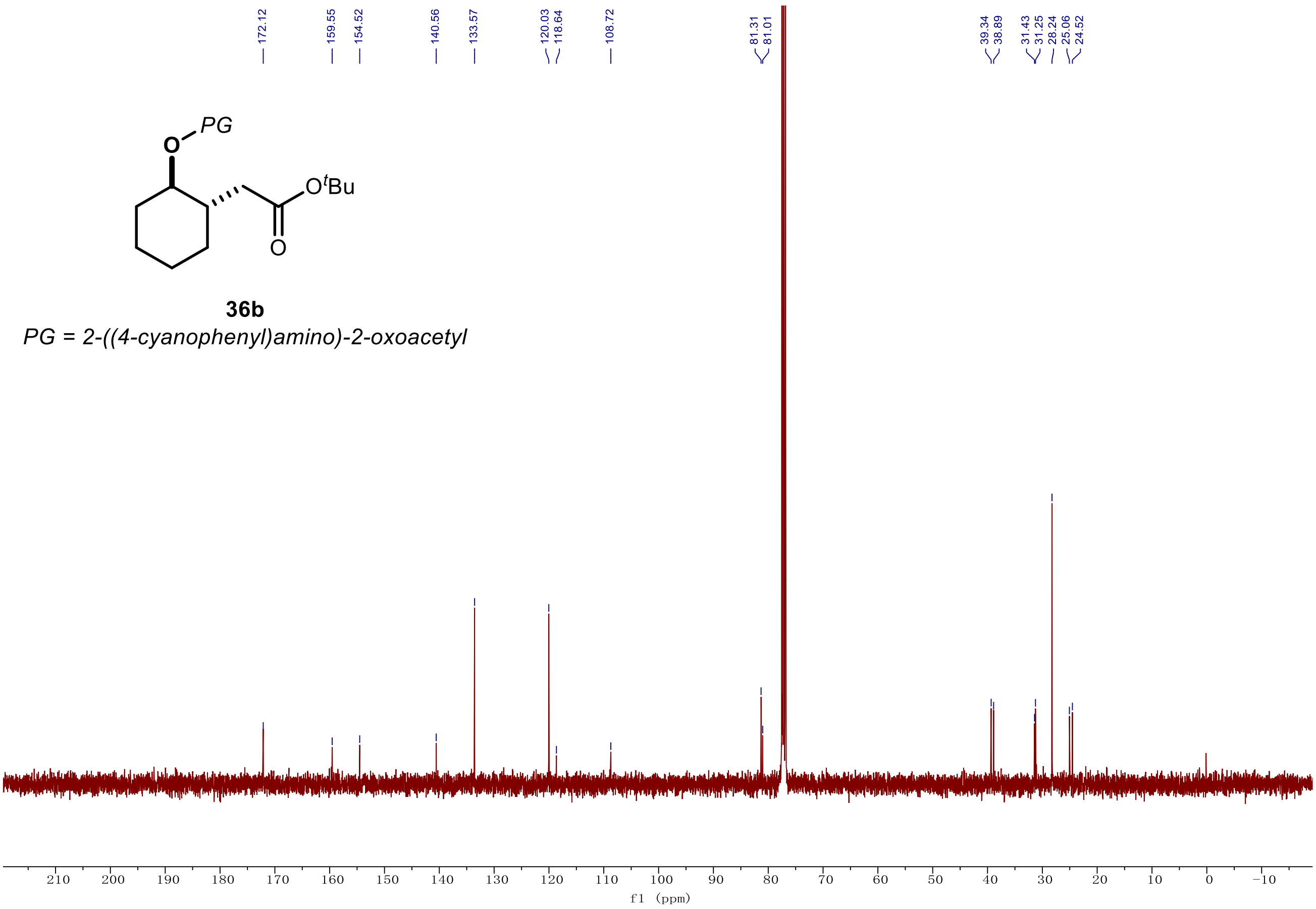

Figure S144 | ^{1}H NMR (400 MHz, $CDCl_3$) spectrum of **38**.

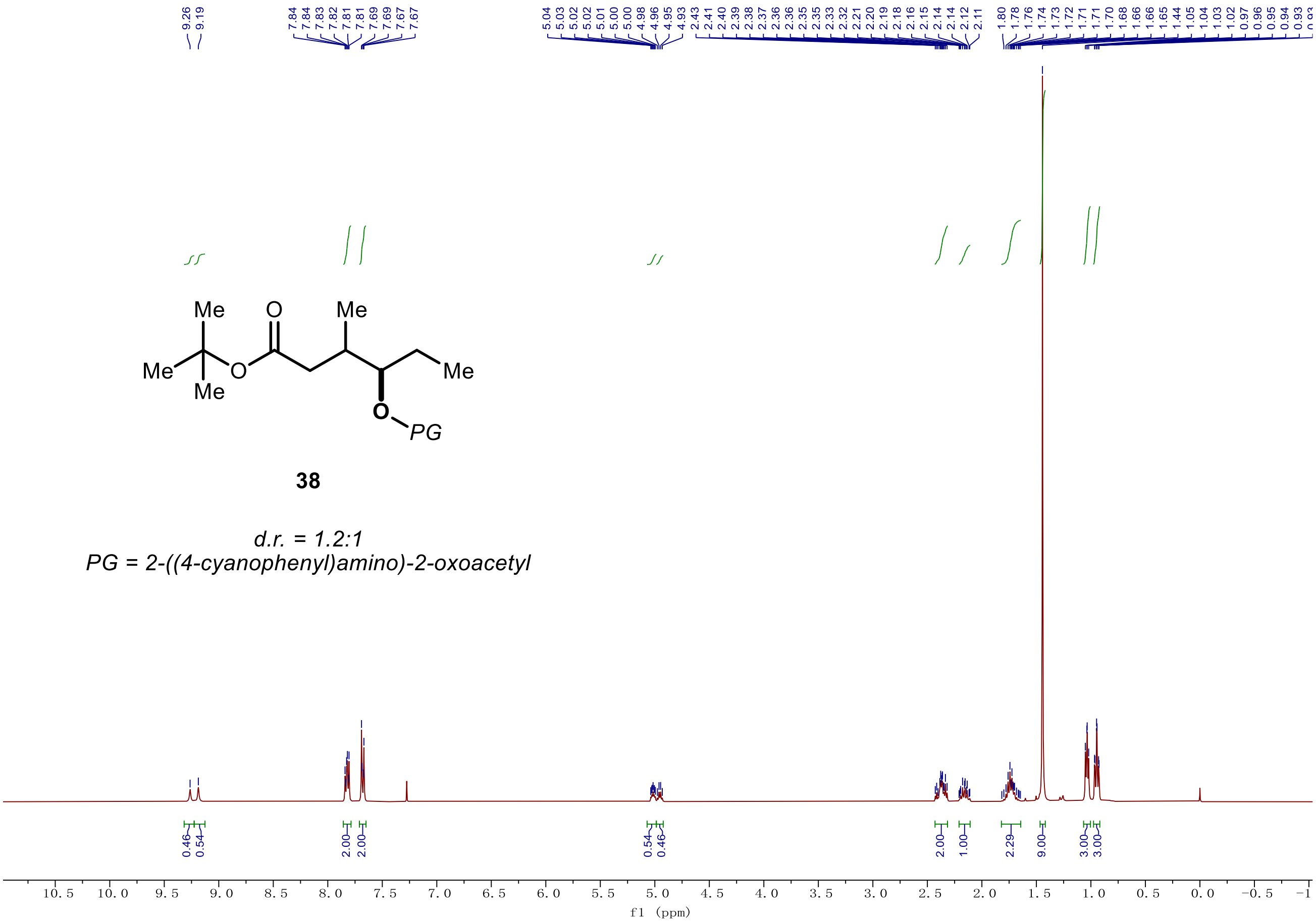

Figure S145 | ^{13}C NMR (101 MHz, $CDCl_3$) spectrum of **38**.

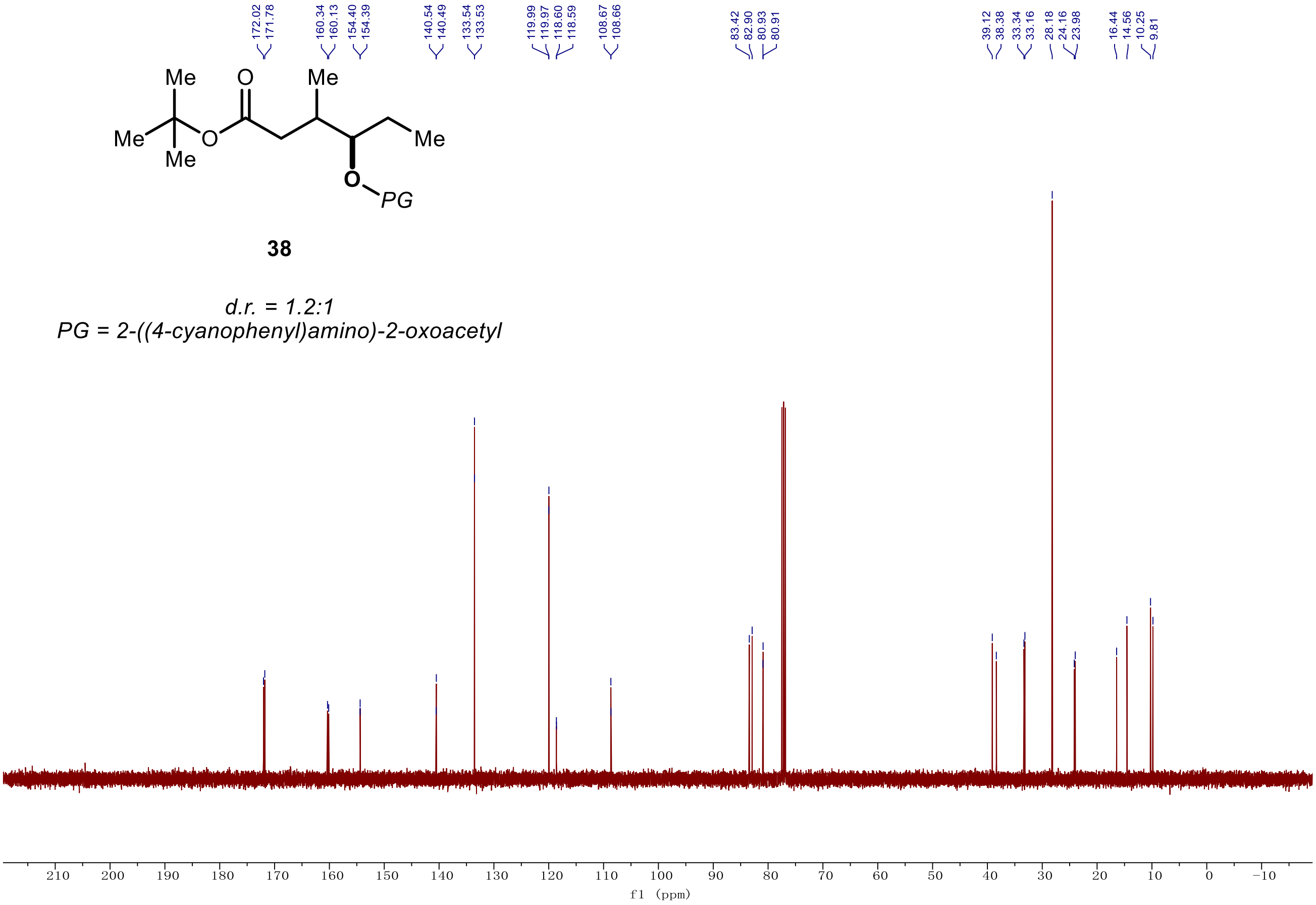

Figure S146 | ^{1}H NMR (600 MHz, $CDCl_3$) spectrum of **40-*diastereomer 1***.

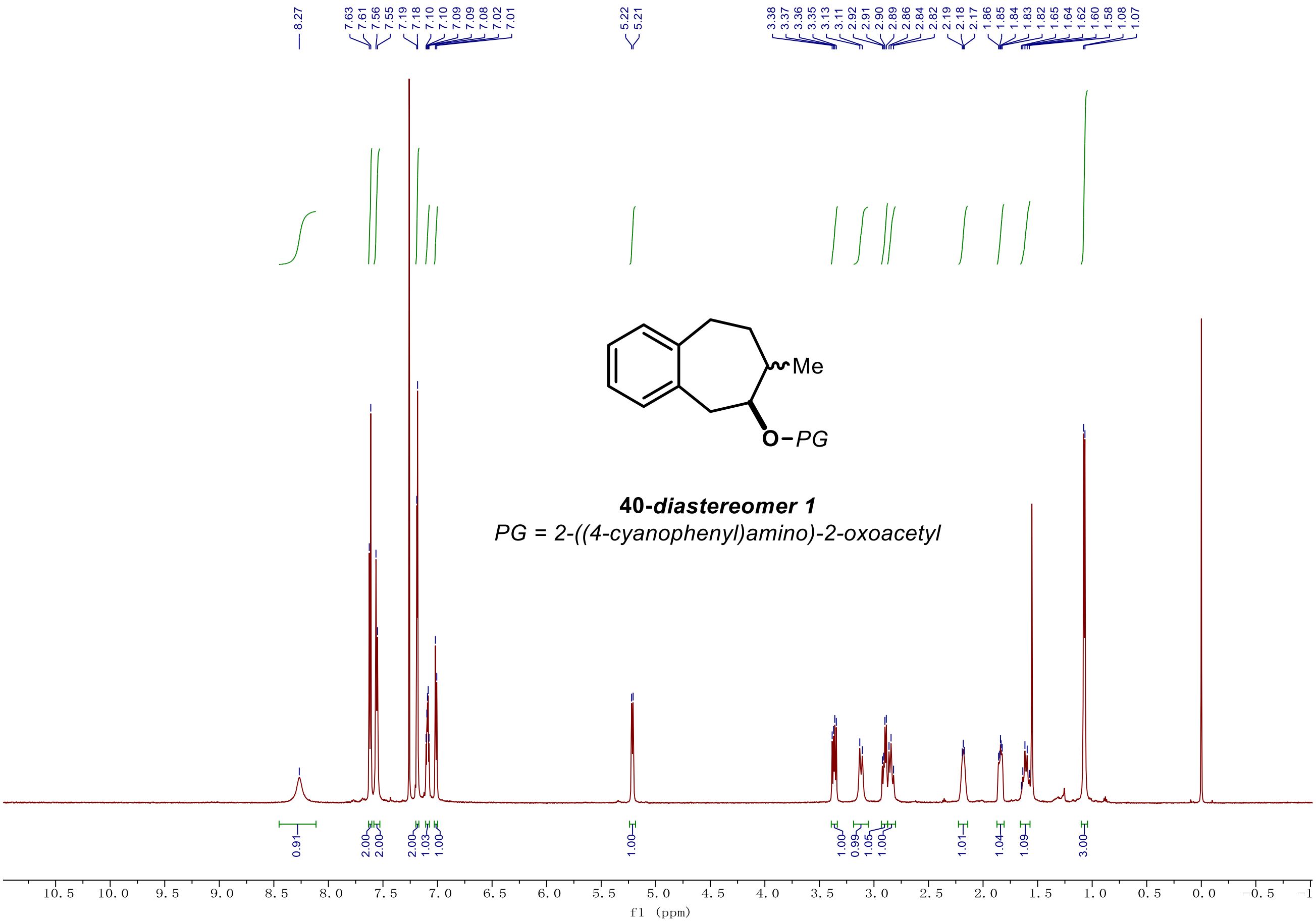

Figure S147 | ^{13}C NMR (151 MHz, $CDCl_3$) spectrum of **40-*diastereomer 1***.

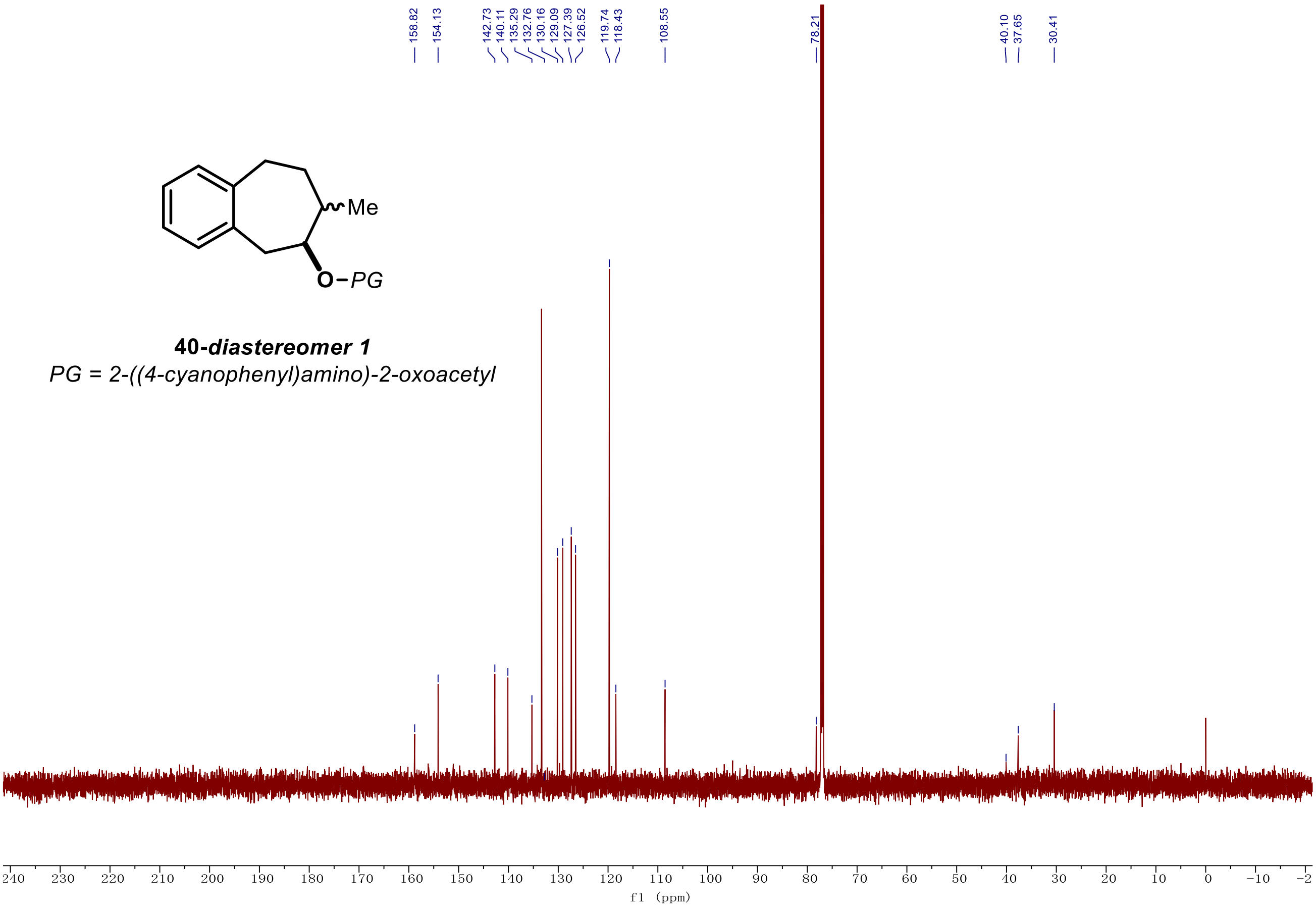

Figure S148 | ^{1}H NMR (500 MHz, $CDCl_3$) spectrum of a mixture of **40-*diastereomer 1***, **40-*diastereomer 2***, and**39**.

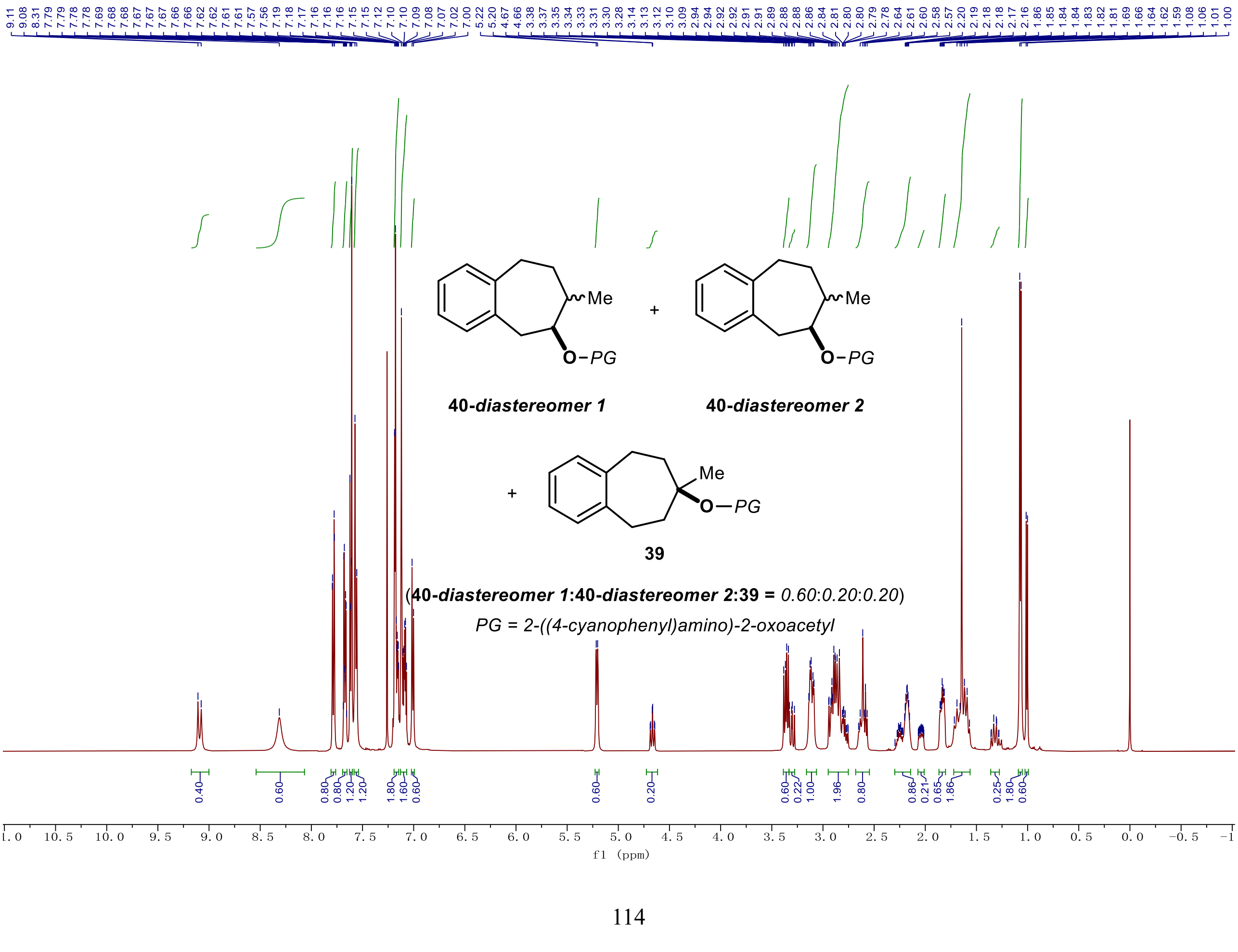

Figure S149 | ^{13}C NMR (101 MHz, $CDCl_3$) spectrum of a mixture of **40-*diastereomer 1***, **40-*diastereomer 2***, and **39**.

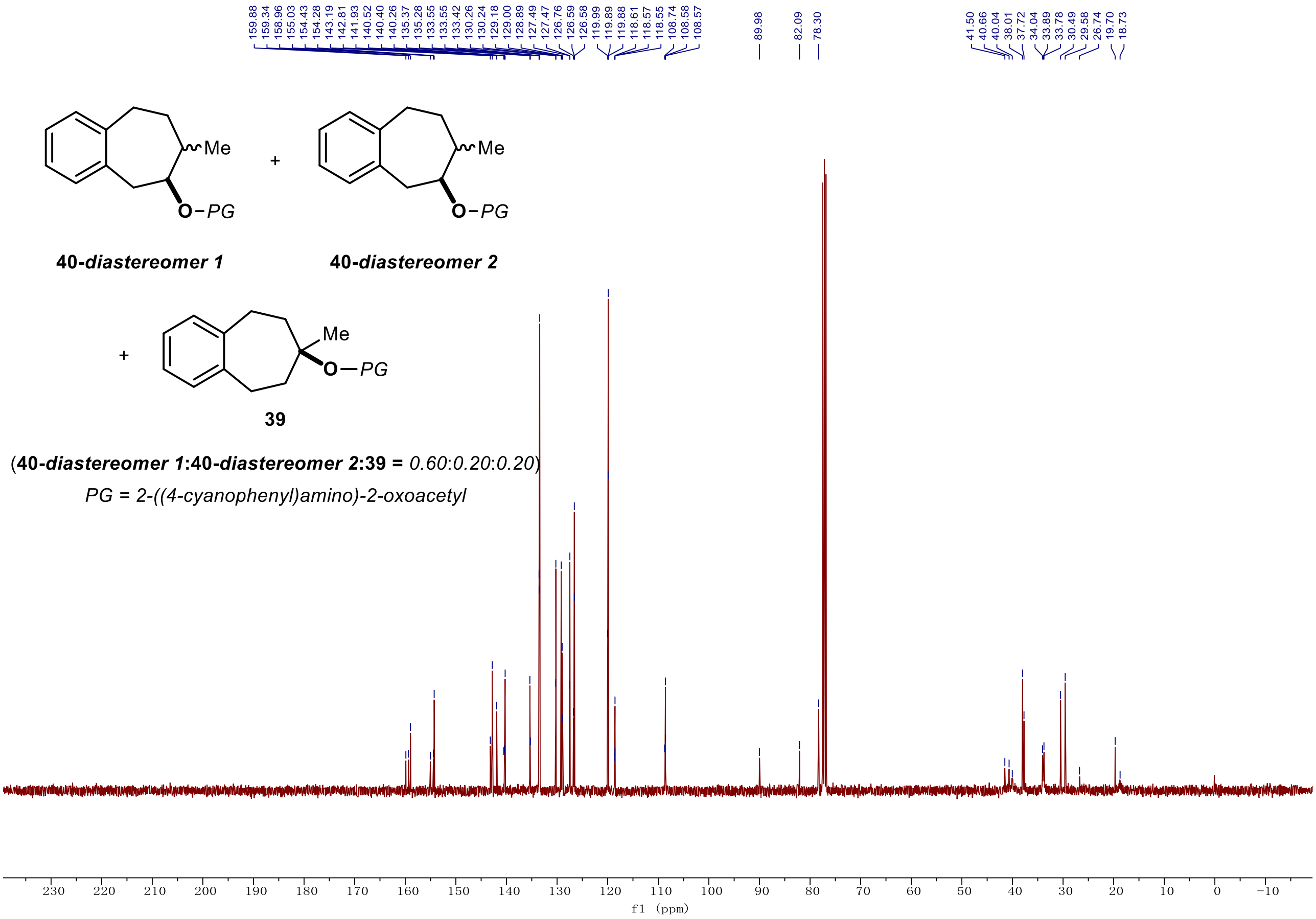

Figure S150 | ^{1}H NMR (400 MHz, $CDCl_3$) spectrum of **42**.

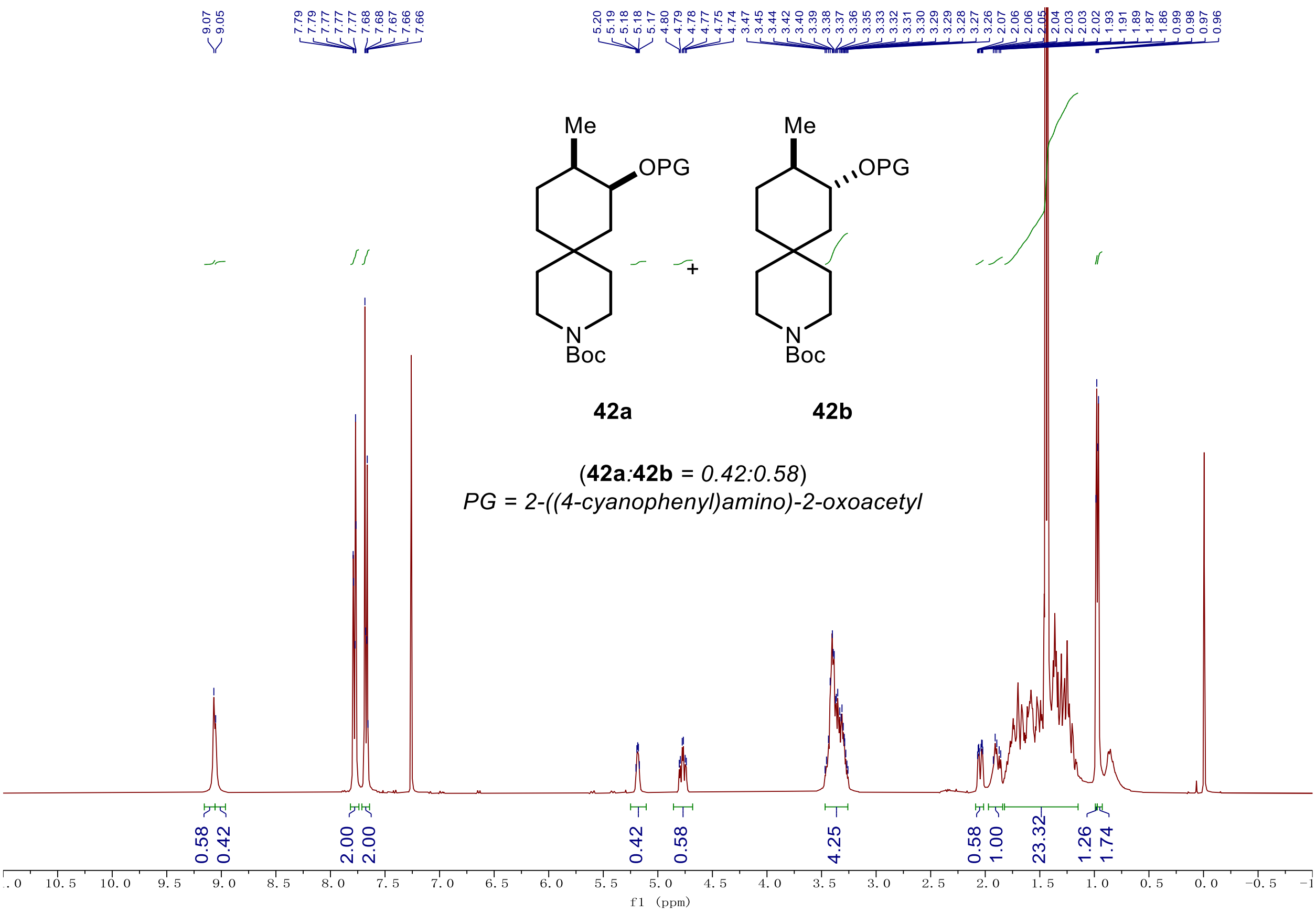

Figure S151 | ^{13}C NMR (101 MHz, $CDCl_3$) spectrum of **42**.

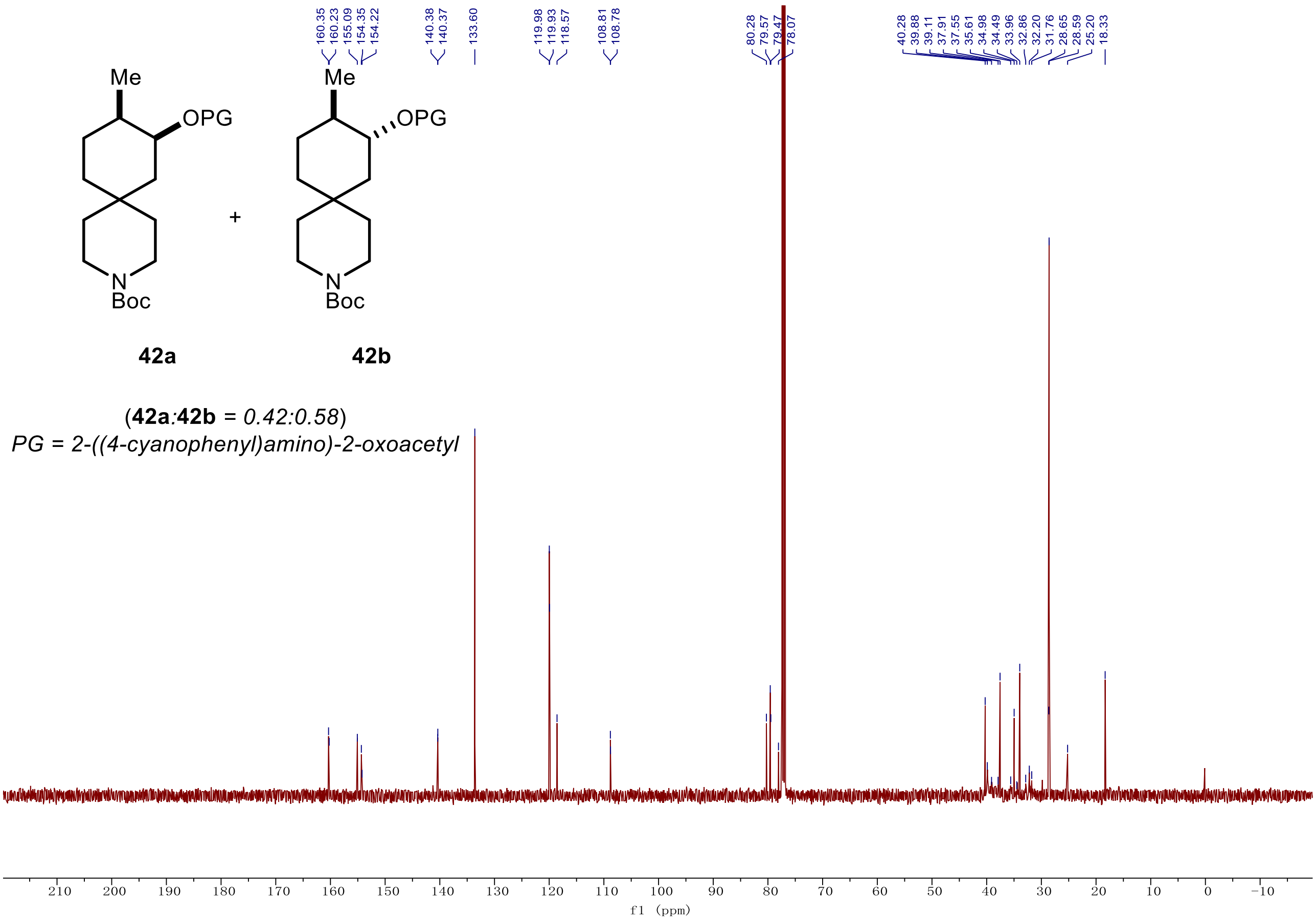

Figure S152 | ^{1}H NMR (500 MHz, $CDCl_3$) spectrum of **44a**.

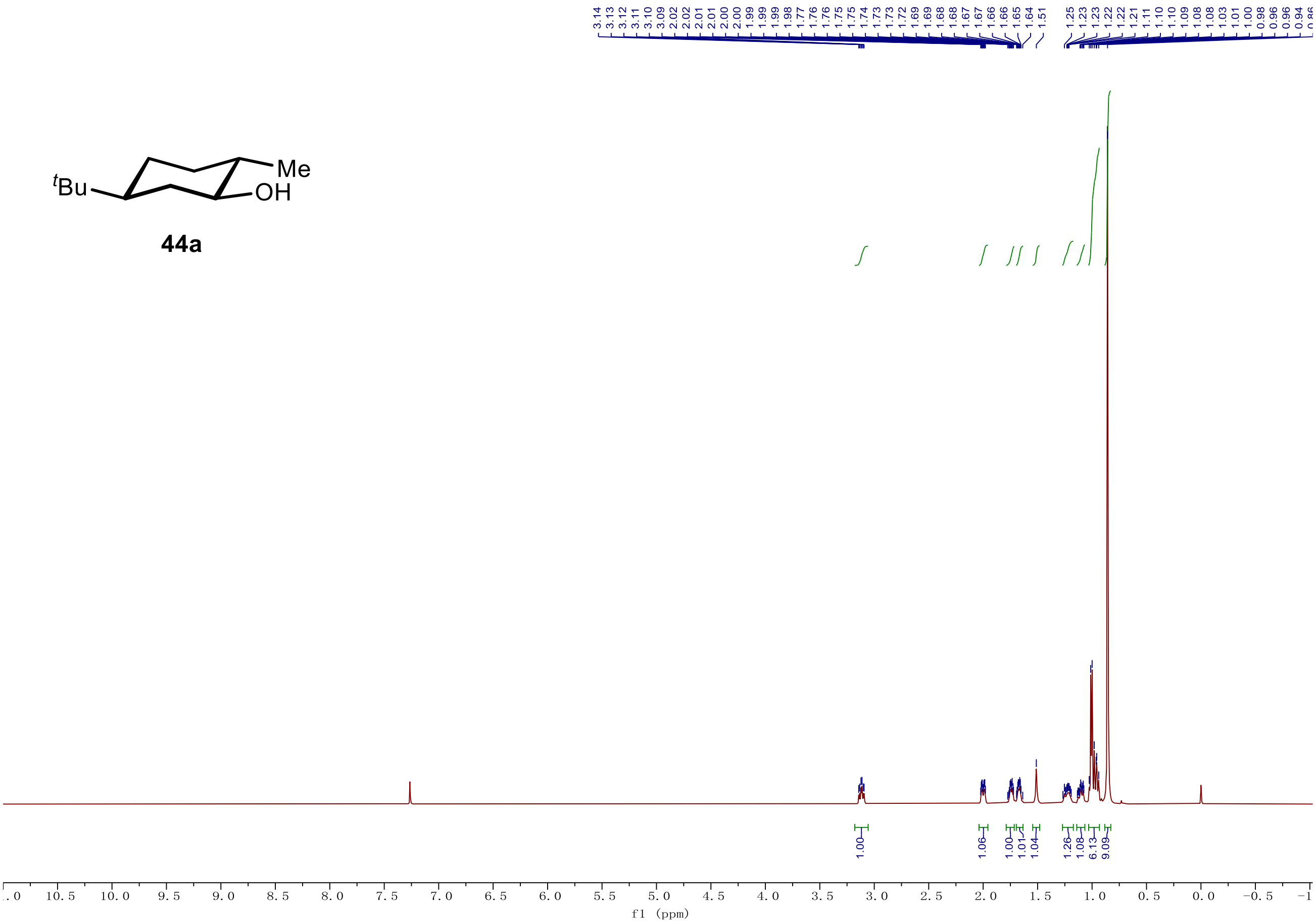

Figure S153 | ^{1}H NMR (500 MHz, C_6D_6) spectrum of **45**.

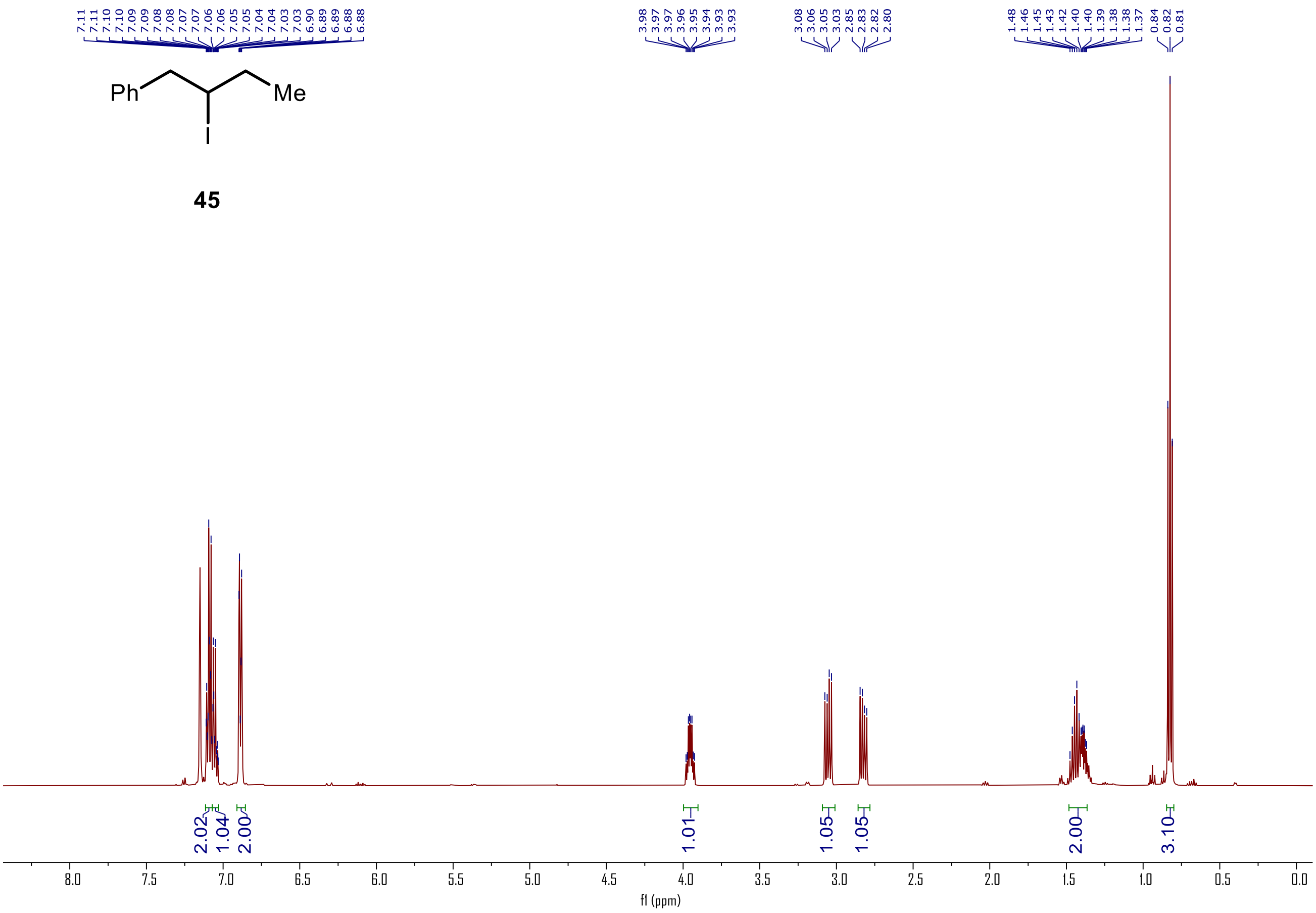

Figure S154 | ^{13}C NMR (101 MHz, C_6D_6) spectrum of **45**.

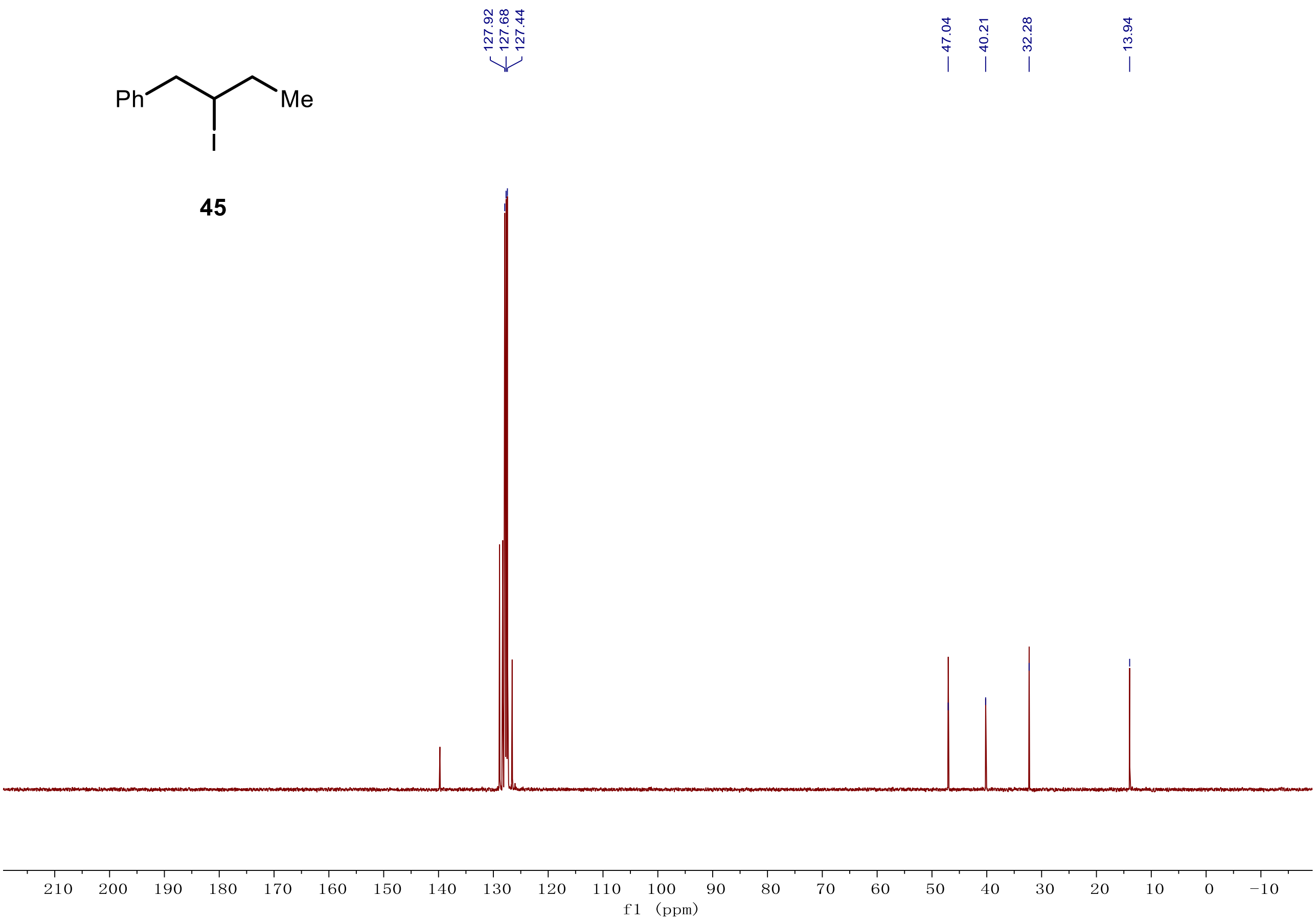

Figure S155 | ^{1}H NMR (400 MHz, $CDCl_3$) spectrum of **46**.

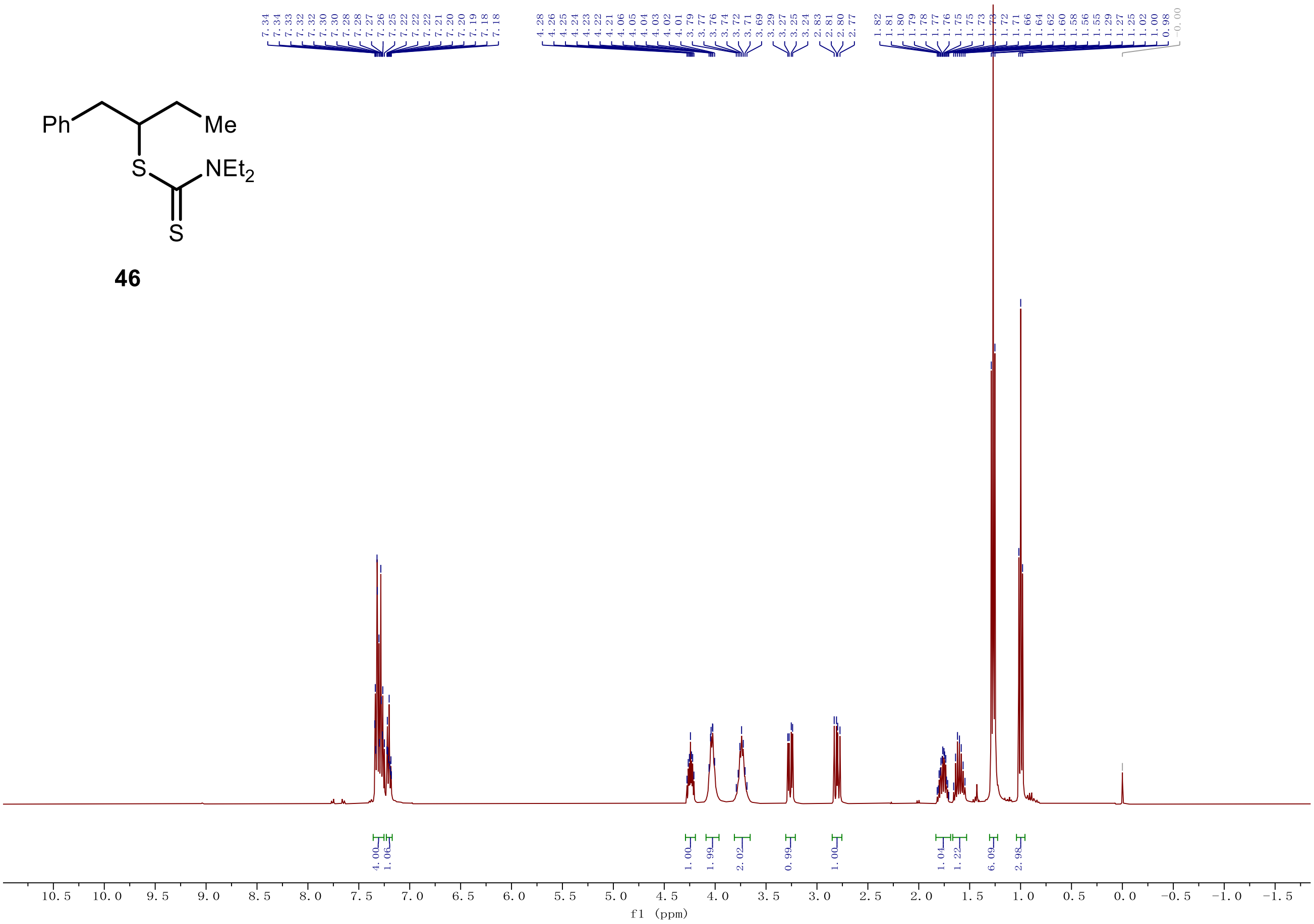

Figure S156 | ^{13}C NMR (101 MHz, $CDCl_3$) spectrum of **46**.

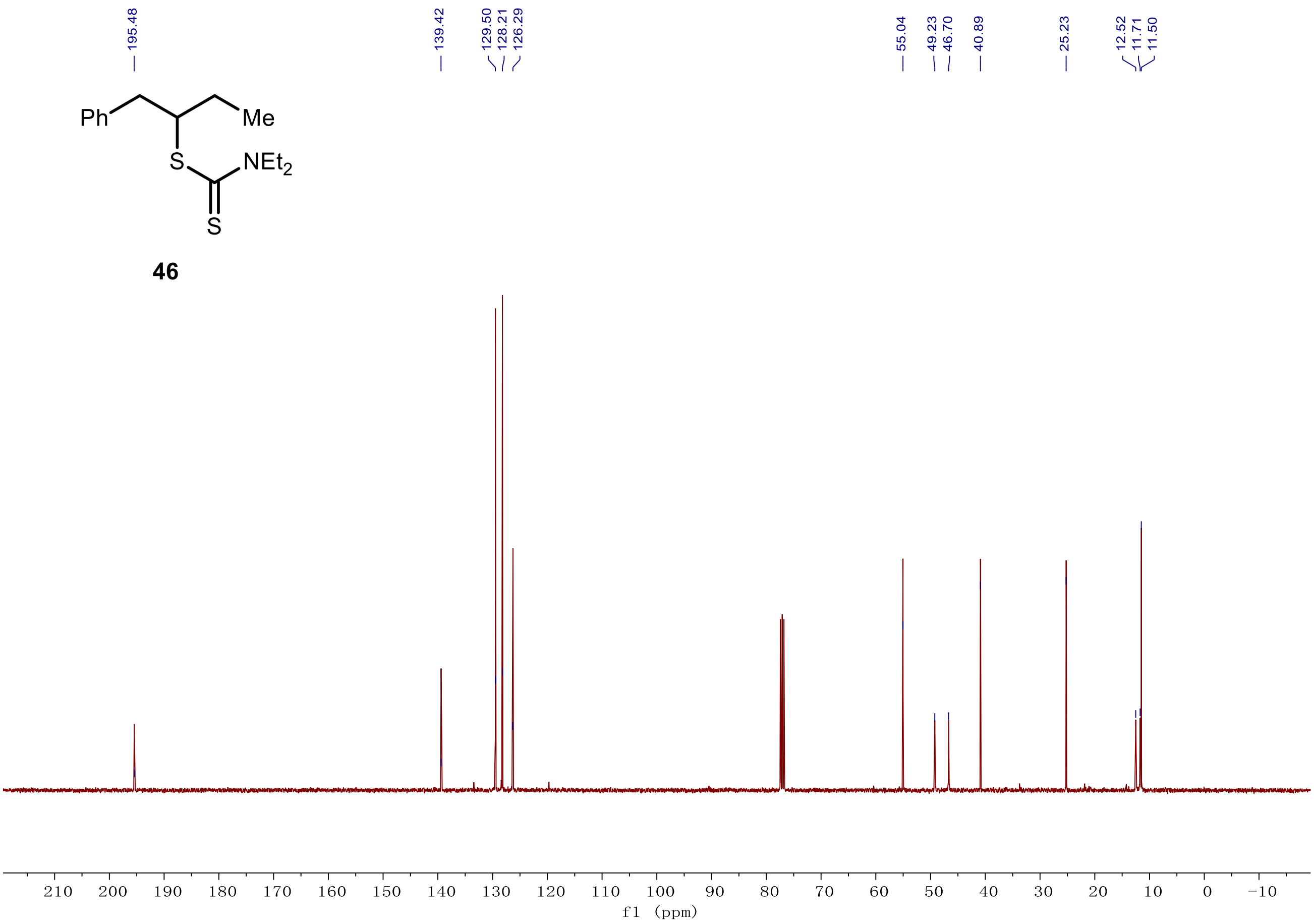

Figure S157 | ^{1}H NMR (400 MHz, $CDCl_3$) spectrum of **49**.

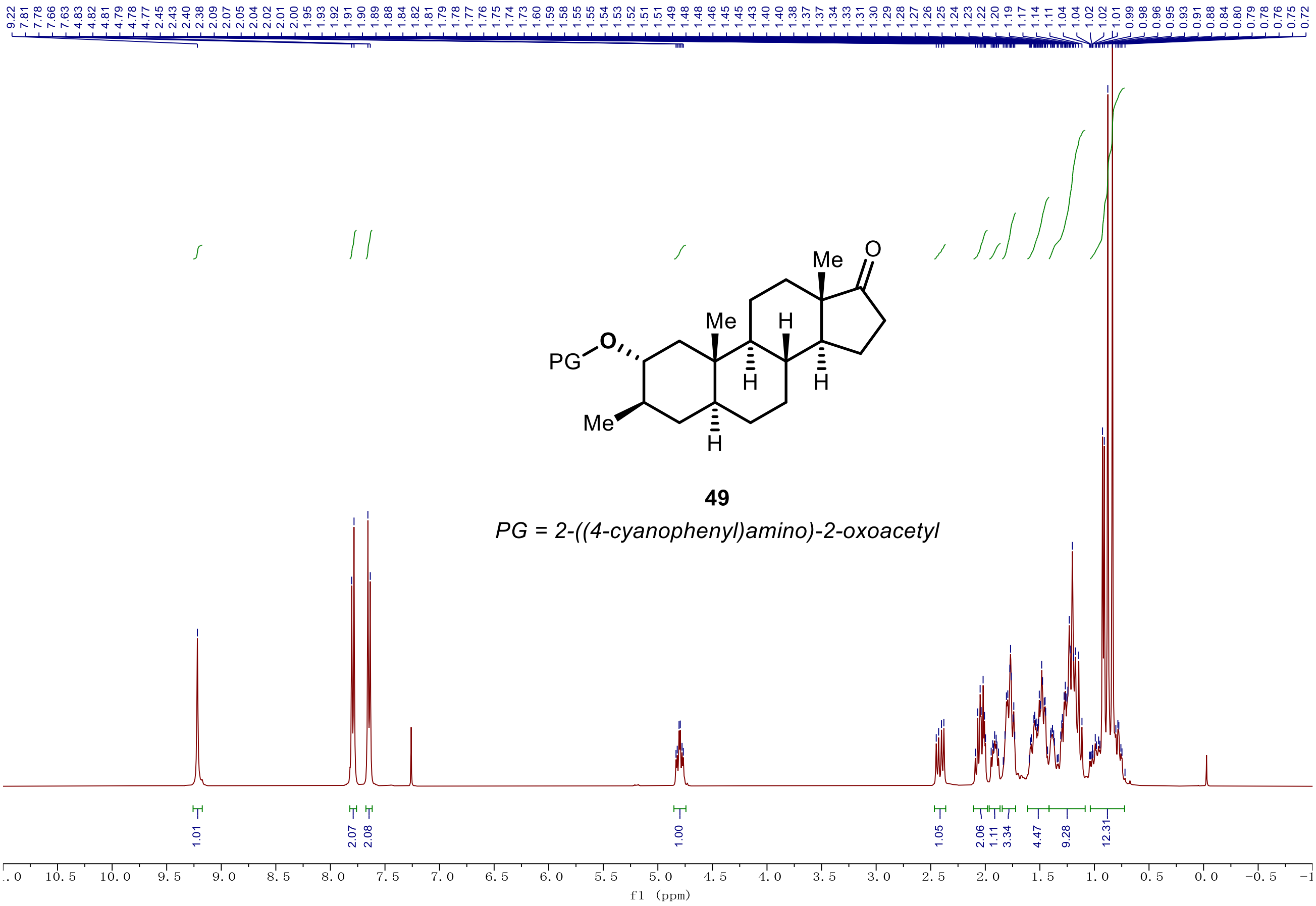

Figure S158 | ^{13}C NMR (101 MHz, $CDCl_3$) spectrum of **49**.

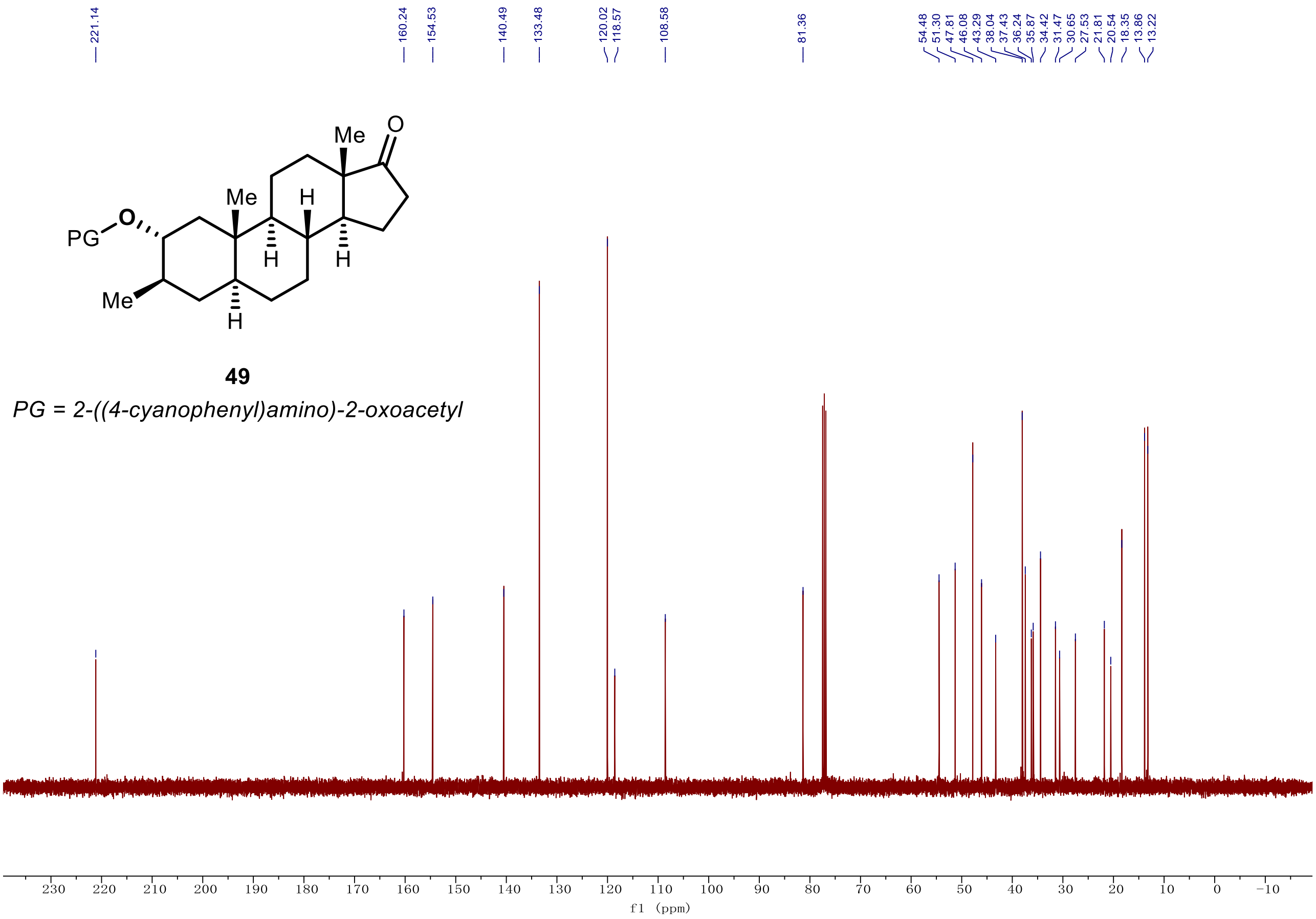

Supplement: Supplementary file 1 [file nwag260_supplemental_file.pdf]
